# Triphenylphosphine Sulfide as an Efficient Ligand in CuAAC to Synthesize 4-Phosphinyl-1,2,3-Triazoles

**DOI:** 10.3390/molecules31214005

**Published:** 2026-11-06

**Authors:** Jie Shen, Jinwei Li, Shitang Xu, Yasuhiro Okuda, Zetao Jiang, Bingting Yao, Hui Li, Yinchun Jiao, Zilong Tang, Lifen Peng, Akihiro Orita, Xinhua Xu

**Affiliations:** 1 State Key Laboratory of Chemo/Biosensing and Chemometrics, College of Chemistry and Chemical Engineering, Hunan University, Changsha 410082, China; 2 Department of Applied Chemistry, Okayama University of Science, 1-1 Ridai-cho, Kita-ku, Okayama 700-0005, Japan; 3 Key Laboratory of Theoretical Organic Chemistry and Functional Molecule of Ministry of Education, Key Laboratory of Molecular Design and Green Chemistry of Hunan Provincial Universities, Hunan Provincial Key Laboratory of Controllable Preparation and Functional Application of Fine Polymers, School of Chemistry and Chemical Engineering, Hunan University of Science and Technology, Xiangtan 411201, China

**Keywords:** triphenylphosphine sulfide, ligand, 4-phosphinyl-1,2,3-triazole, 4-phosphanyl-1,2,3-triazole, CuAAC

## Abstract

Triphenylphosphine sulfide ligand (**L1**) exhibited excellent catalytic performance in the copper-catalyzed azide–alkyne cycloaddition (CuAAC) of ethynyldiphenylphosphine oxide (**1**) and azides (**2**), affording functionalized 4-phosphinyl-1,2,3-triazoles (**3**) in good yields. **L1** showed superior performance to triphenylphosphine oxide, triphenylphosphine, and the sulfide ligands examined, possibly owing to suitable coordination of its P=S moiety to Cu. And, the products could be transformed to 4-phosphanyl-1,2,3-triazoles (**4**) and 1-benzyl-substituted 1,2,3-triazoles (**5**) by reduction and dephosphinylation, respectively. This [Cu]/**L1**-catalyzed protocol, the first example of the application of triphenylphosphine sulfide as a ligand in CuAAC, featured the following advantages: low catalyst loading, relatively short reaction time, broad functional group tolerance, high yields, and facile scalability.

## 1. Introduction

**Figure 1:**
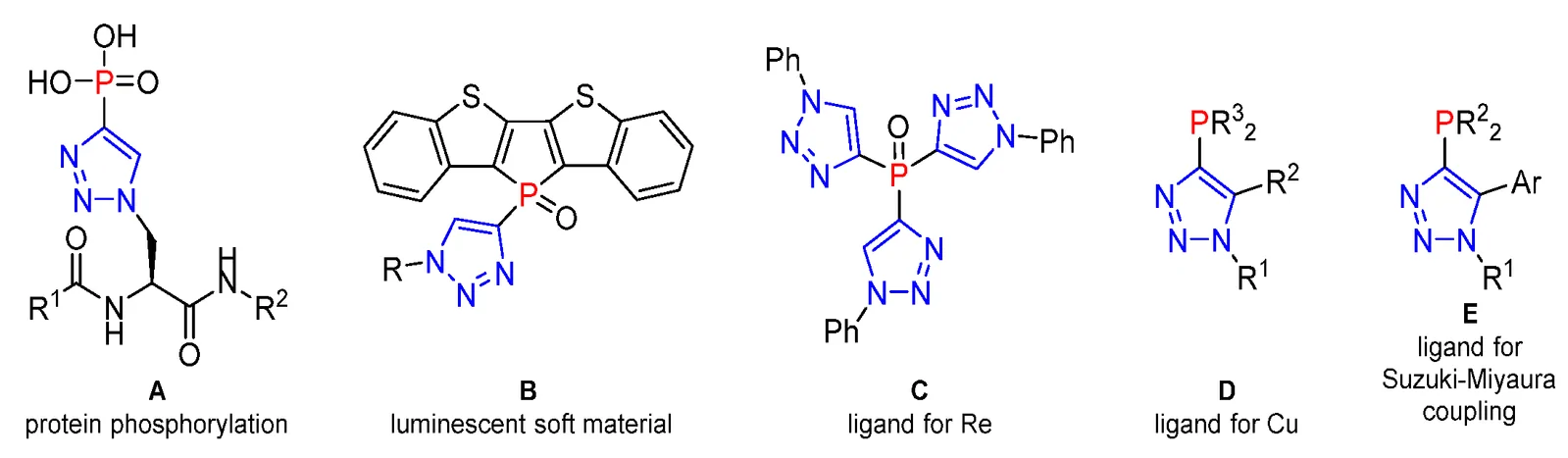
The usefulness of phosphorus-involved 1,2,3-triazoles (**A**–**E**).

1,4-substituted 1,2,3-triazoles, important heterocyclic compounds, are not only important starting materials and intermediates in organic synthesis [1], but also have potential applications in the fields of photoswitch [2], electroluminescent devices [3], liquid crystal [4], biochemistry [5] and drugs [6,7]. The organophosphorus compounds, which could be transformed into various functional molecules through the Horner–Wadsworth–Emmons (HWE) reaction [8], cross-coupling [9], cyclization [10], Wittig reaction [11] and so on, are frequently employed as ligands [12,13,14] or organocatalysts [15,16] in several organic transformations. In addition, organophosphorus materials have extensive applications in the fields of organic electronics [17] and pesticides [18]. Phosphorus-involved 1,2,3-triazoles, possessing the characteristics of 1,2,3-triazoles and organophosphorus compounds, exhibit diverse applications. For example, 4-phosphinyl-1,2,3-triazole (**A**) with a peptide moiety could be used in protein phosphorylation [19], bis(benzothieno)-functionalized 4-phosphinyl-1,2,3-triazole (**B**) is a self-assembled luminescent soft material [20], tris(1,2,3-triazolyl)phosphineoxide (**C**) is a tripodal ligand for Re [21], 4-phosphanyl-1,2,3-triazole (**D**) could be coordinated with Cu to form a luminescent material [22], and 4-phosphanyl-1,2,3-triazole (**E**) is an efficient ligand for Suzuki–Miyaura coupling (Figure 1) [23].

**Scheme 1:**
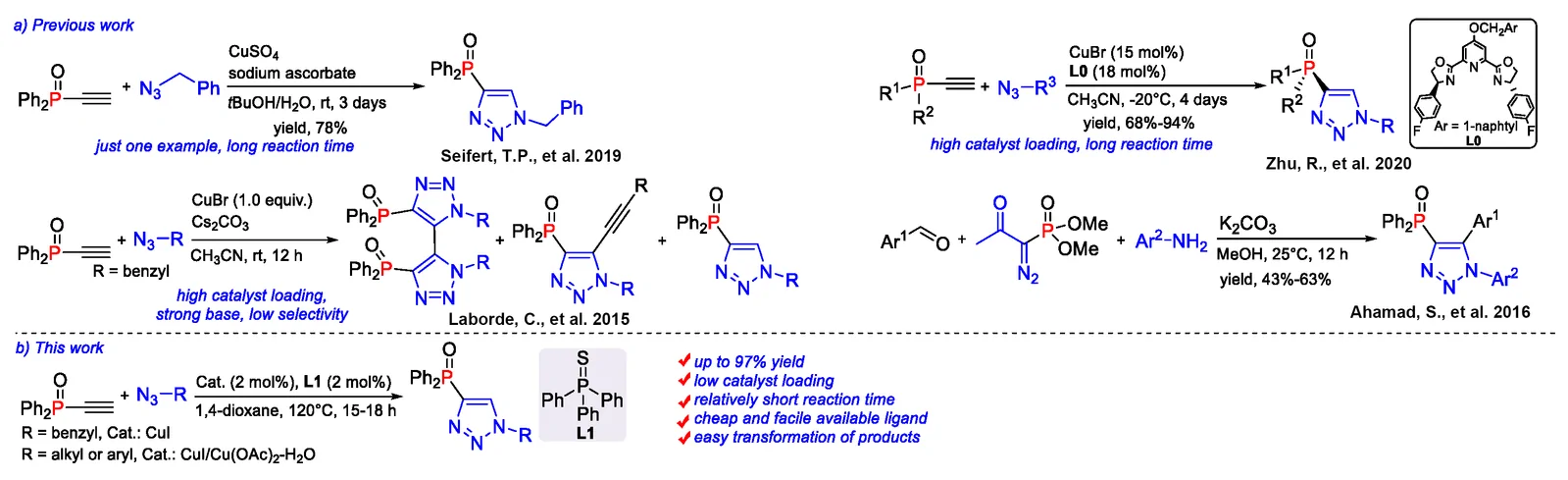
Synthesis of 4-phosphinyl-1,2,3-triazoles (**a**,**b**) [26,27,28,29].

Copper-catalyzed azide–alkyne cycloaddition (CuAAC), which displayed low activation energy and ensured excellent regioselectivity especially for electrophilically activated alkynes [24,25], was frequently utilized to synthesize phosphorus-involved 1,2,3-triazoles. As shown in Scheme 1a, CuSO_4_ catalyzed CuAAC of ethynyldiphenylphosphine oxide and (azidomethyl)benzene afforded the desired 4-phosphinyl-1,2,3-triazole in 78% yield [26], CuBr-promoted CuAAC gave 4-phosphinyl-1,2,3-triazoles in reasonable yields at −20 °C using modified 2,6-bis[(4*S*)-phenyl-2-oxazolin-2-yl]pyridine (PYBOX) as a ligand (**L0**) [27], and CuBr-accelerated CuAAC of ethynyldiphenylphosphine oxide and (azidomethyl)benzene yielded a mixture of bis(triazole), ethyl-substituted triazole and mono(triazole) [28]. Although these approaches produced 4-phosphinyl-1,2,3-triazoles successfully, several drawbacks existed, including limited substrate scope, high catalyst loading and long reaction time. Additionally, three-component domino reactions of aldehydes, the Bestmann–Ohira reagent and amines formed the corresponding 4-phosphinyl-1,2,3-triazoles in moderate yields in the presence of K_2_CO_3_, but just aryl aldehydes and aryl amines were employed [29]. Therefore, a new and efficient synthetic protocol for 4-phosphinyl-1,2,3-triazoles is highly desirable. Herein, triphenylphosphine sulfide was found to be a readily available, inexpensive and an efficient ligand (**L1**) for the CuAAC reaction of ethynyldiphenylphosphine oxide with azides. Using CuI/**L1** as a catalytic system, the reaction between ethynyldiphenylphosphine oxide and substituted benzyl azides proceeded smoothly to generate the 4-phosphinyl-1,2,3-triazoles in good yields. For the CuAAC reaction of alkyl or aryl azides, the dual-copper catalytic system CuI//Cu(OAc)_2_-H_2_O/**L1** was required to achieve high yields (Scheme 1b).

## 2. Results and Discussion

Initially, the performance of different ligands in the CuAAC reaction of ethynyldiphenylphosphine oxide (**1**) and benzyl azide (**2a**) was examined (Table 1). Among triphenylphosphine sulfide (**L1**), triethylphosphine sulfide (**L2**) and *O*,*O*,*O*-triethylthiophosphate (**L3**), **L1** displayed the highest catalytic activity, affording the desired **3a** in 66% yield (entry 1 vs. 2 and 3). **L1** also showed better performance than triphenylphosphine oxide (**L4**) and triphenylphosphine (**L5**) (entry 1 vs. 4 and 5). The sulfide ligands (**L6**–**L9**) generated **3a** in 18–30% yields (entries 6–9). These results suggested that coordination of the P=S moiety to Cu may play an important role in the catalytic system. When other copper catalysts (CuBr, CuCl, Cu(OAc)_2_-H_2_O, CuSO_4_, CuCl_2_) were applied, the yields were between 41% and 54%, which were lower than that of CuI (entries 10–14 vs. 1). The solvent also had a critical effect on the reaction, and 1,4-dioxane was the best solvent for this reaction among toluene, DMF, DMSO and THF (entry 1 vs. entries 15–18). The catalyst loading, temperature (detected in the oil bath) and reaction time also had an impact on the yield. After the reaction had been carefully investigated, the following optimized reaction conditions were obtained: a mixture of **1** (1.0 mmol), **2a** (1.3 mmol), CuI (0.02 mmol) and **L1** (0.02 mmol) in 1,4-dioxane (5 mL) at 120 °C under a nitrogen atmosphere for 15 h, giving **3a** in 90% yield (0.3234 g) (entry 19). In a gram-scale synthesis, 2.2621g (10.0 mmol) of **1** gave 3.1624 g (8.8 mmol) of **3a** in 88% yield (entry 19, Figure S1, Supplementary Materials).

**Scheme 2:**
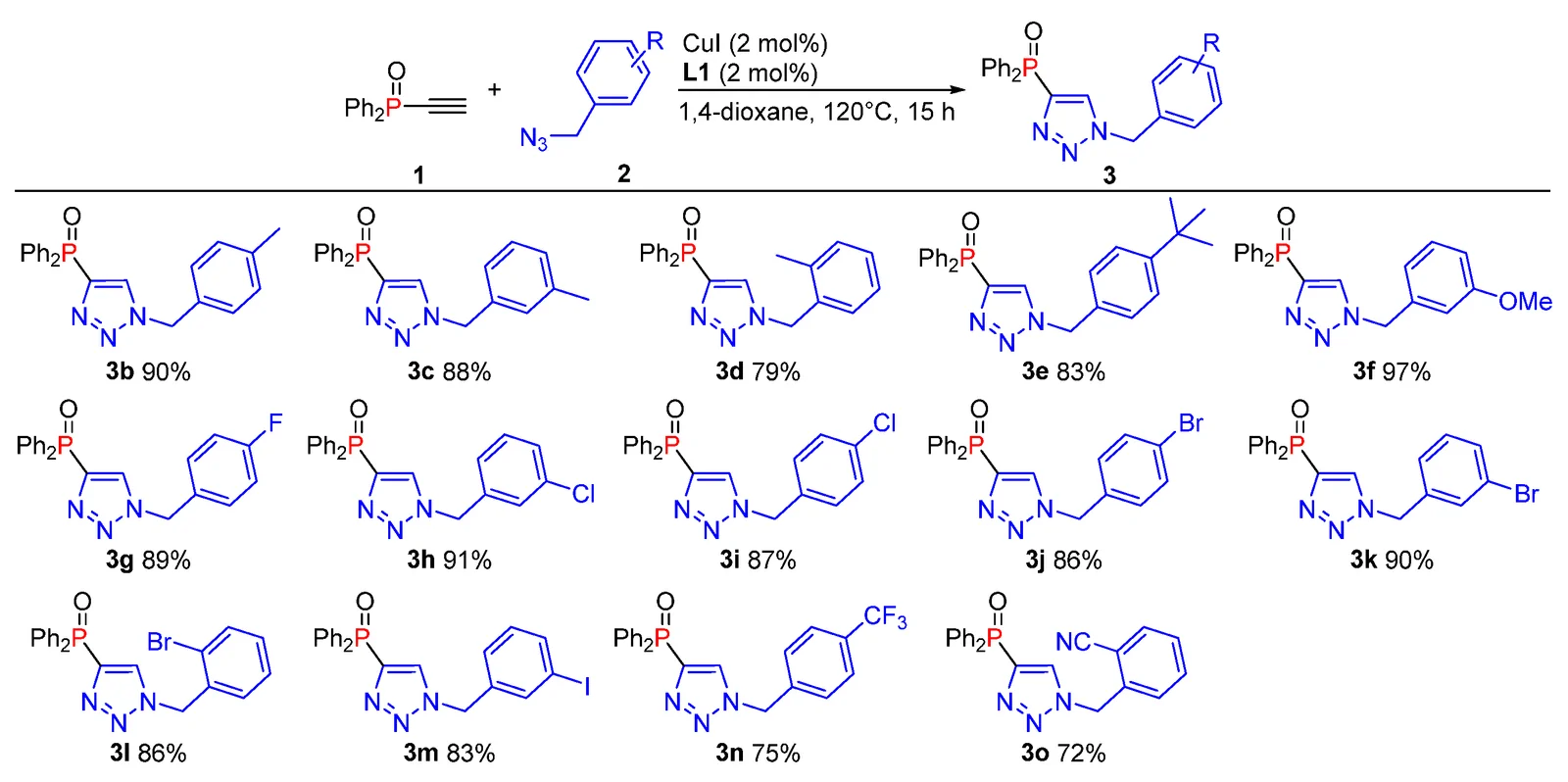
CuI/**L1** catalyzed CuAAC reaction of **1** with benzyl azides.

We then extended the scope of benzyl azides. As shown in Scheme 2, all of the para-, meta-, and ortho-methyl-substituted benzyl azides were suitable for this procedure and afforded the desired 4-phosphinyl-1,2,3-triazoles (**3b**–**3d**) in good-to-excellent yields. In the case of benzyl azides with either electron-donating (*p*-*t*Bu, *m*-OMe) groups, products (**3e**, **3f**) were efficiently produced in 83% and 97% yields, respectively. When benzyl azides with electron-withdrawing (*p*-F, *m*-Cl, *o*-Cl, *p*-Cl, *p*-Br, *m*-Br, *o*-Br, *m*-I, *p*-CF_3_ and *o*-CN) groups were subjected to this CuAAC reaction with **1**, the desired **3g**–**3o** were obtained in 72%–91% yields.

The benzyl, alkyl, and phenyl azides demonstrated notably different reactivities and turnover numbers (TONs) in CuAAC reactions [30,31,32]. When hexyl azide (**2p**) was subjected to the CuAAC with **1** at 120 °C in the presence of CuI and **L1** in 1,4-dioxane, the desired product **3p** was afforded in 36% yield (Table 2, entry 1). Increasing the catalyst loading to 5 mol% improved the yield of **3p** to 55% (entry 2). When the CuAAC between **1** and **2p** was conducted using a dual-copper catalytic system (CuI//Cu(OAc)_2_-H_2_O/**L1**) with the catalyst loading of 2 mol%, the desired cycloaddition was remarkably improved, producing **3p** in 89% yield in 15 h (entry 3). Prolonging the reaction time slightly increased the yield of **3p** (entries 3–5). After detailed optimization of the reaction conditions, the optimum conditions were determined as follows: a mixture of **1** (1.0 mmol), **2p** (1.3 mmol), CuI (0.02 mmol), Cu(OAc)_2_-H_2_O (0.02 mmol) and **L1** (0.02 mmol) in 1,4-dioxane (5 mL) at 120 °C under a nitrogen atmosphere for 18 h, giving **3p** in 92% yield (0.3251 g) (entry 4).

**Scheme 3:**
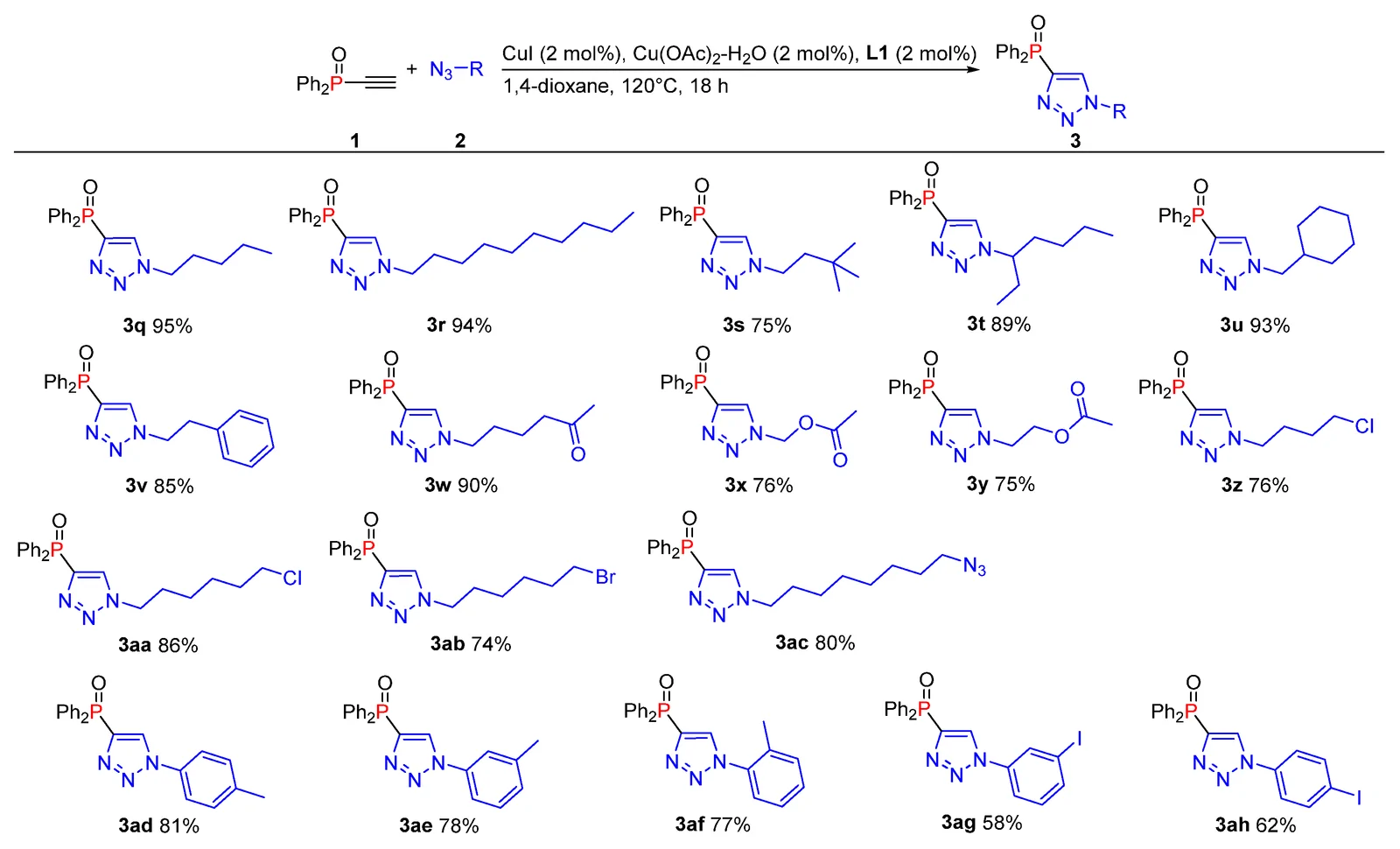
CuI//Cu(OAc)_2_-H_2_O/**L1**-catalyzed CuAAC reaction of **1** with alkyl and phenyl azides.

Under the above optimum conditions, a variety of alkyl and phenyl azides underwent this cycloaddition smoothly to generate functionalized 4-phosphinyl-1,2,3-triazoles in reasonable yields (Scheme 3). Primary and secondary alkyl azides were both well tolerated, affording the desired products **3q**–**3v** in 75–95% yields. In the case of alkyl azides with carbonyl, ester, chloro or bromo groups, products **3w**–**3ab** were efficiently produced in good-to-excellent yields. Notably, the diazide was applicable, forming the corresponding **3ac** with an unreacted azido in 80% yield. The protocol was also compatible with diverse phenyl azides with either electron-donating (*p*-Me, *m*-Me, *o*-Me) or electron-withdrawing (*m*-I, *p*-I) groups, generating the desired products **3ad**–**3ah** in 58–81% yields.

**Scheme 4:**
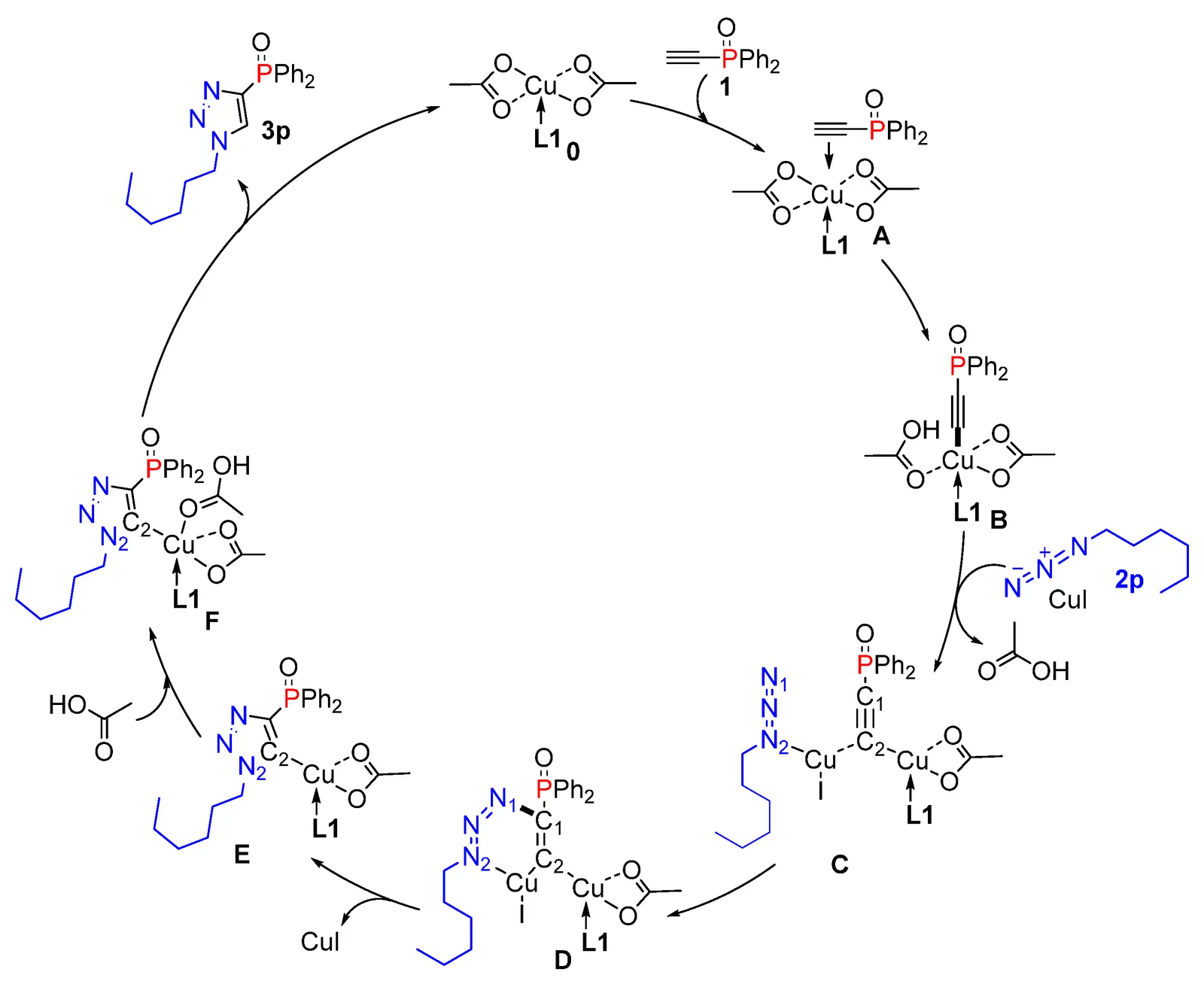
The proposed mechanism.

A plausible mechanism for the above CuI/Cu(OAc)_2_-H_2_O/**L1** catalytic system was deduced. Initially, active **L1**-Cu(OAc)_2_ (**0**) was formed by coordination of Cu(OAc)_2_ to ligand (**L1**). Coordination of **0** with ethynyldiphenylphosphine oxide (**1**) generated the intermediate **A**. Abstraction of a H atom from ethyne by OAc in **A** produced **B**, which was then transformed to the di-copper intermediate **C** through incorporating with CuI and **2p** and the elimination of HOAc. The six-membered metallacycle **D** could be obtained by the intramolecular cyclization of **C**. Forming of C2–N2 bond in **D** produced the five-membered cycle intermediate **E** with the elimination of CuI. Intermediate **F** was then yielded via coordination of HOAc with **E**. The H transformation in **F** afforded the desired **3p** and regenerated the active **0** (Scheme 4). The suitable coordination of P=S moiety in **L1** with copper was critical to enhance the activity of copper, possibly due to the formation of the more-stable six-membered metallacycle **D**, which then lowered the activation energy of this CuAAC reaction. The regioselective formation of 4-phosphinyl-1,2,3-triazole (**3p**) might be due to the facile coordination of **2p** and ethyne moiety in **B** with CuI.

**Scheme 5:**
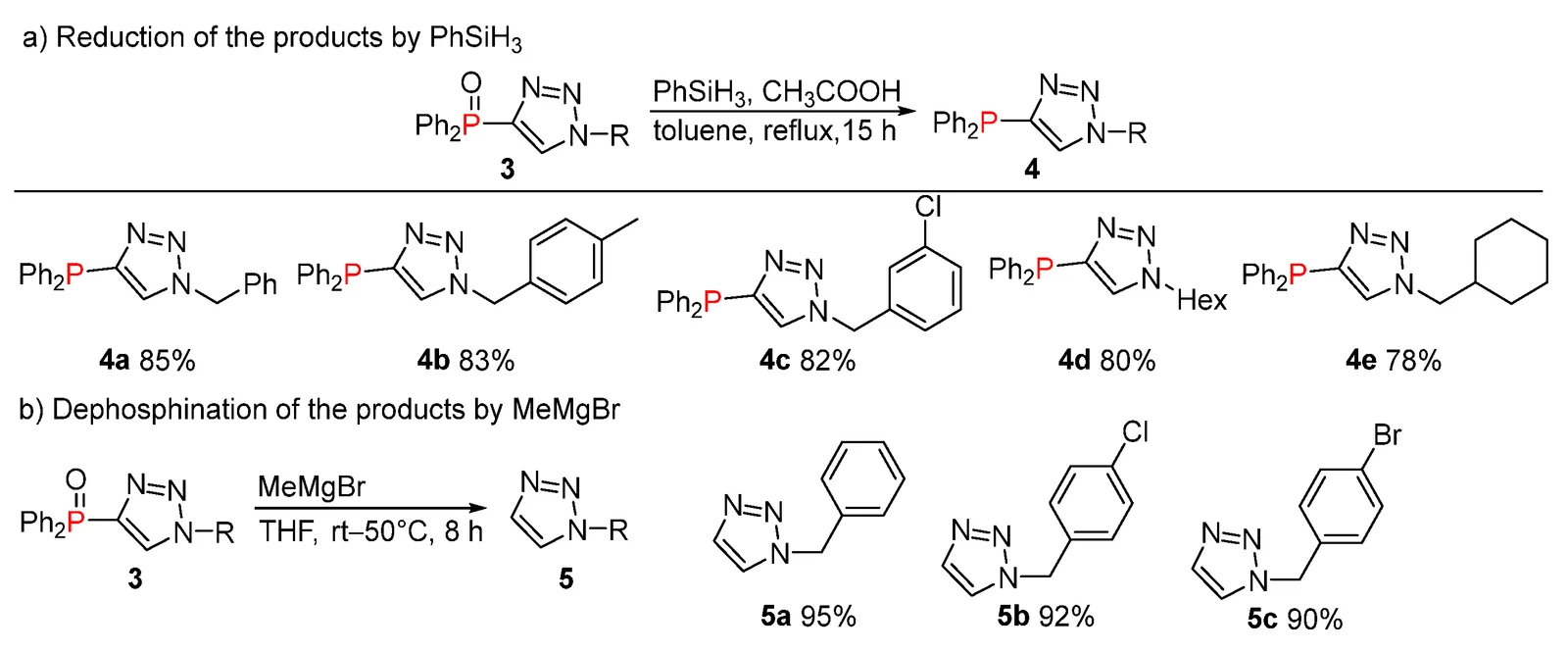
Transformation of products (**a**,**b**).

Two chemical conversions of cycloaddition products (**3**) were conducted to demonstrate their chemical usability (Scheme 5). The 4-phosphinyl-1,2,3-triazoles prepared above could be transformed into 4-phosphanyl-1,2,3-triazoles by reduction with PhSiH_3_ [33]. In this transformation, both of the benzyl- and alkyl-substituted 4-phosphinyl-1,2,3-triazoles were tolerated, resulting in the formation of the corresponding 4-phosphanyl-1,2,3-triazoles (**4a**–**4e**) in good yields. The 4-phosphinyl-1,2,3-triazoles (**3a**, **3i** and **3j**) were transformed to the corresponding 1-benzyl-substituted 1,2,3-triazoles (**5a**–**5c**) in excellent yields via the MeMgBr-promoted dephosphinylation.

## 3. Materials and Methods

Unless otherwise noted, all reactions were carried out under a N_2_ atmosphere. All reagents were commercially available and utilized without any further purification. Glass apparatus was dried in an oven and heated under vacuum before use. Analytical thin layer chromatography (TLC) was performed on Haiyang TLC silica gel GF254 (0.25 mm, from Qingdao Hailang Silica Gel Desiccant Co., Ltd., Qingdao, China) plates. Column chromatography was implemented on silica gel (300–400 mesh) with a suitable eluent solvent. ^1^H, ^13^C, ^31^P and ^19^F NMR spectra were recorded on a Bruker-400 MHz spectrometer from Bruker Switzerland AG, Fällanden, Switzerland (^1^H: 400 MHz, ^13^C: 101 MHz, ^31^P: 121 MHz, and ^19^F: 376 MHz) using the solvent resonances as internal standards (^1^H NMR: CDCl_3_, δ 7.26 ppm; ^13^C NMR: CDCl_3_, δ 77.00 ppm; ^31^P NMR: CDCl_3_; ^19^F NMR: CDCl_3_). Chemical shifts are reported in ppm (δ) relative to internal tetramethylsilane (TMS). Data are reported as follows: chemical shift, multiplicity (s = singlet, d = doublet, t = triplet, q = quartet, m = multiplet); coupling constants (*J*) are reported in hertz. Melting points were measured using a melting point meter RY-1G. Crystal Data were acquired at 296 K on a Rigaku Oxford Diffraction Supernova Dual Source, Cu at zero equipped with an Atlas S2 CCD using Cu Kα radiation. HRMS data were acquired using a Waters Xevo G2-XS QT of mass spectrometer with electrospray ionization (ESI). Ethynyldiphenylphosphine oxide (**1**) and benzyl, alkyl, and phenyl azides were prepared according to the reported methods [26].

General Procedure for Synthesis of 4-phosphinyl-1,2,3-triazoles (**3a**–**3o**). A Schlenk tube of 25 mL equipped with a magnetic stir bar was charged with **1** (0.2262 g, 1.0 mmol), azide **2** (1.3 mmol), CuI (0.0038 g, 0.02 mmol), **L1** (0.0059 g, 0.02 mmol) and 1,4-dioxane (5 mL) under nitrogen. The mixture was stirred at 120 °C for 15 h. After quenching with aqueous NH_4_Cl, the aqueous layer was extracted with AcOEt three times. The combined organic layers were washed with brine and dried over MgSO_4_. After filtration, the solvent was removed under reduced pressure. The crude product was subjected to column chromatography on silica gel (PE/AcOEt, 1:1) to afford 4-phosphinyl-1,2,3-triazoles (**3a**–**3o**) in pure form.

(1-benzyl-1H-1,2,3-triazol-4-yl)diphenylphosphine oxide (**3a**): prepared from **1** (0.2262 g, 1.0 mmol) and azide **2a** (0.1731, 1.3 mmol) as a white solid in 90% yield (0.3234 g). Melting point (M.P.): 158–160 °C; ^1^H NMR (400 MHz, CDCl_3_) *δ* 8.09 (s, CH, 1H), 7.91–7.84 (m, 4H), 7.56–7.49 (m, 2H), 7.48–7.42 (m, 4H), 7.41–7.35 (m, 3H), 7.33–7.27 (m, 2H), 5.57 (s, CH_2_, 2H). ^13^C NMR (101 MHz, CDCl_3_) *δ* 133.39, 132.33 (d, *J* = 110.8 Hz), 132.08 (d, *J* = 2.4 Hz), 131.41 (d, *J* = 10.4 Hz), 130.87, 130.64, 129.25, 129.10, 128.52, 128.40, 54.33 (benzyl C). ^31^P NMR (162 MHz, CDCl_3_) *δ* 16.93. HRMS-ESI (m/z) [M+H^+^] Calcd for C_21_H_19_N_3_OP 360.1266; found, 360.1268.

Gram-scale synthesis of **3a**: A Schlenk tube of 250 mL equipped with a magnetic stir bar was charged with **1** (2.2621 g, 10.0 mmol), azide **2a** (1.7310 g, 13.0 mmol), CuI (0.0381 g, 0.2 mmol), **L1** (0.0589 g, 0.2 mmol) and 1,4-dioxane (50 mL) under nitrogen, after stirring at 120 °C for 15 h. After the usual workup with EtOAc and NH_4_Claq, the combined organic layer was washed with brine, and dried over MgSO_4_. After filtration, the solvents were evaporated. The crude product was subjected to column chromatography on silica gel (PE/AcOEt, 1:1) to afford **3a** as a white solid in 88% yield (3.1624 g).

General Procedure for the Synthesis of 4-phosphinyl-1,2,3-triazoles (**3p**–**3ah**). A Schlenk tube of 25 mL equipped with a magnetic stir bar was charged with **1** (0.2262 g, 1.0 mmol), azide **2** (1.3 mmol), CuI (0.0038 g, 0.02 mmol), Cu(OAc)_2_-H_2_O (0.02 mmol), **L1** (0.0059 g, 0.02 mmol) and 1,4-dioxane (5 mL) under nitrogen. The mixture was stirred at 120 °C for 18 h. After quenching with aqueous NH_4_Cl, the aqueous layer was extracted with AcOEt three times. The combined organic layers were washed with brine and dried over MgSO_4_. After filtration, the solvent was removed under reduced pressure. The crude product was subjected to column chromatography on silica gel (PE/AcOEt, 1:1) to afford 4-phosphinyl-1,2,3-triazoles (**3p**–**3ah**) in pure form.

(1-hexyl-1H-1,2,3-triazol-4-yl)diphenylphosphine oxide (**3p**): prepared from **1** (0.2262 g, 1.0 mmol) and azide **2p** (0.1653 g, 1.3 mmol) as a white solid in 92% yield (0.3251 g). Melting point (M.P.): 76–79 °C; ^1^H NMR (400 MHz, CDCl_3_) *δ* 8.18 (s, 1H), 7.88–7.83 (m, 4H), 7.51–7.47 (m, 2H), 7.45–7.40 (m, 4H), 4.38 (t, *J* = 7.3 Hz, 2H), 1.92–1.85 (m, 2H), 1.31–1.23 (m, 6H), 0.83 (t, *J* = 7.6 Hz, 2H). ^13^C NMR (101 MHz, CDCl_3_) *δ* 141.40 (d, *J* = 134.3 Hz), 132.27 (d, *J* = 111.1 Hz), 131.92, 131.26 (d, *J* = 12.1 Hz), 130.60 (d, *J* = 24.2 Hz), 128.32 (d, *J* = 12.1 Hz), 50.34, 30.86, 29.89, 25.88, 22.15, 13.72. ^31^P NMR (162 MHz, CDCl_3_) *δ* 16.79. HRMS-ESI (m/z) [M+H^+^] Calcd for C_20_H_25_N_3_OP 354.1735; found, 354.1738.

General Procedure for the Synthesis of 4-phosphanyl-1,2,3-triazoles (**4a**–**4e**). A Schlenk tube of 25 mL equipped with a magnetic stir bar was charged with **3** (0.5 mmol), PhSiH_3_ (0.2164 g, 2.0 mmol), CH_3_COOH (0.3003 g, 5.0 mmol), and toluene (5 mL) under nitrogen. The mixture was stirred under refluxing for 15 h. After quenching with aqueous NH_4_Cl, the aqueous layer was extracted with AcOEt three times. The combined organic layers were washed with brine and dried over MgSO_4_. After filtration, the solvent was removed under reduced pressure. The crude product was subjected to column chromatography on silica gel (PE/AcOEt, 6:1) to afford 4-phosphanyl-1,2,3-triazoles (**4a**–**4e**) in pure form.

1-benzyl-4-(diphenylphosphanyl)-1H-1,2,3-triazole (**4a**): prepared from **3a** (0.1797 g, 0.5 mmol) as a white solid in 85% yield (0.1459 g). Melting point (M.P.): 78–88 °C; ^1^H NMR (400 MHz, CDCl_3_) *δ* 7.46–7.41 (m, 4H), 7.36–7.35 (m, 4H), 7.32–7.31 (m, 6H), 7.24–7.22 (m, 2H), 5.52 (s, 2H). ^13^C NMR (101 MHz, CDCl_3_) *δ* 144.82 (d, *J* = 6.1 Hz), 136.26 (d, *J* = 7.1 Hz), 134.47, 133.40 (d, *J* = 20.2 Hz), 131.43 (d, *J* = 10.1 Hz), 129.27 (d, *J* = 26.3 Hz), 128.97 (d, *J* = 18.2 Hz), 128.69, 128.45 (d, *J* = 8.1 Hz), 127.91, 53.91. ^31^P NMR (162 MHz, CDCl_3_) *δ* −31.86. HRMS-ESI (m/z) [M+H^+^] Calcd for C_21_H_19_N_3_P 344.1317; found, 344.1319.

General Procedure for the Synthesis of 1-benzyl-substituted 1,2,3-triazoles (**5a**–**5c**). A Schlenk tube of 25 mL equipped with a magnetic stir bar was charged with **3** (1.0 mmol), MeMgBr (1.5 mmol) and THF (5 mL) under nitrogen. The mixture was stirred at 50 °C for 8 h. After quenching with aqueous NH_4_Cl, the aqueous layer was extracted with AcOEt three times. The combined organic layers were washed with brine and dried over MgSO_4_. After filtration, the solvent was removed under reduced pressure. The crude product was subjected to column chromatography on silica gel (PE/AcOEt, 4:1) to afford 1-benzyl-substituted 1,2,3-triazoles (**5a**–**5c**) in pure form.

1-benzyl-1H-1,2,3-triazole (**5a**): prepared from **3a** (0.3594 g, 1.0 mmol) as a white solid in 95% yield (0.1512 g). ^1^H NMR (400 MHz, CDCl_3_) *δ* 7.64 (s, 1H), 7.41 (s, 1H), 7.33–7.29 (m, 3H), 7.20–7.18 (m, 2H), 5.50 (s, 2H). ^13^C NMR (101 MHz, CDCl_3_) *δ* 134.64, 134.19, 129.06, 128.68, 127.96, 123.28, 53.92.

## 4. Conclusions

In summary, triphenylphosphine sulfide (**L1**) could be employed as an inexpensive, stable and efficient ligand for CuAAC reactions of ethynyldiphenylphosphine oxide (**1**) with azides (**2**), affording functionalized 4-phosphinyl-1,2,3-triazoles (**3**). Using CuI/**L1** as a catalytic system, the CuAAC between **1** and benzyl azides proceeded smoothly under low catalyst loading and relatively short reaction time conditions, giving the corresponding 4-phosphinyl-1,2,3-triazoles (**3a**–**3o**) in high yields. The dual-copper catalytic system CuI/Cu(OAc)_2_-H_2_O/**L1** efficiently promoted the reactions of alkyl and aryl azides, generating 4-phosphinyl-1,2,3-triazoles (**3p**–**3ah**) in reasonable yields. The products (**3**) could be converted to 4-phosphanyl-1,2,3-triazoles (**4**) by reduction with PhSiH_3_ and to 1-benzyl-substituted 1,2,3-triazoles (**5**) by MeMgBr-accelerated dephosphinylation. Compared to the reported CuAAC for synthesis of 4-phosphinyl-1,2,3-triazoles, our [Cu]/**L1**-catalyzed protocol exhibited notable advantages such as a low catalyst loading, relatively short reaction time, a broad substrate scope and high yields.

## Figures and Tables

**Table 1:** Optimization of the reaction conditions of CuAAC between **1** and **2a** ^a^.

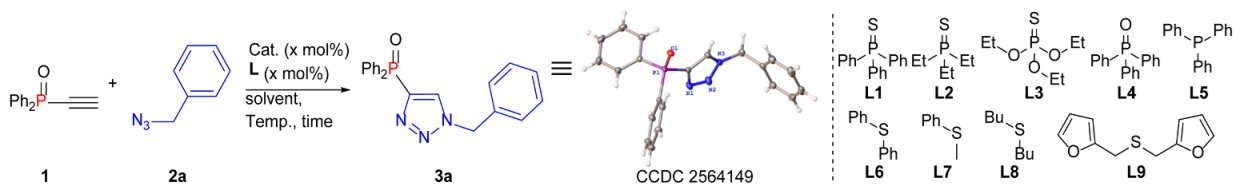								
Entry	Cat.	x	L	y	Solvent	Temp. (°C)	Time (h)	Yield (%)
1	CuI	1	**L1**	1	1,4-dioxane	120	12	66
2	CuI	1	**L2**	1	1,4-dioxane	120	12	45
3	CuI	1	**L3**	1	1,4-dioxane	120	12	40
4	CuI	1	**L4**	1	1,4-dioxane	120	12	31
5	CuI	1	**L5**	1	1,4-dioxane	120	12	23
6	CuI	1	**L6**	1	1,4-dioxane	120	12	25
7	CuI	1	**L7**	1	1,4-dioxane	120	12	20
8	CuI	1	**L8**	1	1,4-dioxane	120	12	18
9	CuI	1	**L9**	1	1,4-dioxane	120	12	30
10	CuBr	1	**L1**	1	1,4-dioxane	120	12	54
11	CuCl	1	**L1**	1	1,4-dioxane	120	12	52
12	Cu(OAc)_2_-H_2_O	1	**L1**	1	1,4-dioxane	120	12	48
13	CuSO_4_	1	**L1**	1	1,4-dioxane	120	12	46
14	CuCl_2_	1	**L1**	1	1,4-dioxane	120	12	41
15	CuI	1	**L1**	1	toluene	120	12	48
16	CuI	1	**L1**	1	DMF	120	12	57
17	CuI	1	**L1**	1	DMSO	120	12	55
18	CuI	1	**L1**	1	THF	70	12	39
19	CuI	2	**L1**	2	1,4-dioxane	120	15	90 (88 ^b^)

^a^ **1** (1.0 mmol), **2a** (1.3 mmol), solvent (5 mL), isolated yield. ^b^ **1** (10.0 mmol), **2a** (13.0 mmol), solvent (50 mL), isolated yield.

**Table 2:** Optimization of the reaction conditions of CuAAC between **1** and **2p** ^a^.

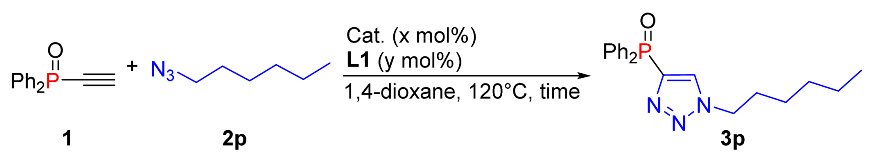					
Entry	Cat.	x	y	Time (h)	Yield (%)
1	CuI	2	2	15	36
2	CuI	5	5	15	55
3	CuI/Cu(OAc)_2_-H_2_O	2	2	15	89
4	CuI/Cu(OAc)_2_-H_2_O	2	2	18	92
5	CuI/Cu(OAc)_2_-H_2_O	2	2	19	90
6	CuI/Cu(OAc)_2_-H_2_O	3	3	18	91

^a^ **1** (1.0 mmol), **2p** (1.3 mmol), 1,4-dioxane (5 mL), isolated yield.

## Data Availability

The original contributions presented in this study are included in the article and Supplementary Materials. Further inquiries can be directed to the corresponding authors.

## Supplementary Materials

The following supporting information can be downloaded at https://www.mdpi.com/article/10.3390/molecules31214005/s1. Figure S1: ORTEP drawing of 3a; Table S1: Crystal data and structure refinement for **3a**. Refs. [34,35,36,37,38,39] are cited in the Supplementary Materials.
