# Supplementary material for: Triphenylphosphine Sulfide as an Efficient Ligand in CuAAC to Synthesize 4-Phosphinyl-1,2,3-Triazoles
Source: Molecules. 2026 Nov 6;31(21):4005. doi: 10.3390/molecules31214005 (PMC13764721; doi:10.3390/molecules31214005)
Supplement: Supplementary file 1 [file molecules-31-04005-s001.zip › molecules-4646550-supplementary.pdf]

Supporting Information

Triphenylphosphine Sulfide as an Efficient Ligand in CuAAC to Synthesize 4-Phosphinyl-1,2,3-Triazoles

Jie Shen[1], Jinwei Li[1], Shitang Xu[1], Yasuhiro Okuda[3], Zetao Jiang[2], Bingting Yao[1], Hui Li[2], Yinchun Jiao[2], Zilong Tang[2], Lifen Peng[2,*], Akihiro Orita[3,*] and Xinhua Xu[1,*]

[1]State Key Laboratory of Chemo/Biosensing and Chemometrics, College of Chemistry and Chemical Engineering, Hunan University, Changsha 410082, China; jieshen@hnu.edu.cn (J.S.); lijinwei@hnu.edu.cn (J.L.); xst978@hnu.edu.cn (S.X.); Y3011226292@hnu.edu.cn (B.Y.); huil23@hnu.edu.cn (H.L.)

[2]Key Laboratory of Theoretical Organic Chemistry and Functional Molecule of Ministry of Education, Key Laboratory of Molecular Design and Green Chemistry of Hunan Provincial Universities, Hunan Provincial Key Laboratory of Controllable Preparation and Functional Application of Fine Polymers, School of Chemistry and Chemical Engineering, Hunan University of Science and Technology, Xiangtan 411201, China; 24010601012@mail.hnust.edu.cn (Z.J.); yinchunjiao@hnust.edu.cn (Y.J.); zltang@hnust.edu.cn (Z.T.)

[3]Department of Applied Chemistry, Okayama University of Science, 1-1 Ridai-cho, Kita-ku, Okayama 700-0005, Japan.

*Correspondence: 1060137@hnust.edu.cn (L.P.); orita@ous.ac.jp (A.O.); xhx1581@hnu.edu.cn (X.X.)

Table of Contents

1. General Information 1

2. General Procedure for Synthesis of 4-Phosphinyl-1,2,3-Triazoles (3a−3o)................................1

3. General Procedure for Synthesis of 4-Phosphinyl-1,2,3-Triazoles (3o−3ah)..............................6

4. General Procedure for Synthesis of 4-Phosphanyl-1,2,3-Triazoles (4a−4e) 13

5. General Procedure for Synthesis of 1-Benzyl-Substituted 1,2,3-Triazoles (5a−5c) 15

1. General Information

All reagents were commercially available and used without further purification. Glassware was dried in an oven and heated under reduced pressure before use. Column chromatography was undertaken on silica gel (300-400 mesh) using a proper eluent system. Analytical thin layer chromatography (TLC) was performed on Haiyang TLC silica gel GF254 (0.25 mm) plates. ^{1}H, ^{13}C, ^{31}P and ^{19}F NMR spectra were recorded on a Bruker-400 MHz spectrometer (^{1}H: 400 MHz, ^{13}C: 101 MHz, ^{31}P: 121 MHz, and ^{19}F: 376 MHz) using the solvent resonances as internal standards (^{1}H NMR: $CDCl_3$, δ 7.26 ppm; ^{13}C NMR: $CDCl_3$, δ 77.00 ppm; ^{31}P NMR: $CDCl_3$; ^{19}F NMR: $CDCl_3$). Chemical shifts are reported in ppm (δ) relative to internal tetramethylsilane (TMS). Data are reported as follows: chemical shift, multiplicity (s = singlet, d = doublet, t = triplet, q = quartet, m = multiplet); coupling constants (*J*) are reported in hertz. Melting points were measured using a melting point meter RY-1G. Crystal Data were acquired at 296 K on a Rigaku Oxford Diffraction Supernova Dual Source, Cu at zero equipped with an Atlas S2 CCD using Cu Kα radiation. HRMS data were acquired using a Waters Xevo G2-XS QTof mass spectrometer with electrospray ionization (ESI). Ethynyldiphenylphosphine oxide (1), benzyl, alkyl, and phenyl azides were prepared according to the reported methods[1].

2. General Procedure for Synthesis of 4-phosphinyl-1,2,3-triazoles (3a–3o)

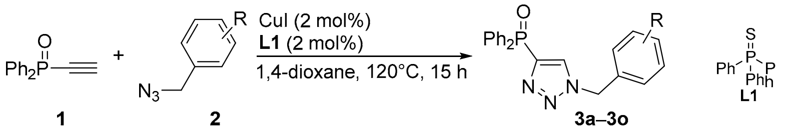

A Schlenk tube of 25 mL equipped with a magnetic stir bar was charged with **1** (0.2262 g, 1.0 mmol), azide **2** (1.3 mmol), CuI (0.0038 g, 0.02 mmol), **L1** (0.0059 g, 0.02 mmol) and 1,4-dioxane (5 mL) under nitrogen. And the mixture was stirred at 120°C for 15 h. After usual workup with EtOAc and NH_4Claq, the combined organic layer was washed with brine, and dried over $MgSO_4$. After filtration, the solvents were evaporated. The crude product was subjected to column chromatography on silica gel (PE/AcOEt, 1:1) to afford 4-phosphinyl-1,2,3-triazoles (**3a**–**3o**) in a pure form.

(1-benzyl-1*H*-1,2,3-triazol-4-yl)diphenylphosphine oxide (3a):[3] prepared from **1** (0.2262 g, 1.0 mmol) and azide **2a** (0.1731, 1.3 mmol) as a white solid in 90% yield (0.3234 g).

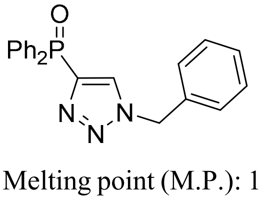

Melting point (M.P.): 158−160°C; ^{1}H NMR (400 MHz, $CDCl_3$) *δ* 8.09 (s, 1H), 7.91−7.84 (m, 4H), 7.56 −7.49 (m, 2H), 7.48−7.42 (m, 4H), 7.41−7.35 (m, 3H), 7.33−7.27 (m, 2H), 5.57 (s, 2H). ^{13}C NMR (101 MHz, $CDCl_3$) *δ* 133.39, 132.33 (d, *J* = 110.8 Hz), 132.08 (d, *J* = 2.4 Hz), 131.41 (d, *J* = 10.4 Hz), 130.87, 130.64, 129.25, 129.10, 128.52, 128.40, 54.33. ^{31}P NMR (162 MHz, $CDCl_3$) δ 16.93. HRMS-

ESI (m/z) [M+H$^+$] Calcd for $C_{21}H_{19}N_3OP$ 360.1266; found, 360.1268.

Gram-scale synthesis of 3a: A Schlenk tube of 250 mL equipped with a magnetic stir bar was charged with **1** (2.2621 g, 10.0 mmol), azide **2a** (1.7310 g, 13.0 mmol), CuI (0.0381 g, 0.2 mmol), **L1** (0.0589 g, 0.2 mmol) and 1,4-dioxane (50 mL) under nitrogen, after stirring at 120°C for 15 h. After usual workup with EtOAc and NH_4Claq, the combined organic layer was washed with brine, and dried over $MgSO_4$. After filtration, the solvents were evaporated. The crude product was subjected to column chromatography on silica gel (PE/AcOEt, 1:1) to afford **3a** as a white solid in 88% yield (3.1624 g).

(1-(4-methylbenzyl)-1H-1,2,3-triazol-4-yl)diphenylphosphine oxide (3b):[3] prepared from **1** (0.2262 g, 1.0 mmol) and azide **2b** (0.1913, 1.3 mmol) as a white solid in 90% yield (0.3360 g).

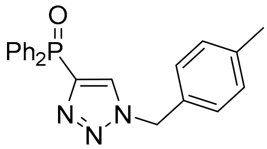

Melting point (M.P.): 152−154 °C; ^{1}H NMR (400 MHz, $CDCl_3$) δ 8.07 (s, 1H), 7.91−7.83 (m, 4H), 7.55−7.49 (m, 2H), 7.48−7.41 (m, 4H), 7.23−7.14 (m, 4H), 5.52 (s, 2H), 2.34 (s, 3H). ^{13}C NMR (101 MHz, $CDCl_3$) δ 141.75 (d, J = 136.4 Hz), 138.95, 132.22 (d, J = 110.7 Hz), 131.96 (d, J = 2.8 Hz), 131.29 (d, J = 11.1 Hz), 131.59 (d, J = 24.2 Hz), 130.30, 129.76, 128.44 (d, J = 5.0 Hz), 128.28, 53.99, 21.01. ^{31}P NMR (162 MHz, $CDCl_3$) δ 16.75. HRMS-ESI (m/z) [M+H$^+$] Calcd for $C_{22}H_{21}N_3OP$ 374.1422; found, 374.1421.

(1-(3-methylbenzyl)-1H-1,2,3-triazol-4-yl)diphenylphosphine oxide (3c): prepared from **1** (0.2262 g, 1.0 mmol) and azide **2c** (0.1913, 1.3 mmol) as a white solid in 88% yield (0.3286 g).

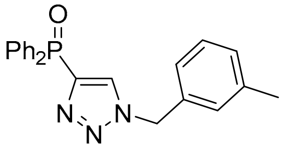

Melting point (M.P.): 160−162°C; ^{1}H NMR (400 MHz, $CDCl_3$) δ 8.10 (s, 1H), 7.87−7.82 (m, 4H), 7.48−7.45 (m, 2H), 7.42−7.38 (m, 4H), 7.20 (t, J = 6.0 Hz, 1H), 7.12 (d, J = 7.6 Hz, 1H), 7.07−7.05 (m, 2H), 5.48 (s, 2H), 2.28 (s, 3H). ^{13}C NMR (101 MHz, $CDCl_3$) δ 142.96 (d, J = 136.4 Hz), 138.85, 131.18, 132.14 (d, J = 110.1 Hz), 131.90 (d, J = 3.0 Hz), 131.20 (d, J = 10.1 Hz), 130.63 (d, J = 24.2 Hz), 129.61, 129.09, 128.90, 128.28 (d, J = 13.1 Hz), 125.44, 54.09, 21.08. ^{31}P NMR (162 MHz, $CDCl_3$) δ 16.71. HRMS-ESI (m/z) [M+H$^+$] Calcd for $C_{22}H_{21}N_3OP$ 374.1422; found, 374.1425.

(1-(2-methylbenzyl)-1H-1,2,3-triazol-4-yl)diphenylphosphine oxide (3d):[3] prepared from **1** (0.2262 g, 1.0 mmol) and azide **2d** (0.1913, 1.3 mmol) as a white solid in 79% yield (0.2950 g).

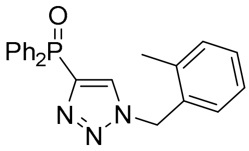

Melting point (M.P.): 189−192°C; ^{1}H NMR (400 MHz, $CDCl_3$) *δ* 7.99 (s, 1H), 7.89−7.84 (m, 4H), 7.54−7.50 (m, 2H), 7.48−7.43 (m, 4H), 7.33−7.29 (m, 1H), 7.25−7.20 (m, 3H), 5.59 (s, 2H), 2.27 (s, 3H). ^{13}C NMR (101 MHz, $CDCl_3$) *δ* 141.82 (d, J = 136.4 Hz), 137.04, 132.34 (d, *J* = 110.8 Hz), 132.10 (d, *J* = 2.8 Hz), 131.42 (d, *J* = 10.5 Hz), 131.31, 131.23, 130.55 (d, *J* = 24.2 Hz), 130.02, 129.60, 128.48 (d, *J* = 12.8 Hz), 126.84, 52.45, 18.96. ^{31}P NMR (162 MHz, $CDCl_3$) *δ* 16.84. HRMS-ESI (m/z) $[M+H^+]$ Calcd for $C_{22}H_{21}N_3OP$ 374.1422; found, 374.1426.

(1-(4-(tert-butyl)benzyl)-1H-1,2,3-triazol-4-yl)diphenylphosphine oxide (3e): prepared from **1** (0.2262 g, 1.0 mmol) and azide **2e** (0.2460, 1.3 mmol) as a white solid in 83% yield (0.3448 g).

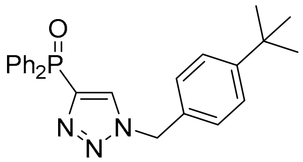

Melting point (M.P.): 210−213°C; ^{1}H NMR (400 MHz, $CDCl_3$) *δ* 8.10 (s, 1H), 7.89−7.79 (m, 4H), 7.49−7.33 (m, 8H), 7.20 (d, *J* = 7.0 Hz, 2H), 5.48 (s, 2H), 1.27 (s, 9H). ^{13}C NMR (101 MHz, $CDCl_3$) *δ* 152.07, 141.64 (d, *J* = 136.6 Hz), 132.24 (d, *J* = 110.7 Hz), 131.92 (d, *J* = 2.8 Hz), 131.26 (d, *J* = 10.5 Hz), 130.78, 130.54, 130.23, 128.32 (d, *J* = 13.1 Hz), 126.03, 53.84, 34.45, 31.03. ^{31}P NMR (162 MHz, $CDCl_3$) *δ* 16.74. HRMS-ESI (m/z) $[M+H^+]$ Calcd for $C_{25}H_{27}N_3OP$ 416.1892; found, 416.1895.

(1-(3-methoxybenzyl)-1H-1,2,3-triazol-4-yl)diphenylphosphine oxide (3f): prepared from **1** (0.2262 g, 1.0 mmol) and azide **2f** (0.2121, 1.3 mmol) as a white solid in 97% yield (0.3777 g).

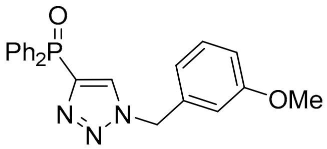

Melting point (M.P.): 156−159°C; ^{1}H NMR (400 MHz, $CDCl_3$) *δ* 8.11 (s, 1H), 7.90−7.85 (m, 4H), 7.54−7.50 (m, 2H), 7.47−7.43 (m, 4H), 7.30−7.29 (m, 1H), 6.89 (t, *J* = 8.0 Hz, 2H), 6.82 (s, 1H), 5.53 (s, 2H), 3.78 (s, 3H). ^{13}C NMR (101 MHz, $CDCl_3$) *δ* 160.11, 141.99 (d, *J* = 135.9 Hz), 134.75, 132.23 (d, *J* = 110.9 Hz), 132.09 (d, *J* = 2.7 Hz), 131.40 (d, *J* = 10.4 Hz), 130.91, 130.68, 130.34, 128.46 (d, *J* = 12.8 Hz), 120.66, 114.32 (d, *J* = 32.5 Hz), 55.22, 54.26. ^{31}P NMR (162 MHz, $CDCl_3$) δ 18.31.HRMS-ESI (m/z) $[M+H^+]$ Calcd for $C_{22}H_{21}N_3O_2P$ 390.1371; found, 390.1375.

(1-(4-fluorobenzyl)-1H-1,2,3-triazol-4-yl)diphenylphosphine oxide (3g): prepared from **1** (0.2262 g, 1.0 mmol) and azide **2g** (0.1965, 1.3 mmol) as a white solid in 89% yield (0.3358 g).

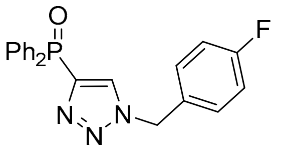

Melting point (M.P.): 165−168°C; ^{1}H NMR (400 MHz, $CDCl_3$) δ 8.16 (s, 1H), 7.89−7.84 (m, 4H), 7.54−7.50 (m, 2H), 7.47−7.42 (m, 4H), 7.31−7.28 (m, 2H), 7.05 (t, J = 8.6 Hz, 2H), 5.54 (s, 2H). ^{13}C NMR (101 MHz, $CDCl_3$) δ 162.88 (d, J = 249.5 Hz), 142.01 (d, J = 131.4 Hz), 132.13 (d, J = 110.9 Hz), 132.09, 131.32 (d, J = 10.5 Hz), 130.71 (d, J = 24.2 Hz), 130.38 (d, J = 8.5 Hz), 129.40, 128.43 (d, J = 12.8 Hz), 116.15 (d, J = 22.2Hz), 53.42. ^{31}P NMR (162 MHz, $CDCl_3$) δ 16.77. ^{19}F NMR (376 MHz, $CDCl_3$) δ 111.99. HRMS-ESI (m/z) [M+H^{+}] Calcd for $C_{21}H_{18}FN_3OP$ 378.1172; found, 378.1176.

(1-(3-chlorobenzyl)-1H-1,2,3-triazol-4-yl)diphenylphosphine oxide (3h): prepared from **1** (0.2262 g, 1.0 mmol) and azide **2h** (0.2179, 1.3 mmol) as a white solid in 91% yield (0.3584 g).

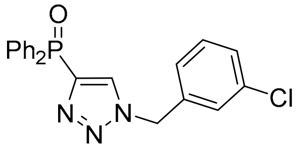

Melting point (M.P.): 185−187°C; ^{1}H NMR (400 MHz, $CDCl_3$) δ 8.15 (s, 1H), 7.90−7.85 (m, 4H), 7.54−7.51 (m, 2H), 7.49−7.46 (m, 4H), 7.35−7.31 (m, 3H), 7.18 (d, J = 7.3 Hz, 1H), 5.55 (s, 2H). ^{13}C NMR (101 MHz, $CDCl_3$) δ 142.37 (d, J = 135.4 Hz), 135.41, 135.07, 132.17 (d, J = 110.9 Hz), 132.13 (d, J = 2.8 Hz), 131.38 (d, J = 10.5 Hz), 130.96, 130.73, 130.51, 129.28, 128.49 (d, J = 12.6 Hz), 126.51, 53.54. ^{31}P NMR (162 MHz, $CDCl_3$) δ 16.66. HRMS-ESI (m/z) [M+H^{+}] Calcd for $C_{21}H_{18}ClN_3OP$ 394.0876; found, 394.0878.

(1-(4-chlorobenzyl)-1H-1,2,3-triazol-4-yl)diphenylphosphine oxide (3i): prepared from **1** (0.2262 g, 1.0 mmol) and azide **2i** (0.2179, 1.3 mmol) as a white solid in 87% yield (0.3426 g).

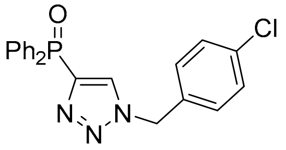

Melting point (M.P.): 164 −166°C; ^{1}H NMR (400 MHz, $CDCl_3$) δ 8.17 (s, 1H), 7.89−7.84 (m, 4H), 7.54−7.50 (m, 2H), 7.46−7.44 (m, 4H), 7.33 (d, J = 7.9 Hz, 2H), 7.23 (d, J = 8.2 Hz, 2H), 5.54 (s, 2H). ^{13}C NMR (101 MHz, $CDCl_3$) δ 142.38 (d, J = 135.6 Hz), 135.40, 135.07, 132.18 (d, J = 110.9 Hz), 132.14 (d, J = 2.8 Hz), 131.39 (d, J = 10.5 Hz), 130.96, 130.72, 130.50, 129.28, 128.48 (d, J = 12.1 Hz), 126.50, 53.54. ^{31}P NMR (162 MHz, $CDCl_3$) δ 16.67. HRMS-ESI (m/z) [M+H^{+}] Calcd for $C_{21}H_{18}ClN_3OP$ 394.0876; found, 394.0873.

(1-(4-bromobenzyl)-1H-1,2,3-triazol-4-yl)diphenylphosphine oxide (3j): prepared from **1** (0.2262 g, 1.0 mmol), azide **2j** (0.2757 g, 1.3 mmol) as a white solid in 86% yield (0.3769 g).

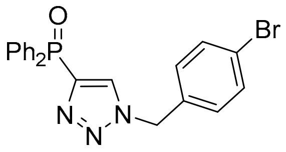

Melting point (M.P.): 169−172°C; ^{1}H NMR (400 MHz, $CDCl_3$) δ 8.15 (s, 1H), 7.89−7.84 (m, 4H), 7.54−7.43 (m, 8H), 7.18 (d, J = 7.8 Hz, 2H), 5.53 (s, 2H). ^{13}C NMR (101 MHz, $CDCl_3$) δ 142.19 (d, J = 135.6 Hz), 132.26, 132.12 (d, J = 110.8 Hz),, 132.07 (d, J = 2.7 Hz), 131.31 (d, J = 10.4 Hz), 130.93, 130.70, 130.00, 128.43 (d, J = 12.8 Hz), 123.15, 53.44. ^{31}P NMR (162 MHz, $CDCl_3$) δ 16.72. HRMS-ESI (m/z) [M+H$^+$] Calcd for $C_{21}H_{18}BrN_3OP$ 438.0371; found, 438.0375.

(1-(3-bromobenzyl)-1H-1,2,3-triazol-4-yl)diphenylphosphine oxide (3k): prepared from **1** (0.2262 g, 1.0 mmol) and azide **2k** (0.2757 g, 1.3 mmol) as a white solid in 90% yield (0.3944 g).

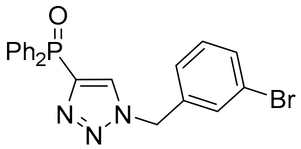

Melting point (M.P.): 188−191°C; ^{1}H NMR (400 MHz, $CDCl_3$) δ 8.16 (s, 1H), 7.90−7.84 (m, 4H), 7.55−7.44 (m, 8H), 7.27−7.21 (m, 2H), 5.54 (s, 2H). ^{13}C NMR (101 MHz, $CDCl_3$) δ 142.24 (d, J = 136.3 Hz), 135.67, 132.13, 132.11, 132.09 (d, J = 110.9 Hz), 132.08, 131.33 (d, J = 10.3 Hz), 130.88 (d, J = 24.2 Hz), 130.70, 128.45 (d, J = 12.8 Hz), 126.95, 123.05, 53.39. ^{31}P NMR (162 MHz, $CDCl_3$) δ 16.76. HRMS-ESI (m/z) [M+H$^+$] Calcd for $C_{21}H_{18}BrN_3OP$ 438.0371; found, 438.0376.

(1-(2-bromobenzyl)-1H-1,2,3-triazol-4-yl)diphenylphosphine oxide (3l): prepared from **1** (0.2262 g, 1.0 mmol) azide **2a** (0.2757 g, 1.3 mmol) as a white solid in 86% yield (0.3769 g).

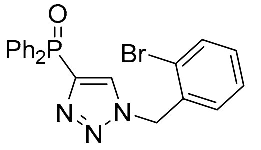

Melting point (M.P.): 194−197°C; ^{1}H NMR (400 MHz, $CDCl_3$) δ 8.18 (s, 1H), 7.93−7.81 (m, 4H), 7.62 (d, J = 8.0 Hz, 1H), 7.56−7.49 (m, 2H), 7.49−7.41 (m, 4H), 7.35−7.29 (m, 1H), 7.29−7.22 (m, 2H), 5.72 (s, 2H). ^{13}C NMR (101 MHz, $CDCl_3$) δ 141.87 (d, J = 136.16 Hz), 133.34, 132.97, 132.20 (d, J = 110.8 Hz), 132.05 (d, J = 2.7 Hz), 131.35 (d, J = 10.5 Hz), 130.97 (d, J = 24.2 Hz), 130.92, 130.77, 128.42 (d, J = 13.1 Hz), 128.22, 123.90, 53.95. ^{31}P NMR (162 MHz, $CDCl_3$) δ 16.84. HRMS-ESI (m/z) [M+H$^+$] Calcd for $C_{21}H_{18}BrN_3OP$ 438.0371; found, 438.0376.

(1-(3-iodobenzyl)-1H-1,2,3-triazol-4-yl)diphenylphosphine oxide (3m): prepared from **1** (0.2262 g, 1.0 mmol) and azide **2m** (0.3368, 1.3 mmol) as a white solid in 83% yield (0.4028 g).

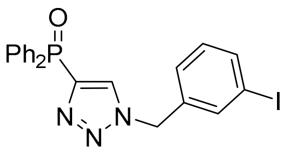

Melting point (M.P.): 171−173°C; ^{1}H NMR (400 MHz, $CDCl_3$) δ 8.16 (s, 1H), 7.90−7.85 (m, 4H),

7.71−7.67 (m, 2H), 7.54−7.51 (m, 2H), 7.48−7.46 (m, 4H), 7.27−7.25 (m, 1H), 7.10 (t, J = 8.0 Hz, 1H), 5.51 (s, 2H). ^{13}C NMR (101 MHz, $CDCl_3$) δ 142.21 (d, J = 135.6 Hz), 138.05, 137.21, 132.09 (d, J = 2.8 Hz), 132.08 (d, J = 110.9 Hz), 131.33 (d, J = 10.5 Hz), 130.97, 130.78, 130.73, 128.45 (d, J = 12.8 Hz), 127.57, 94.74, 53.26. ^{31}P NMR (162 MHz, $CDCl_3$) δ 16.81. HRMS-ESI (m/z) [M+H$^+$] Calcd for $C_{21}H_{18}IN_3OP$ 486.0232; found, 486.0235.

diphenyl(1-(4-(trifluoromethyl)benzyl)-1H-1,2,3-triazol-4-yl)phosphine oxide (3n): prepared from **1** (0.2262 g, 1.0 mmol) and azide **2n** (0.2615 g, 1.3 mmol) as a white solid in 75% yield (0.3205 g).

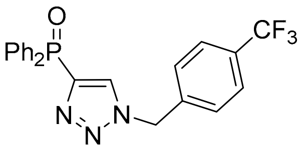

Melting point (M.P.): 141−144°C; ^{1}H NMR (400 MHz, $CDCl_3$) δ 8.19 (s, 1H), 7.90−7.85 (m, 4H), 7.64 (d, J = 7.8 Hz, 2H), 7.55−7.52 (m, 2H), 7.48−7.41 (m, 6H), 5.65 (s, 2H). ^{13}C NMR (101 MHz, $CDCl_3$) δ 142.5 (d, J = 135.0 Hz), 137.55, 132.18 (d, J = 110.7 Hz), 132.16 (d, J = 2.8 Hz), 131.46 (d, J = 10.4 Hz), 131.04 (d, J = 22.3 Hz), 128.64, 128.56, 128.43, 126.14, 123.61 (q, J = 273.2 Hz), 53.50. ^{31}P NMR (162 MHz, $CDCl_3$) δ 16.63. ^{19}F NMR (376 MHz, $CDCl_3$) δ 62.79. HRMS-ESI (m/z) [M+H$^+$] Calcd for $C_{22}H_{18}F_3N_3OP$ 428.1140; found, 428.1142.

2-((4-(diphenylphosphoryl)-1H-1,2,3-triazol-1-yl)methyl)benzonitrile (3o): prepared from **1** (0.2262 g, 1.0 mmol) and azide **2o** (0.2056 g, 1.3 mmol) as a white solid in 72% yield (0.2768 g).

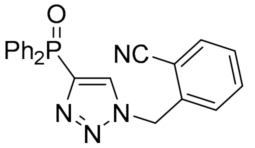

Melting point (M.P.): 165−167°C; ^{1}H NMR (400 MHz, $CDCl_3$) δ 8.27 (s, 1H), 7.90−7.85 (m, 4H), 7.73 (d, J = 7.6, 1H), 7.60 (t, J = 7.8, 1H), 7.53−7.45 (m, 7H), 7.37 (d, J = 7.8 Hz, 1H), 5.82 (s, 2H). ^{13}C NMR (101 MHz, $CDCl_3$) δ 142.66 (d, J = 135.1 Hz), 136.96, 133.69, 133.24, 132.16 (d, J = 3.0 Hz), 132.05 (d, J = 111.1 Hz), 131.42 (d, J = 10.5 Hz), 131.11 (d, J = 24.2 Hz), 129.68 (d, J = 9.1 Hz), 128.50 (d, J = 13.1 Hz), 116.63, 112.24, 51.80. ^{31}P NMR (162 MHz, $CDCl_3$) δ 16.44. HRMS-ESI (m/z) [M+H$^+$] Calcd for $C_{22}H_{18}N_4OP$ 385.1218; found, 385.1215.

3. General Procedure for Synthesis of 4-phosphinyl-1,2,3-triazoles (3p−3ah)

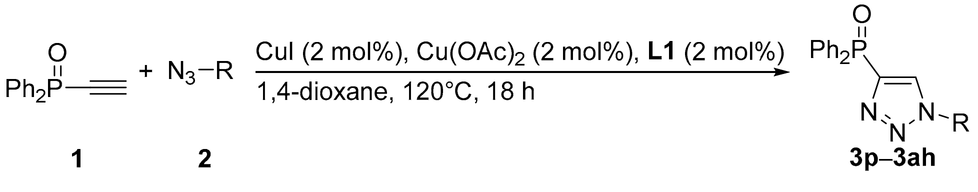

A Schlenk tube of 25 mL equipped with a magnetic stir bar was charged with **1** (0.2262 g, 1.0 mmol), azides **2** (1.3 mmol), CuI (0.0038 g, 0.02 mmol), $Cu(OAc)_2$-H_2O (0.0040 g, 0.02 mmol), **L1** (0.0059 g, 0.02 mmol) and 1,4-dioxane (5 mL) under nitrogen, and the mixture was stirred at 120°C for 18 h.

After usual workup with EtOAc and NH_4Claq, the combined organic layer was washed with brine, and dried over $MgSO_4$. After filtration, the solvents were evaporated. The crude product was subjected to column chromatography on silica gel (PE/AcOEt, 1:1) to afford 4-phosphinyl-1,2,3-triazoles (**3o**−**3ah**) in a pure form.

(1-hexyl-1H-1,2,3-triazol-4-yl)diphenylphosphine oxide (3p): prepared from **1** (0.2262 g, 1.0 mmol) and azide **2p** (0.1653 g, 1.3 mmol) as a white solid in 92% yield (0.3251 g).

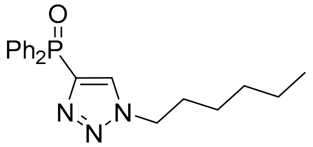

Melting point (M.P.): 76−79°C; ^{1}H NMR (400 MHz, $CDCl_3$) δ 8.18 (s, 1H), 7.88−7.83 (m, 4H), 7.51−7.47 (m, 2H), 7.45−7.40 (m, 4H), 4.38 (t, J = 7.3 Hz, 2H), 1.92−1.85 (m, 2H), 1.31−1.23 (m, 6H), 0.83 (t, J = 7.6 Hz, 2H). ^{13}C NMR (101 MHz, $CDCl_3$) δ 141.40 (d, J = 134.3 Hz), 132.27 (d, J = 111.1 Hz), 131.92, 131.26 (d, J = 12.1 Hz), 130.60 (d, J = 24.2 Hz), 128.32 (d, J = 12.1 Hz), 50.34, 30.86, 29.89, 25.88, 22.15, 13.72. ^{31}P NMR (162 MHz, $CDCl_3$) δ 16.79. HRMS-ESI (m/z) [M+H$^+$] Calcd for $C_{20}H_{25}N_3OP$ 354.1735; found, 354.1738.

(1-pentyl-1H-1,2,3-triazol-4-yl)diphenylphosphine oxide (3q): prepared from **1** (0.2262 g, 1.0 mmol) and azide **2q** (0.1471 g, 1.3 mmol) as a white solid in 95% yield (0.3224 g).

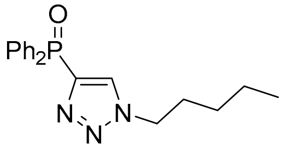

Melting point (M.P.): 93−96°C; ^{1}H NMR (400 MHz, $CDCl_3$) δ 8.18 (s, 1H), 7.86−7.80 (m, 4H), 7.45−7.43 (m, 2H), 7.41−7.37 (m, 4H), 4.34 (t, J = 7.8 Hz, 2H), 1.89−1.81 (m, 2H), 1.30−1.21 (d, J = 5.5 Hz, 4H), 0.82 (t, J = 6.9 Hz, 3H). ^{13}C NMR (101 MHz, $CDCl_3$) δ 141.25 (d, J = 137.1 Hz), 132.16 (d, J = 110.7 Hz), 131.83 (d, J = 2.7 Hz), 131.15 (d, J = 10.4 Hz), 130.58 (d, J = 24.2 Hz), 128.23 (d, J = 12.8 Hz), 50.20, 29.50, 28.17, 21.69, 13.49. ^{31}P NMR (162 MHz, $CDCl_3$) δ 16.79. HRMS-ESI (m/z) [M+H$^+$] Calcd for $C_{19}H_{23}N_3OP$ 340.1579; found, 340.1575.

(1-decyl-1H-1,2,3-triazol-4-yl)diphenylphosphine oxide (3r): prepared from **1** (0.2262 g, 1.0 mmol) and azide **2r** (0.2383 g, 1.3 mmol) as a white solid in 94% yield (0.3849 g).

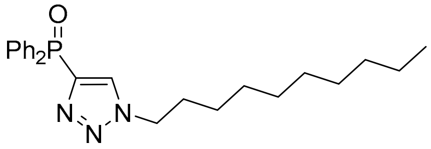

Melting point (M.P.): 93−95°C; ^{1}H NMR (400 MHz, $CDCl_3$) δ 8.18 (s, 1H), 7.85−7.79 (m, 4H), 7.45−7.36 (m, 6H), 4.33 (t, J = 7.8 Hz, 2H), 1.83 (br, 2H), 1.23−1.18 (m, 14H), 0.80 (t, J = 7.8 Hz, 3H). ^{13}C NMR (101 MHz, $CDCl_3$) δ 141.02 (d, J = 143.9 Hz), 132.1 (d, J = 110.4 Hz), 131.63, 130.99 (d, J = 10.3 Hz), 130.55 (d, J = 24.2 Hz), 128.06 (d, J = 12.8 Hz), 50.04, 31.36, 29.67,

28.96, 28.86, 28.76, 28.46, 25.95, 22.18, 13.67. ^{31}P NMR (162 MHz, $CDCl_3$) δ 16.61. HRMS-ESI (m/z) [M+H$^+$] Calcd for $C_{24}H_{33}N_3OP$ 410.2361; found, 410.2365.

(1-(3,3-dimethylbutyl)-1H-1,2,3-triazol-4-yl)diphenylphosphine oxide (3s): prepared from **1** (0.2262 g, 1.0 mmol) and azide **2s** (0.1653 g, 1.3 mmol) as a white solid in 75% yield (0.2650 g).

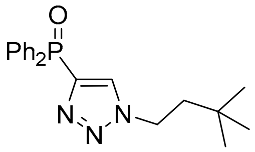

Melting point (M.P.): 126−130°C; ^{1}H NMR (400 MHz, $CDCl_3$) δ 8.20 (s, 1H), 7.86−7.81 (m, 4H), 7.49−7.38 (m, 6H), 4.38 (t, J = 7.7 Hz, 2H), 1.80 (t, J = 7.8 Hz, 2H), 0.93 (s, 9H). ^{13}C NMR (101 MHz, $CDCl_3$) δ 141.69 (d, J = 137.1 Hz), 132.41 (d, J = 110.7 Hz), 132.12 (d, J = 2.8 Hz), 131.46 (d, J = 10.5 Hz), 130.69 (d, J = 24.2 Hz), 128.51 (d, J = 12.8 Hz), 47.40, 43.80, 29.96, 29.13. ^{31}P NMR (162 MHz, $CDCl_3$) δ 16.84. HRMS-ESI (m/z) [M+H$^+$] Calcd for $C_{20}H_{25}N_3OP$ 354.1735; found, 354.1737.

(1-(heptan-3-yl)-1H-1,2,3-triazol-4-yl)diphenylphosphine oxide (3t): prepared from **1** (0.2262 g, 1.0 mmol) and azide **2t** (0.1836 g, 1.3 mmol) as a yellow liquid in 89% yield (0.3270 g).

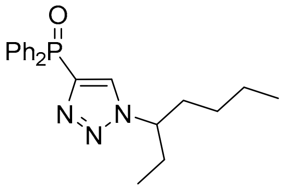

^{1}H NMR (400 MHz, $CDCl_3$) δ 8.15 (s, 1H), 7.85−7.80 (m, 4H), 7.47−7.43 (m, 2H), 7.41−7.37 (m, 4H), 4.26 (d, J = 6.8 Hz, 2H), 1.84 (s, 1H), 1.25−1.19 (s, 6H), 0.85−0.79 (m, 6H). ^{13}C NMR (101 MHz, $CDCl_3$) δ 141.10 (d, J = 137.4 Hz), 132.16 (d, J = 111.1 Hz), 131.80 (d, J = 3.0 Hz), 131.12 (d, J = 10.1 Hz), 131.10 (d, J = 23.2 Hz), 128.19 (d, J = 12.1 Hz), 53.34, 39.93, 30.02, 28.11, 23.37, 22.42, 13.61, 10.11. ^{31}P NMR (162 MHz, $CDCl_3$) δ 16.85. HRMS-ESI (m/z) [M+H$^+$] Calcd for $C_{21}H_{27}N_3OP$ 368.1892; found, 368.1896.

(1-(cyclohexylmethyl)-1H-1,2,3-triazol-4-yl)diphenylphosphine oxide (3u): prepared from **1** (0.2262 g, 1.0 mmol) and azide **2u** (0.1810 g, 1.3 mmol) as a white solid in 93% yield (0.3398 g).

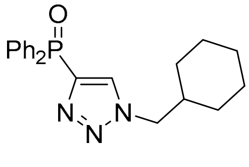

Melting point (M.P.): 136−139°C; ^{1}H NMR (400 MHz, $CDCl_3$) δ 8.13 (s, 1H), 7.83−7.79 (m, 4H), 7.41−7.36 (m, 6H), 4.16−4.14 (m, 2H), 1.59−1.49 (m, 5H), 1.18−1.07 (m, 4H), 0.92−0.87 (m, 2H). ^{13}C NMR (101 MHz, $CDCl_3$) δ 141.04 (d, J = 137.4 Hz), 132.18 (d, J = 111.1 Hz), 131.78 (d, J = 3.0 Hz), 131.06 (d, J = 24.2 Hz), 131.10 (d, J = 11.1 Hz), 128.20 (d, J = 13.1 Hz), 56.22, 38.19, 30.03, 25.62, 25.06. ^{31}P NMR (162 MHz, $CDCl_3$) δ 16.84. HRMS-ESI (m/z) [M+H$^+$] Calcd for $C_{21}H_{25}N_3OP$ 366.1735; found, 366.1737.

(1-phenethyl-1H-1,2,3-triazol-4-yl)diphenylphosphine oxide (3v): prepared from **1** (0.2262 g, 1.0 mmol) and azide **2v** (0.1913 g, 1.3 mmol) as a white solid in 85% yield (0.3174 g).

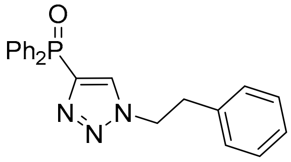

Melting point (M.P.): 160−162°C; ^{1}H NMR (400 MHz, $CDCl_3$) δ 7.90 (s, 1H), 7.85−7.80(m, 4H), 7.55−7.51 (m, 2H), 7.48−7.43 (m, 4H), 7.25−7.22 (m, 3H), 7.07−7.05 (m, 2H), 4.65 (t, J = 7.2 Hz, 2H), 3.22 (t, J = 6.6 Hz, 2H). ^{13}C NMR (101 MHz, $CDCl_3$) δ 141.40 (d, J = 136.9 Hz), 136.36, 132.20 (d, J = 111.0 Hz), 132.11 (d, J = 2.8 Hz), 131.47 (d, J = 10.5 Hz), 131.28, 131.04, 128.83, 128.47 (d, J = 13.2 Hz), 127.23, 51.73, 36.54. ^{31}P NMR (162 MHz, $CDCl_3$) δ 17.09. HRMS-ESI (m/z) [M+H^{+}] Calcd for $C_{22}H_{21}N_3OP$ 374.1422; found, 374.1426.

6-(4-(diphenylphosphoryl)-1H-1,2,3-triazol-1-yl)hexan-2-one (3w): prepared from **1** (0.2262 g, 1.0 mmol) and azide **2w** (0.1653 g, 1.3 mmol) as a yellow liquid in 90% yield (0.3306 g).

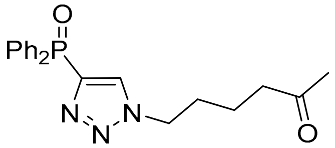

^{1}H NMR (400 MHz, $CDCl_3$) δ 8.22 (s, 1H), 7.83−7.78 (m, 4H), 7.48−7.44 (m, 2H), 7.42-7.37 (m, 4H), 4.36 (t, J = 7.2 Hz, 2H), 2.40 (t, J = 7.1 Hz, 2H), 2.04 (s, 3H), 1.89−1.82 (m, 2H), 1.56−1.48 (m, 2H). ^{13}C NMR (101 MHz, $CDCl_3$) δ 207.66, 141.31 (d, J = 137.4 Hz), 132.00 (d, J = 3.0 Hz), 132.97 (d, J = 111.1 Hz), 131.24 (d, J = 10.1 Hz), 130.80 (d, J = 24.2 Hz), 128.34 (d, J = 13.1 Hz), 50.03, 42.19, 29.72, 29.21, 20.10. ^{31}P NMR (162 MHz, $CDCl_3$) δ 17.17. HRMS-ESI (m/z) [M+H^{+}] Calcd for $C_{20}H_{23}N_3O_2P$ 368.1528; found, 368.1525.

(4-(diphenylphosphoryl)-1H-1,2,3-triazol-1-yl)methyl acetate (3x): prepared from **1** (0.2262 g, 1.0 mmol) and azide **2x** (0.1496 g, 1.3 mmol) as a yellow liquid in 76% yield (0.2594 g).

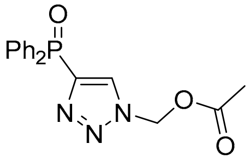

^{1}H NMR (400 MHz, $CDCl_3$) δ 8.44 (s, 1H), 7.89−7.84 (m, 4H), 7.55−7.51 (m, 2H), 7.48−7.44 (m, 4H), 6.27 (s, 2H), 2.11 (s, 3H). ^{13}C NMR (101 MHz, $CDCl_3$) δ 169.72, 142.80 (d, J = 134.3 Hz), 132.66, 132.25 (d, J = 2.9 Hz), 131.88 (d, J = 111.2 Hz), 131.43 (d, J = 10.5 Hz), 128.54 (d, J = 12.8 Hz), 69.27, 20.38. ^{31}P NMR (162 MHz, $CDCl_3$) δ 16.33. HRMS-ESI (m/z) [M+H^{+}] Calcd for $C_{17}H_{17}N_3O_3P$ 342.1008; found, 342.1011.

2-(4-(diphenylphosphoryl)-1H-1,2,3-triazol-1-yl)ethyl acetate (3y): prepared from **1** (0.2262 g, 1.0 mmol) and azide **2y** (0.1678 g, 1.3 mmol) as a white solid in 75% yield (0.2665 g).

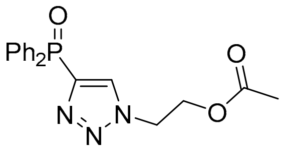

Melting point (M.P.): 120−124°C; ^{1}H NMR (400 MHz, $CDCl_3$) δ 8.31 (s, 1H), 7.84−7.79 (m, 4H), 7.50−7.45 (m, 2H), 7.43−7.38 (m, 4H), 4.63 (t, J = 4.9 Hz, 2H), 4.41 (t, J = 5.4 Hz, 2H), 1.97 (s, 3H). ^{13}C NMR (101 MHz, $CDCl_3$) δ 170.05, 141.57 (d, J = 136.7 Hz), 131.99 (d, J = 2.8 Hz), 131.93 (d, J = 100.9 Hz), 131.50 (d, J = 34.3 Hz), 131.18 (d, J = 10.5 Hz), 128.30 (d, J = 12.8 Hz), 61.50, 49.48, 20.38. ^{31}P NMR (162 MHz, $CDCl_3$) δ 17.03. HRMS-ESI (m/z) [M+H$^+$] Calcd for $C_{18}H_{19}N_3O_3P$ 356.1164; found, 356.1168.

(1-(5-chloropentyl)-1H-1,2,3-triazol-4-yl)diphenylphosphine oxide (3z): prepared from **1** (0.2262 g, 1.0 mmol) and azide **2z** (0.1919 g, 1.3 mmol) as a white solid in 76% yield (0.2734 g).

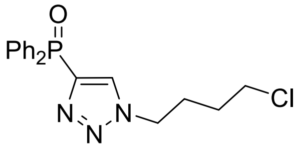

Melting point (M.P.): 104−108°C; ^{1}H NMR (400 MHz, $CDCl_3$) δ 8.21 (s, 1H), 7.89−7.84 (m, 4H), 7.53−7.43 (m, 6H), 4.46 (t, J = 7.1 Hz, 2H), 3.53 (t, J = 6.3 Hz, 2H), 2.13−2.06 (m, 2H), 1.81−1.75 (m, 2H). ^{13}C NMR (101 MHz, $CDCl_3$) δ 142.02 (d, J = 136.4 Hz), 132.26 (d, J = 111.1 Hz), 132.12 (d, J = 3.0 Hz), 131.40 (d, J = 10.1 Hz), 130.71 (d, J = 24.2 Hz), 128.48 (d, J = 13.1 Hz), 49.63, 43.75, 29.06, 27.39. ^{31}P NMR (162 MHz, $CDCl_3$) δ 16.70. HRMS-ESI (m/z) [M+H$^+$] Calcd for $C_{18}H_{20}ClN_3OP$ 360.1033; found, 360.1036.

(1-(6-chlorohexyl)-1H-1,2,3-triazol-4-yl)diphenylphosphine oxide (3aa): prepared from **1** (0.2262 g, 1.0 mmol) and azide **2aa** (0.2101 g, 1.3 mmol) as a white solid in 86% yield (0.3335 g).

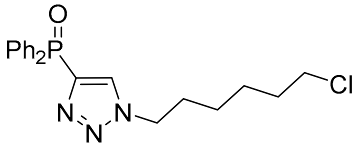

Melting point (M.P.): 115−119°C; ^{1}H NMR (400 MHz, $CDCl_3$) δ 8.18 (s, 1H), 7.83−7.78 (m, 4H), 7.44−7.38 (m, 6H), 4.34 (t, J = 6.9 Hz, 2H), 3.40 (t, J = 6.4 Hz, 2H), 1.87−1.83 (m, 2H), 1.66−1.63 (m, 2H), 1.38−1.36 (m, 2H), 1.27−1.25 (m, 2H). ^{13}C NMR (101 MHz, $CDCl_3$) δ 141.22 (d, J = 139.2 Hz), 132.04 (d, J = 110.7 Hz), 131.83 (d, J = 2.1 Hz), 131.09 (d, J = 10.4 Hz), 130.64 (d, J = 24.2 Hz), 128.21 (d, J = 12.8 Hz), 49.98, 44.43, 31.77, 29.59, 25.78, 25.31. ^{31}P NMR (162 MHz, $CDCl_3$) δ 16.77. HRMS-ESI (m/z) [M+H$^+$] Calcd for $C_{20}H_{24}ClN_3OP$ 388.1346; found, 388.1349.

(1-(6-bromohexyl)-1H-1,2,3-triazol-4-yl)diphenylphosphine oxide (3ab): prepared from **1** (0.2262 g, 1.0 mmol) and azide **2ab** (0.2679 g, 1.3 mmol) as a white solid in 74% yield (0.3199 g).

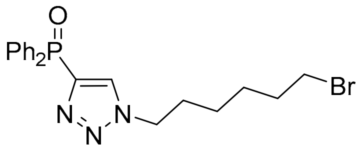

Melting point (M.P.): 114−118°C; ^{1}H NMR (400 MHz, $CDCl_3$) δ 8.24 (s, 1H), 7.85−7.80 (m, 4H), 7.47−7.40 (m, 6H), 4.36 (t, J = 7.8 Hz, 2H), 3.30 (t, J = 7.6 Hz, 2H), 1.89−1.86 (m, 2H), 1.77−1.74 (m, 2H), 1.40−1.39 (m, 2H), 1.28 (br, 2H). ^{13}C NMR (101 MHz, $CDCl_3$) δ 141.38 (d, J = 136.4 Hz), 132.10 (d, J = 110.8 Hz), 131.92 (d, J = 2.8 Hz),131.19 (d, J = 10.5 Hz), 130.66 (d, J = 23.2 Hz), 128.30 (d, J = 12.8 Hz), 50.08, 33.30, 32.00, 29.67, 27.13, 24.65. ^{31}P NMR (162 MHz, $CDCl_3$) δ 16.84. HRMS-ESI (m/z) [M+H$^+$] Calcd for $C_{20}H_{24}BrN_3OP$ 432.0840; found, 432.0845.

(1-(8-azidooctyl)-1H-1,2,3-triazol-4-yl)diphenylphosphine oxide (3ac): prepared from **1** (0.2262 g, 1.0 mmol) and azide **2ac** (0.2551 g, 1.3 mmol) as a yellow liquid in 80% yield (0.3380 g).

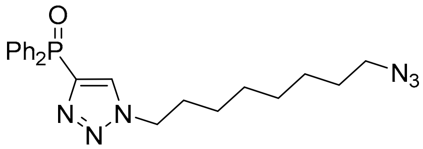

^{1}H NMR (400 MHz, $CDCl_3$) δ 8.21 (s, 1H), 7.91−7.86 (m, 4H), 7.56−7.45 (m, 6H), 4.42 (t, J = 7.0 Hz, 2H), 3.24 (t, J = 6.8 Hz, 2H), 1.95−1.91 (m, 2H), 1.59−1.54 (m, 2H), 1.3 (br, 8H). ^{13}C NMR (101 MHz, $CDCl_3$) δ 141.65 (d, J = 137.4 Hz), 132.33 (d, J = 110.7 Hz), 132.09, 131.40 (d, J = 10.3 Hz), 130.67 (d, J = 24.2 Hz), 128.47 (d, J = 12.7 Hz), 51.28, 50.44, 30.00, 28.75, 28.69, 28.65, 26.45, 26.25. ^{31}P NMR (162 MHz, $CDCl_3$) δ 16.97. HRMS-ESI (m/z) [M+H$^+$] Calcd for $C_{22}H_{28}N_6OP$ 423.2062; found, 423.2066.

diphenyl(1-(p-tolyl)-1H-1,2,3-triazol-4-yl)phosphine oxide (3ad): prepared from **1** (0.2262 g, 1.0 mmol) and azide **2ad** (0.1731 g, 1.3 mmol) as a white solid in 81% yield (0.2911 g).

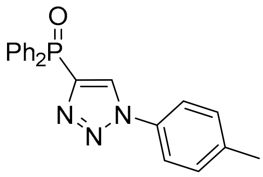

Melting point (M.P.): 222−226°C; ^{1}H NMR (400 MHz, $CDCl_3$) δ 8.61 (s, 1H), 7.97−7.92 (m, 4H), 7.62 (d, J = 8.3 Hz, 2H), 7.56−7.47 (m, 6H), 7.33 (d, J = 8.1 Hz, 2H), 2.42 (s, 3H). ^{13}C NMR (101 MHz, $CDCl_3$) δ 142.49 (d, J = 135.3 Hz), 139.59, 134.07, 132.70, 132.26 (d, J = 3.0 Hz), 131.54 (d, J = 11.1 Hz), 130.43, 128.98 (d, J = 23.2 Hz), 128.60 (d, J = 13.1 Hz), 120.60, 21.13. ^{31}P NMR (162 MHz, $CDCl_3$) δ 16.89. HRMS-ESI (m/z) [M+H$^+$] Calcd for $C_{21}H_{19}N_3OP$ 360.1266; found, 360.1268.

diphenyl(1-(m-tolyl)-1H-1,2,3-triazol-4-yl)phosphine oxide (3ae): prepared from **1** (0.2262 g, 1.0 mmol) and azide **2ae** (0.1731 g, 1.3 mmol) as a white solid in 78% yield (0.2803 g).

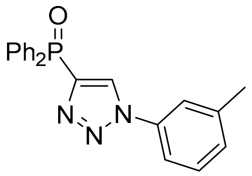

Melting point (M.P.): 219−220°C; ^{1}H NMR (400 MHz, $CDCl_3$) δ 8.63 (s, 1H), 7.96−7.91 (m, 4H), 7.58−7.47 (m, 8H), 7.41 (t, J = 8.0 Hz, 1H), 7.29−7.27 (m, 1H), 2.45 (s, 3H). ^{13}C NMR (101 MHz, $CDCl_3$) δ 142.57 (d, J = 135.3 Hz), 140.17, 136.27, 132.20 (d, J = 3.0 Hz), 132.14 (d, J = 111.1 Hz), 131.49 (d, J = 10.1 Hz), 130.06, 129.65, 129.03 (d, J = 24.2 Hz), 128.54 (d, J = 13.1 Hz), 121.38, 117.76, 21.37. ^{31}P NMR (162 MHz, $CDCl_3$) δ 16.69. HRMS-ESI (m/z) [M+H^{+}] Calcd for $C_{21}H_{19}N_3OP$ 360.1266; found, 360.1265.

diphenyl(1-(o-tolyl)-1H-1,2,3-triazol-4-yl)phosphine oxide (3af): prepared from **1** (0.2262 g, 1.0 mmol) and azide **2af** (0.1731 g, 1.3 mmol) as a white solid in 77% yield (0.2767 g).

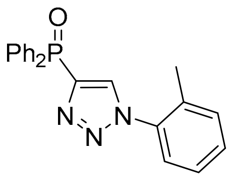

Melting point (M.P.): 153−156°C; ^{1}H NMR (400 MHz, $CDCl_3$) δ 8.38 (s, 1H), 7.98−7.93 (m, 4H), 7.57−7.48 (m, 6H), 7.45−7.42 (m, 1H), 7.39 (d, J = 8.0 Hz, 1H), 7.35−7.34 (m, 2H), 2.23 (s, 3H). ^{13}C NMR (101 MHz, $CDCl_3$) δ 141.82 (d, J = 135.3 Hz),135.68, 133.50, 132.27 (d, J = 111.1 Hz), 132.22 (d, J = 23.2 Hz), 132.18 (d, J = 3.0 Hz), 131.59, 131.46 (d, J = 11.1 Hz), 130.23, 128.56 (d, J = 13.1 Hz), 126.97, 125.82, 17.92. ^{31}P NMR (162 MHz, $CDCl_3$) δ 16.50. HRMS-ESI (m/z) [M+H^{+}] Calcd for $C_{21}H_{19}N_3OP$ 360.1266; found, 360.1269.

(1-(3-iodophenyl)-1H-1,2,3-triazol-4-yl)diphenylphosphine oxide (3ag): prepared from **1** (0.2262 g, 1.0 mmol) and azide **2ag** (0.3185 g, 1.3 mmol) as a white solid in 58% yield (0.2733 g).

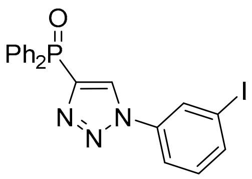

Melting point (M.P.): 176−180°C; ^{1}H NMR (400 MHz, $CDCl_3$) δ 8.67 (s, 1H), 7.96−7.91 (m, 4H), 7.58−7.48 (m, 9H), 7.13 (d, J = 7.1 Hz, 1H). ^{13}C NMR (101 MHz, $CDCl_3$) δ 143.00 (d, J = 134.3 Hz), 142.08, 137.44, 132.46, 132.26 (d, J = 3.0 Hz), 131.42 (d, J = 11.1 Hz), 131.14, 129.02 (d, J = 23.2 Hz), 128.54 (d, J = 12.1 Hz), 119.52, 116.52, 111.44. ^{31}P NMR (162 MHz, $CDCl_3$) δ 16.59. HRMS-ESI (m/z) [M+H^{+}] Calcd for $C_{20}H_{16}IN_3OP$ 472.0076; found, 472.0078.

(1-(4-iodophenyl)-1H-1,2,3-triazol-4-yl)diphenylphosphine oxide (3ah): prepared from **1** (0.2262 g, 1.0 mmol) and azide **2ah** (0.3185 g, 1.3 mmol) as a white solid in 62% yield (0.2922 g).

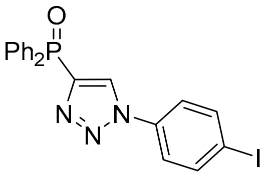

Melting point (M.P.): 186−190°C; ^{1}H NMR (400 MHz, $CDCl_3$) δ 8.65 (s, 1H), 7.96−7.91 (m, 4H), 7.75 (d, J = 8.2 Hz, 2H), 7.57−7.54 (m, 2H), 7.51−7.47 (m, 4H), 7.18 (d, J = 8.0 Hz, 2H). ^{13}C NMR (101 MHz, $CDCl_3$) δ 142.88 (d, J = 134.3 Hz), 141.23, 133.04, 132.26 (d, J = 3.0 Hz), 132.01 (d, J = 111.1 Hz), 131.46 (d, J = 11.1 Hz), 128.88 (d, J = 24.2 Hz), 128.57 (d, J = 12.1 Hz), 122.16, 120.27. ^{31}P NMR (162 MHz, $CDCl_3$) δ 16.65. HRMS-ESI (m/z) [M+H$^+$] Calcd for $C_{20}H_{16}IN_3OP$ 472.0076; found, 472.0079.

4. General Procedure for Synthesis of 4-phosphanyl-1,2,3-triazoles (4a−4e)

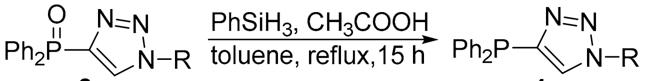

A Schlenk tube of 25 mL equipped with a magnetic stir bar was charged with **3** (0.5 mmol), $PhSiH_3$ (0.2164 g, 2.0 mmol), CH_3COOH (0.3003 g, 5.0 mmol), and toluene (5 mL) under nitrogen, and the mixture was stirred under refluxing for 15 h. After usual workup with EtOAc and NH_4Claq, the combined organic layer was washed with brine, and dried over $MgSO_4$. After filtration, the solvents were evaporated. The crude product was subjected to column chromatography on silica gel (PE/AcOEt, 6:1) to afford 4-phosphanyl-1,2,3-triazoles (**4a**−**4e**) in a pure form.

1-benzyl-4-(diphenylphosphanyl)-1H-1,2,3-triazole (4a): prepared from **3a** (0.1797 g, 0.5 mmol) as a white solid in 85% yield (0.1459 g).

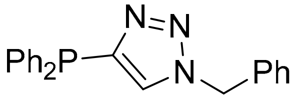

Melting point (M.P.): 78−88°C; ^{1}H NMR (400 MHz, $CDCl_3$) δ 7.46−7.41 (m, 4H), 7.36−7.35 (m, 4H), 7.32−7.31 (m, 6H), 7.24−7.22 (m, 2H), 5.52 (s, 2H). ^{13}C NMR (101 MHz, $CDCl_3$) δ 144.82 (d, J = 6.1 Hz), 136.26 (d, J = 7.1 Hz), 134.47, 133.40 (d, J = 20.2 Hz), 131.43 (d, J = 10.1 Hz), 129.27 (d, J = 26.3 Hz), 128.97 (d, J = 18.2 Hz), 128.69, 128.45 (d, J = 8.1 Hz), 127.91, 53.91. ^{31}P NMR (162 MHz, $CDCl_3$) δ -31.86. HRMS-ESI (m/z) [M+H$^+$] Calcd for $C_{21}H_{19}N_3P$ 344.1317; found, 344.1319.

4-(diphenylphosphanyl)-1-(4-methylbenzyl)-1H-1,2,3-triazole (4b): prepared from **3b** (0.1867 g, 0.5 mmol) as a white solid in 83% yield (0.1483 g).

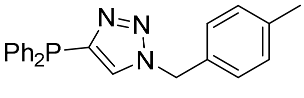

Melting point (M.P.): 66−70°C; ^{1}H NMR (400 MHz, $CDCl_3$) δ 7.46−7.40 (m, 4H), 7.34−7.29

(m, 7H), 7.17−7.10 (m, 4H), 5.46 (s, 2H), 2.33 (s, 3H). ^{13}C NMR (101 MHz, $CDCl_3$) δ 144.65 (d, J = 6.1 Hz), 138.60, 136.35 (d, J = 6.9 Hz), 133.39 (d, J = 20.1 Hz), 131.45, 129.70, 129.17 (d, J = 26.3 Hz), 128.83, 128.42 (d, J = 7.3 Hz), 127.95, 53.71, 21.09. ^{31}P NMR (162 MHz, $CDCl_3$) δ -31.86. HRMS-ESI (m/z) [M+H$^+$] Calcd for $C_{22}H_{21}N_3P$ 358.1473; found, 358.1476.

1-(3-chlorobenzyl)-4-(diphenylphosphanyl)-1H-1,2,3-triazole (4c): prepared from **3h** (0.1969 g, 0.5 mmol) as a white solid in 82% yield (0.1549 g).

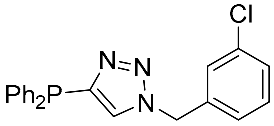

Melting point (M.P.): 60− 68°C; ^{1}H NMR (400 MHz, $CDCl_3$) δ 7.46−7.41 (m, 4H), 7.36 (s, 1H), 7.32−7.28 (m, 7H), 7.26−7.25 (m, 1H), 7.20 (s, 1H), 7.10 (d, J = 7.1 Hz, 1H), 5.48 (s, 2H). ^{13}C NMR (101 MHz, $CDCl_3$) δ 145.28 (d, J = 6.1 Hz), 136.46, 136.14 (d, J = 7.0 Hz), 134.94, 133.39 (d, J = 20.1 Hz), 130.35, 129.14 (d, J = 25.2 Hz), 128.92 (d, J = 4.3 Hz), 128.49 (d, J = 7.3 Hz), 127.92, 125.91, 53.16. ^{31}P NMR (162 MHz, $CDCl_3$) δ -31.90. HRMS-ESI (m/z) [M+H$^+$] Calcd for $C_{21}H_{18}ClN_3P$ 378.0927; found, 378.0923.

1-(4-(diphenylphosphanyl)-1H-1,2,3-triazol-1-yl)hexan-1-one (4d): prepared from **3p** (0.1767 g, 0.5 mmol) as a yellow liquid in 80% yield (0.1406 g).

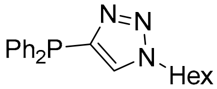

^{1}H NMR (400 MHz, $CDCl_3$) δ 7.47−7.42 (m, 4H), 7.34−7.31 (m, 7H), 4.32 (t, J = 7.3 Hz, 2H), 1.90−1.83 (m, 2H), 1.31−1.26 (m, 6H), 0.86 (t, J = 7.8 Hz, 3H). ^{13}C NMR (101 MHz, $CDCl_3$) δ 144.33 (d, J = 4.0 Hz), 136.46 (d, J = 7.1 Hz), 133.36 (d, J = 20.2 Hz), 129.02 (d, J = 23.2 Hz), 128.85, 128.46 (d, J = 7.1 Hz), 50.20, 31.03, 30.17, 26.08, 22.35, 13.86. ^{31}P NMR (162 MHz, $CDCl_3$) δ -31.91. HRMS-ESI (m/z) [M+H$^+$] Calcd for $C_{20}H_{25}N_3OP$ 354.1735; found, 354.1738.

1-(cyclohexylmethyl)-4-(diphenylphosphanyl)-1H-1,2,3-triazole (4e): prepared from **3u** (0.1822 g, 0.5 mmol) as a white solid in 78% yield (0.1363 g).

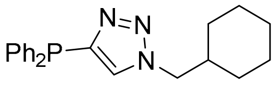

Melting point (M.P.): 129−136°C; ^{1}H NMR (400 MHz, $CDCl_3$) δ 7.47−7.42 (m, 4H), 7.34−7.32 (m, 7H), 4.16 (d, J = 7.2 Hz, 2H), 1.90−1.82 (m, 1H), 1.74−1.66 (m, 4H), 1.59 (d, J = 7.8 Hz, 2H), 1.20−1.16 (m, 2H), 1.01−0.91 (m, 2H). ^{13}C NMR (101 MHz, $CDCl_3$) δ 144.10 (d, J = 5.0 Hz), 136.52 (d, J = 7.1 Hz), 133.38 (d, J = 20.0 Hz), 129.67 (d, J = 24.2 Hz), 128.84, 128.46 (d, J = 7.3 Hz), 56.32, 38.60, 30.44, 26.02, 25.42. ^{31}P NMR (162 MHz, $CDCl_3$) δ -31.99. HRMS-ESI (m/z) [M+H$^+$] Calcd for $C_{21}H_{25}N_3P$ 350.1786; found, 350.1788.

5. General Procedure for Synthesis of 1-benzyl-substituted 1,2,3-triazoles (5a−5c)

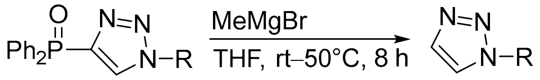

A Schlenk tube of 25 mL equipped with a magnetic stir bar was charged with **3** (1.0 mmol), MeMgBr (1.5 mmol) and THF (5 mL) under nitrogen, and the mixture was stirred at 50°C for 8 h. After usual workup with EtOAc and NH_4Claq, the combined organic layer was washed with brine, and dried over $MgSO_4$. After filtration, the solvents were evaporated. The crude product was subjected to column chromatography on silica gel (PE/AcOEt, 4:1) to afford 1-benzyl-substituted 1,2,3-triazoles (**5a−5c**) in a pure form.

1-benzyl-1H-1,2,3-triazole (5a)[2]: prepared from **3a** (0.3594 g, 1.0 mmol) as a white solid in 95% yield (0.1512 g).

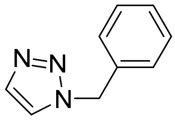

^{1}H NMR (400 MHz, $CDCl_3$) δ 7.64 (s, 1H), 7.41 (s, 1H), 7.33−7.29 (m, 3H), 7.20−7.18 (m, 2H), 5.50 (s, 2H). ^{13}C NMR (101 MHz, $CDCl_3$) δ 134.64, 134.19, 129.06, 128.68, 127.96, 123.28, 53.92.

1-(4-chlorobenzyl)-1H-1,2,3-triazole (5b)[3]: prepared from **3i** (0.3938 g, 1.0 mmol) as a white solid in 92% yield (0.1781 g).

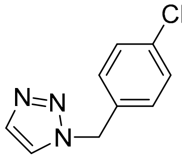

^{1}H NMR (400 MHz, $CDCl_3$) δ 7.73 (s, 1H), 7.48 (s, 1H), 7.35 (d, J = 8.4 Hz, 2H), 7.20 (d, J = 8.3 Hz, 2H), 5.54 (s, 2H). ^{13}C NMR (101 MHz, $CDCl_3$) δ 134.78, 134.37, 133.16, 129.30, 129.29, 123.25, 53.20.

1-(4-bromobenzyl)-1H-1,2,3-triazole (5c)[4]: prepared from **3j** (0.4383 g, 1.0 mmol) as a white solid in 90% yield (0.2143 g).

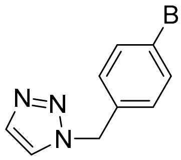

^{1}H NMR (400 MHz, $CDCl_3$) δ 7.72 (s, 1H), 7.50 (d, J = 8.6 Hz, 3H), 7.14 (d, J = 8.3 Hz, 2H), 5.53 (s, 2H). ^{13}C NMR (101 MHz, $CDCl_3$) δ 134.36, 133.66, 132.24, 129.55, 123.28, 122.85, 53.23.

6. X-Ray Crystallographic Data

X-Ray crystallographic data for compound **6k** was recorded at 296 K on a Rigaku Oxford Diffraction Supernova Dual Source, Cu at zero equipped with an AtlasS2 CCD using Cu Kα radiation. Data reduction was carried out with the diffractometer software. The structures were solved by direct methods using Olex2 software[5] and the non-hydrogen atoms were located from the trial structure and then refined anisotropically with SHELXL-2014[6] using a full-matrix least squares procedure based on F2. The weighted R factor, wR and goodness-of-fit S values were obtained based on F2.The hydrogen atom positions were fixed geometrically at the calculated distances and allowed to ride on their parent atoms. Crystallographic data for the structure reported in this paper have been deposited at the Cambridge Crystallographic Data Center and allocated with the deposition number: 2564149.

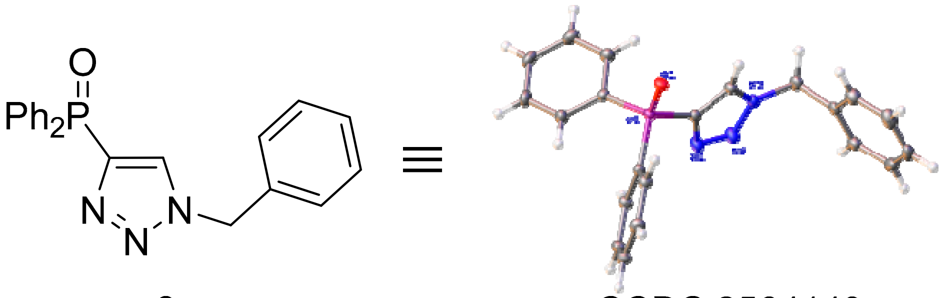

3a CCDC 2564149

Figure S1. ORTEP drawing of **3a.**

Table S1. Crystal data and structure refinement for 3a.

Identification code	37671_P
Empirical formula	$C_{21}H_{18}N_3OP$
Formula weight	359.35
Temperature/K	150.00(10)
Crystal system	triclinic
Space group	P-1
a/Å	8.5253(2)
b/Å	9.5366(3)
c/Å	11.4393(3)
α/°	87.058(2)
β/°	85.528(2)
γ/°	79.857(2)
Volume/Å^3	912.05(4)
Z	2
ρ_{calc}g/cm^3	1.309
μ/mm^{-1}	1.448
F(000)	376.0
Crystal size/mm^3	0.16 × 0.14 × 0.12
Radiation	Cu Kα (λ = 1.54184)
2Θ range for data collection/°	7.758 to 148.92
Index ranges	$-7 \leq h \leq 10$, $-11 \leq k \leq 11$, $-14 \leq l \leq 14$
Reflections collected	9044
Independent reflections	3530 [R_{int} = 0.0221, R_{sigma} = 0.0188]

Data/restraints/parameters	3530/0/235
Goodness-of-fit on F^2	1.054
Final R indexes [I>=2σ (I)]	$R_1 = 0.0399$, $wR_2 = 0.1087$
Final R indexes [all data]	$R_1 = 0.0408$, $wR_2 = 0.1097$
Largest diff. peak/hole / e Å^{-3}	0.48/-0.44

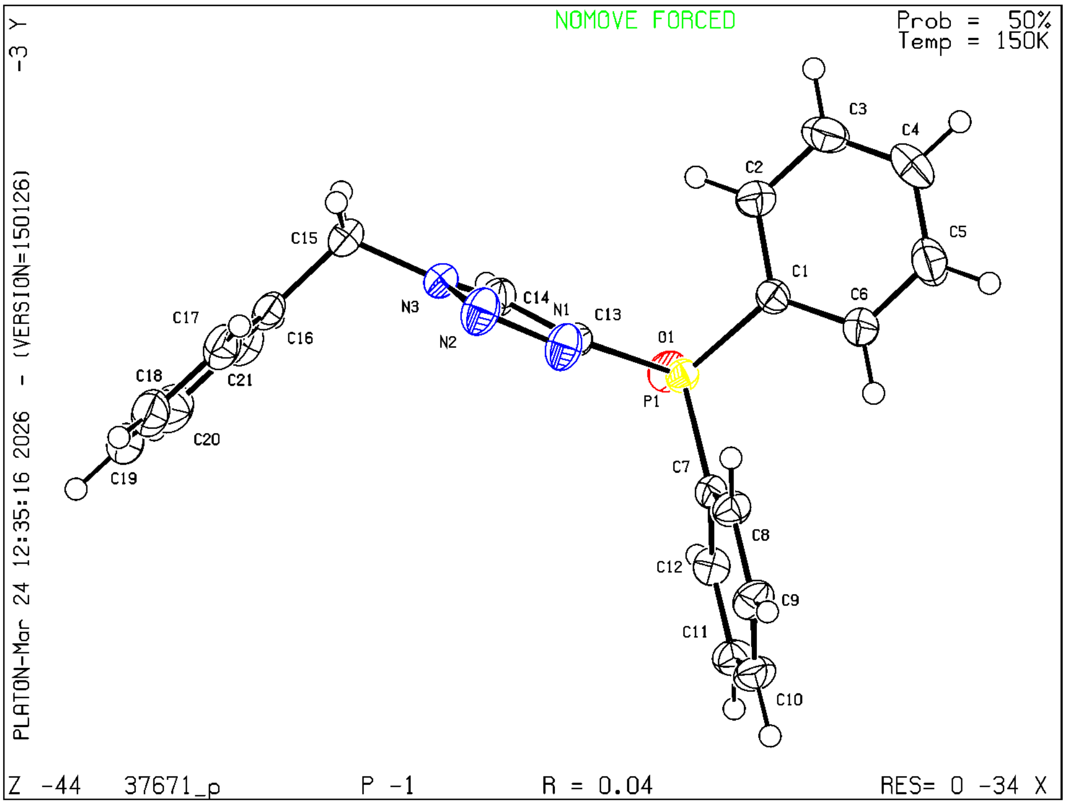

The ellipsoid contour % probability level in the caption for the image of the structure is 50%.

General procedure for preparation of single crystal of compound 3a

3a (30 mg) was dissolved in 5.0 mL of EtOAc in 10 mL of test tube, and then 2 mL of *n*-hexane was added into the test tube. Subsequently, the tube was sealed with sealing film and placed in rt to give **3a** as single crystals in 6 days.

7. References

1. Peng, L.; Zhao, Y.; Okuda, Y.; Le, L.; Tang, Z.; Yin, S.; Qiu, R.; Orita, A. "Process-Divergent Syntheses of 4- and 5-Sulfur-Functionalized 1,2,3-Triazoles via Copper-Catalyzed Azide–Alkyne Cycloadditions of 1-Phosphinyl-2-sulfanylethynes," *J. Org. Chem.* **2023**, 88, 3089–3108.
2. Patel, A.; Naumann, R.A.; Frank, L.G.; Williams, V.E.; Romero, N.A. Alkenylthianthrenium Salts as Synthetic Equivalents of (Cyclo)alkynes in Azide Cycloaddition Reactions. *Angew. Chem. Int. Ed.* **2026**, *65*, e20308.

3. Voronin, V.V.; Ledovskaya, M.S.; Rodygin, K.S.; Ananikov, V.P. Cycloaddition Reactions of in situ Generated C_2D_2 in Dioxane: Efficient Synthetic Approach to D_2-Labeled Nitrogen Heterocycles. *Eur. J. Org. Chem*. **2021**, *41*, 5640–5648.
4. Fu, R.; Lei, Y.; Wang, Y.; Huang, B.; Qiu, D.; Wang, J. Synthesis of Substituted 1-Benzyl-2*H*-1,2,3-Triazoles by a Click Reaction Using Calcium Carbide as an Acetylene Source. *Synlett* **2025**, *36*, 874–878.
5. Dolomanov, O.V.; Bourhis, L.J.; Gildea, R.J.; Howard, J.A.K.; Puschmann, H. *OLEX2*: A Complete Structure Solution, Refinement and Analysis Pprogram. *J. Appl. Cryst.* **2009**, *42*, 339–341.
6. Kratzert, D.; Krossing, I.; Holstein, J.J. DSR: Enhanced Modelling and Refinement of Disordered Structures with SHELXL. *J. Appl. Cryst.* **2015**, *48*, 933–938.

8. NMR Copies of All Compounds

^{1}H-NMR (400 MHz, $CDCl_3$) spectrum of 3a

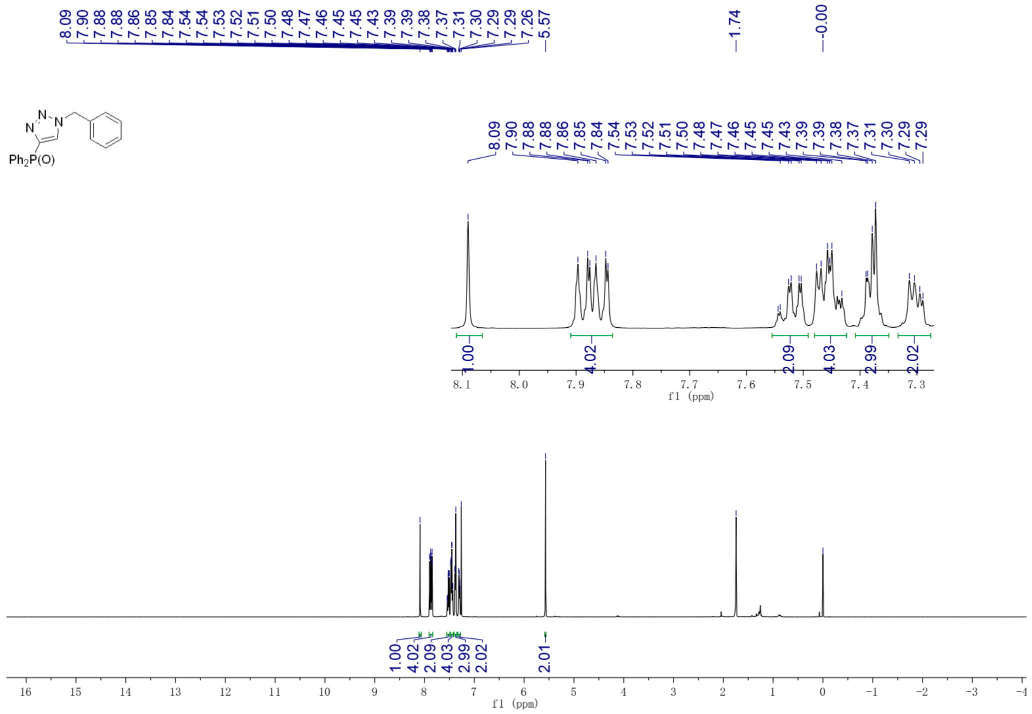

^{13}C-NMR (101 MHz, $CDCl_3$) spectrum of **3a**

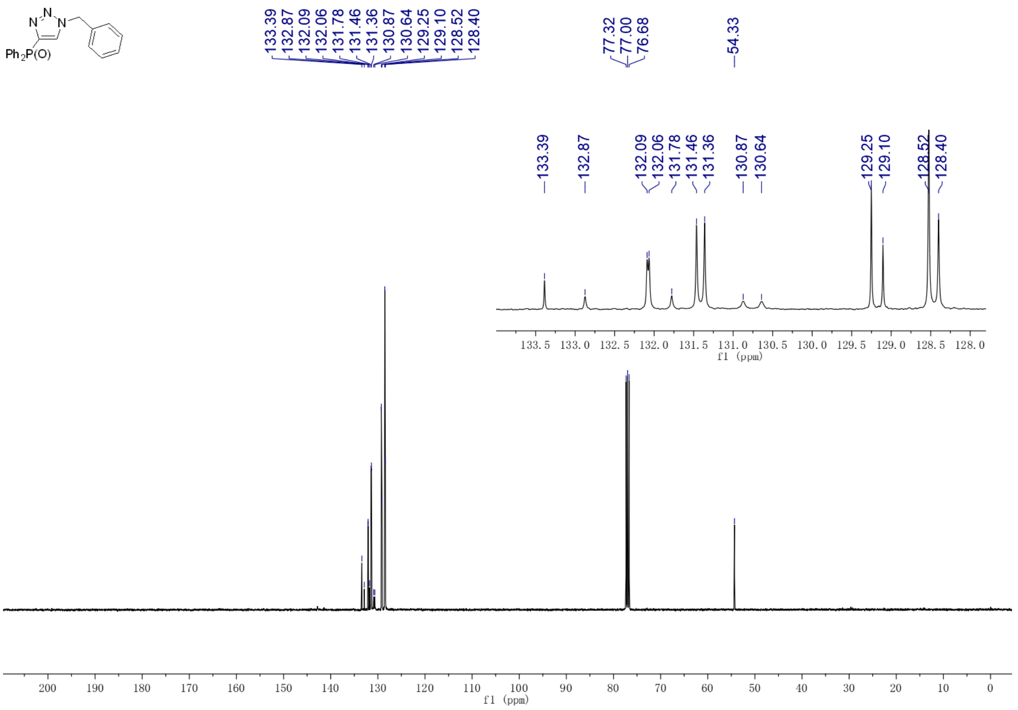

^{31}P NMR (162 MHz, Chloroform-*d*) spectrum of **3a**

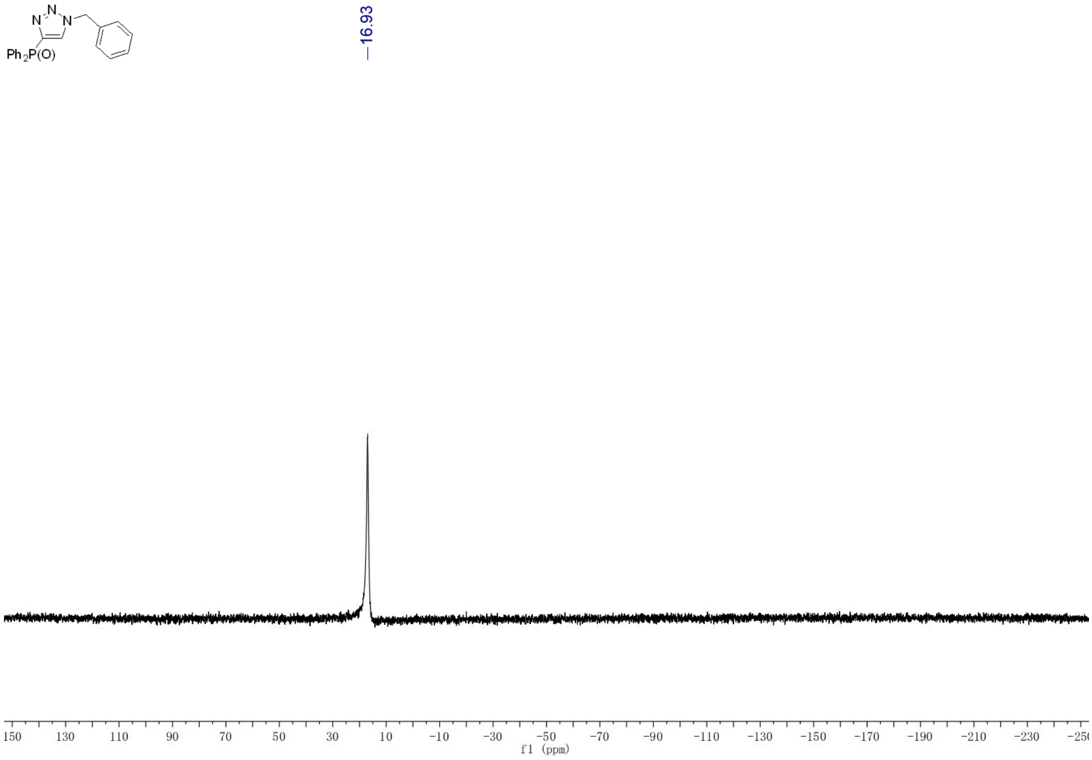

^{1}H-NMR (400 MHz, $CDCl_3$) spectrum of **3b**

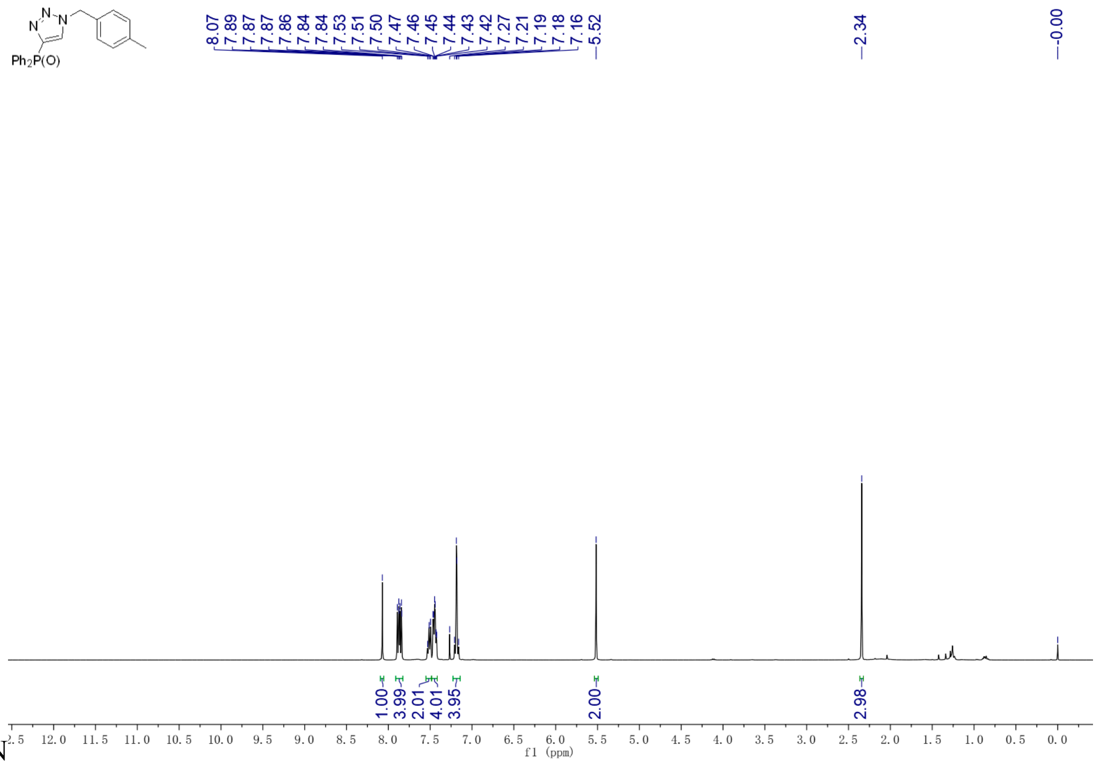

N

^{13}C-NMR (101 MHz, $CDCl_3$) spectrum of **3b**

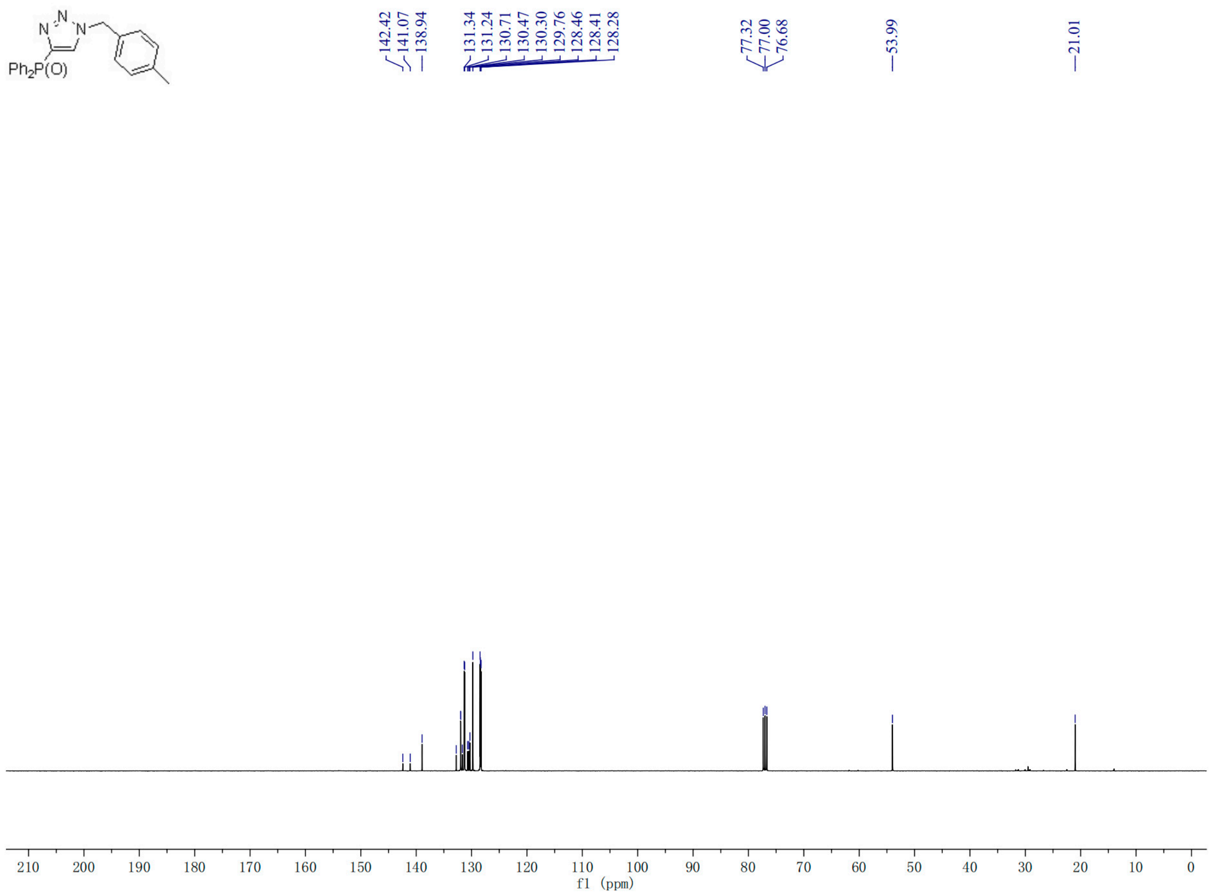

^{31}P NMR (162 MHz, Chloroform-*d*) spectrum of **3b**

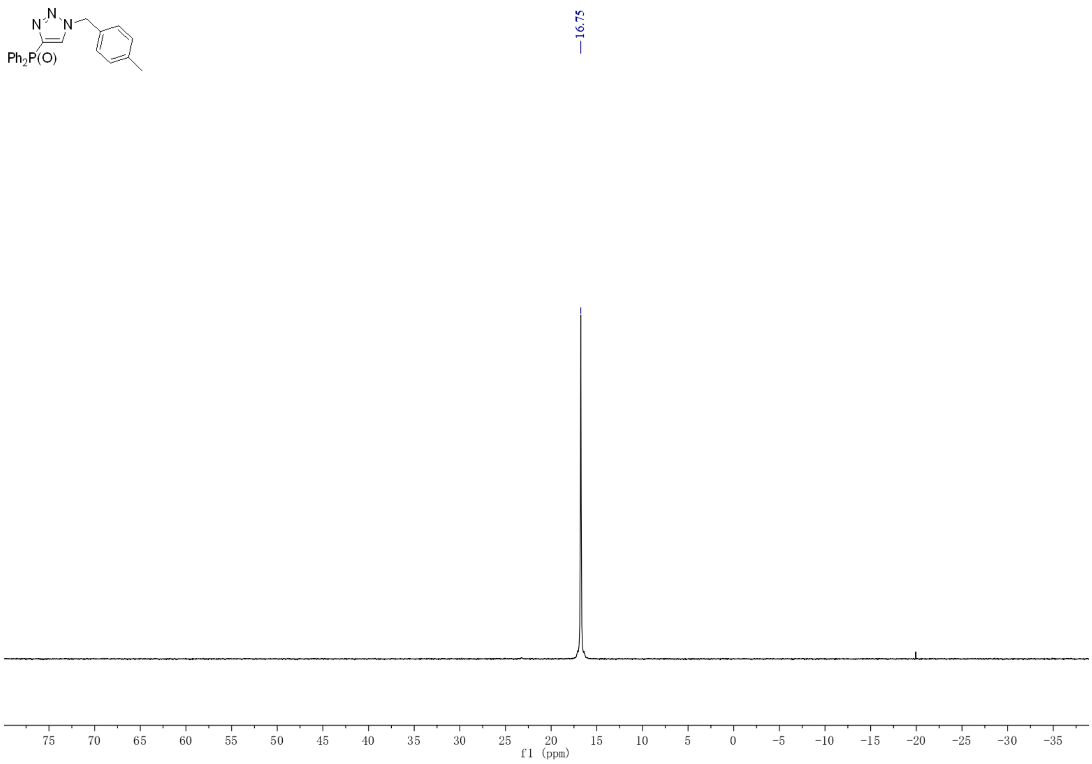

^{1}H-NMR (400 MHz, $CDCl_3$) spectrum of **3c**

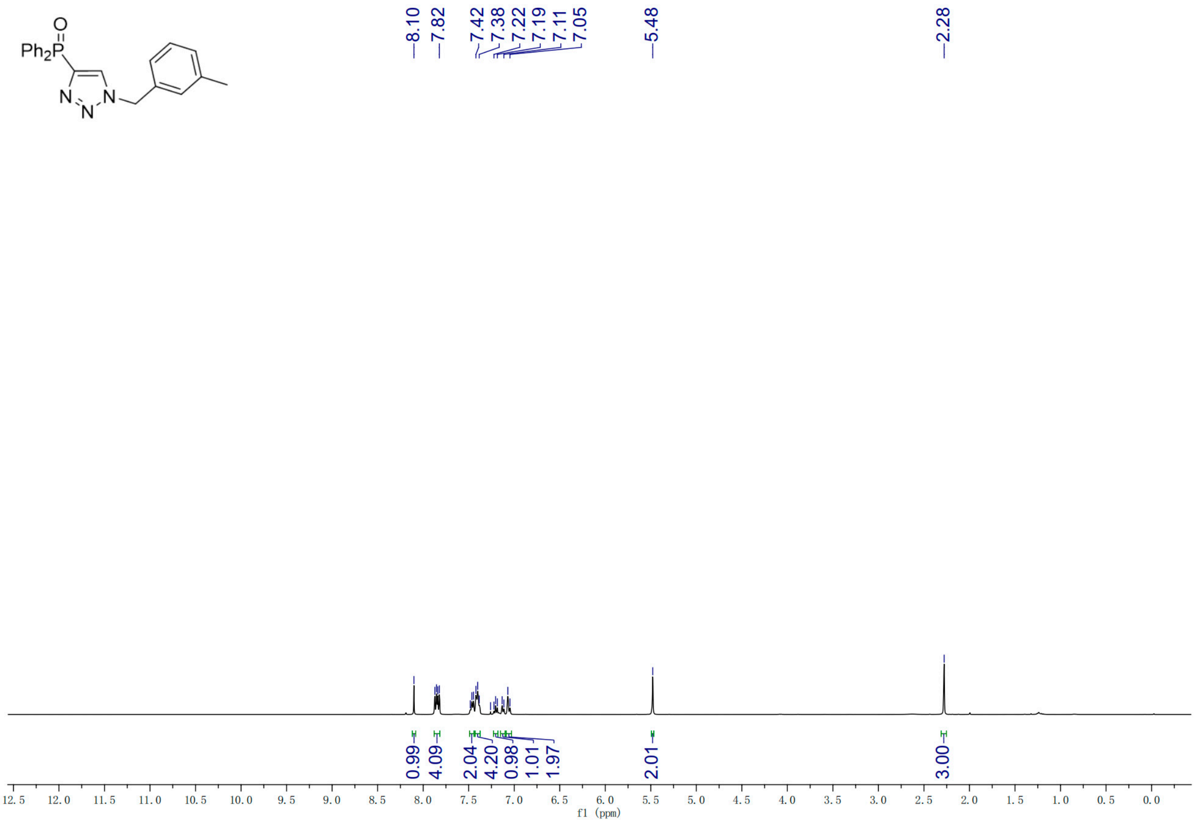

^{13}C-NMR (101 MHz, $CDCl_3$) spectrum of **3c**

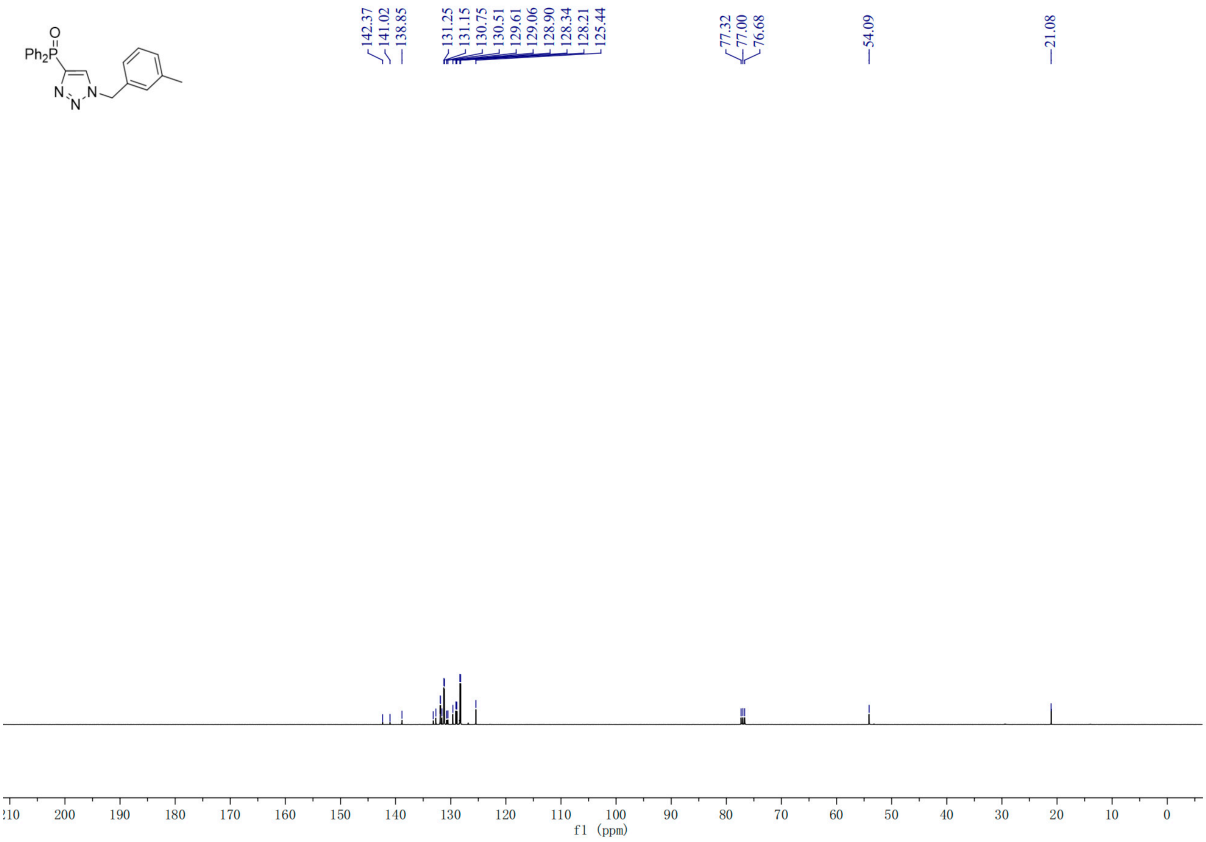

^{31}P NMR (162 MHz, Chloroform-*d*) spectrum of **3c**

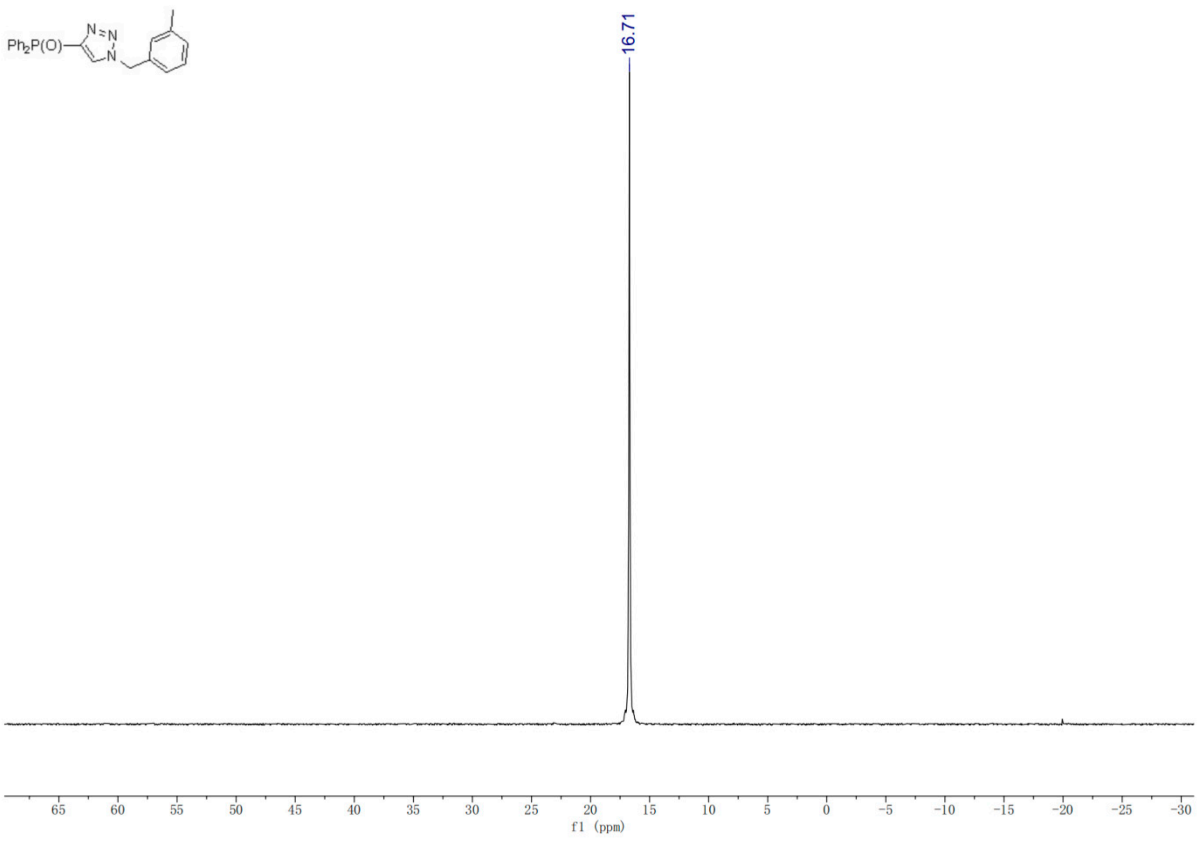

^{1}H-NMR (400 MHz, $CDCl_3$) spectrum of **3d**

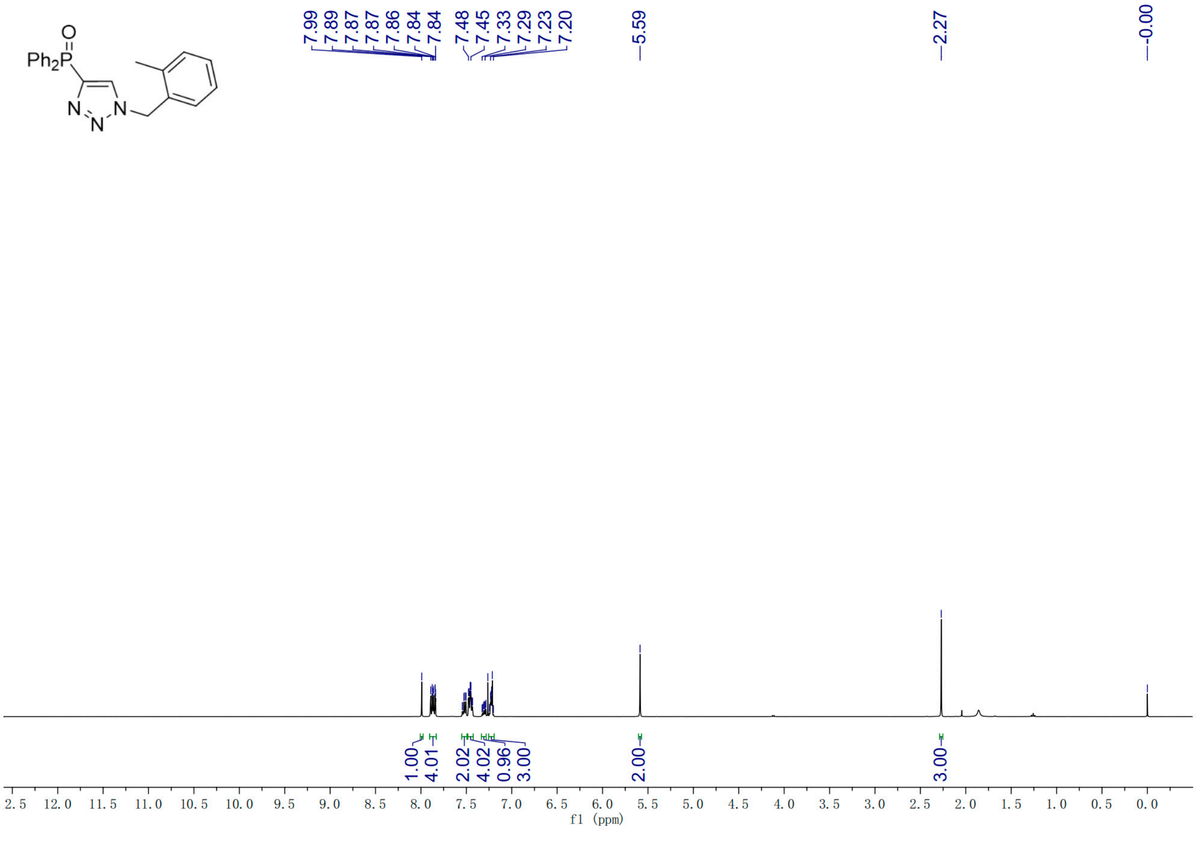

^{13}C-NMR (101 MHz, $CDCl_3$) spectrum of **3d**

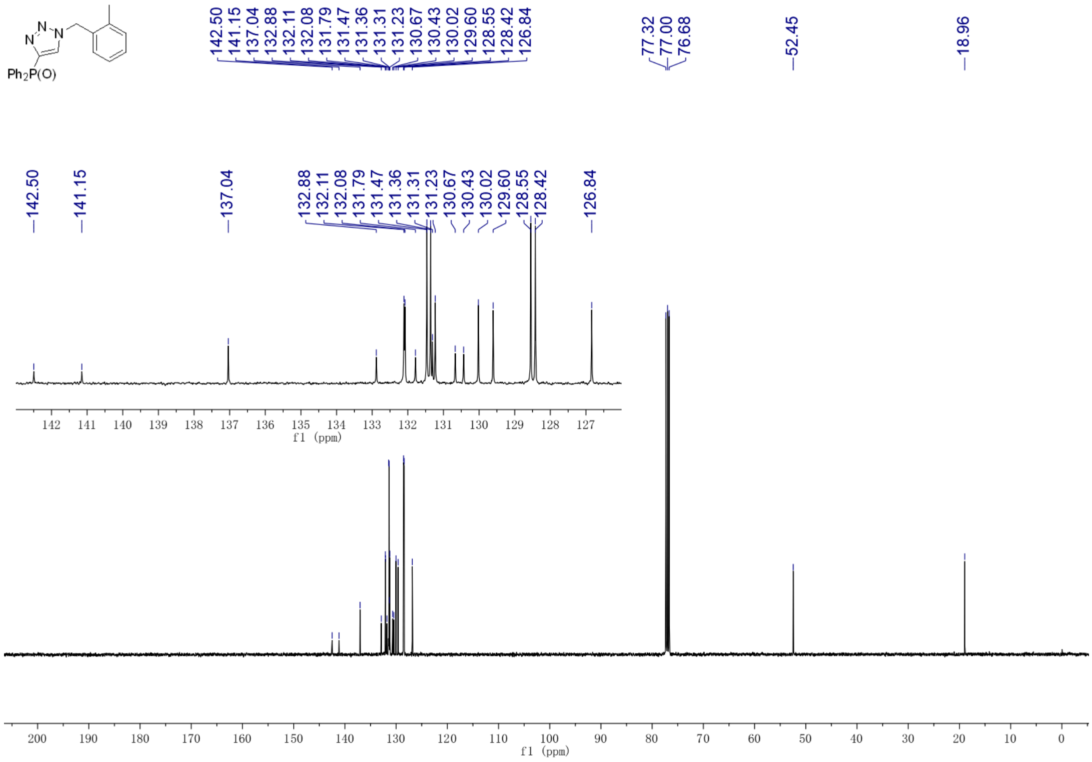

^{31}P NMR (162 MHz, Chloroform-*d*) spectrum of **3d**

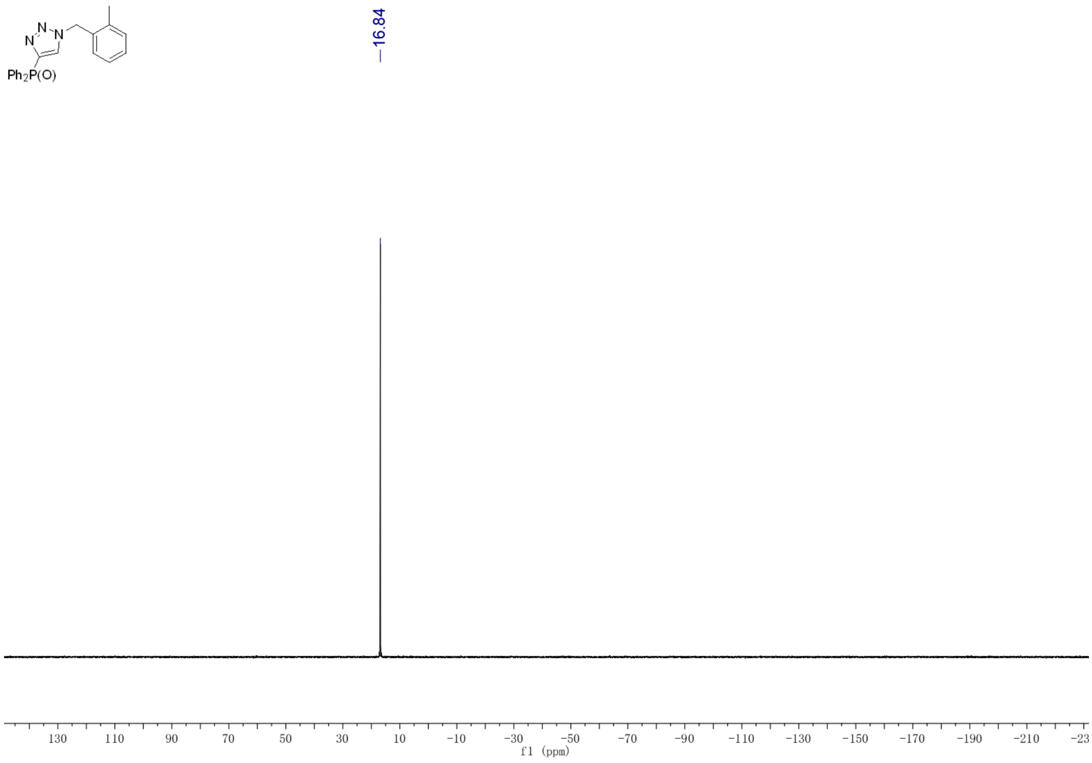

^{1}H-NMR (400 MHz, $CDCl_3$) spectrum of **3e**

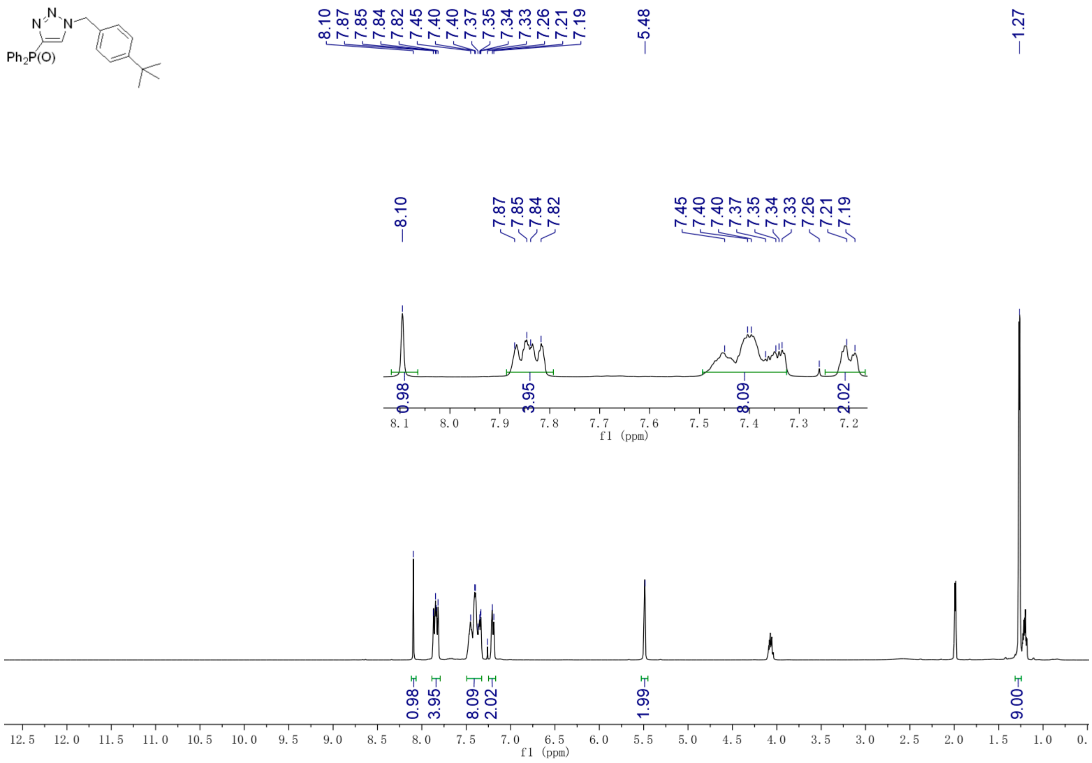

^{13}C-NMR (101 MHz, $CDCl_3$) spectrum of **3e**

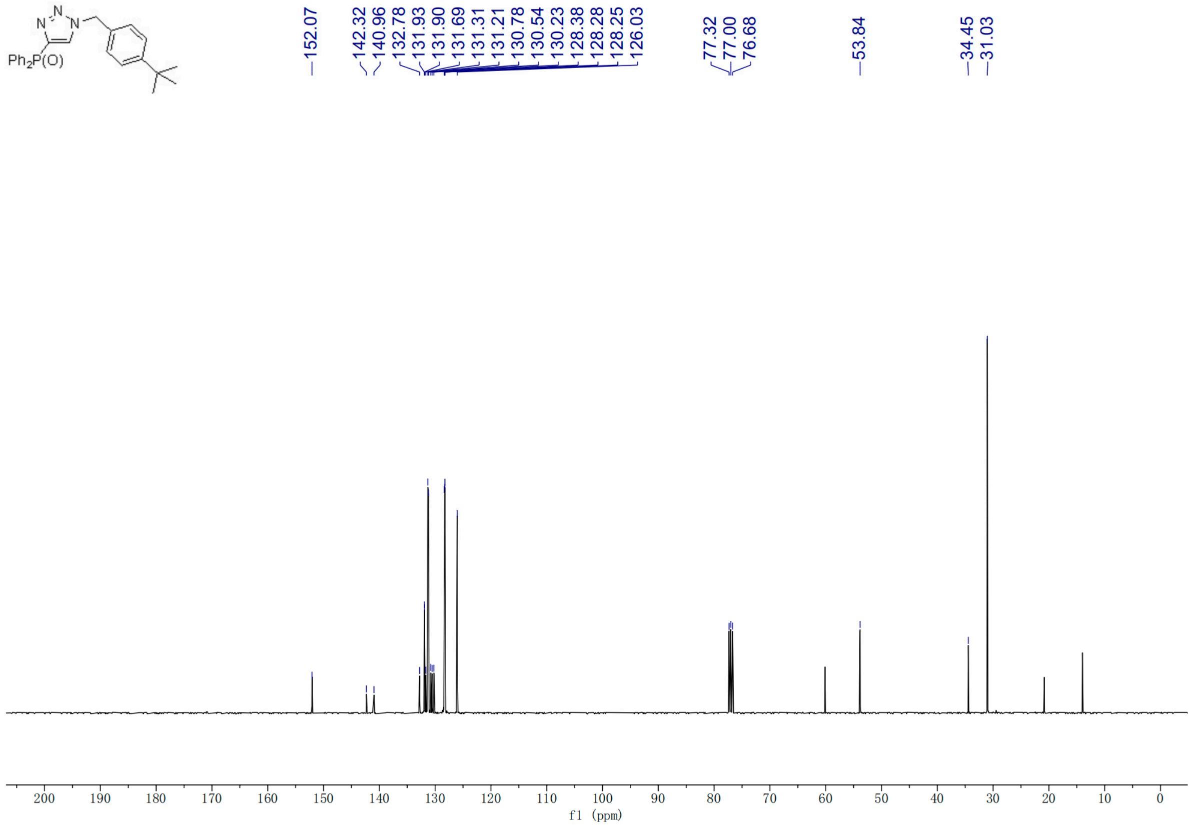

^{31}P NMR (162 MHz, Chloroform-*d*) spectrum of **3e**

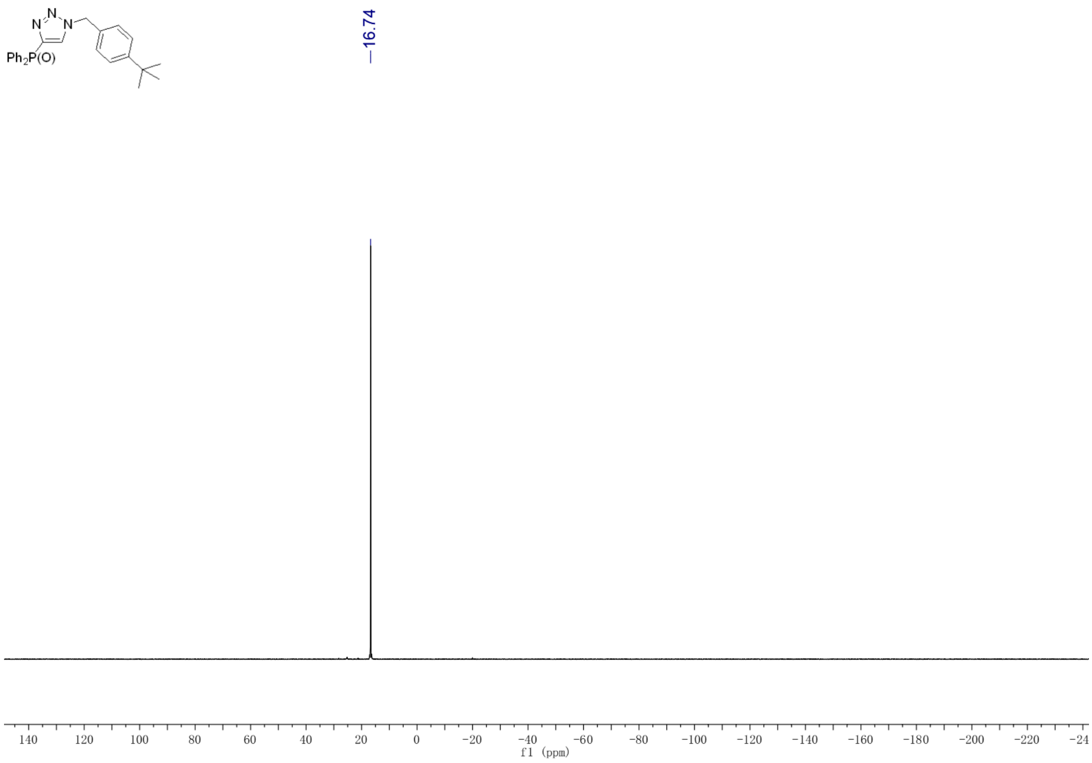

^{1}H-NMR (400 MHz, $CDCl_3$) spectrum of **3f**

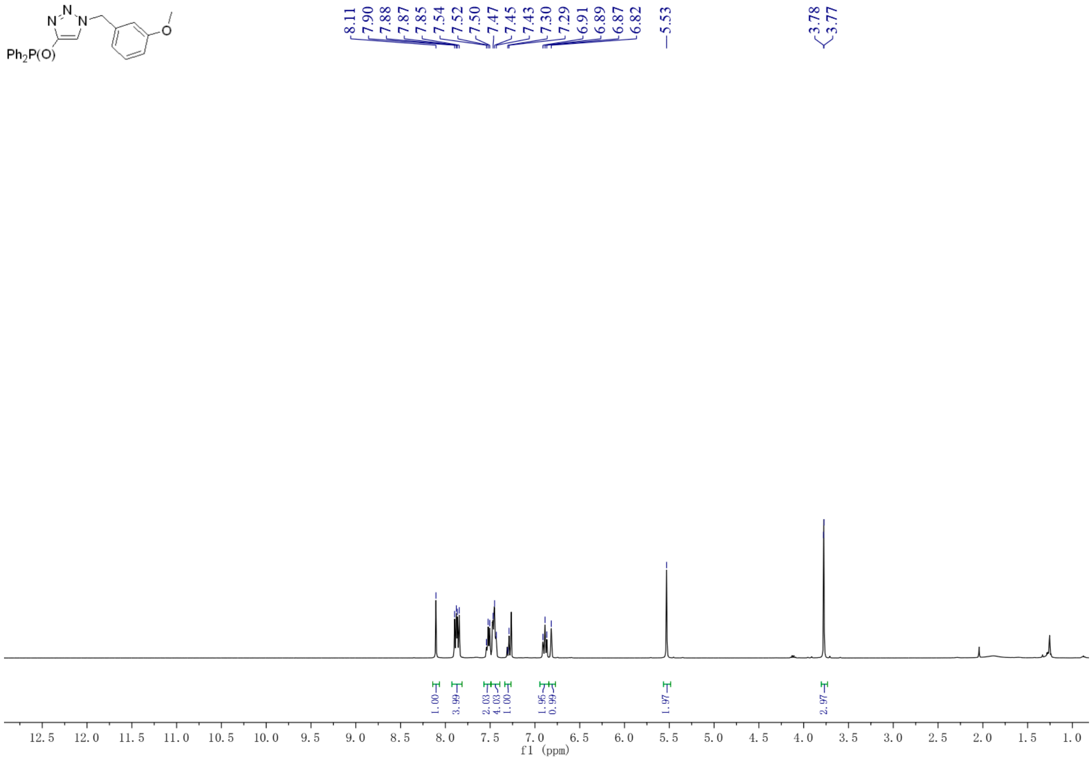

^{13}C-NMR (101 MHz, $CDCl_3$) spectrum of **3f**

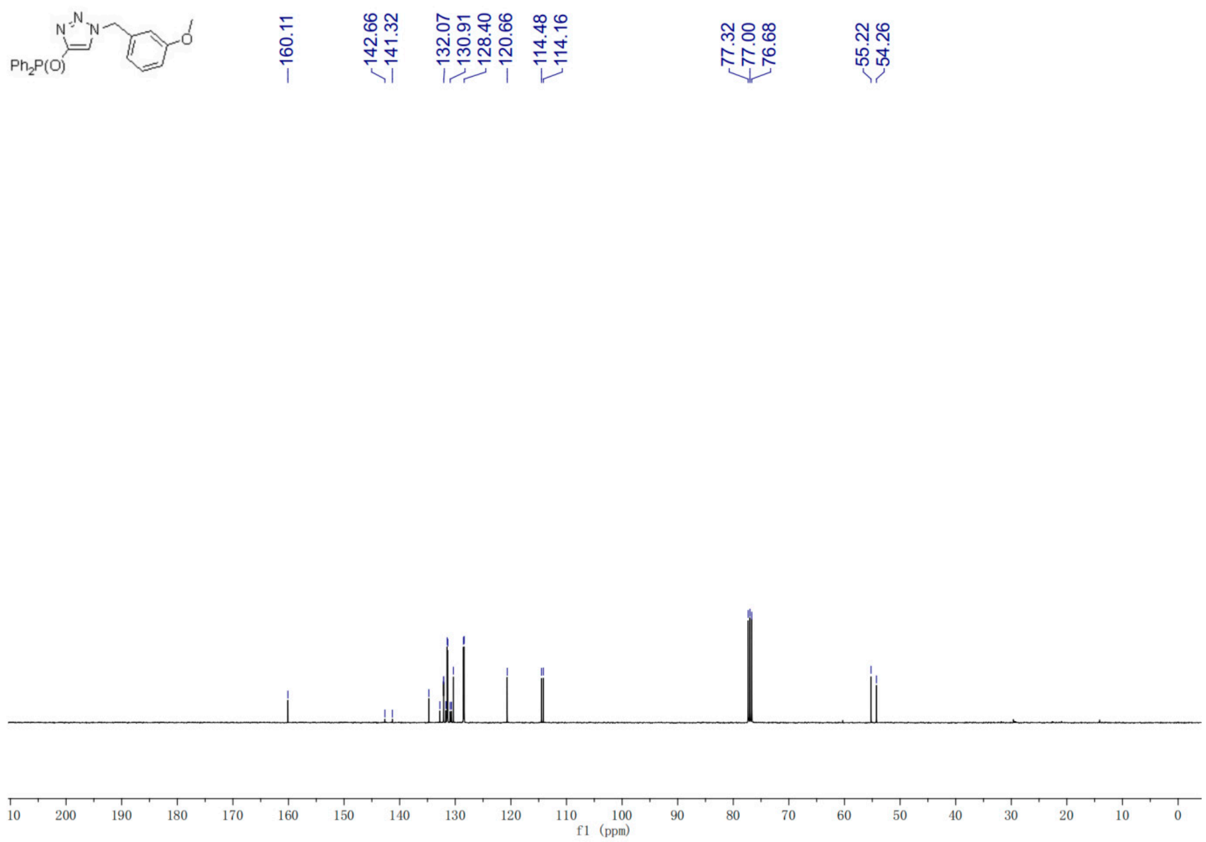

^{31}P NMR (162 MHz, Chloroform-*d*) spectrum of **3f**

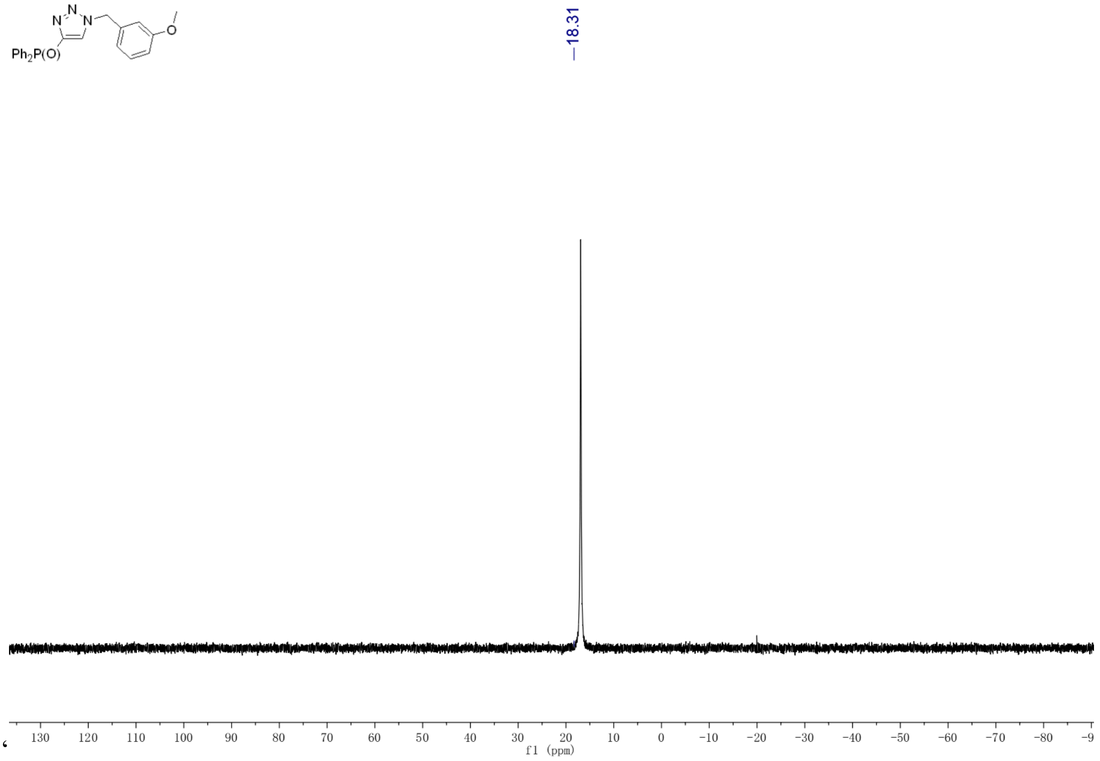

^{1}H-NMR (400 MHz, $CDCl_3$) spectrum of **3g**

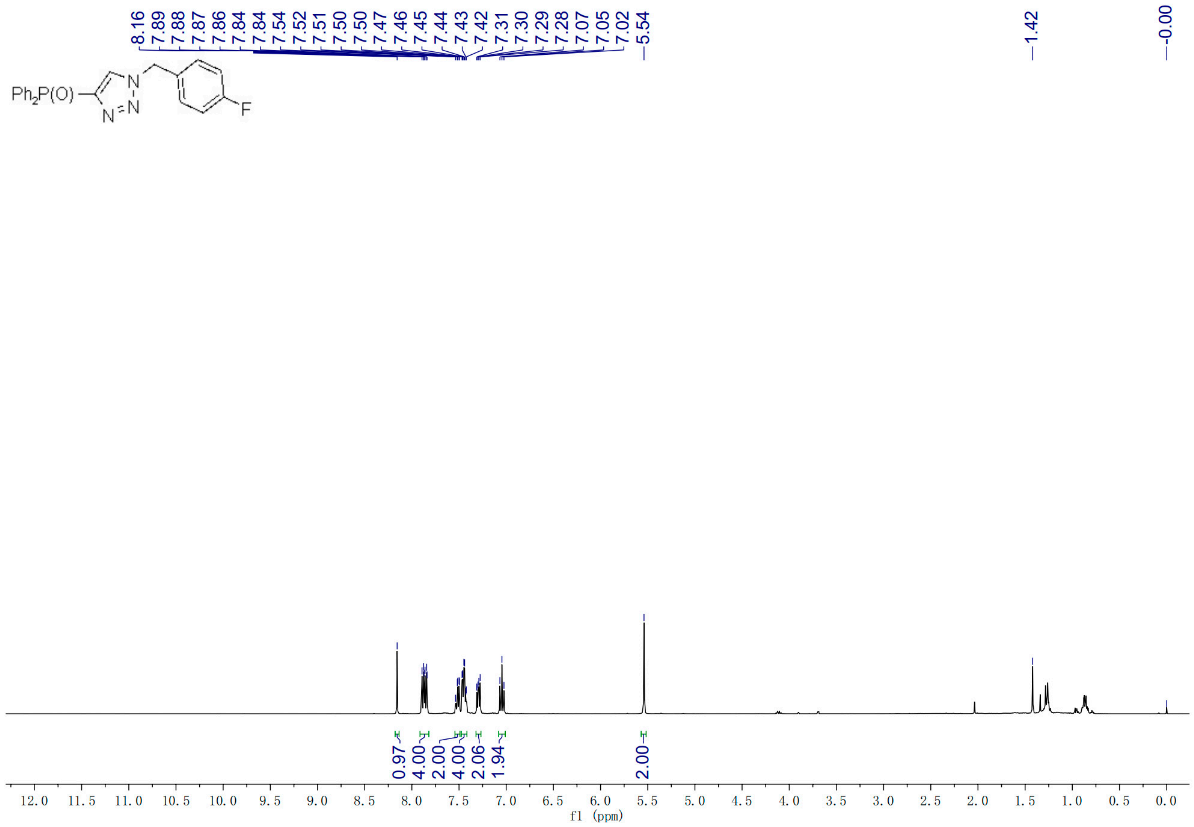

^{13}C-NMR (101 MHz, $CDCl_3$) spectrum of **3g**

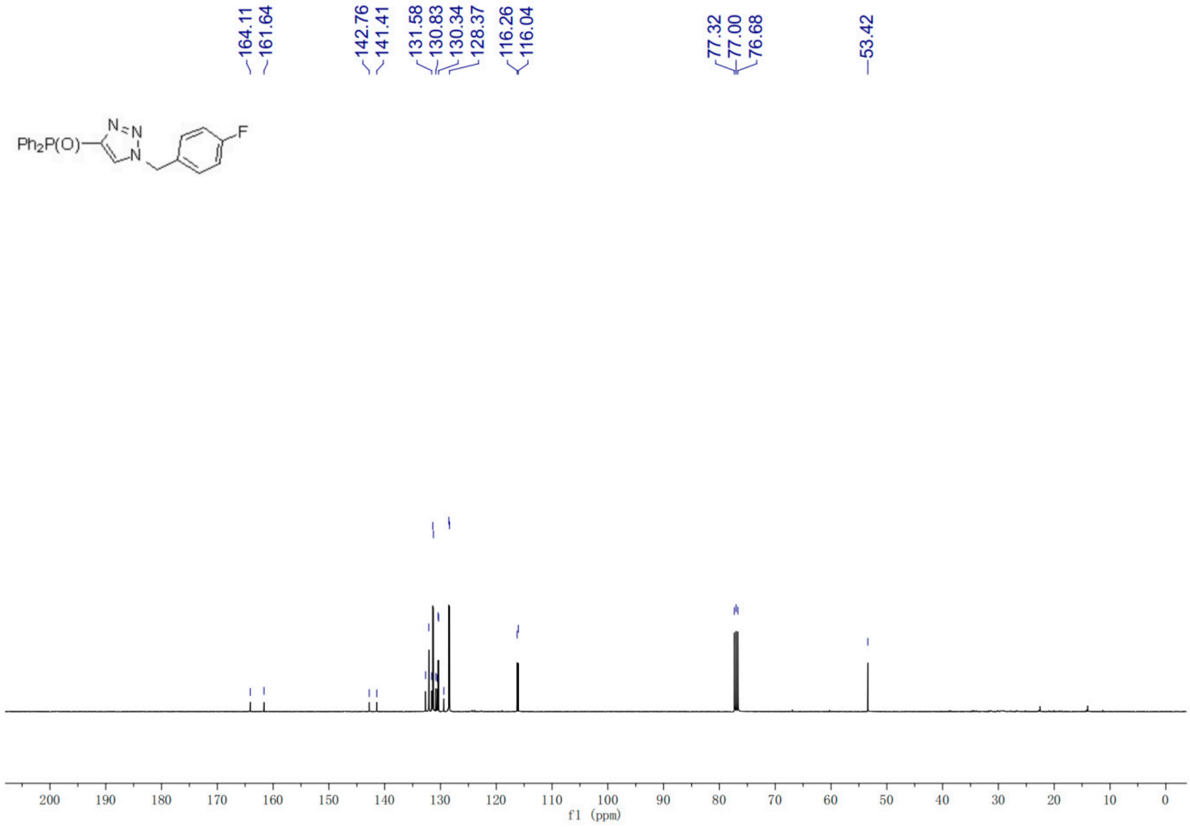

^{31}P NMR (162 MHz, Chloroform-*d*) spectrum of **3g**

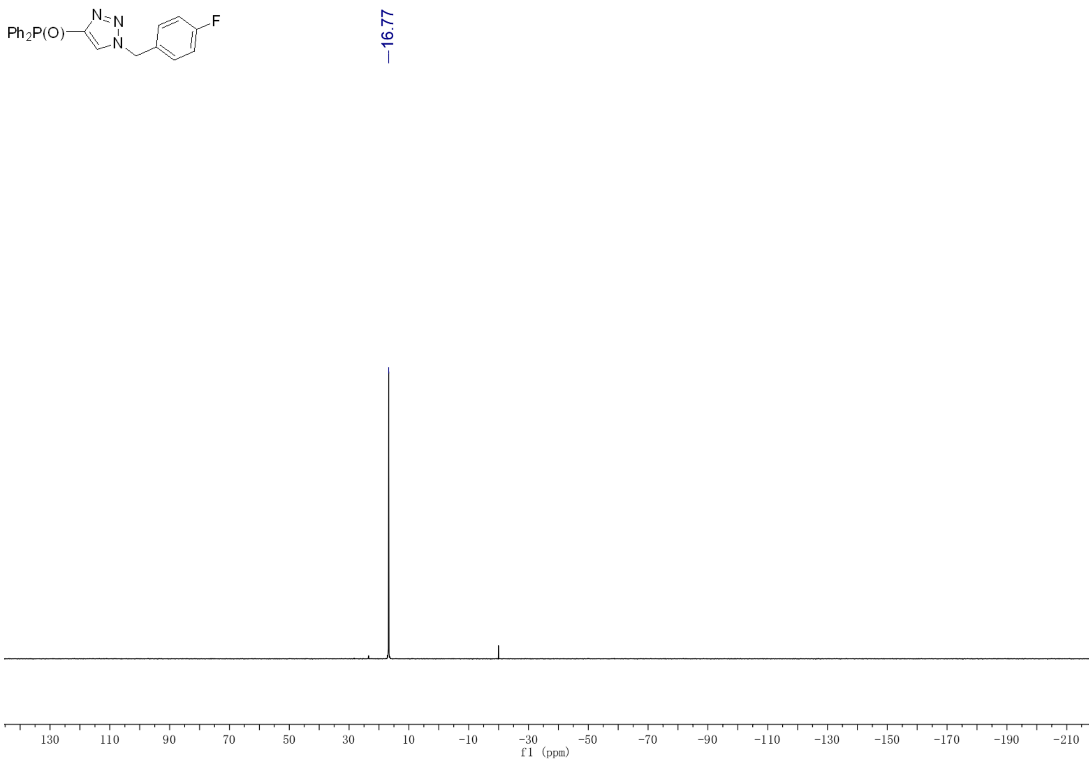

^{19}F NMR (376 MHz, Chloroform-*d*) spectrum of **3g**

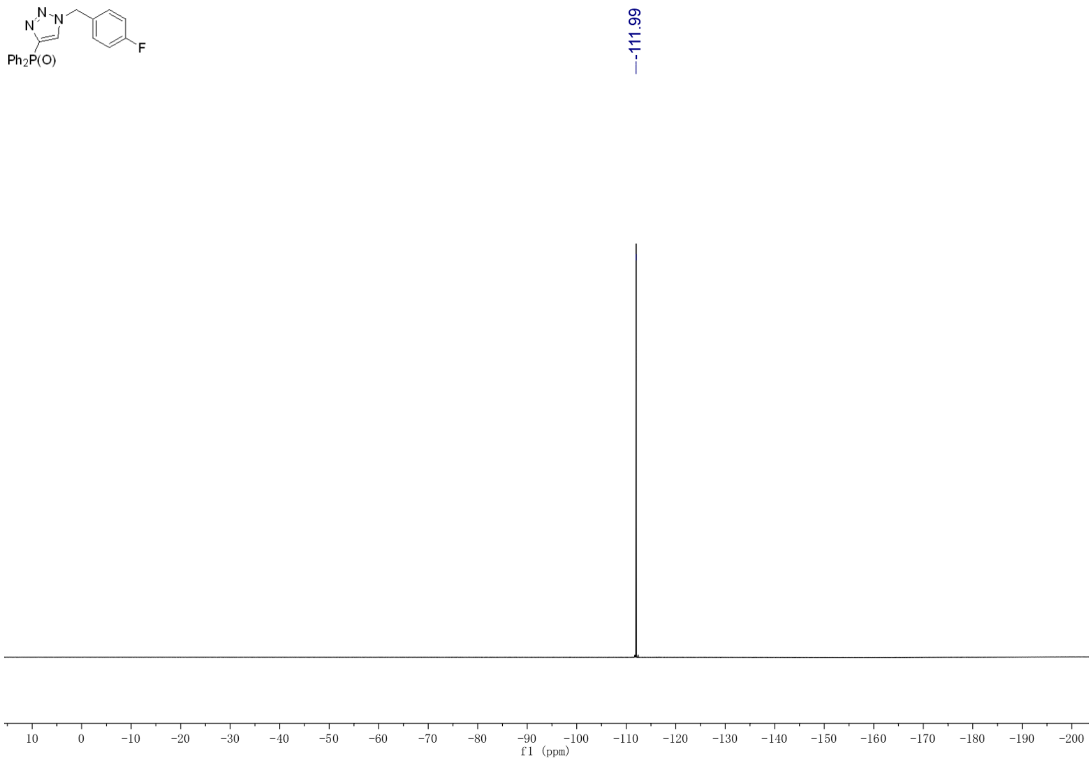

^{1}H-NMR (400 MHz, $CDCl_3$) spectrum of **3h**

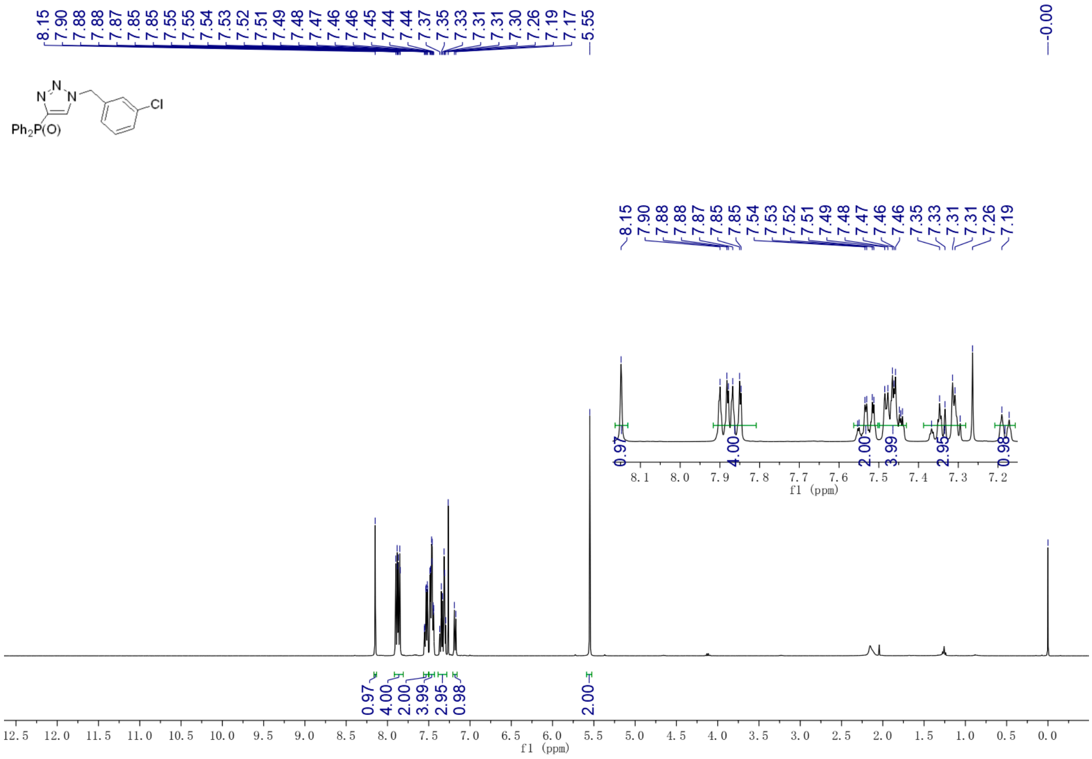

^{13}C-NMR (101 MHz, $CDCl_3$) spectrum of **3h**

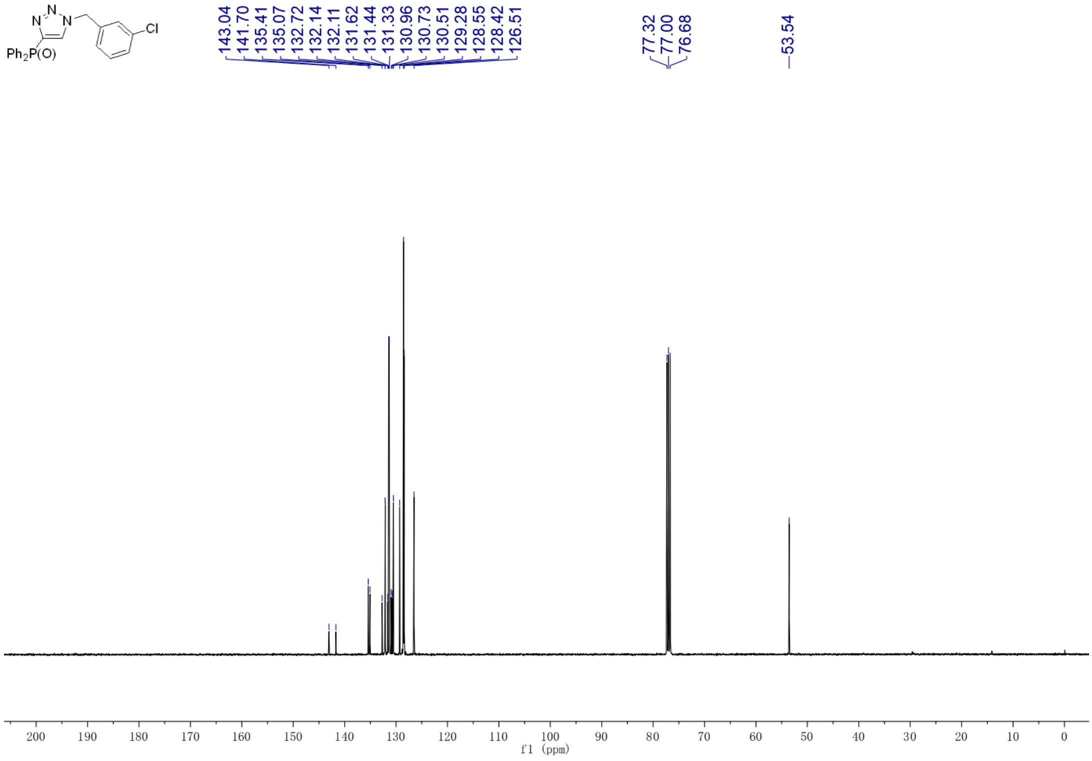

^{31}P NMR (162 MHz, Chloroform-*d*) spectrum of **3h**

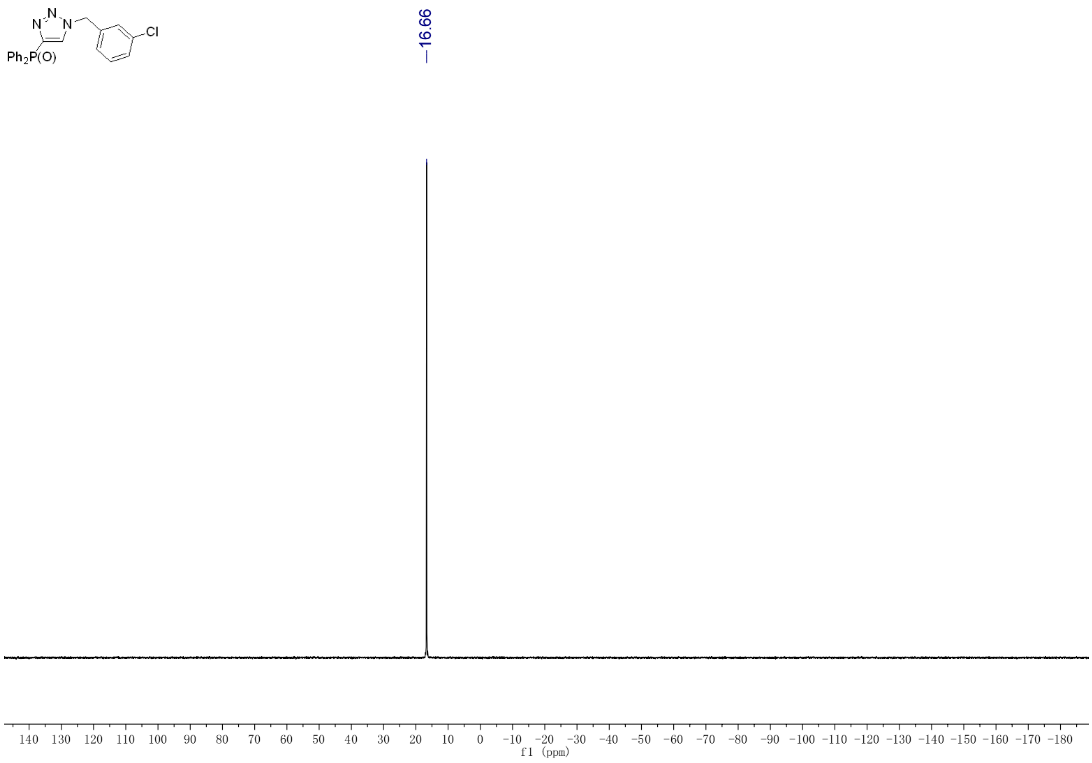

^{1}H-NMR (400 MHz, $CDCl_3$) spectrum of **3i**

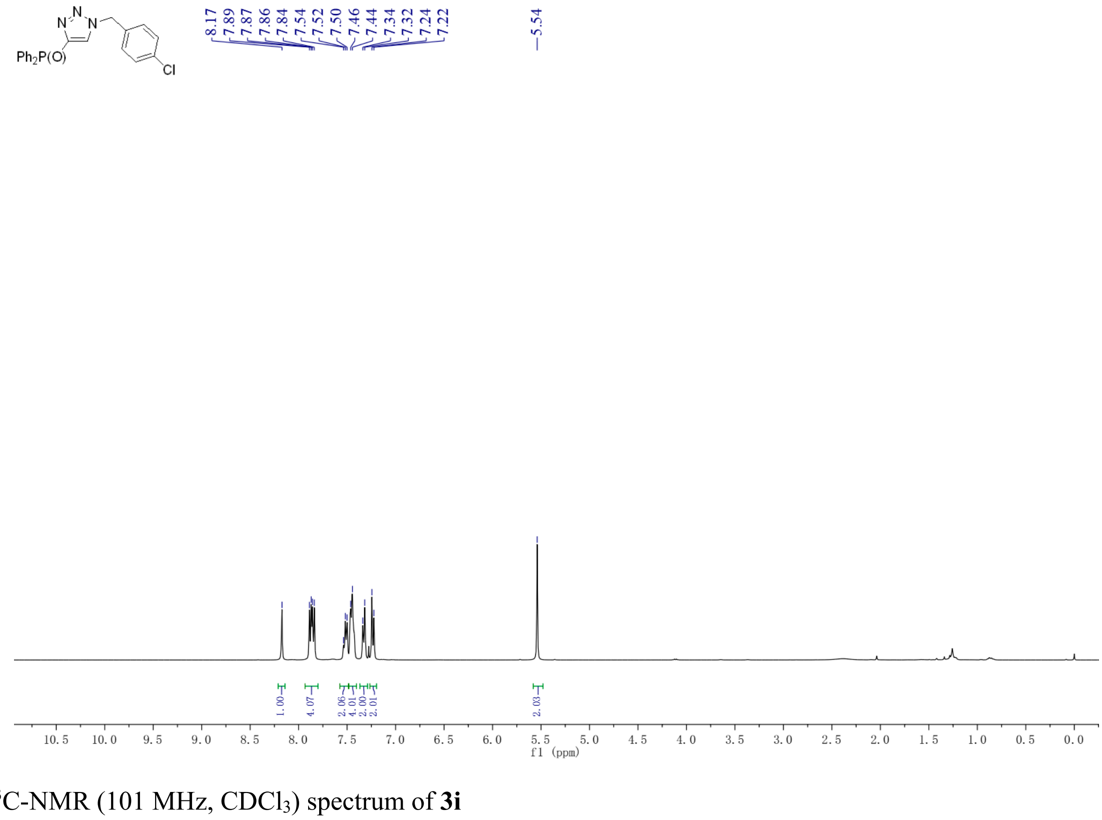

^{13}C-NMR (101 MHz, $CDCl_3$) spectrum of **3i**

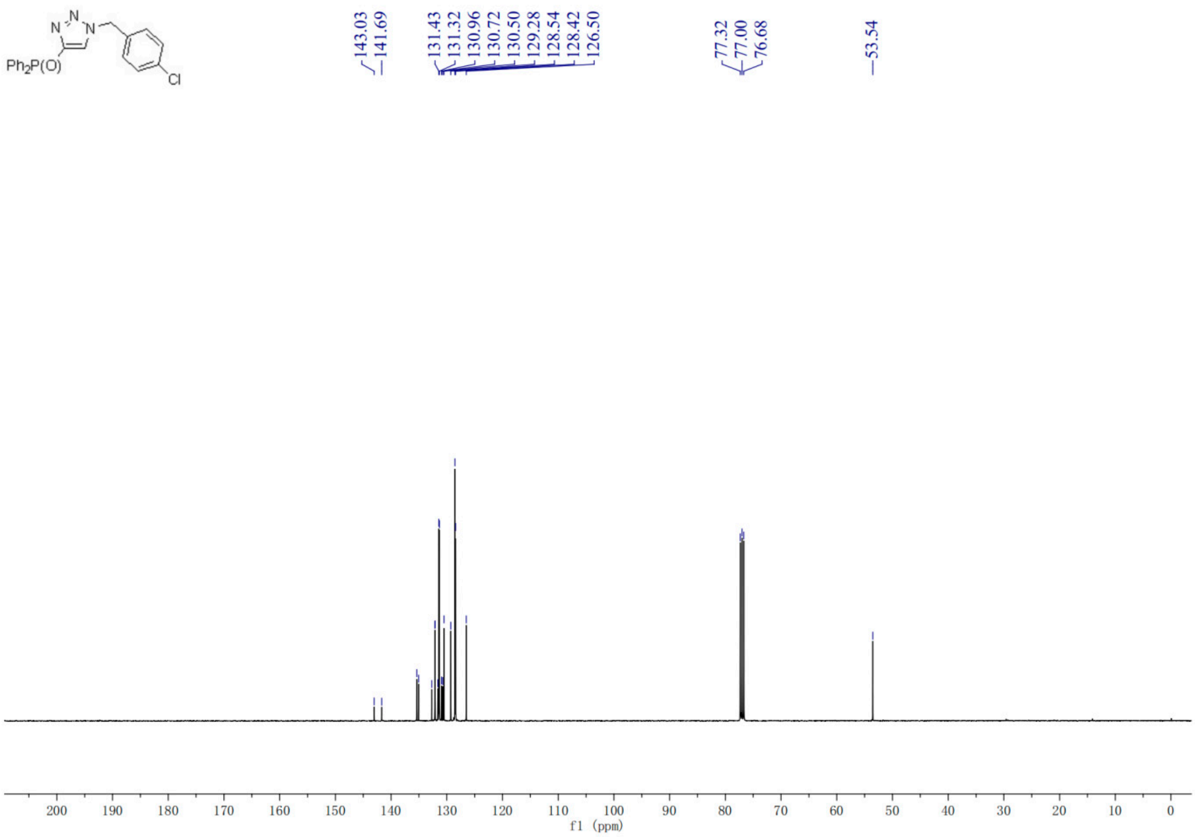

^{31}P NMR (162 MHz, Chloroform-*d*) spectrum of **3i**

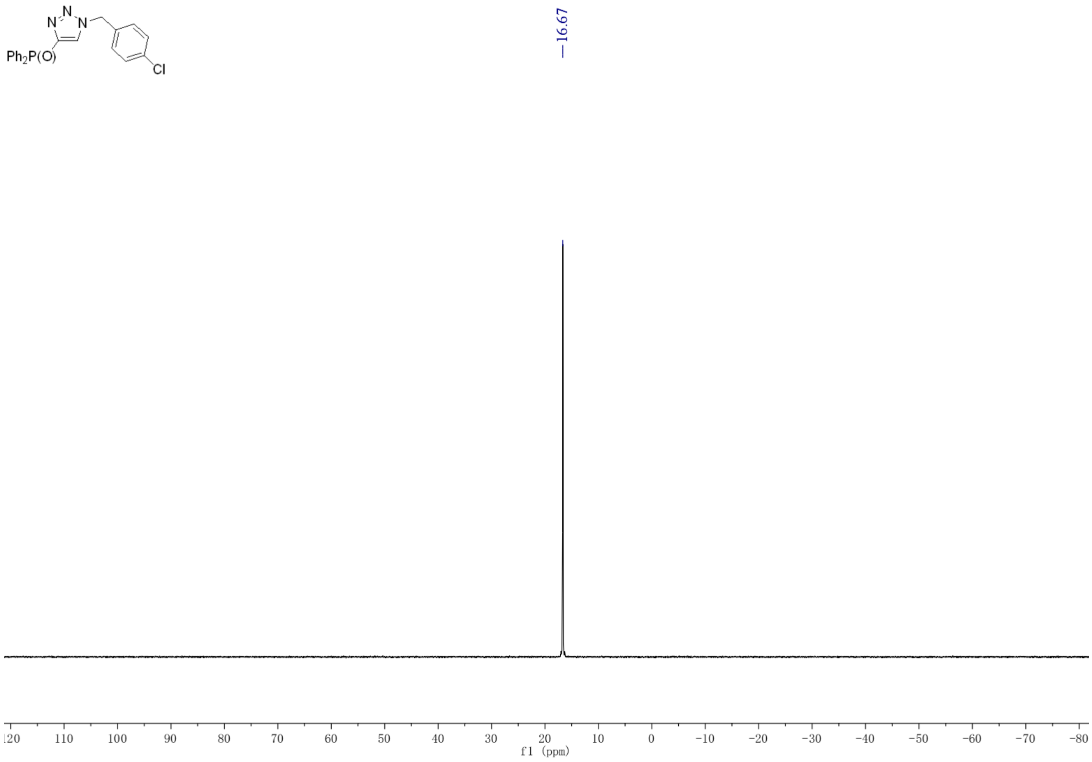

^{1}H-NMR (400 MHz, $CDCl_3$) spectrum of **3j**

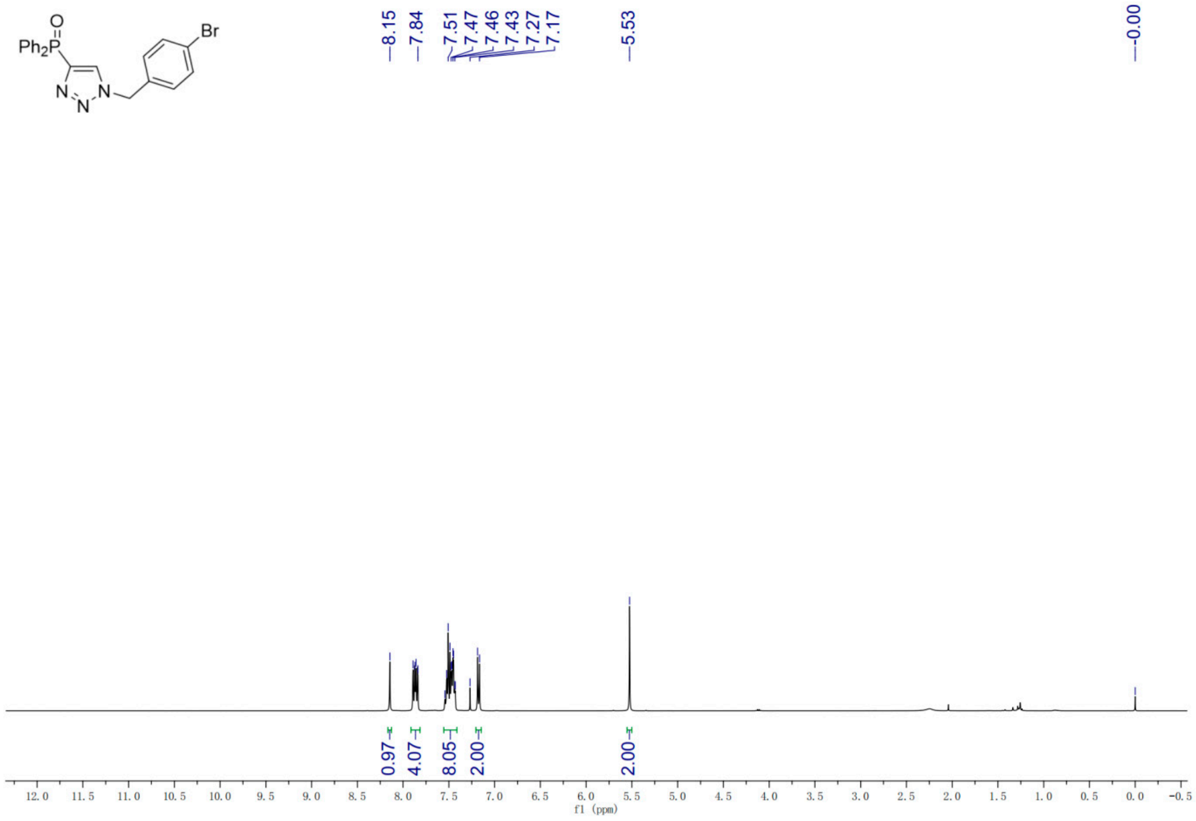

^{13}C-NMR (101 MHz, $CDCl_3$) spectrum of **3j**

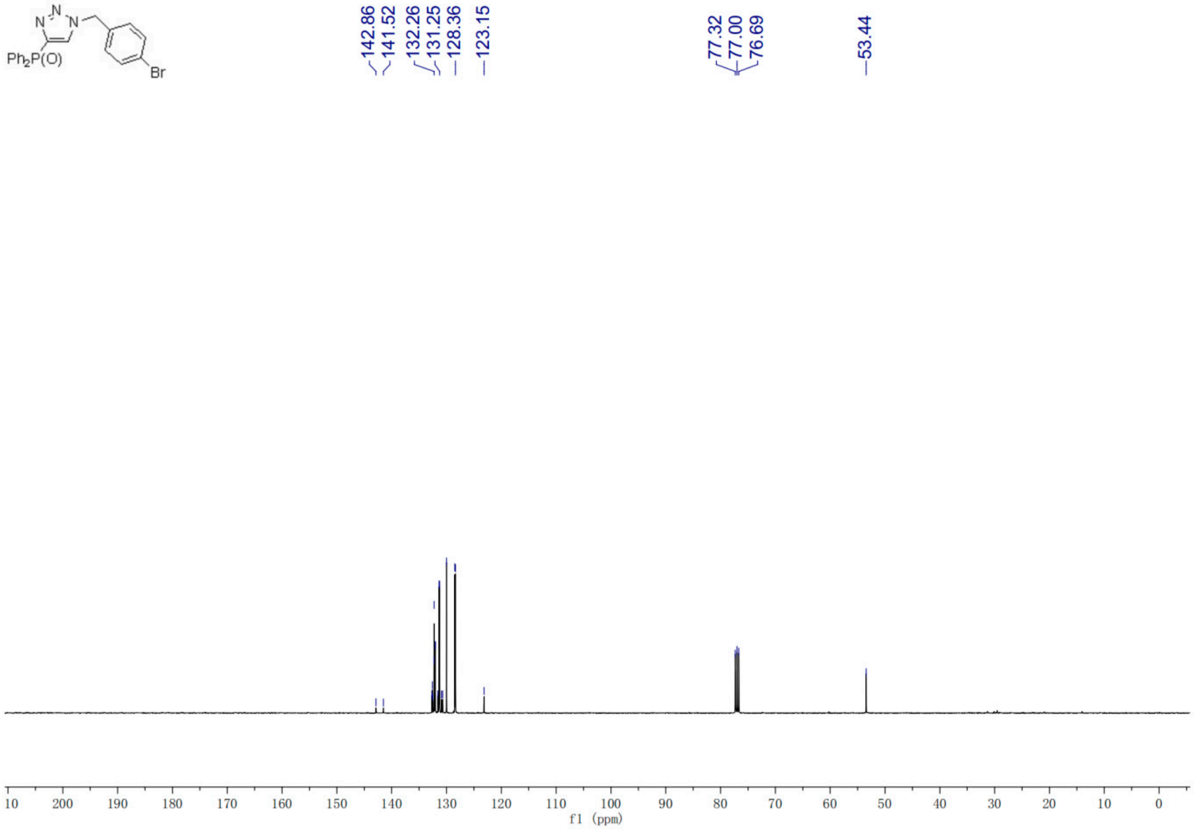

^{31}P NMR (162 MHz, Chloroform-*d*) spectrum of **3j**

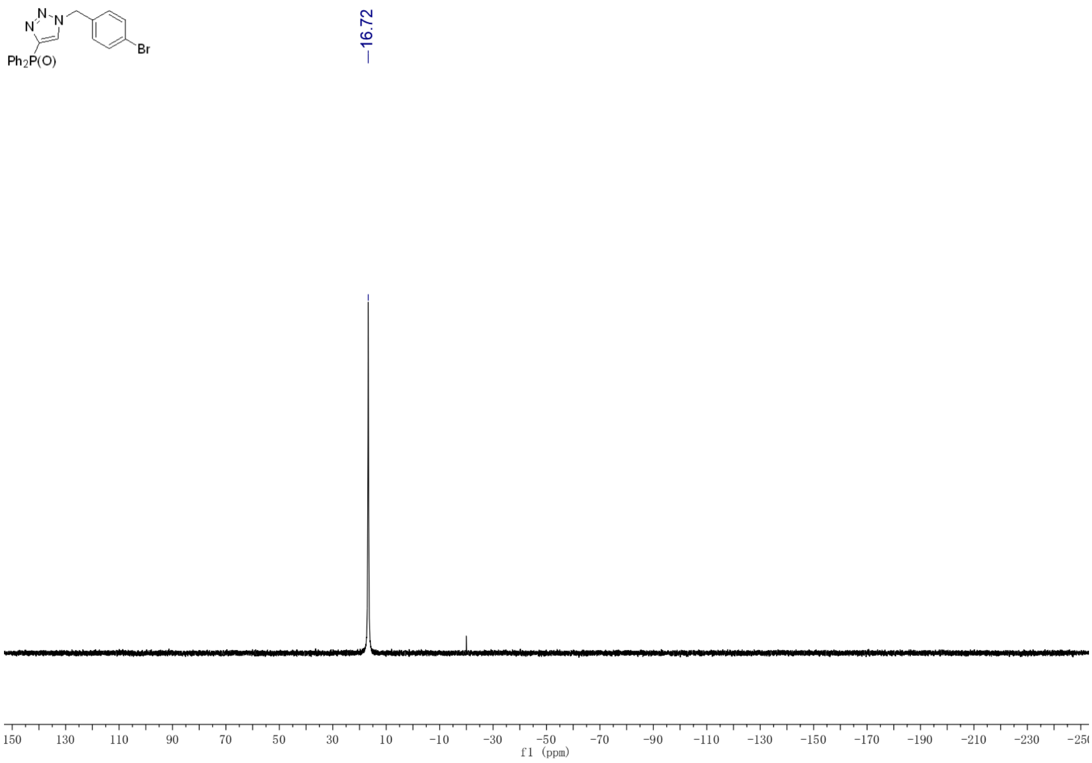

^{1}H-NMR (400 MHz, CDCl$_3$) spectrum of **3k**

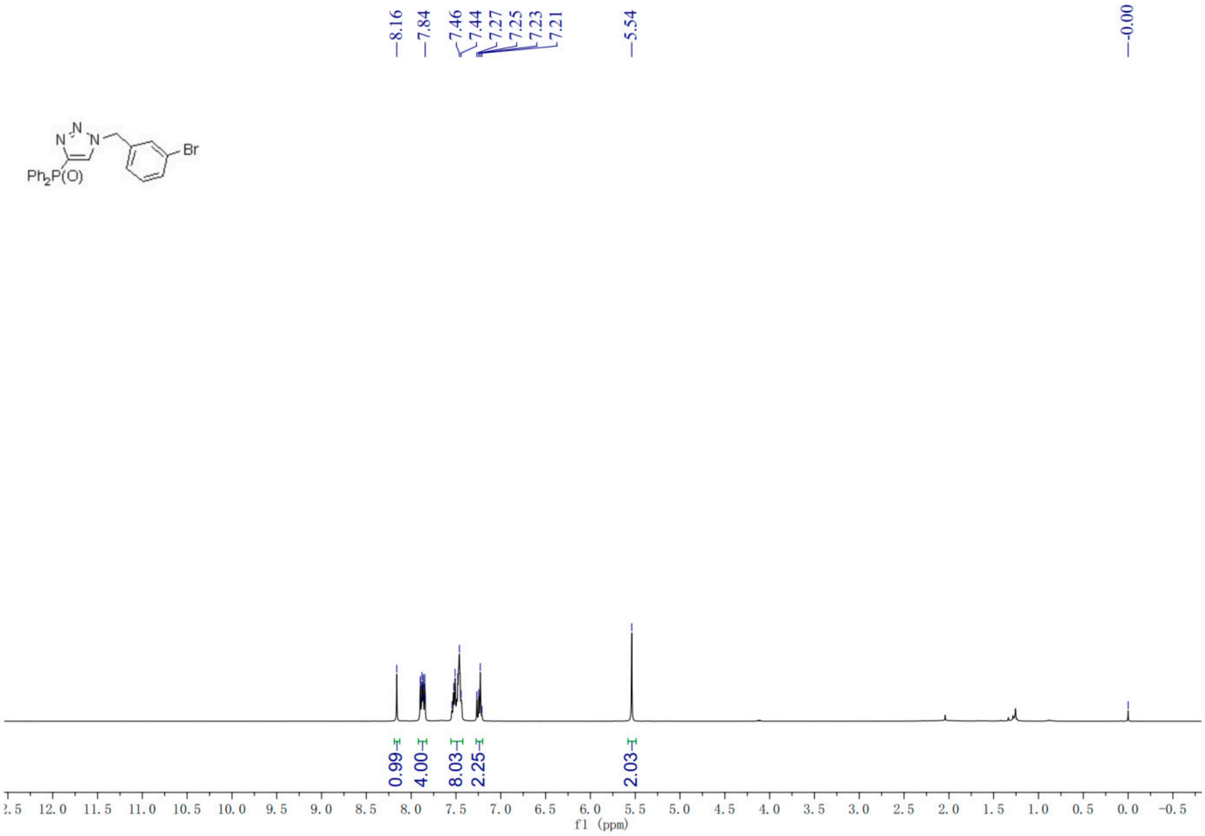

^{13}C-NMR (101 MHz, CDCl$_3$) spectrum of **3k**

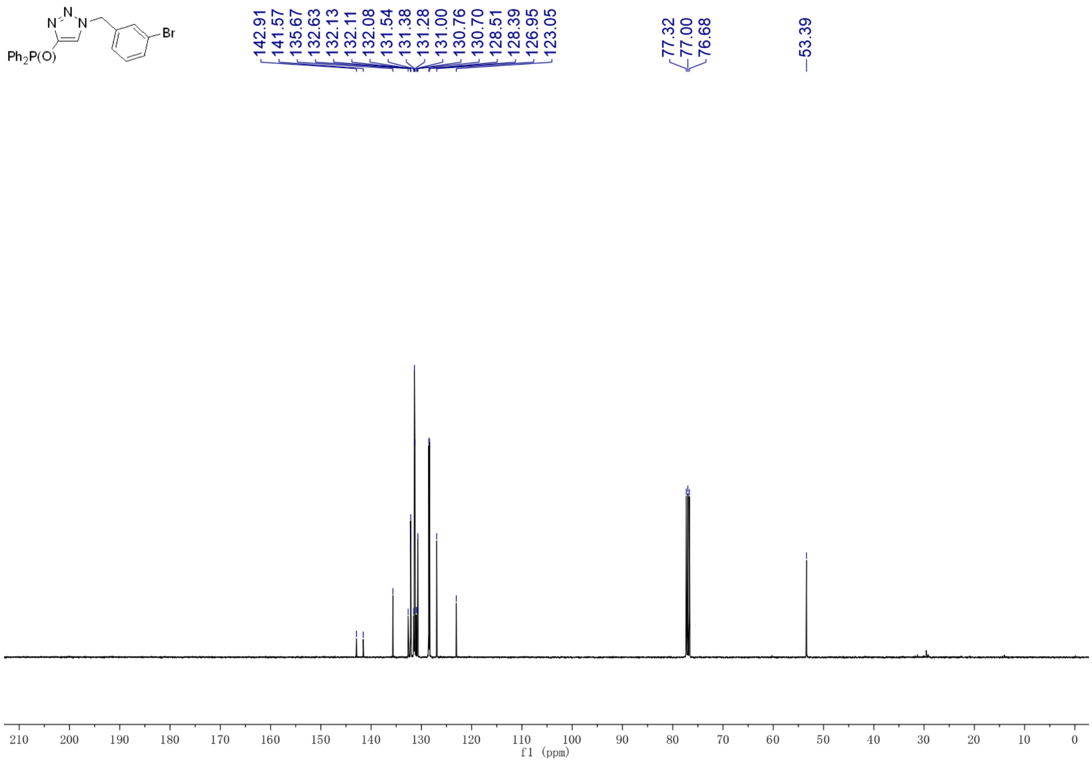

^{31}P NMR (162 MHz, Chloroform-*d*) spectrum of **3k**

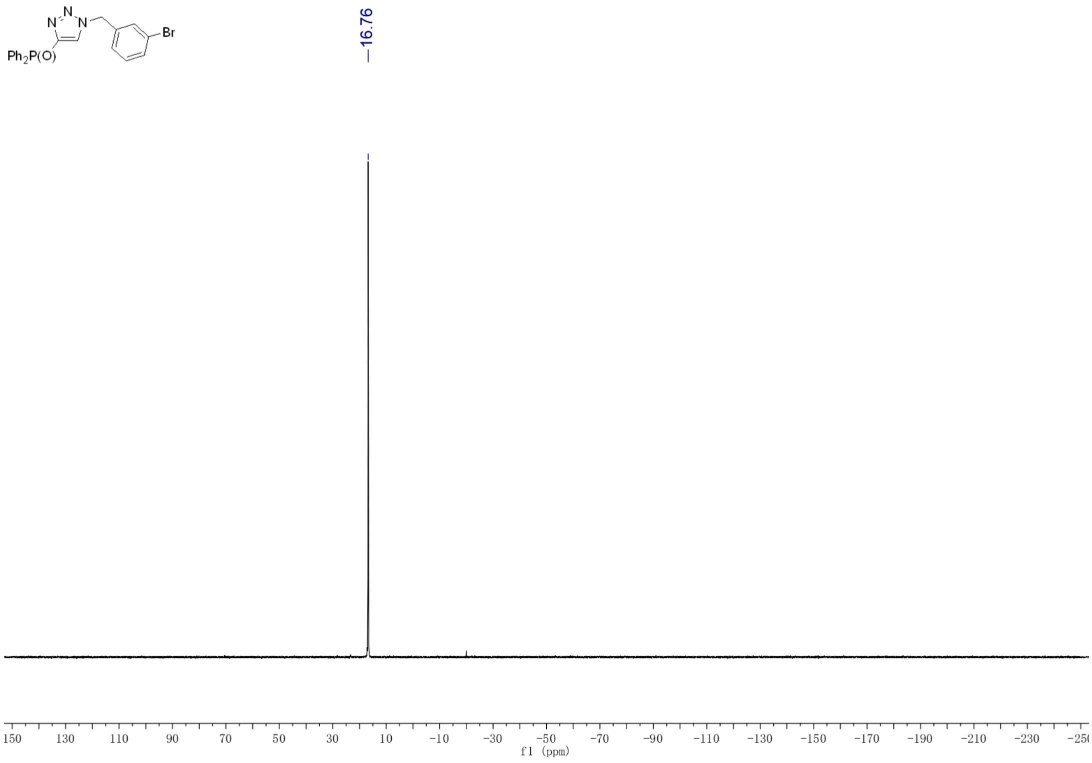

^{1}H-NMR (400 MHz, $CDCl_3$) spectrum of **3l**

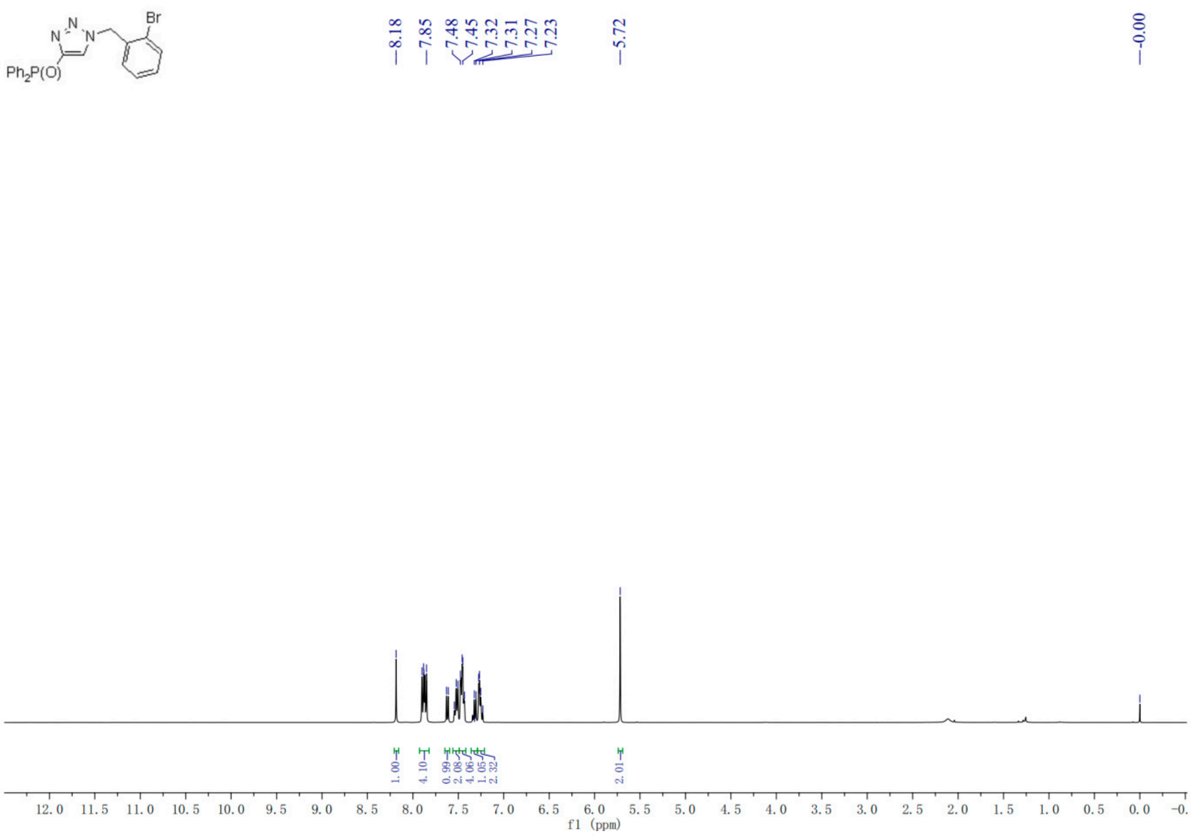

^{13}C-NMR (101 MHz, $CDCl_3$) spectrum of **3l**

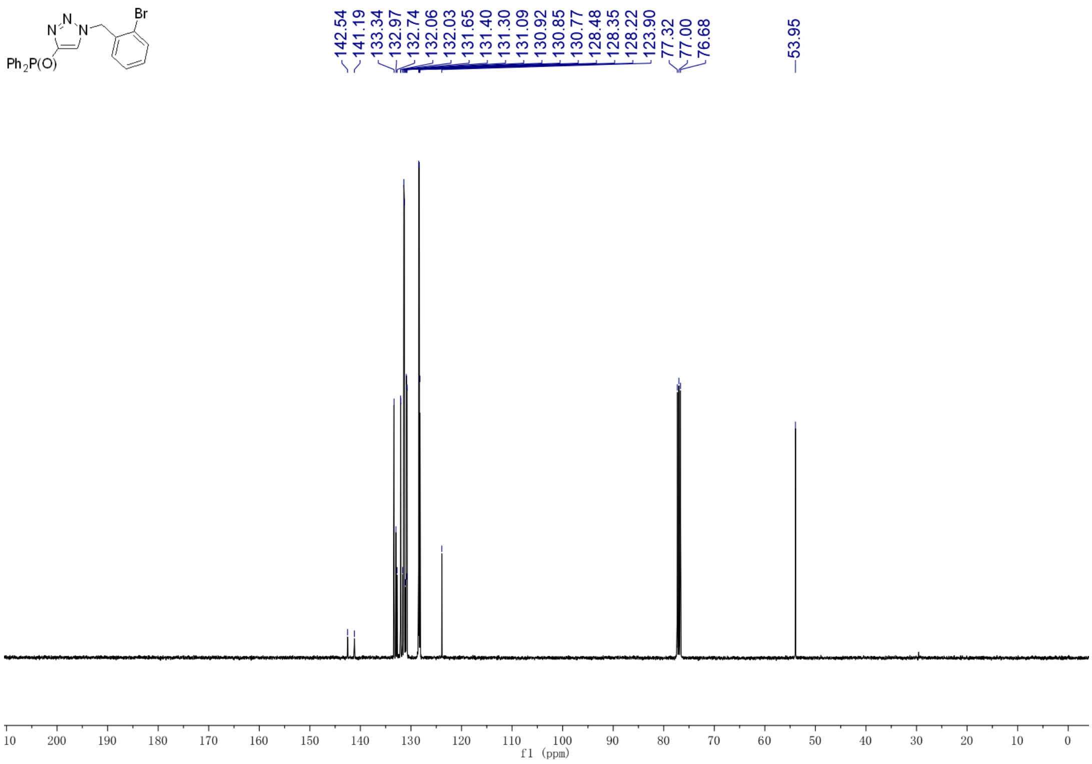

^{31}P NMR (162 MHz, Chloroform-*d*) spectrum of **3l**

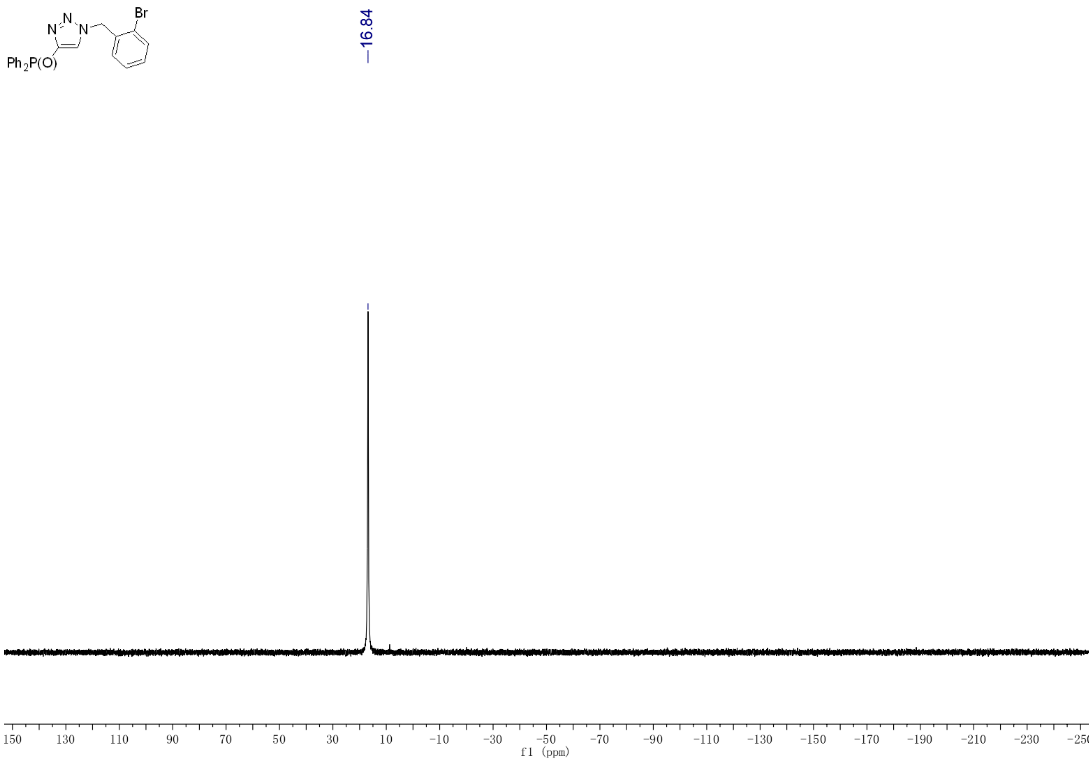

^{1}H-NMR (400 MHz, $CDCl_3$) spectrum of **3m**

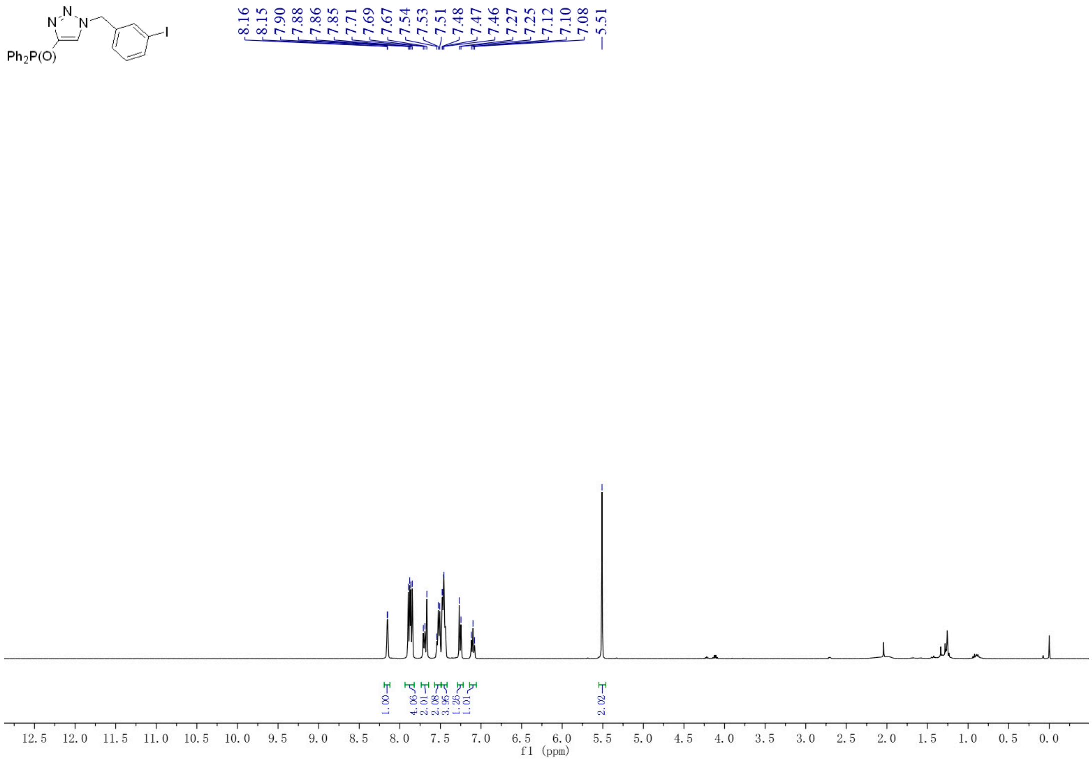

^{13}C-NMR (101 MHz, $CDCl_3$) spectrum of **3m**

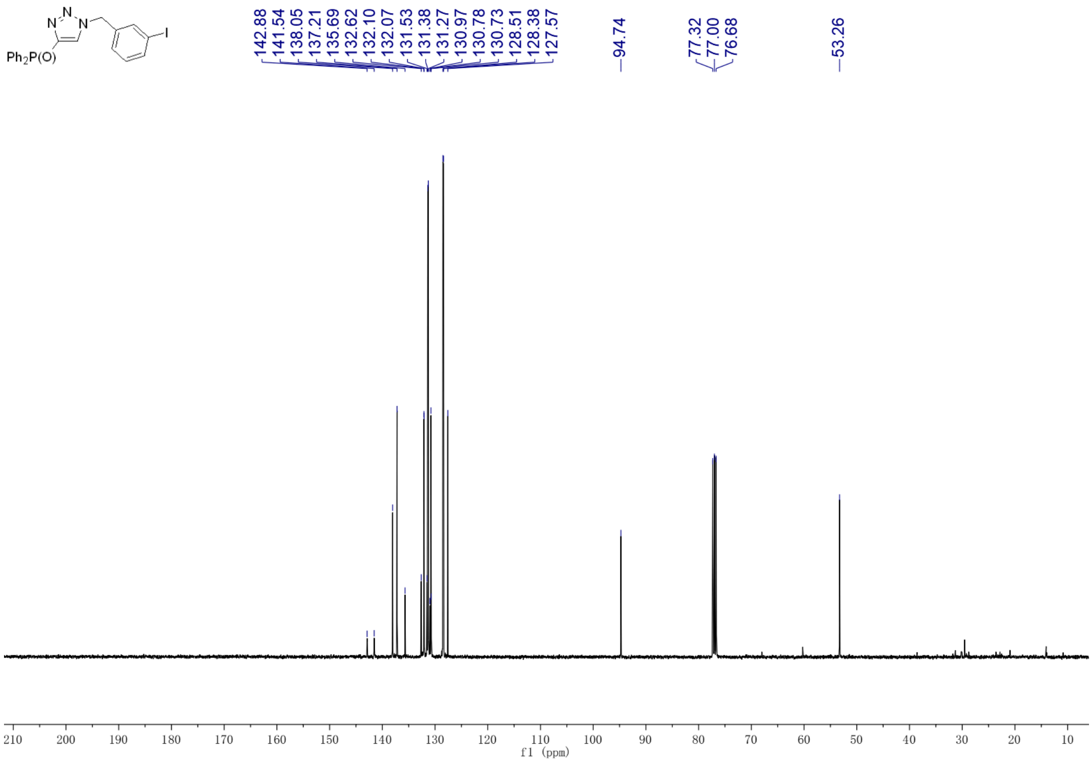

^{31}P NMR (162 MHz, Chloroform-*d*) spectrum of **3m**

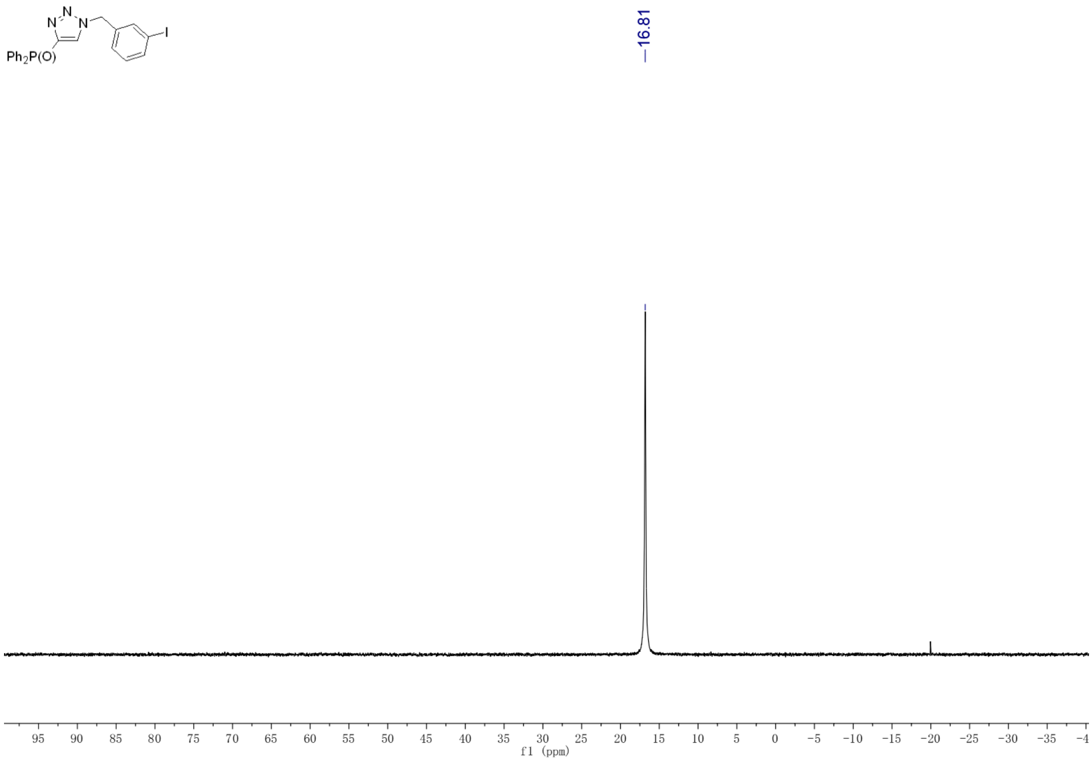

^{1}H-NMR (400 MHz, $CDCl_3$) spectrum of **3n**

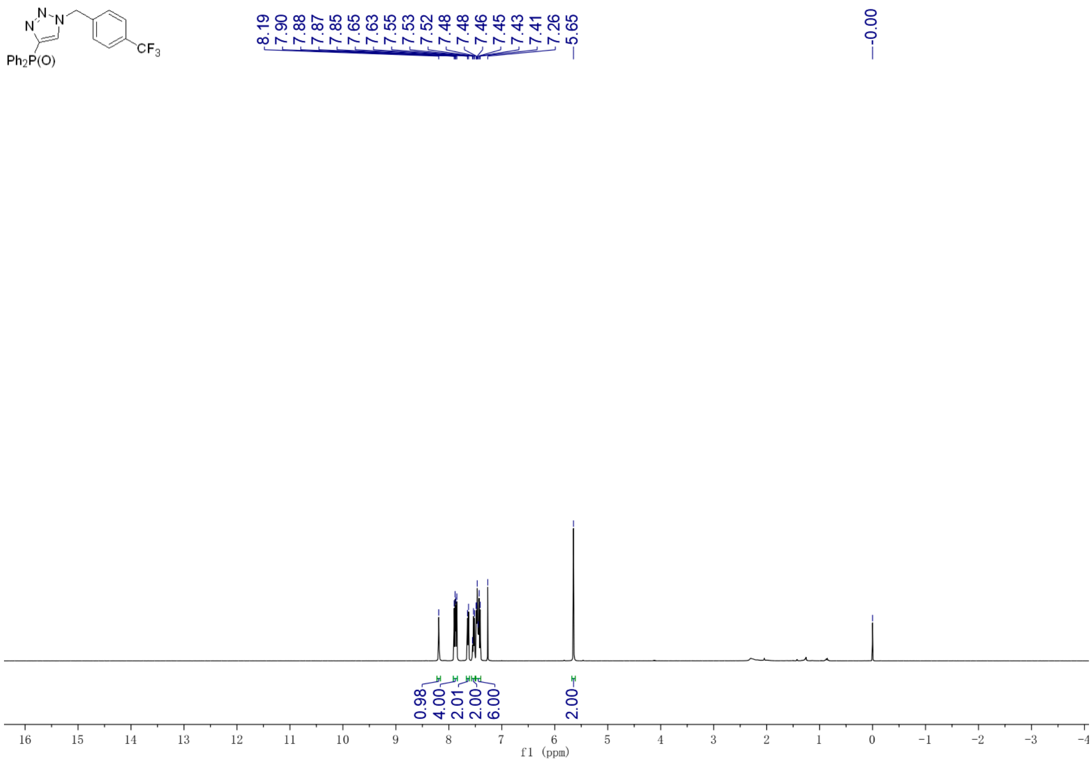

^{13}C-NMR (101 MHz, $CDCl_3$) spectrum of **3n**

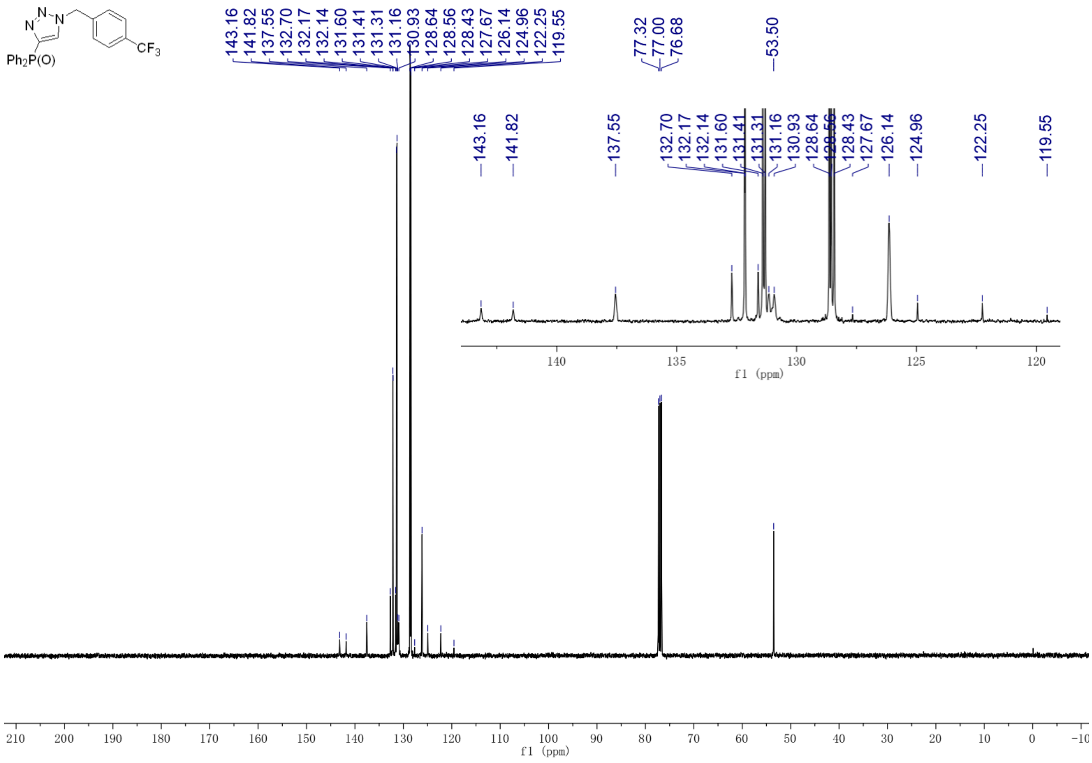

^{31}P NMR (162 MHz, Chloroform-*d*) spectrum of **3n**

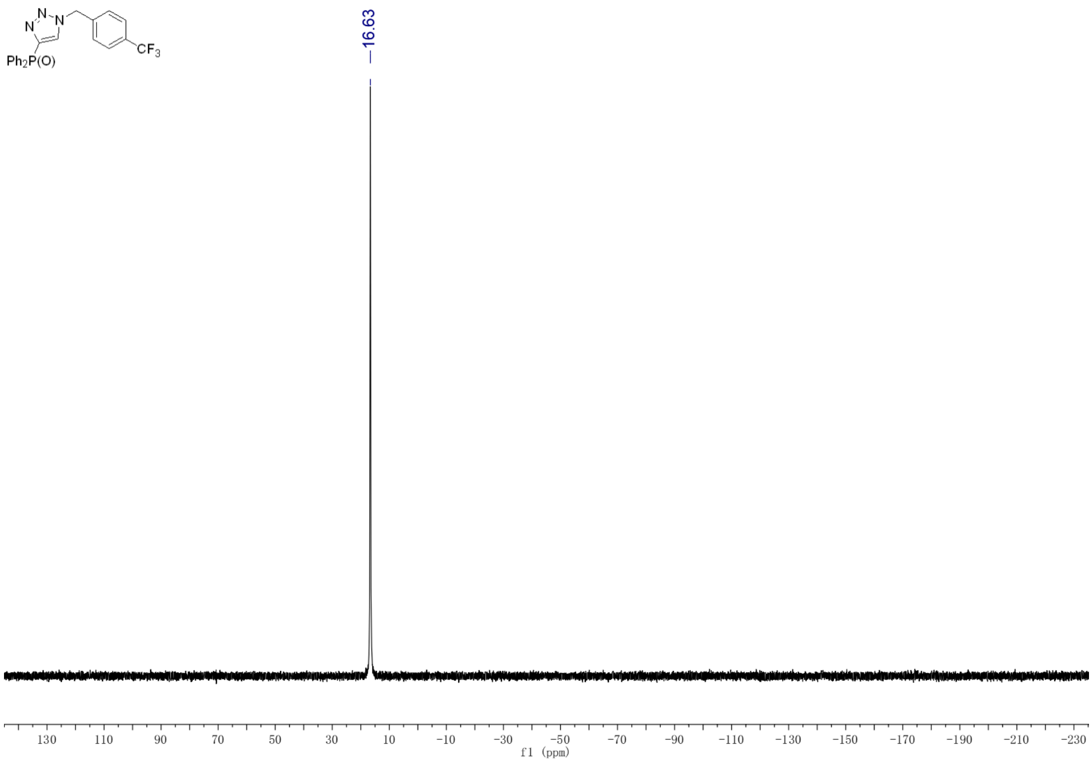

^{19}F NMR (376 MHz, Chloroform-*d*) spectrum of **3n**

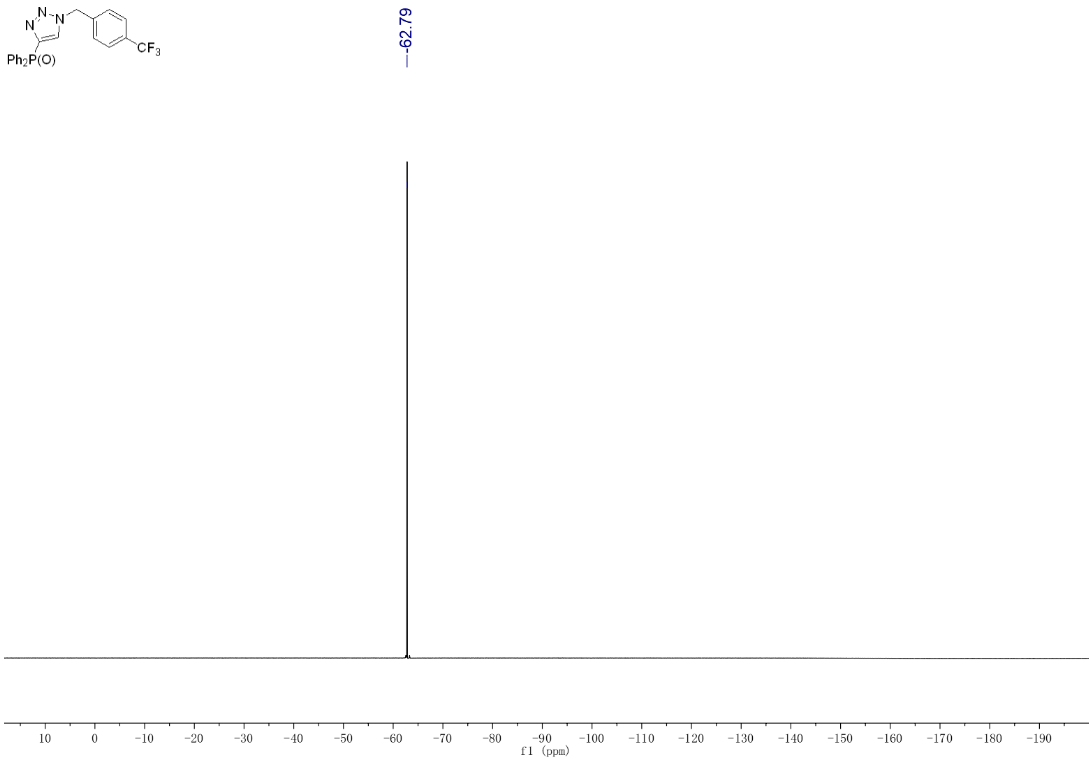

^{1}H-NMR (400 MHz, $CDCl_3$) spectrum of **3o**

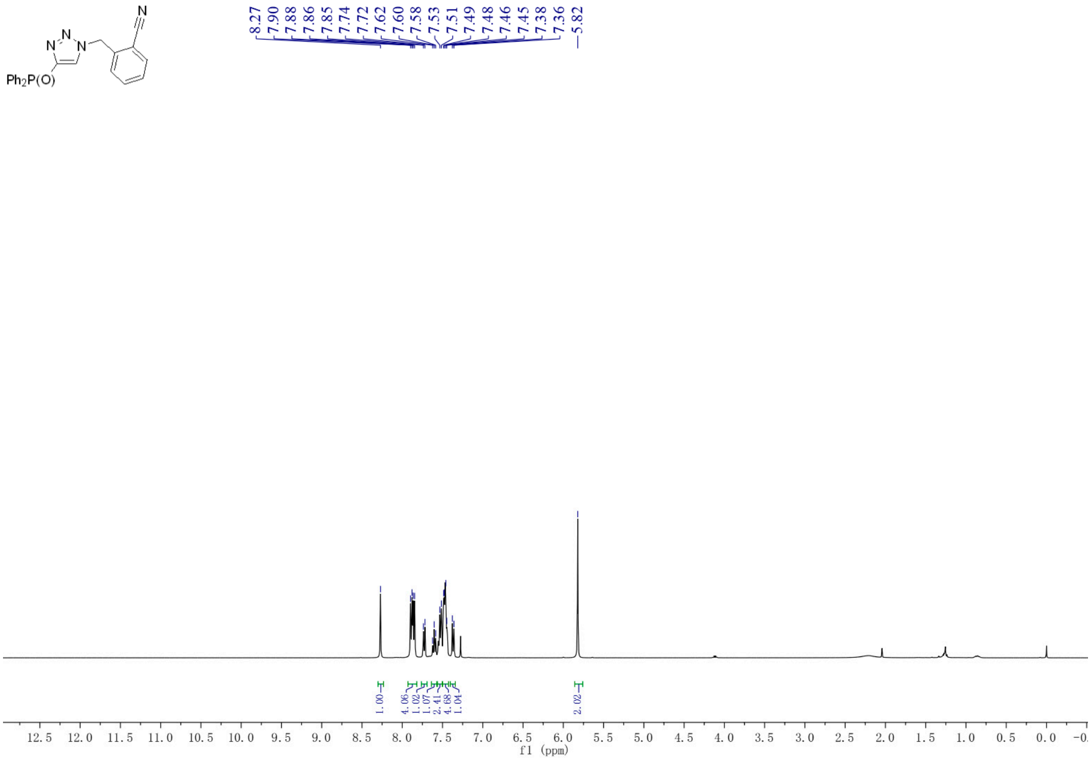

^{13}C-NMR (101 MHz, $CDCl_3$) spectrum of3**o**

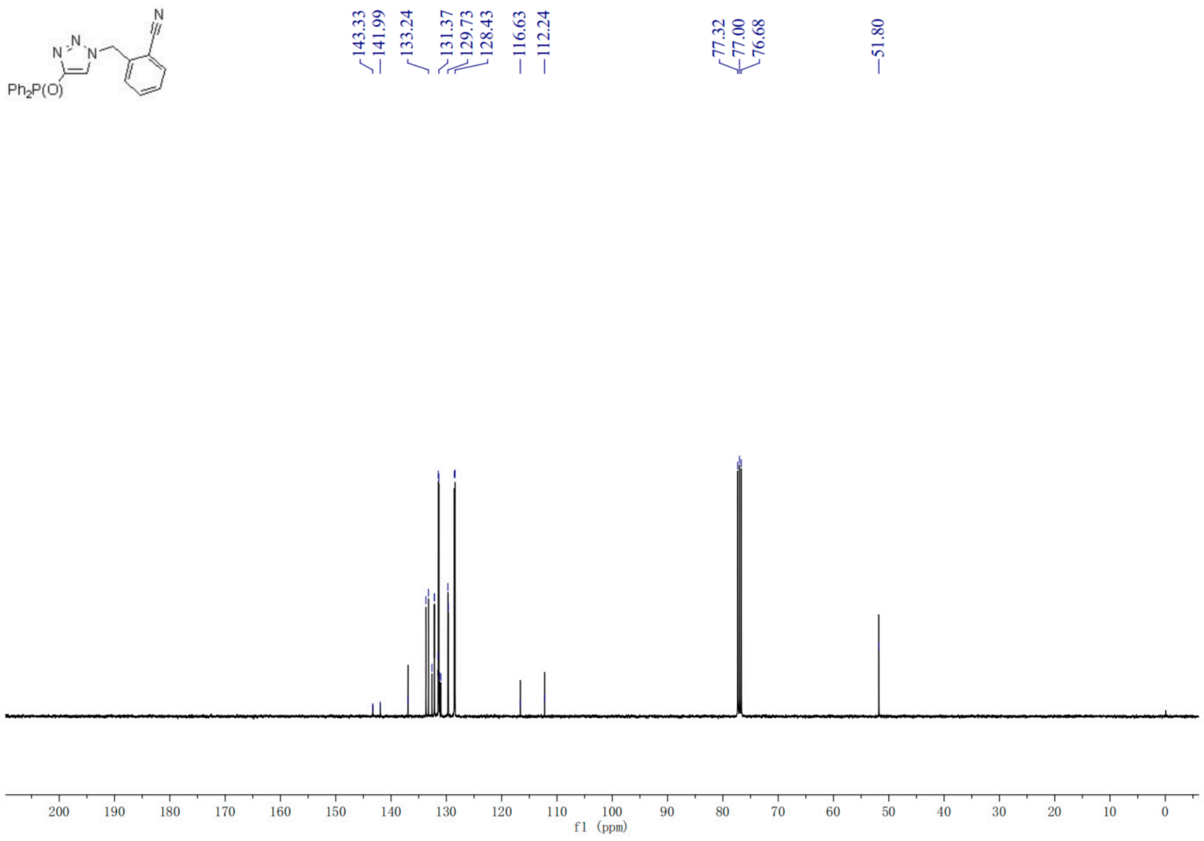

^{31}P NMR (162 MHz, Chloroform-*d*) spectrum of **3o**

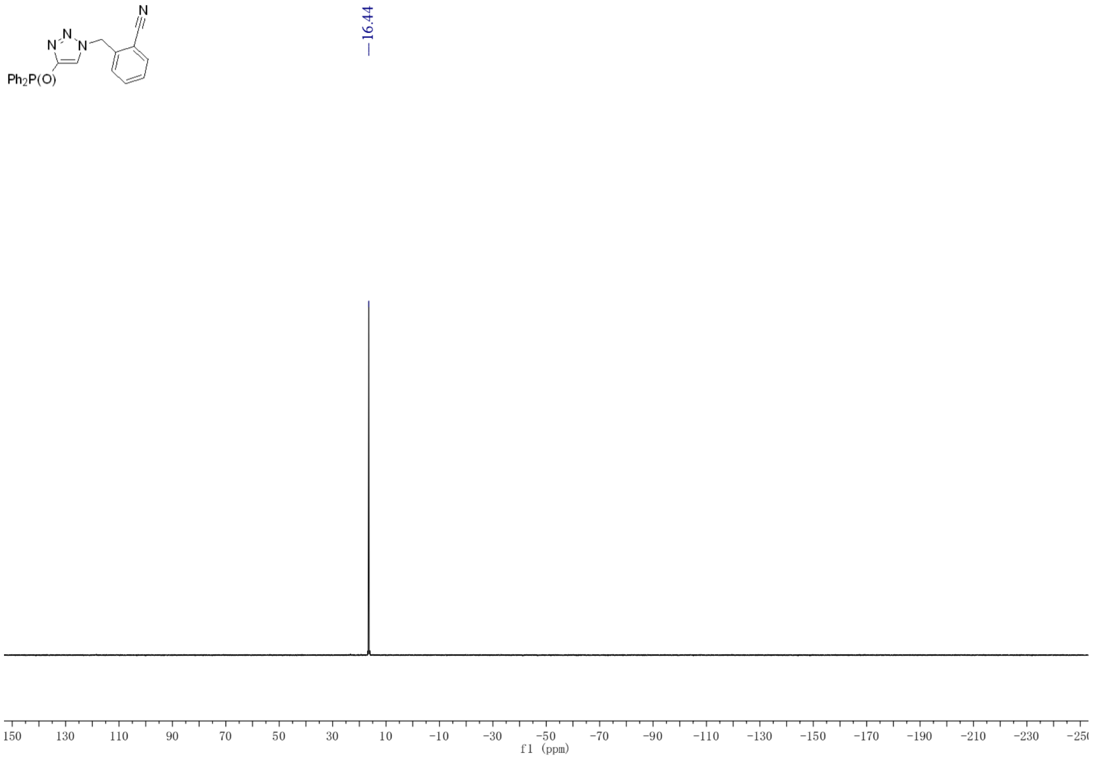

^{1}H-NMR (400 MHz, $CDCl_3$) spectrum of **3p**

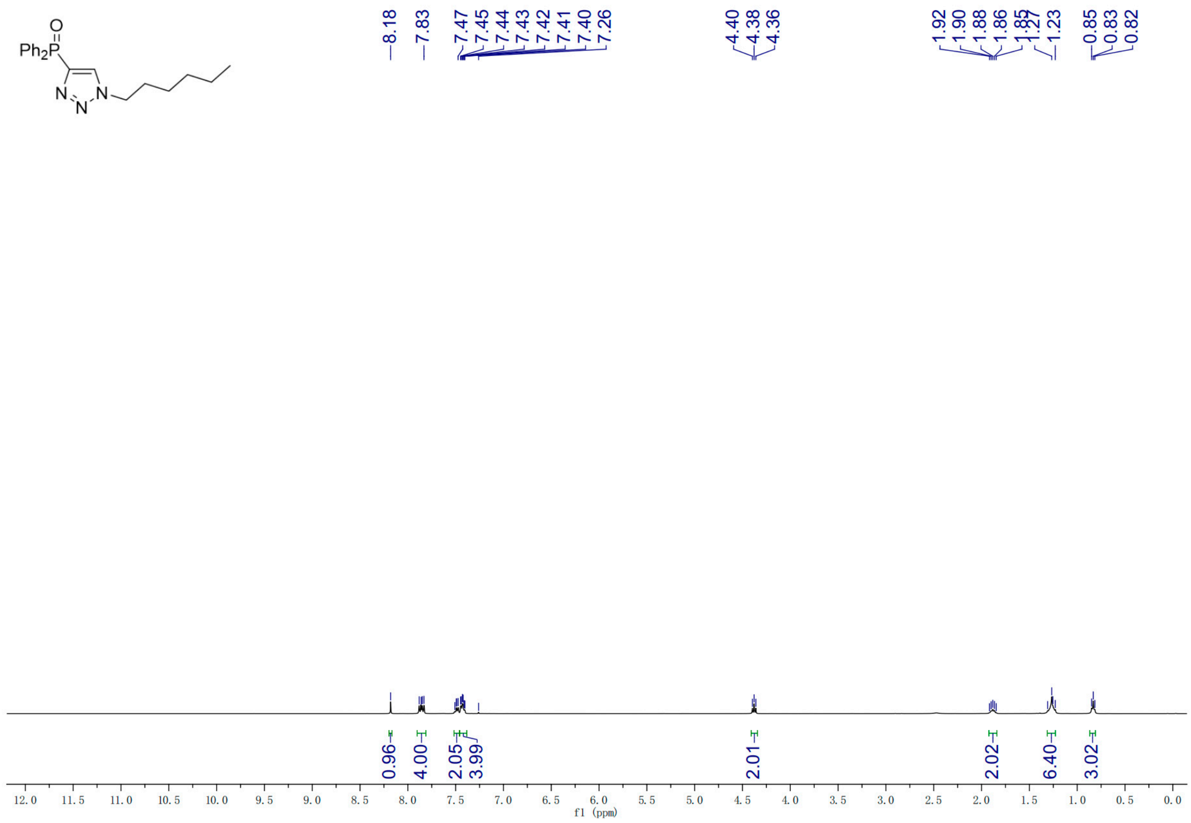

^{13}C-NMR (101 MHz, $CDCl_3$) spectrum of **3p**

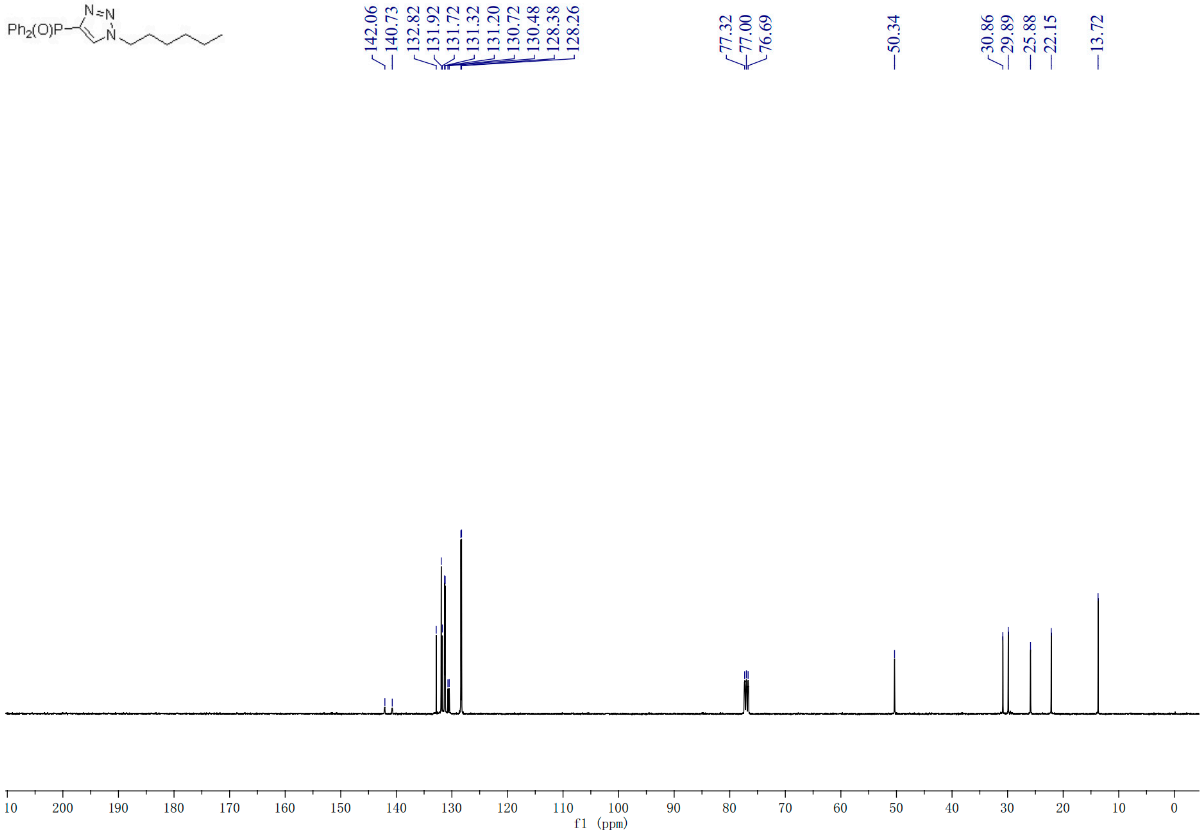

^{31}P NMR (162 MHz, Chloroform-*d*) spectrum of **3p**

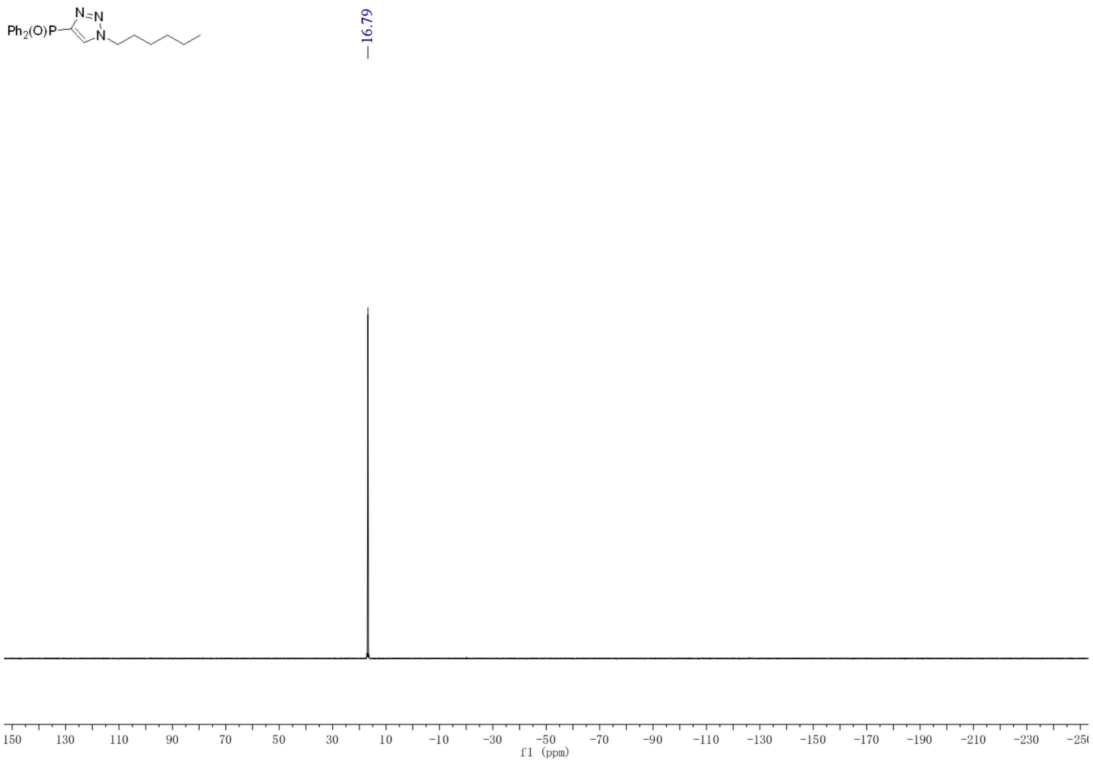

^{1}H-NMR (400 MHz, $CDCl_3$) spectrum of **3q**

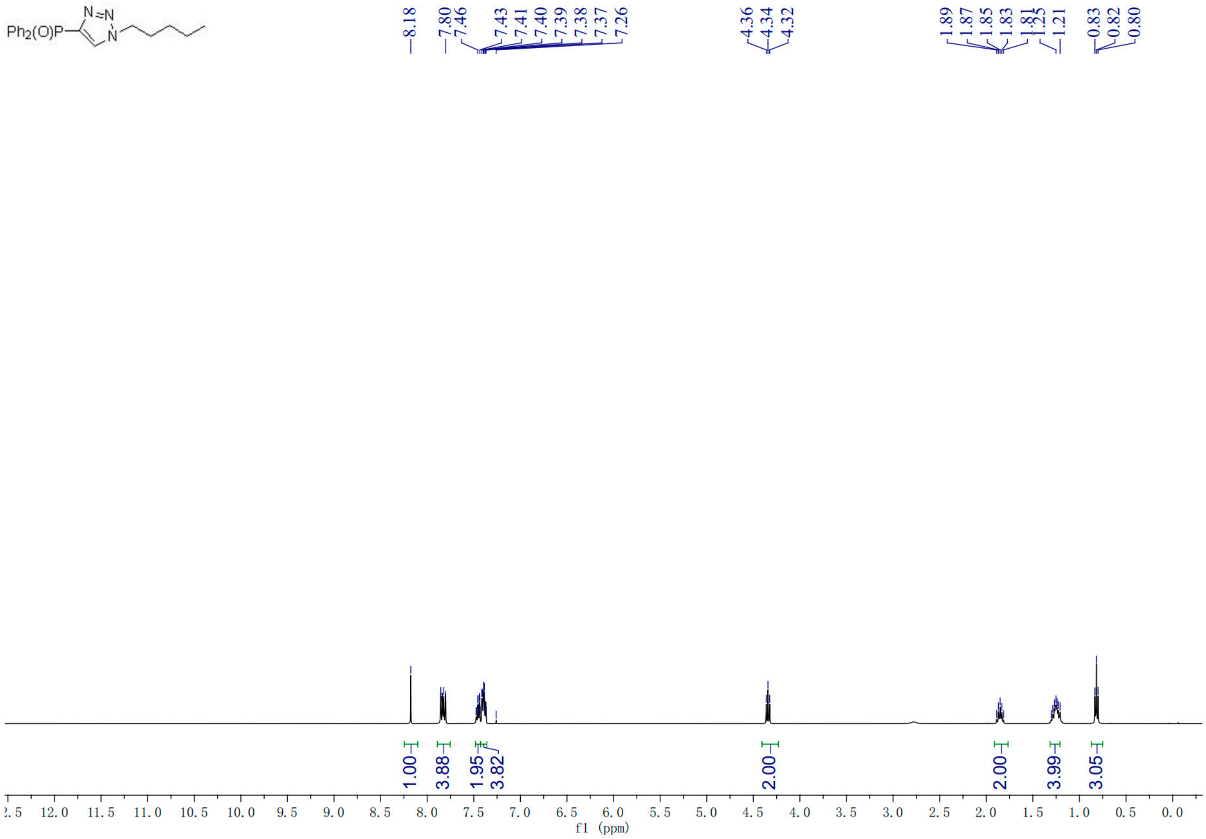

^{13}C-NMR (101 MHz, $CDCl_3$) spectrum of **3q**

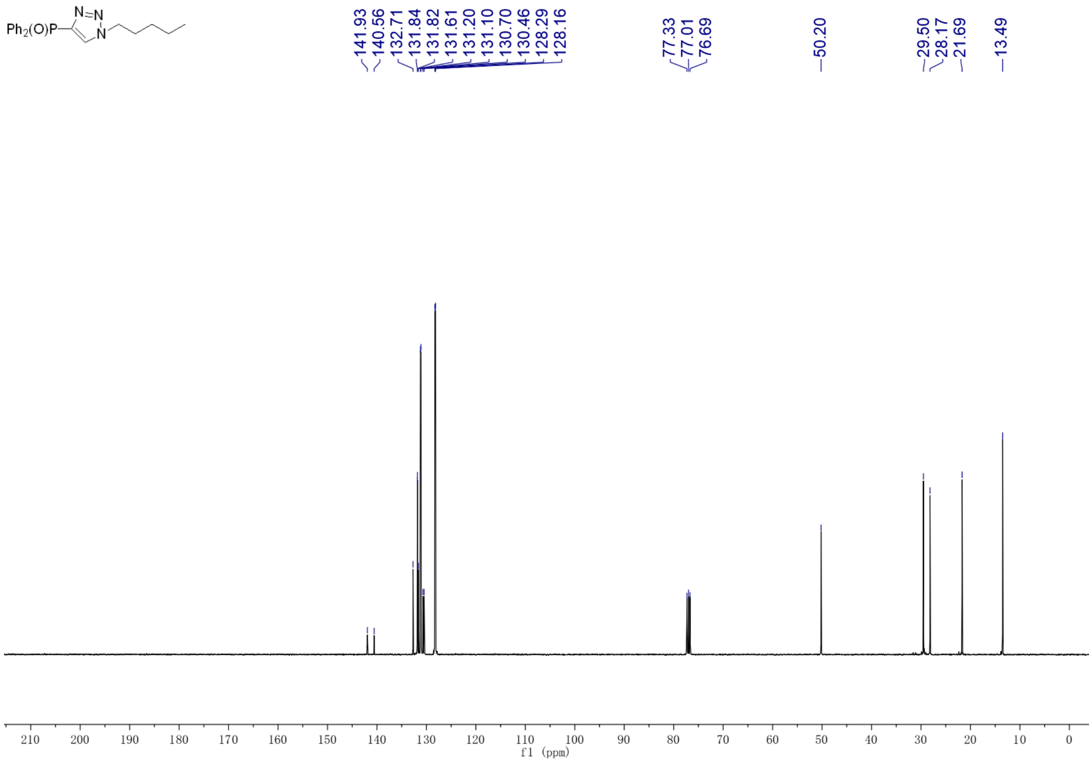

^{31}P NMR (162 MHz, Chloroform-*d*) spectrum of **3q**

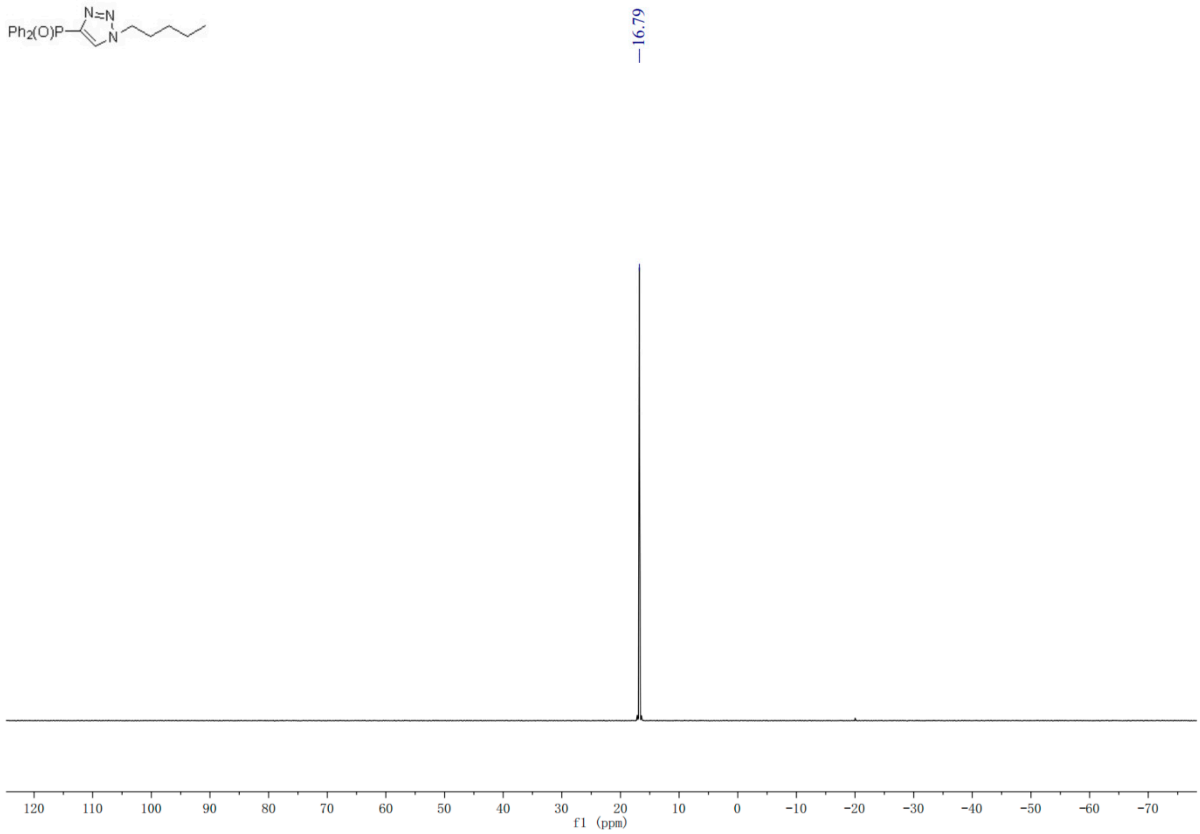

^{1}H-NMR (400 MHz, $CDCl_3$) spectrum of **3r**

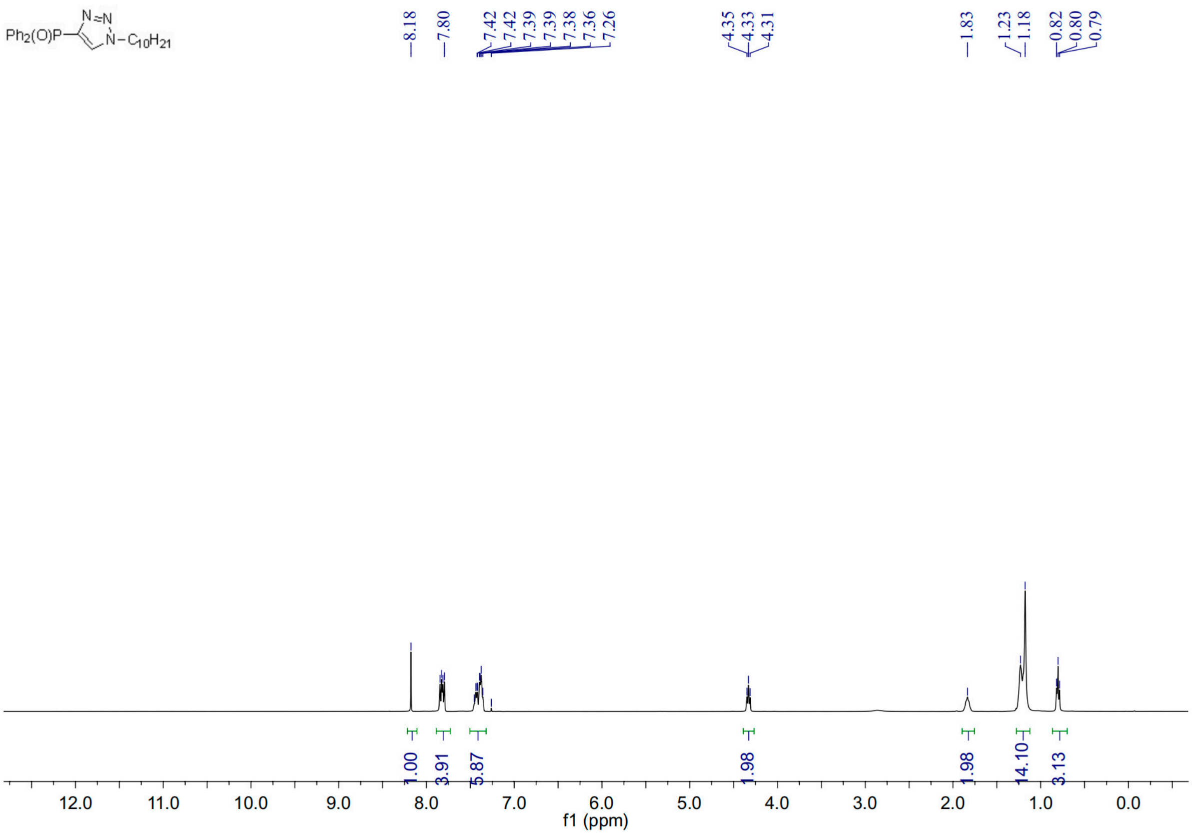

^{13}C-NMR (101 MHz, $CDCl_3$) spectrum of **3r**

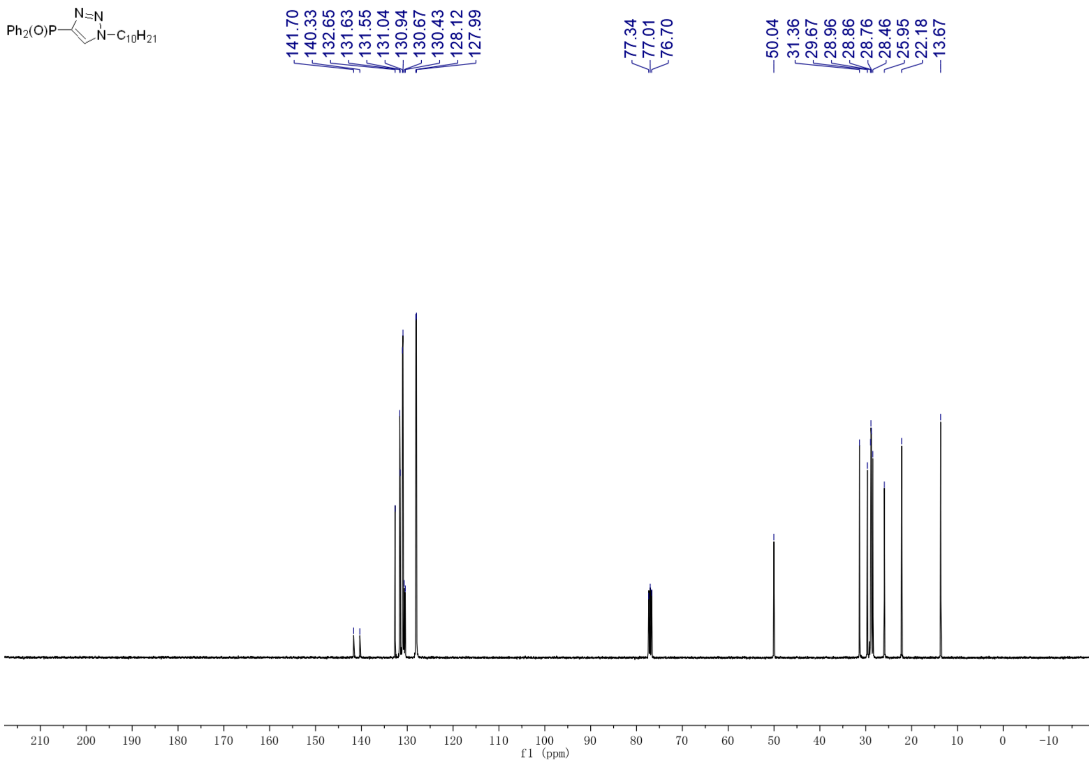

^{31}P NMR (162 MHz, Chloroform-*d*) spectrum of **3r**

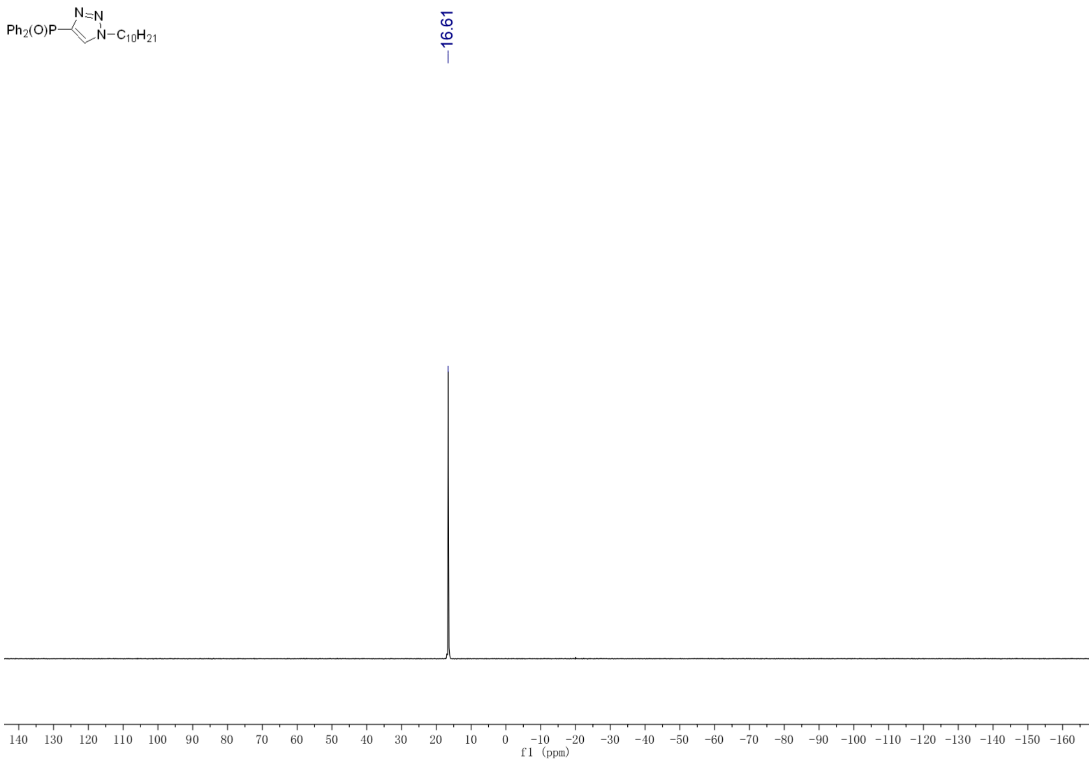

^{1}H-NMR (400 MHz, $CDCl_3$) spectrum of **3s**

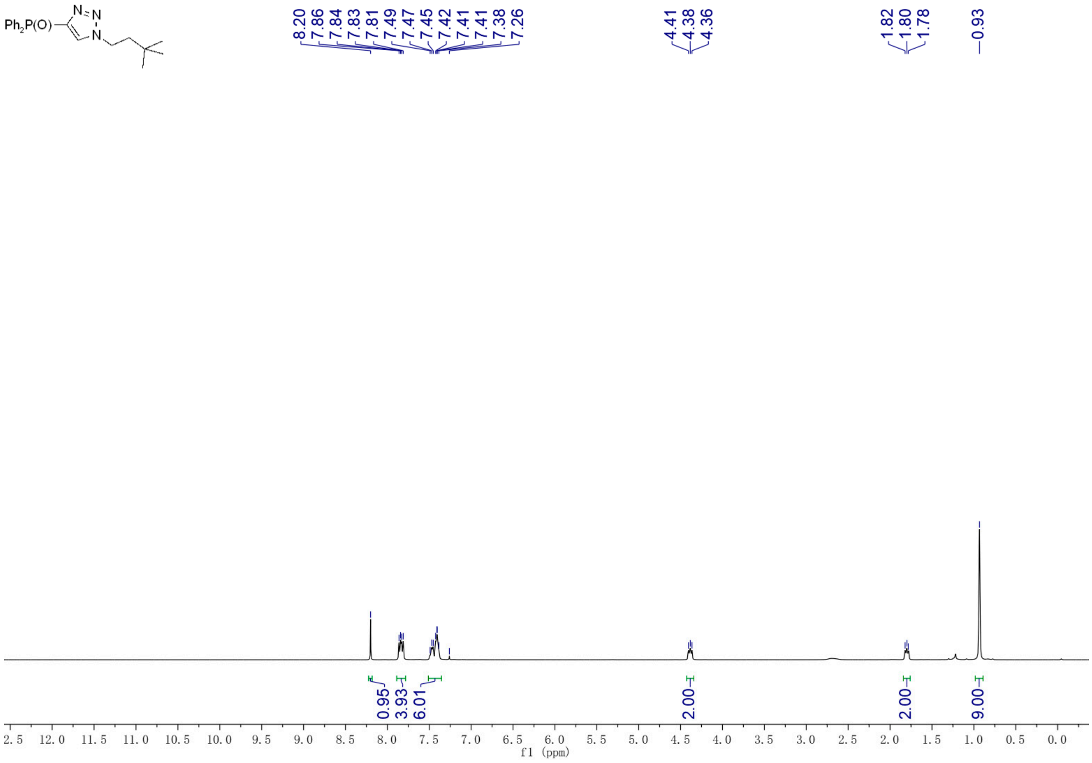

^{13}C-NMR (101 MHz, $CDCl_3$) spectrum of **3s**

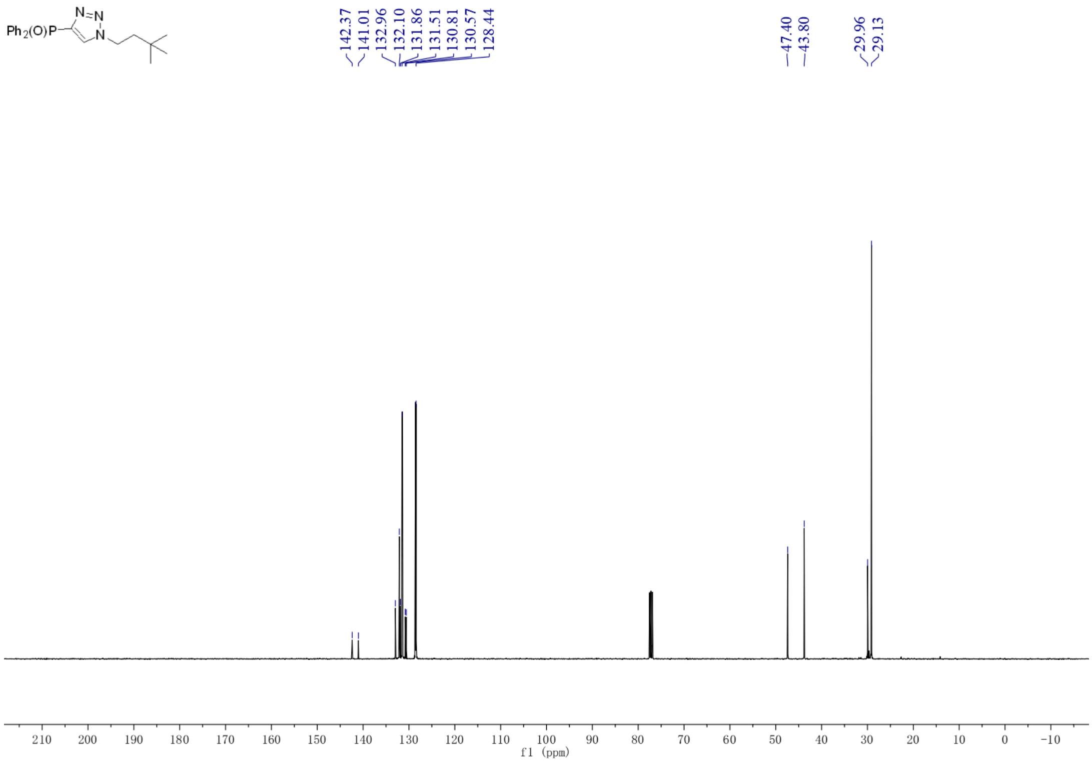

^{31}P NMR (162 MHz, Chloroform-*d*) spectrum of **3s**

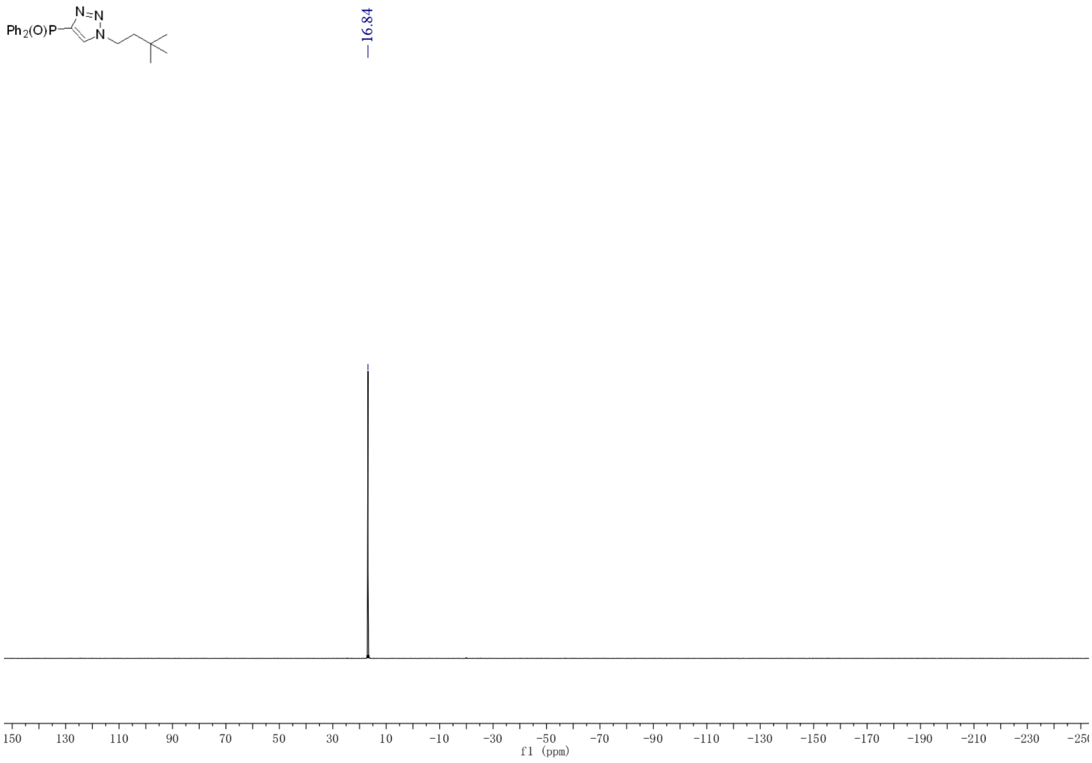

^{1}H-NMR (400 MHz, $CDCl_3$) spectrum of **3t**

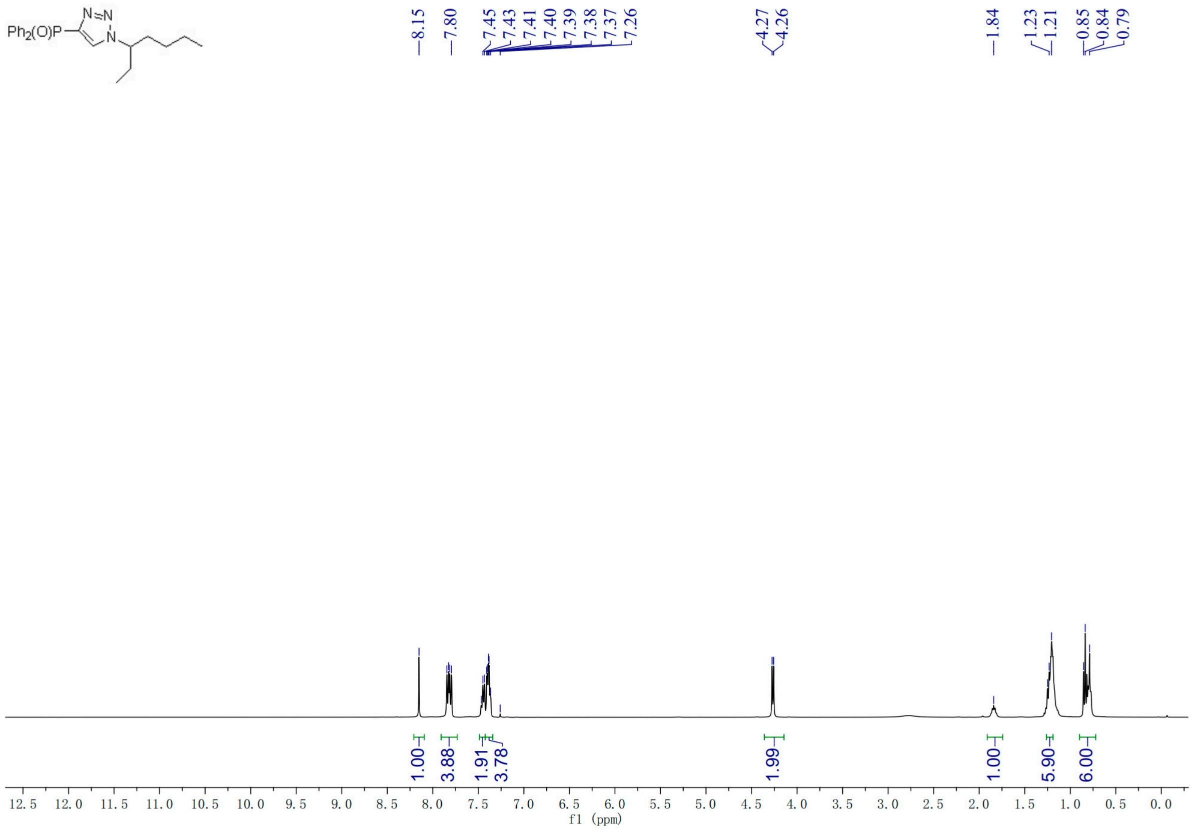

^{13}C-NMR (101 MHz, $CDCl_3$) spectrum of **3t**

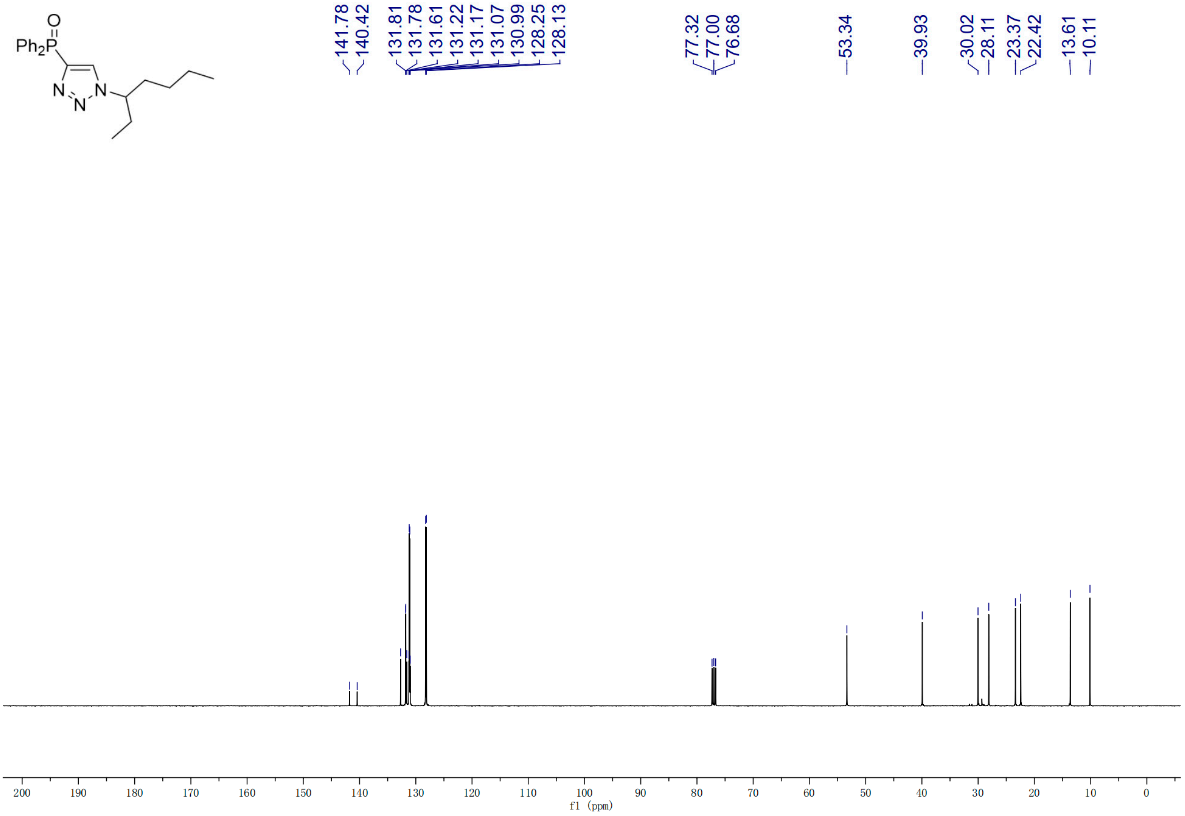

^{31}P NMR (162 MHz, Chloroform-*d*) spectrum of **3t**

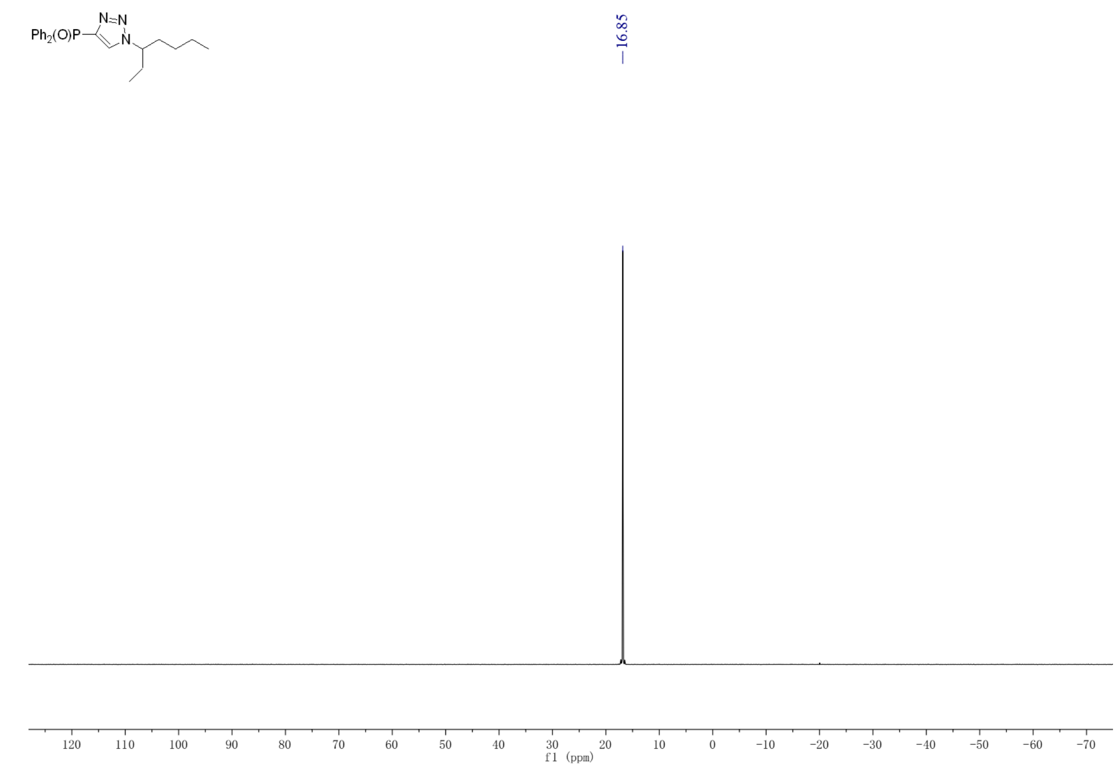

^{1}H-NMR (400 MHz, $CDCl_3$) spectrum of **3u**

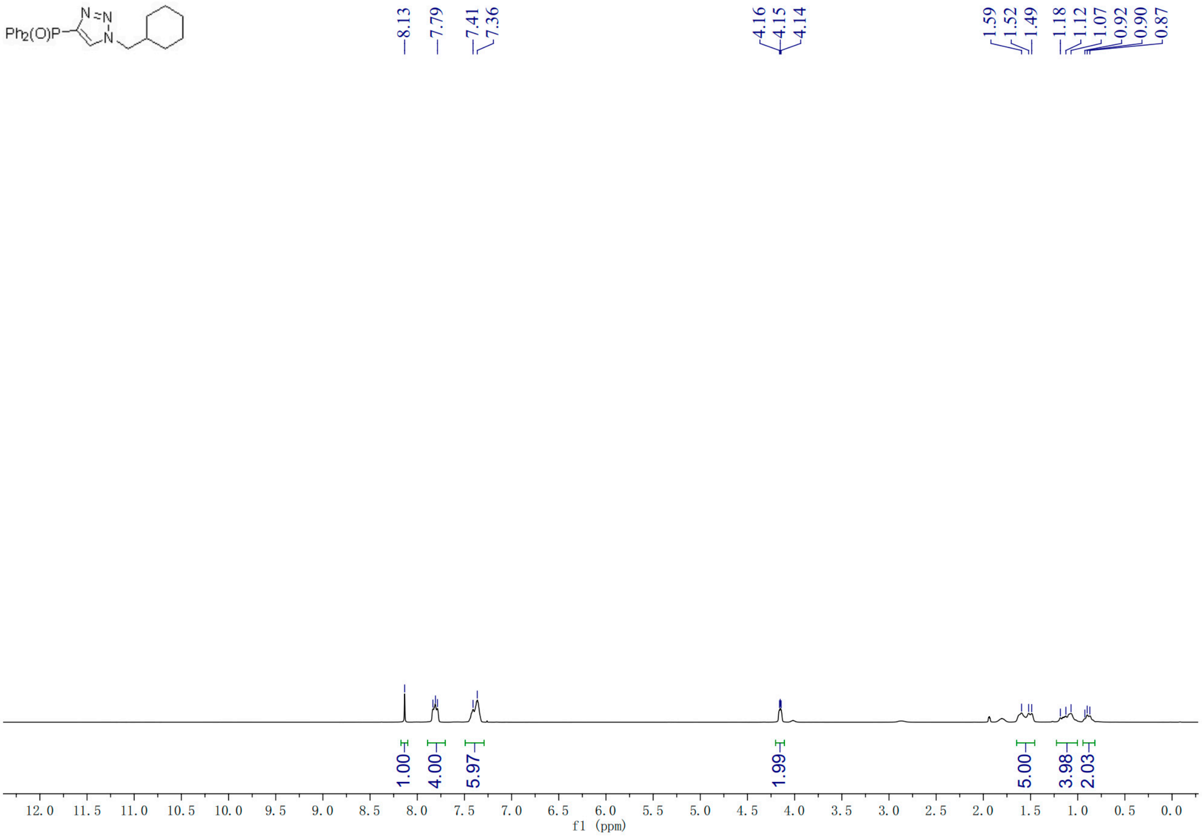

^{13}C-NMR (101 MHz, $CDCl_3$) spectrum of **3u**

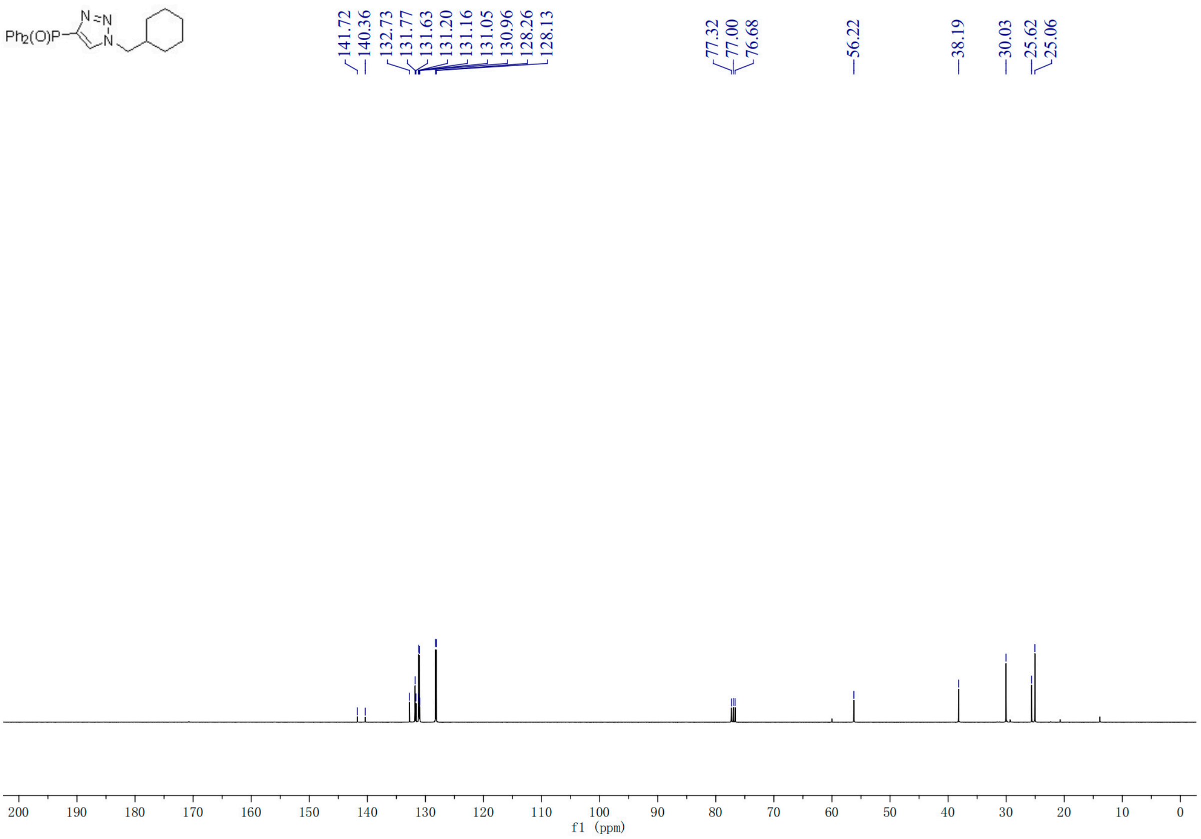

^{31}P NMR (162 MHz, Chloroform-*d*) spectrum of **3u**

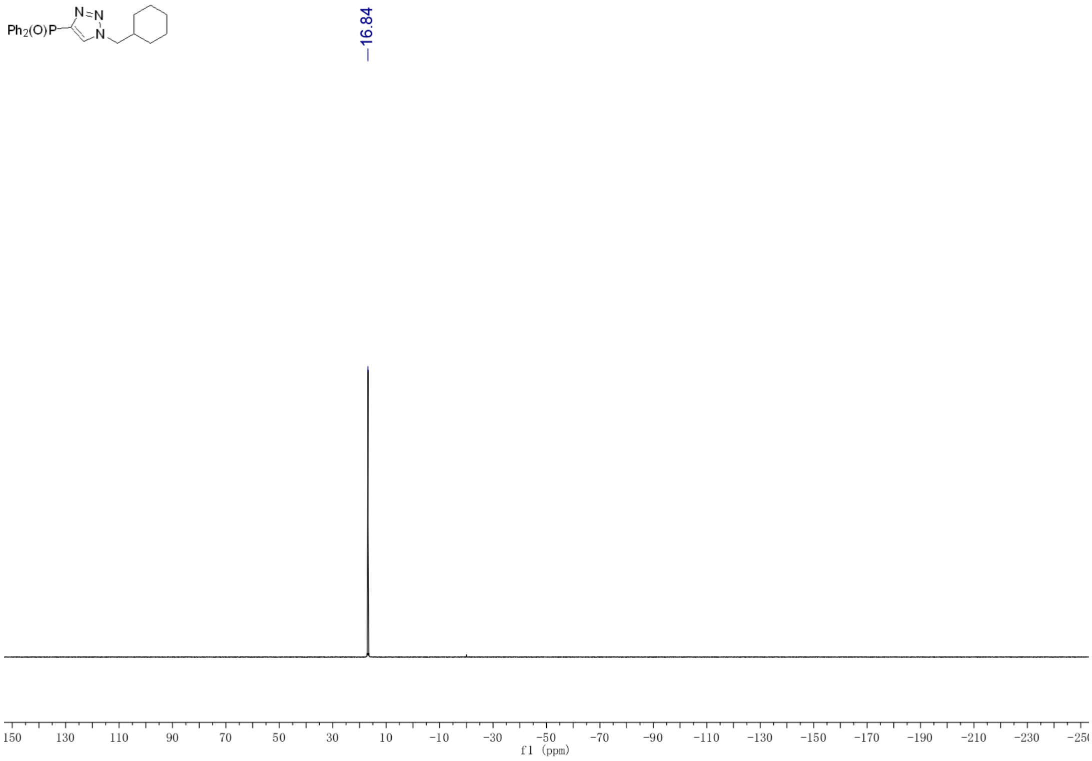

^{1}H-NMR (400 MHz, $CDCl_3$) spectrum of **3v**

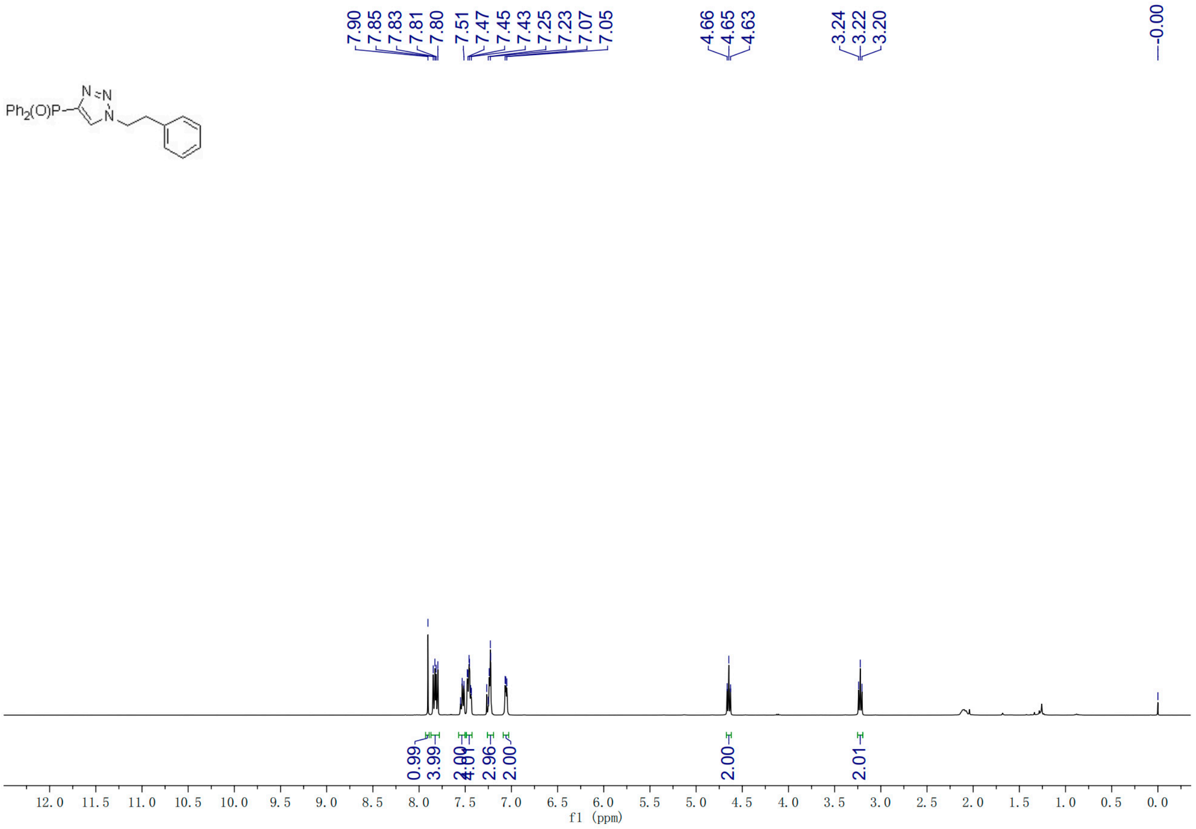

^{13}C-NMR (101 MHz, $CDCl_3$) spectrum of **3v**

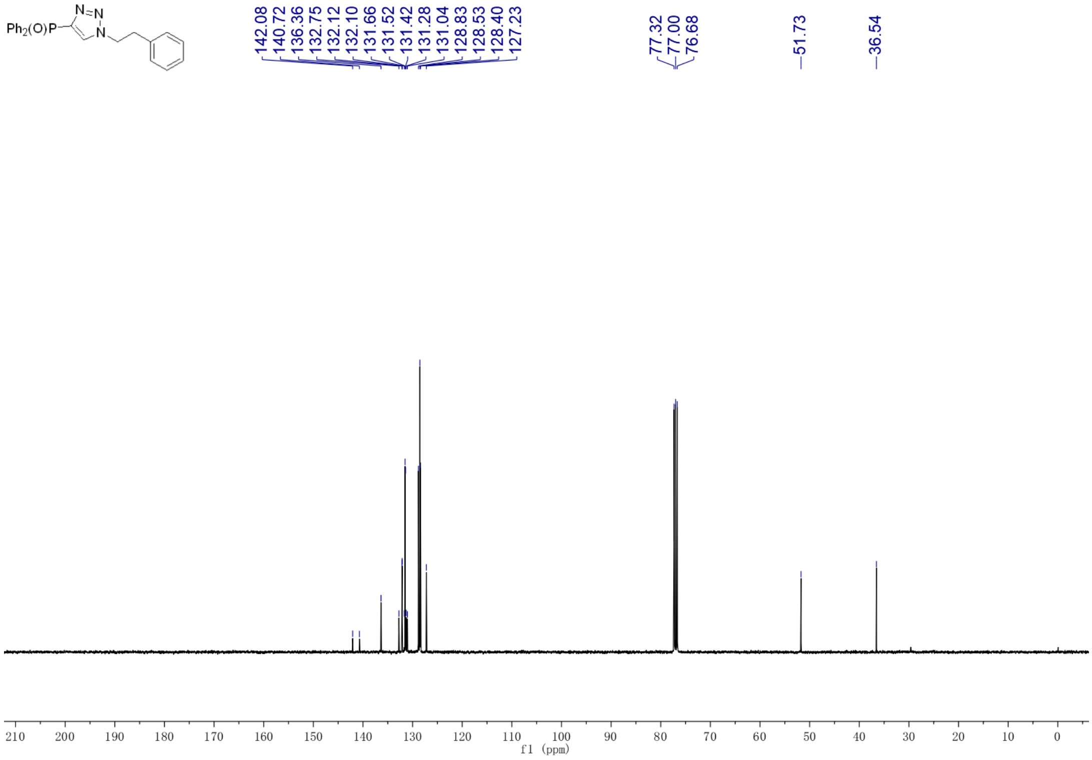

^{31}P NMR (162 MHz, Chloroform-*d*) spectrum of **3v**

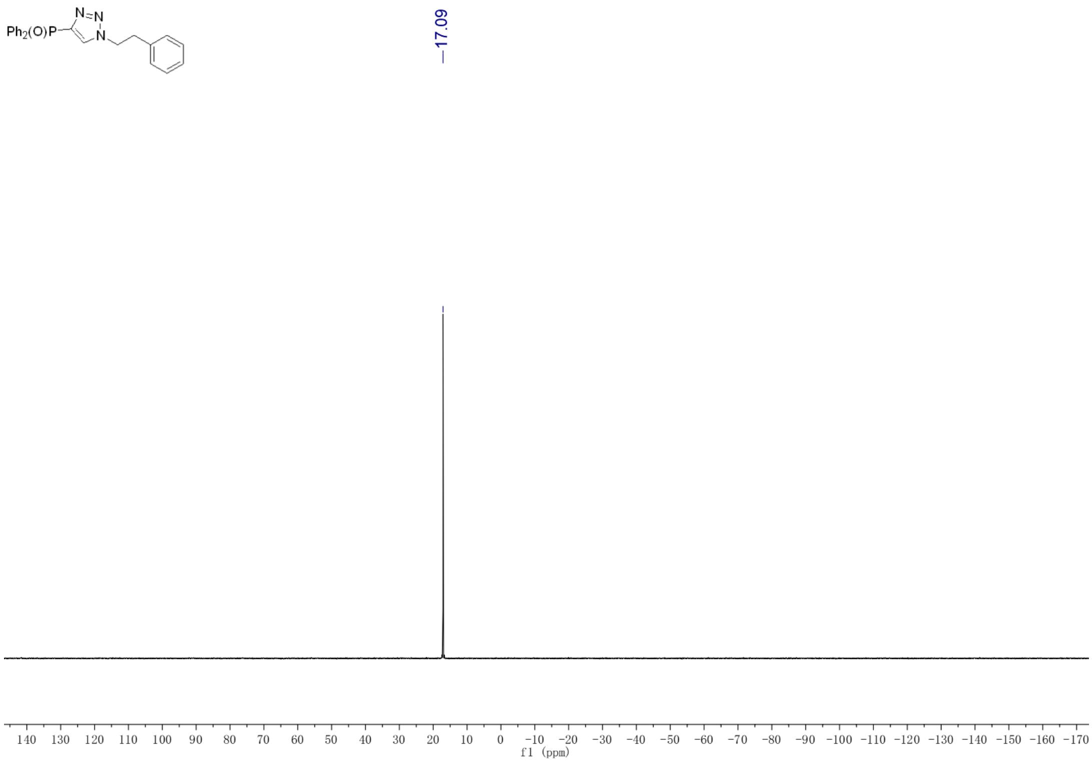

^{1}H-NMR (400 MHz, $CDCl_3$) spectrum of **3w**

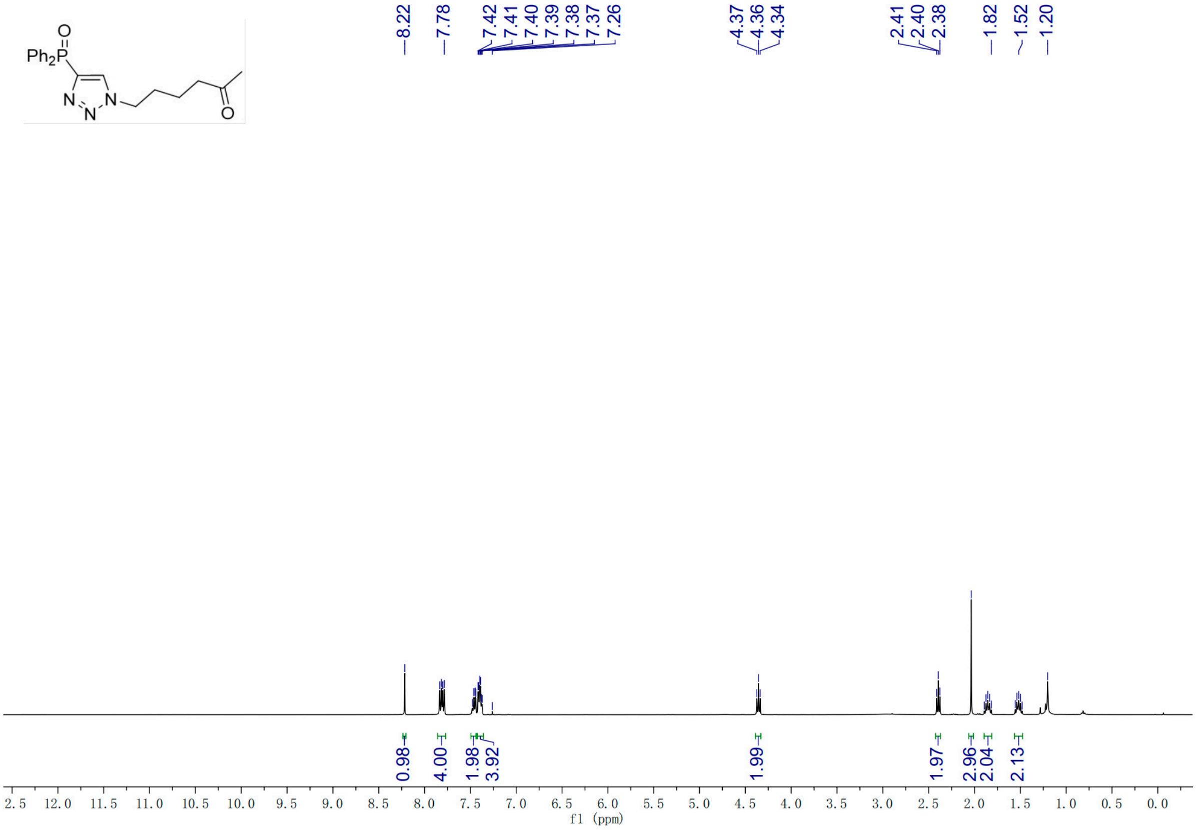

^{13}C-NMR (101 MHz, $CDCl_3$) spectrum of **3w**

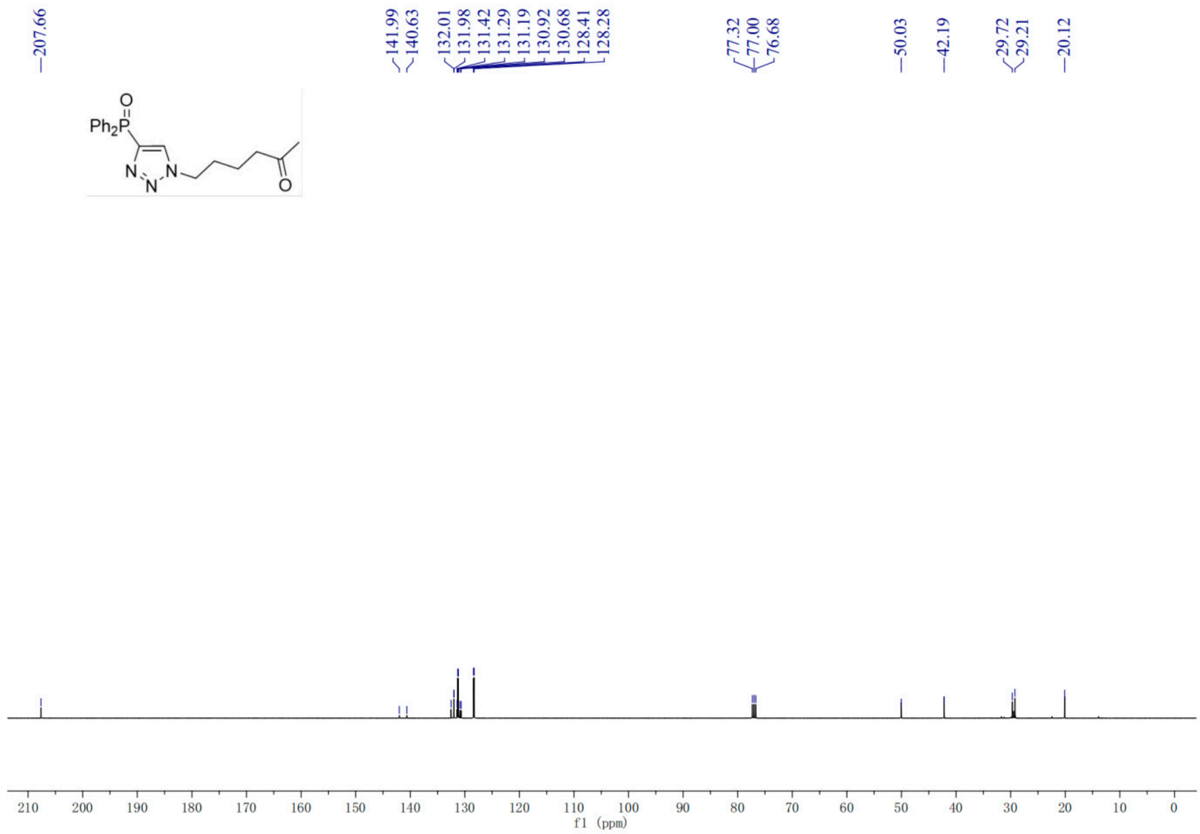

^{31}P NMR (162 MHz, Chloroform-*d*) spectrum of **3w**

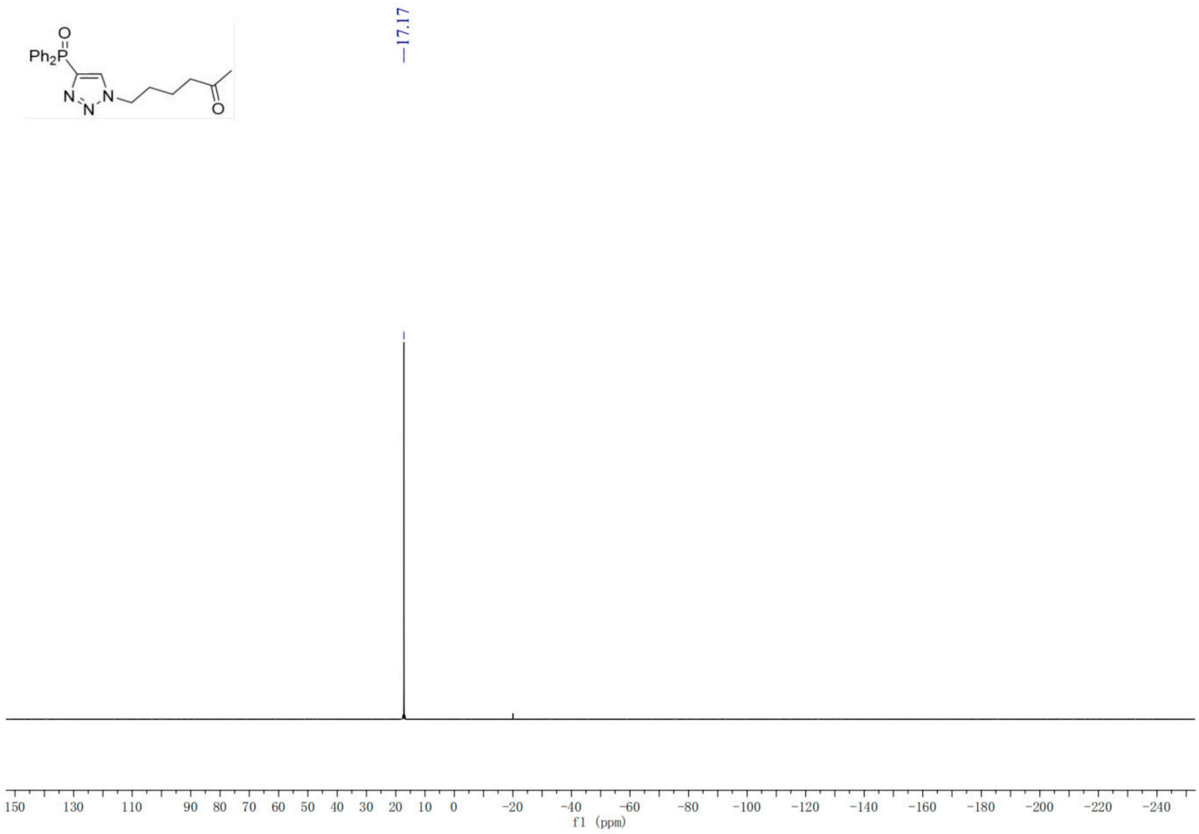

^{1}H-NMR (400 MHz, $CDCl_3$) spectrum of **3x**

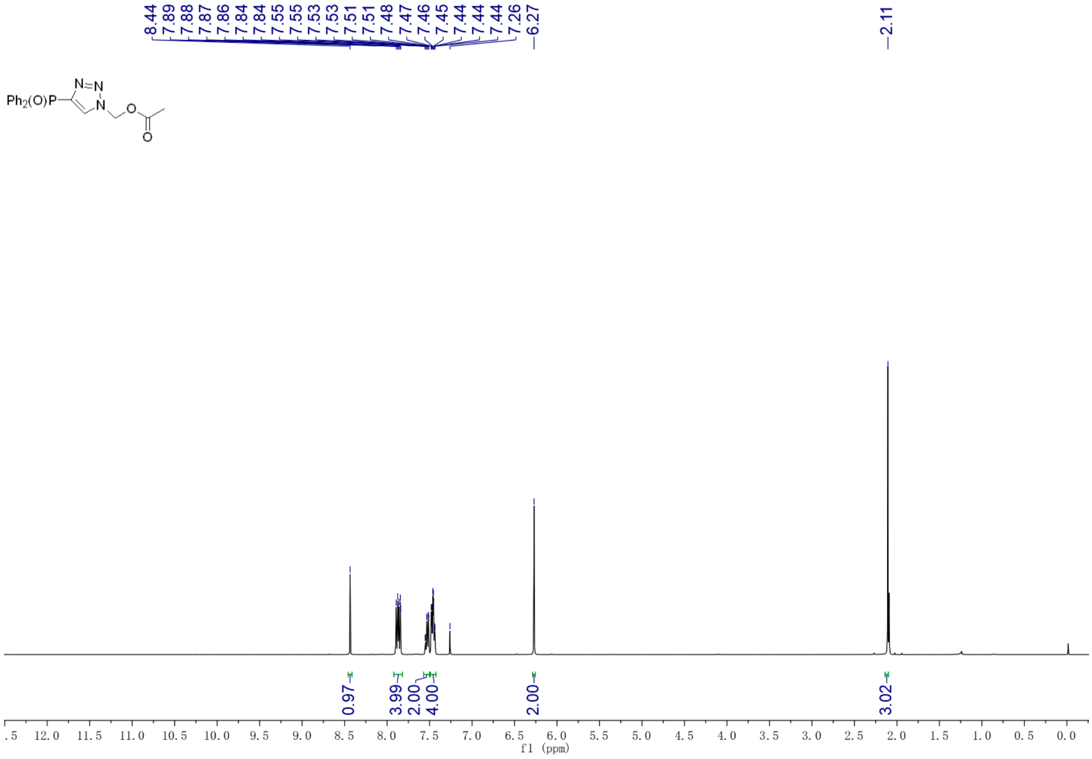

^{13}C-NMR (101 MHz, $CDCl_3$) spectrum of **3x**

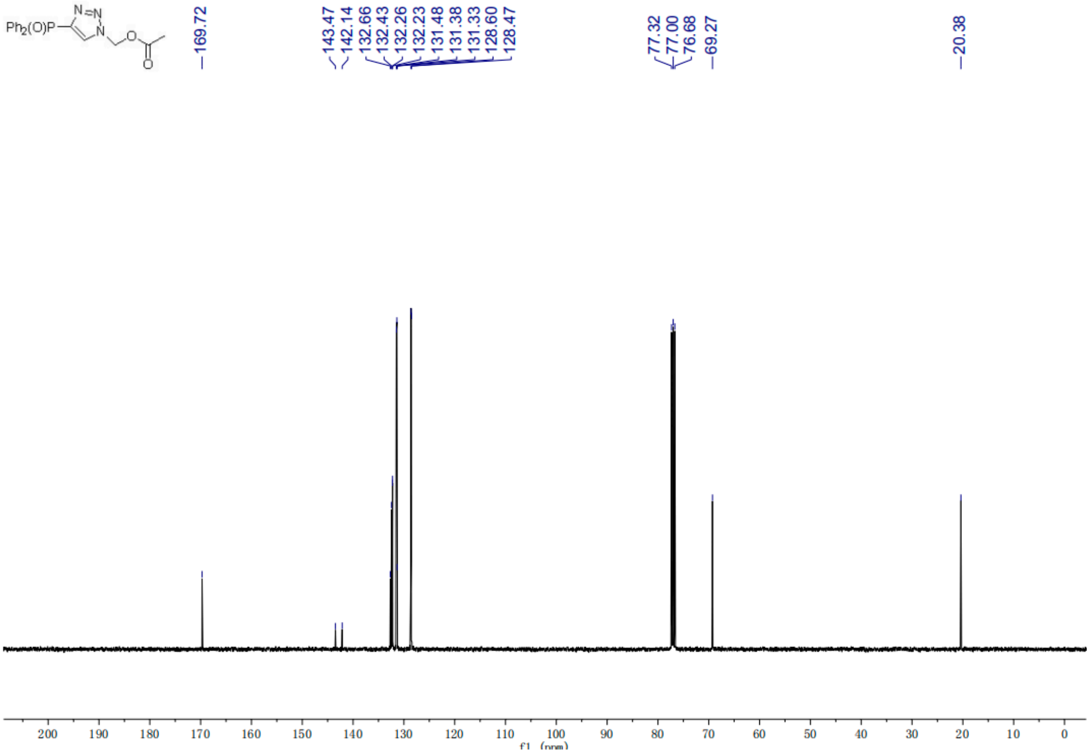

^{31}P NMR (162 MHz, Chloroform-*d*) spectrum of **3x**

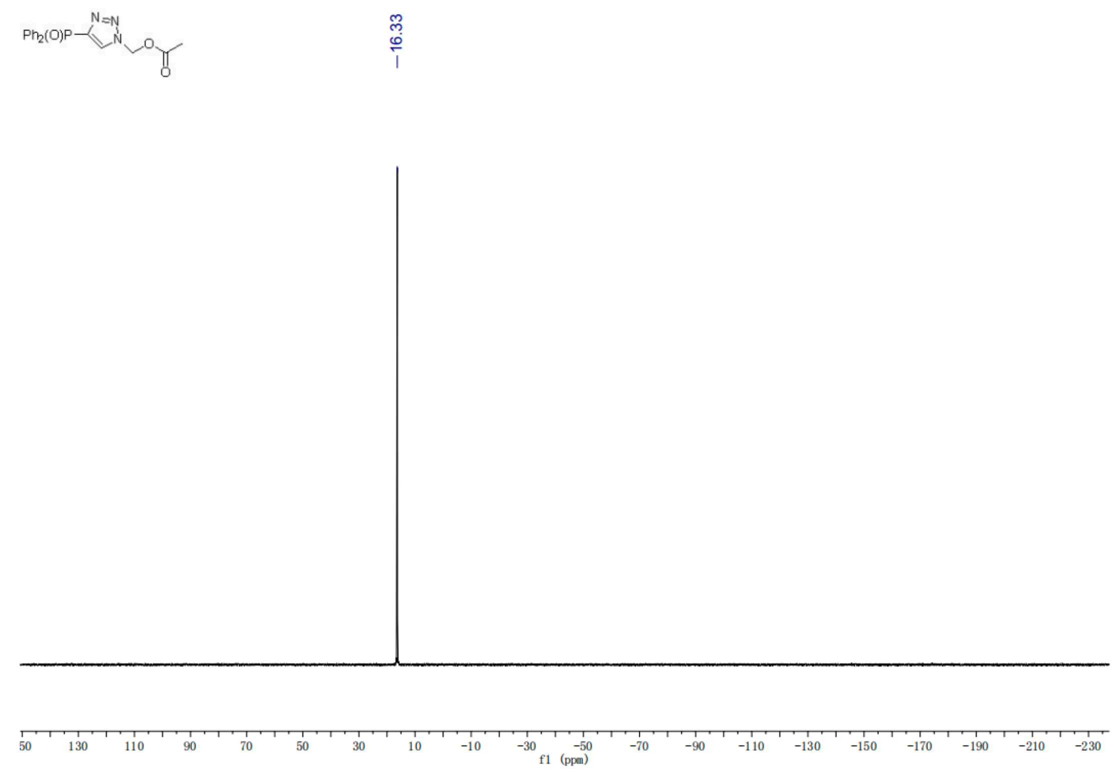

^{1}H-NMR (400 MHz, $CDCl_3$) spectrum of **3y**

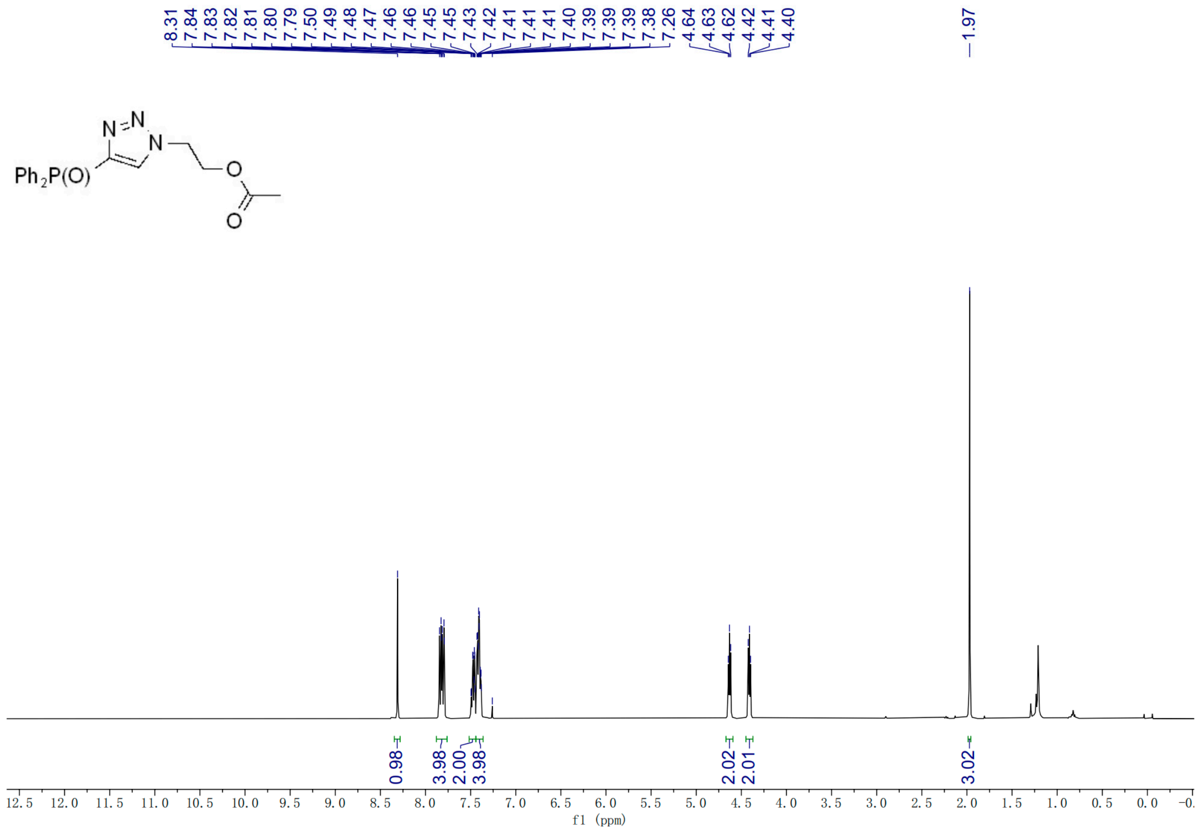

^{13}C-NMR (101 MHz, $CDCl_3$) spectrum of **3y**

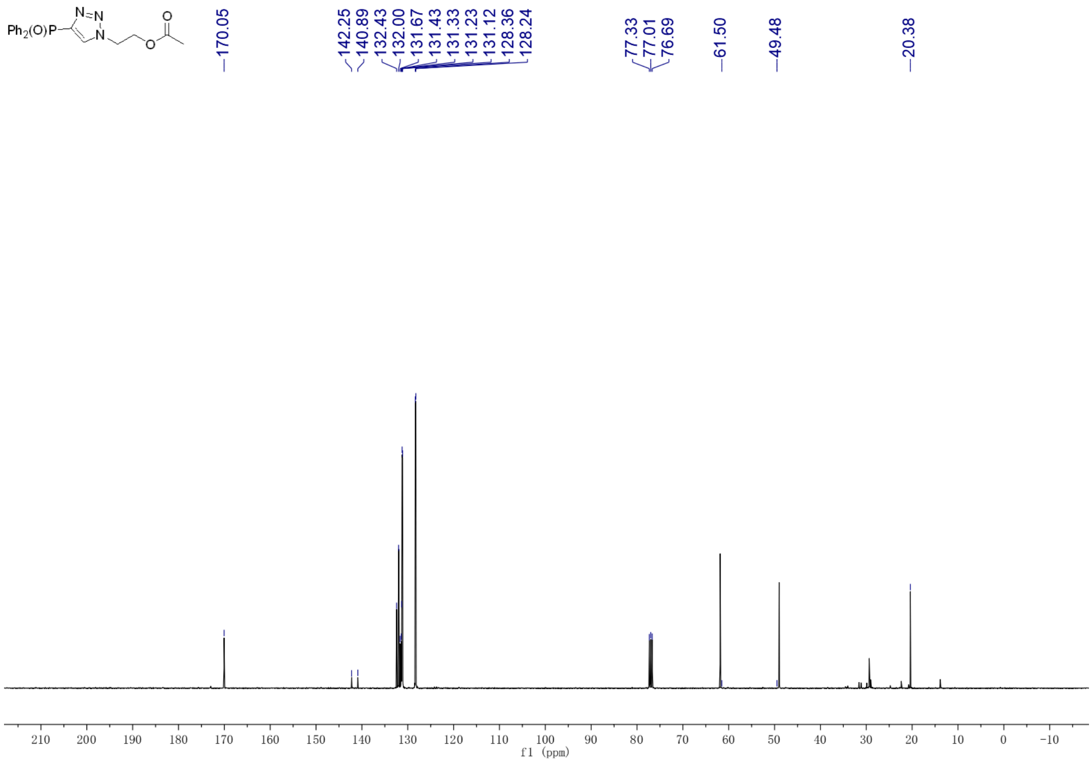

^{31}P NMR (162 MHz, Chloroform-*d*) spectrum of **3y**

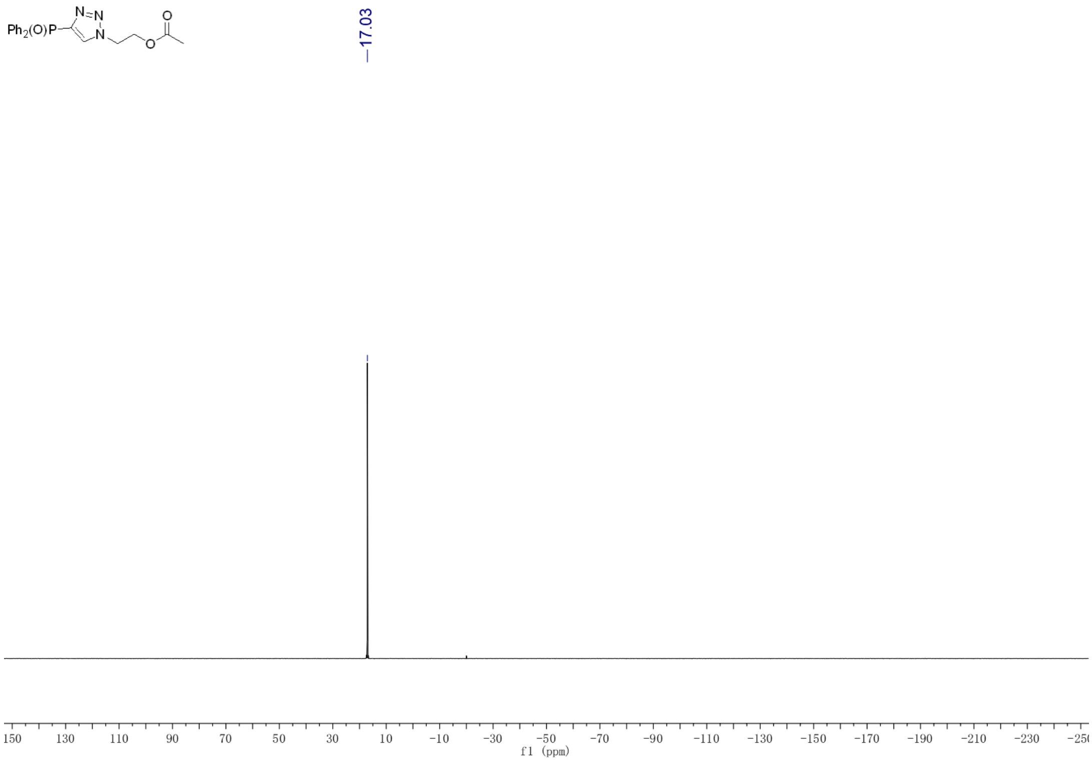

^{1}H-NMR (400 MHz, $CDCl_3$) spectrum of **3z**

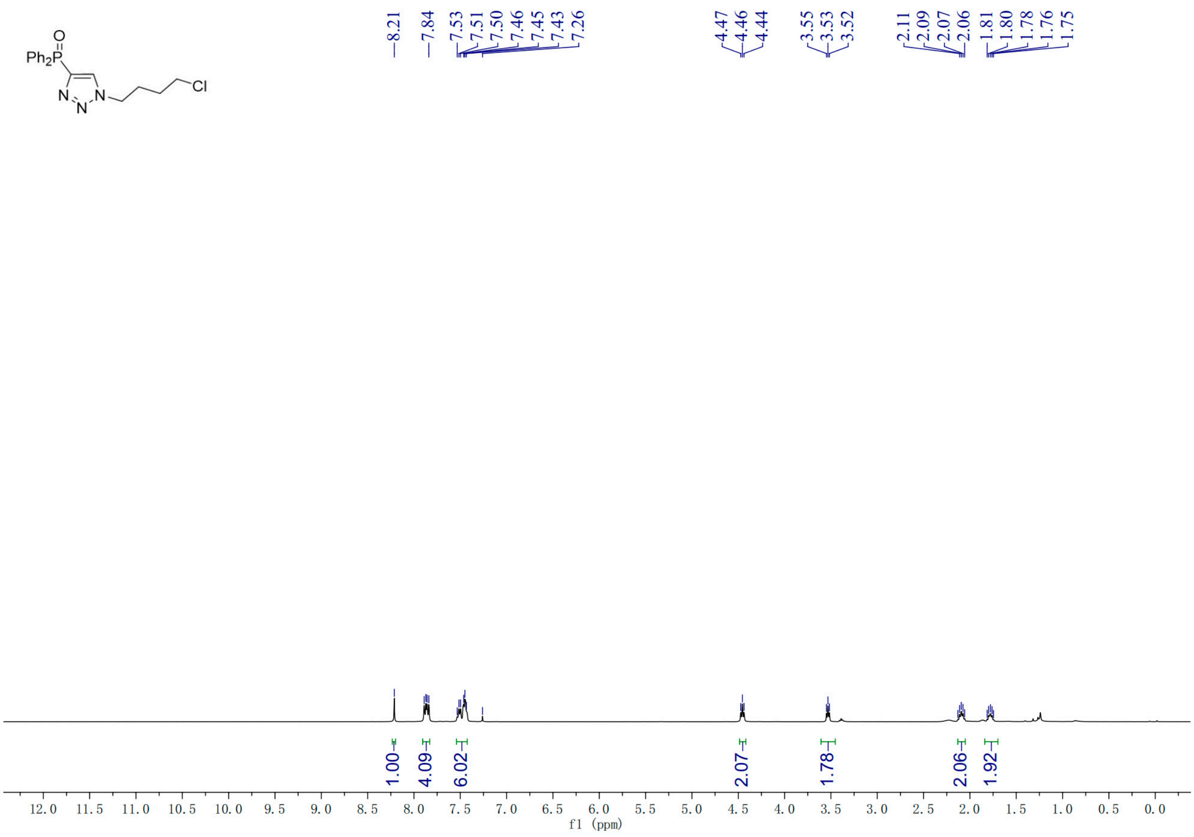

^{13}C-NMR (101 MHz, CDCl$_3$) spectrum of **3z**

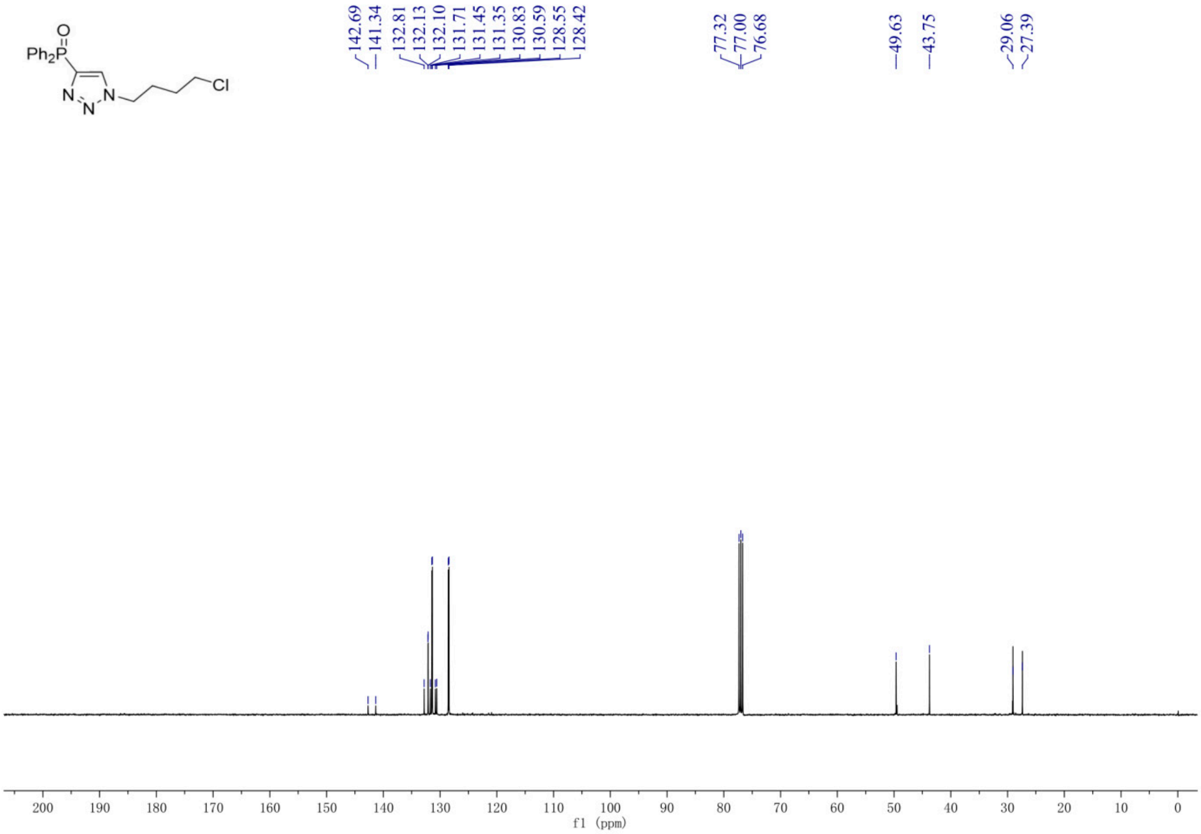

^{31}P NMR (162 MHz, Chloroform-*d*) spectrum of **3z**

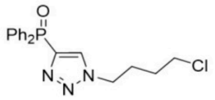

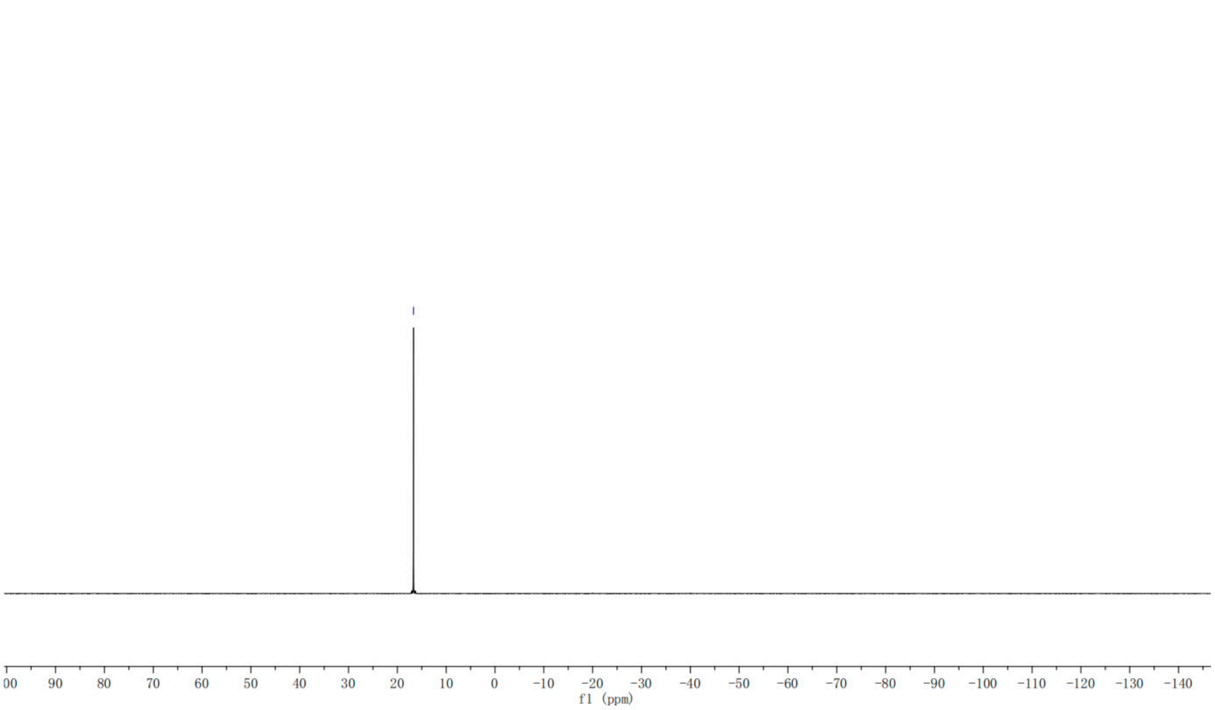

^{1}H-NMR (400 MHz, $CDCl_3$) spectrum of **3aa**

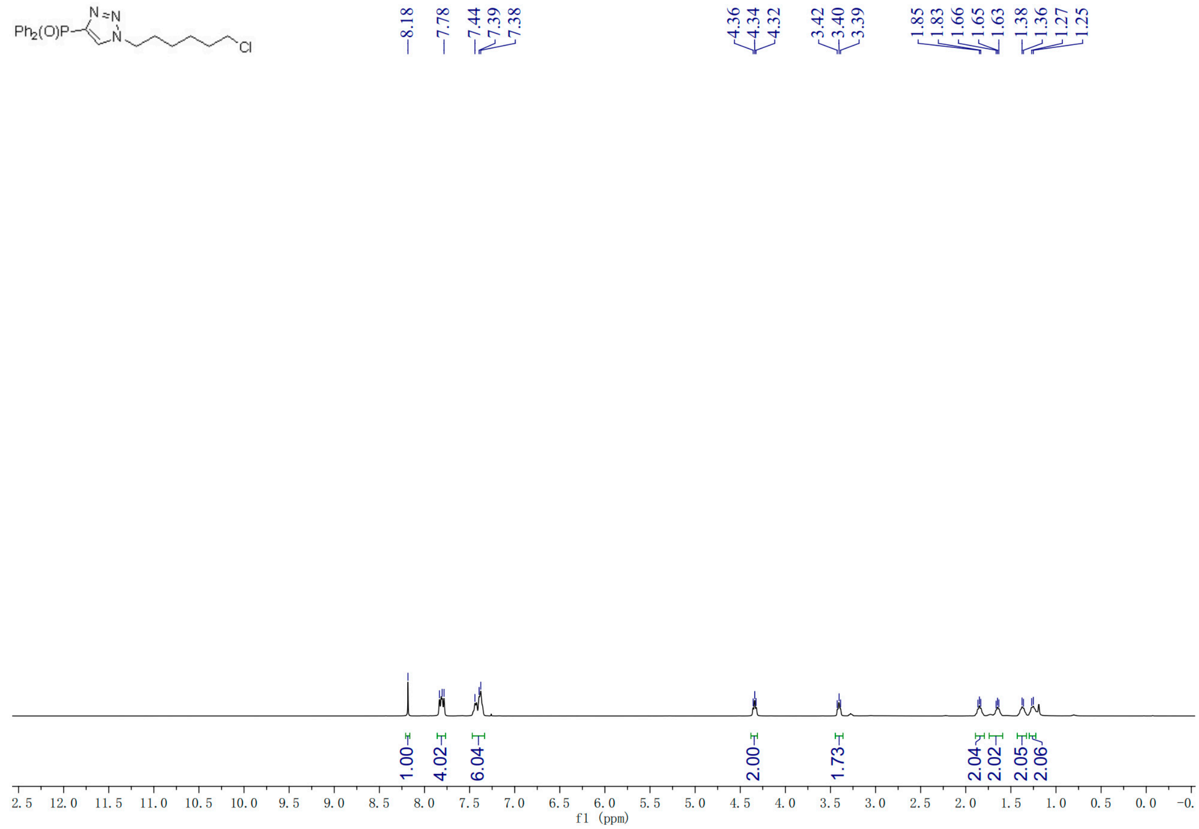

^{13}C-NMR (101 MHz, $CDCl_3$) spectrum of **3aa**

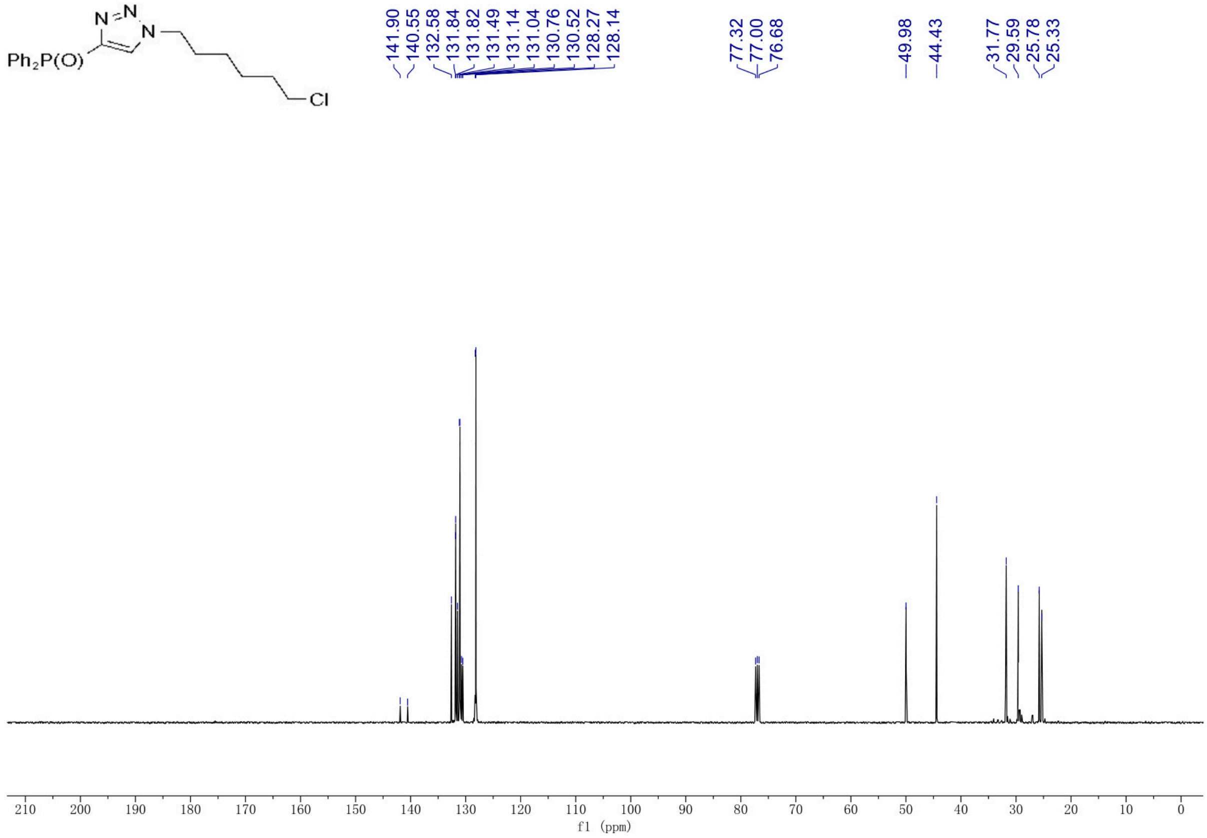

^{31}P NMR (162 MHz, Chloroform-*d*) spectrum of **3aa**

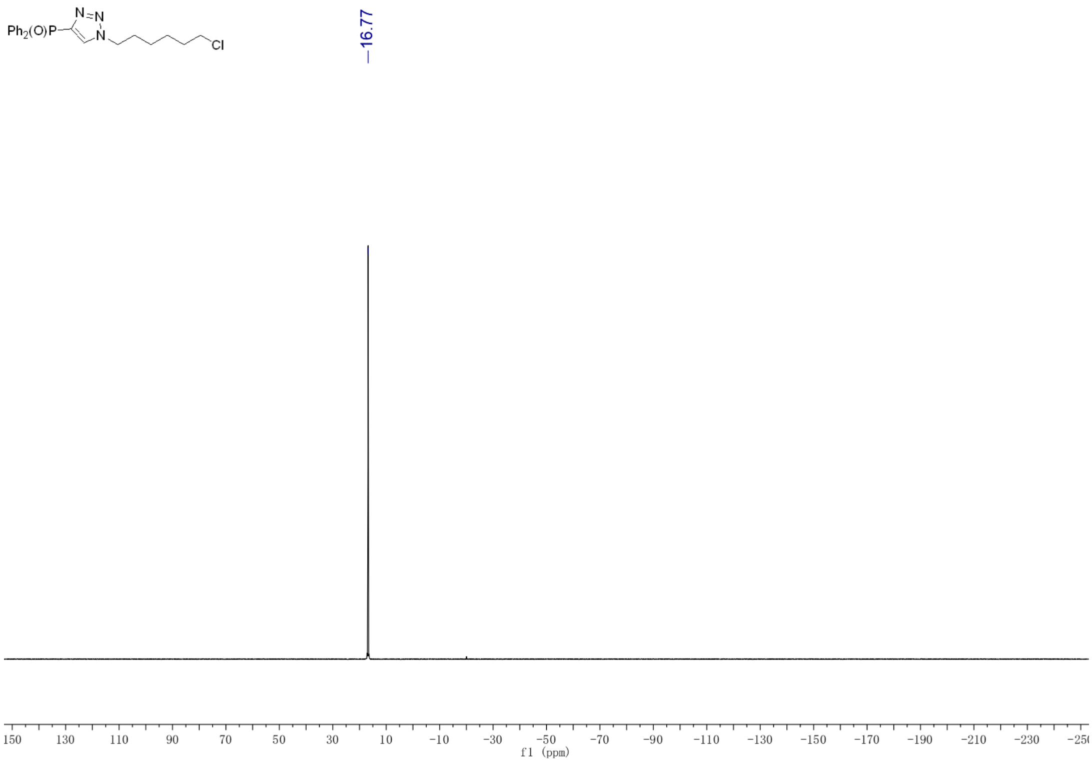

^{1}H-NMR (400 MHz, $CDCl_3$) spectrum of **3ab**

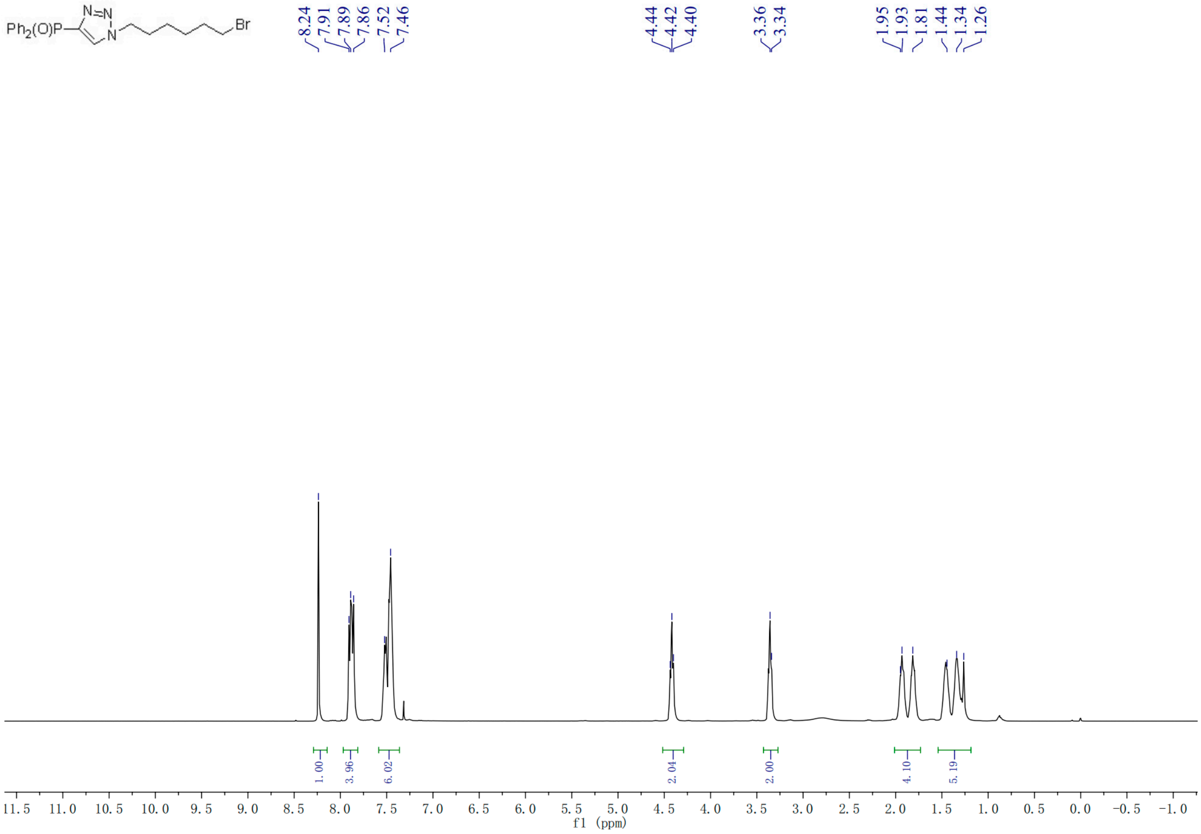

^{13}C-NMR (101 MHz, $CDCl_3$) spectrum of **3ab**

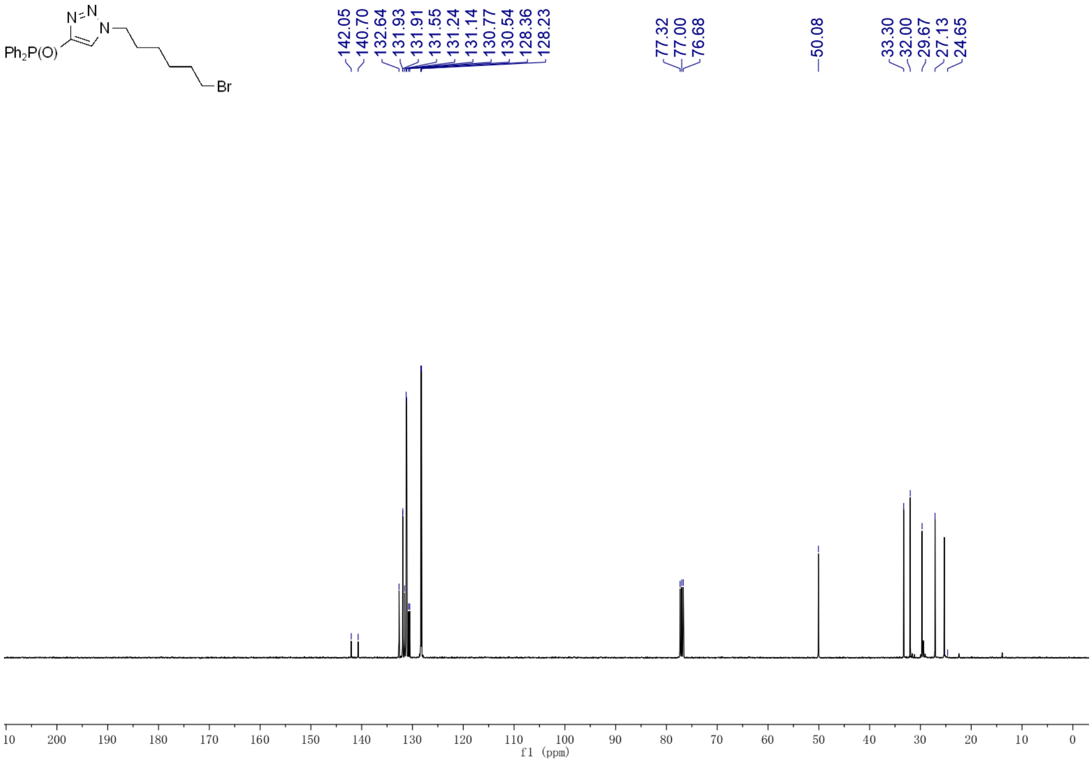

^{31}P NMR (162 MHz, Chloroform-*d*) spectrum of **3ab**

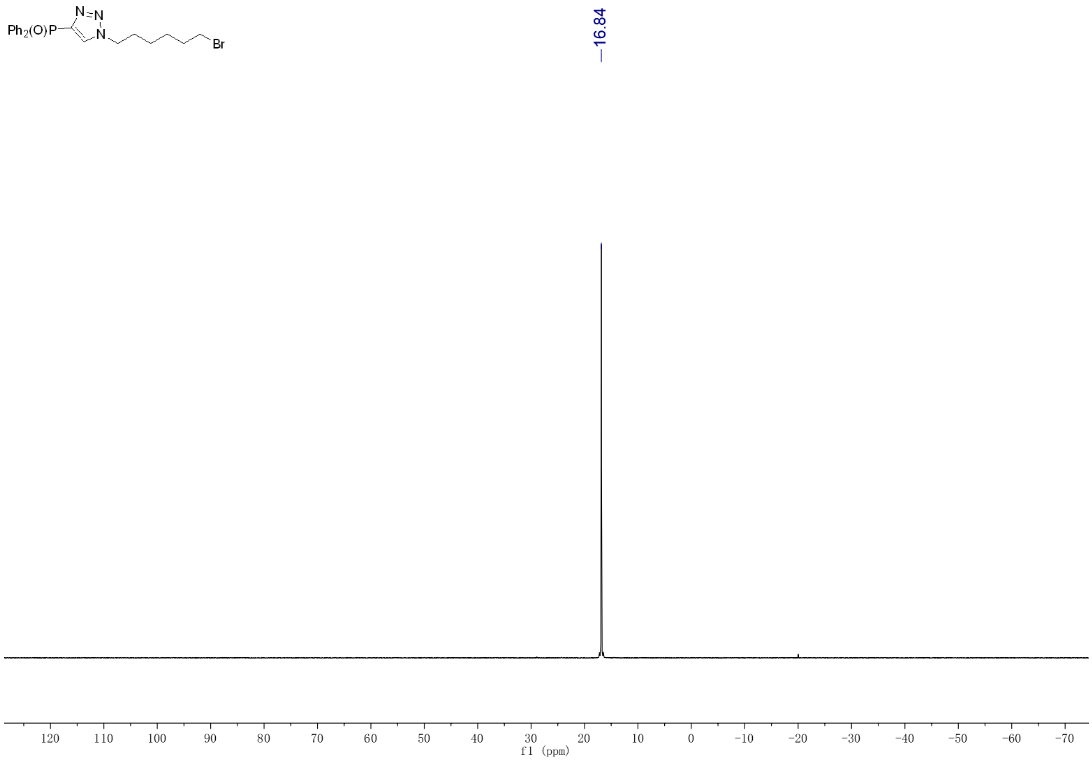

^{1}H-NMR (400 MHz, $CDCl_3$) spectrum of **3ac**

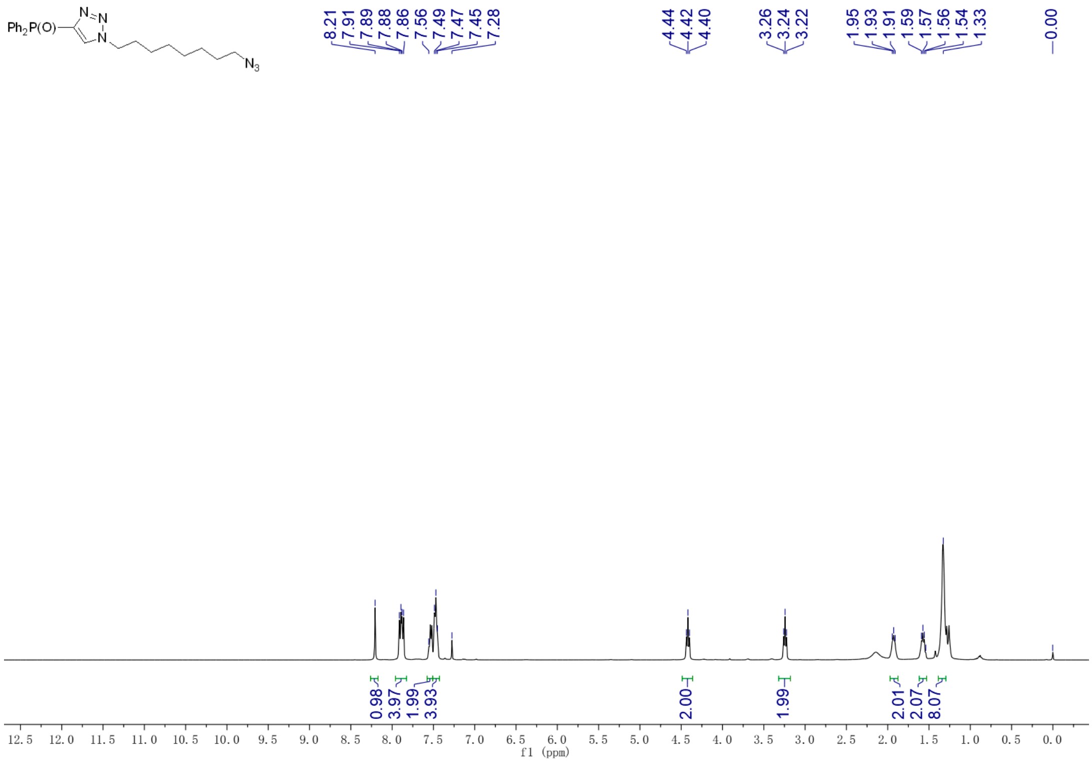

^{13}C-NMR (101 MHz, $CDCl_3$) spectrum of **3ac**

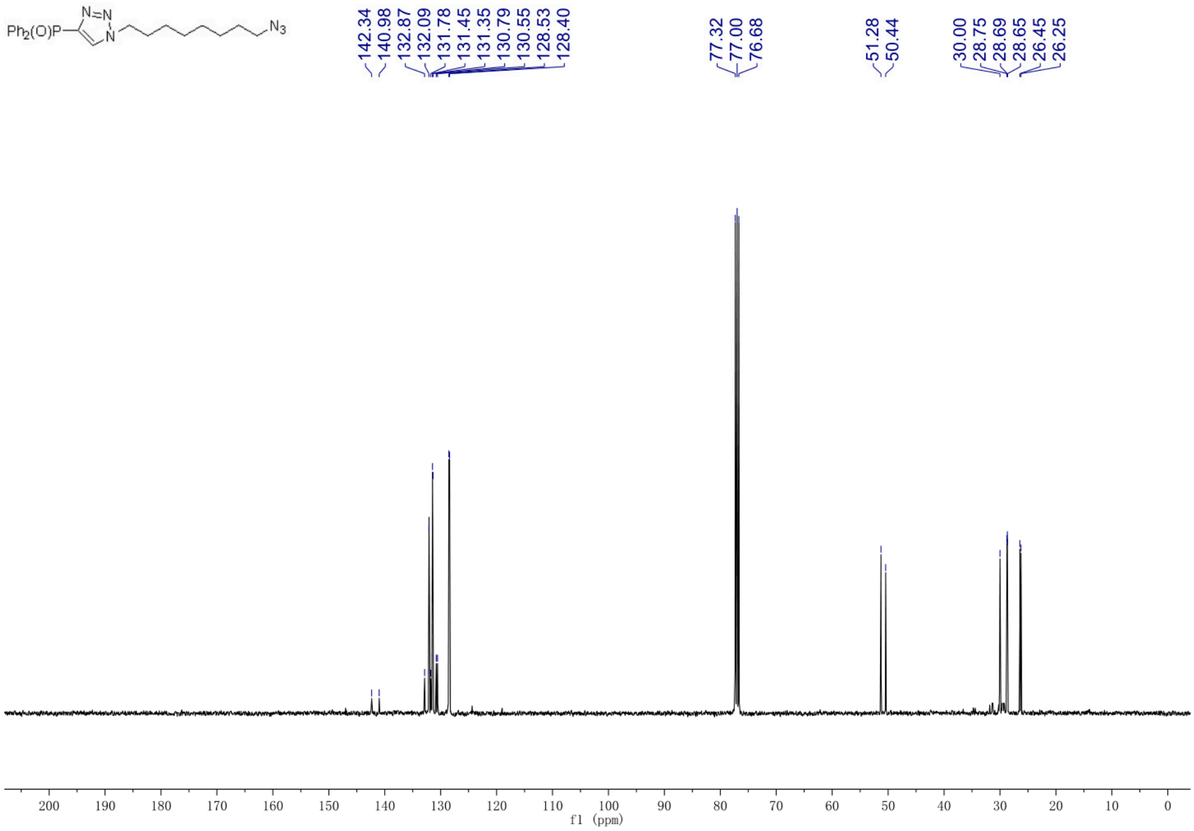

^{31}P NMR (162 MHz, Chloroform-*d*) spectrum of **3ac**

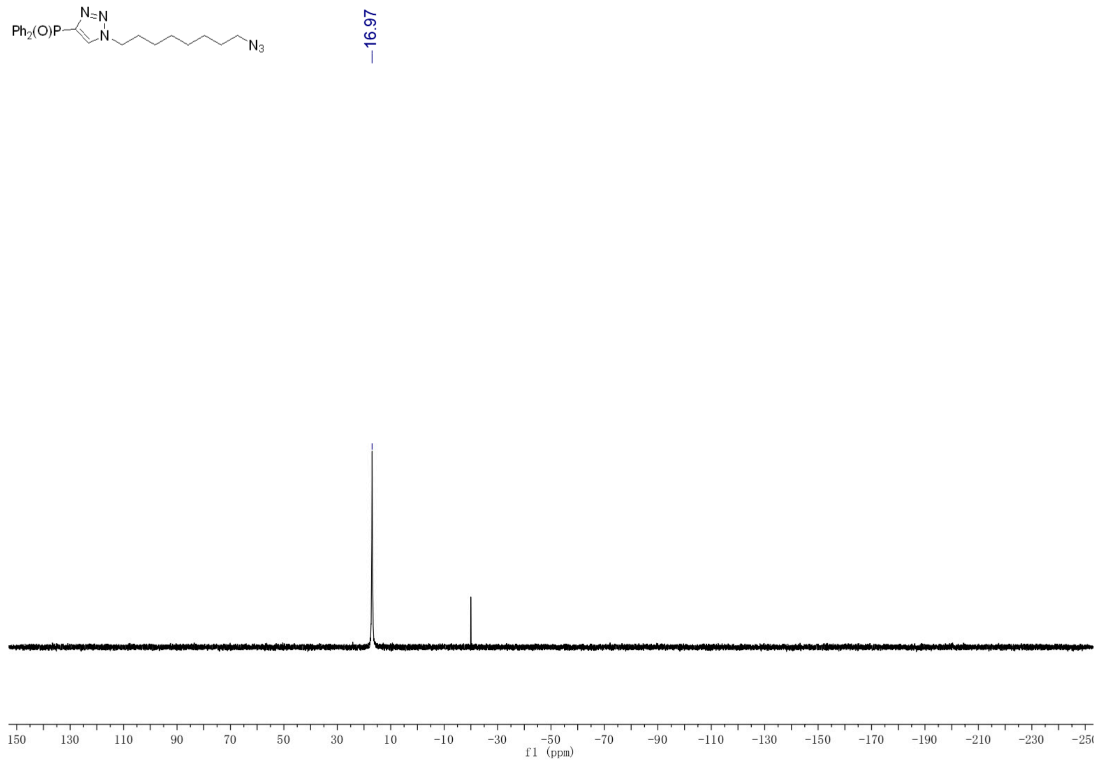

^{1}H-NMR (400 MHz, CDCl$_3$) spectrum of **3ad**

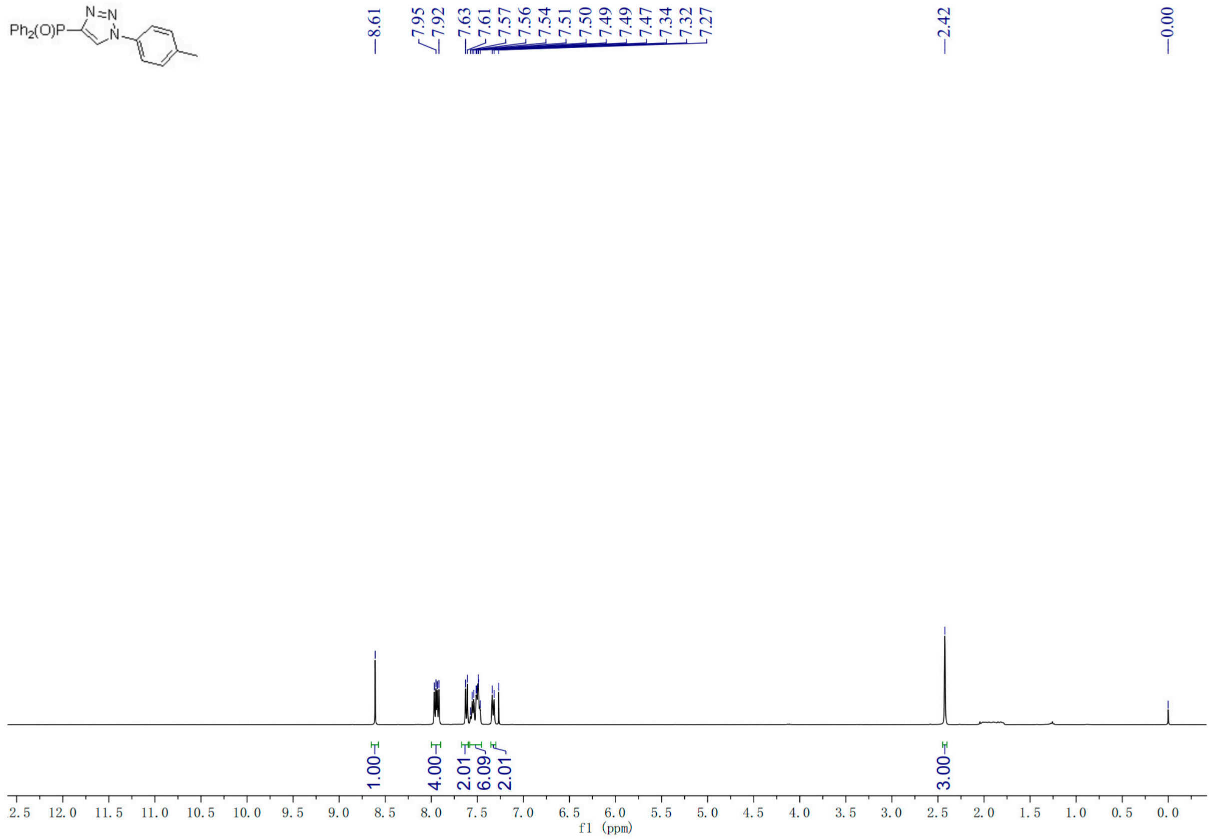

^{13}C-NMR (101 MHz, $CDCl_3$) spectrum of **3ad**

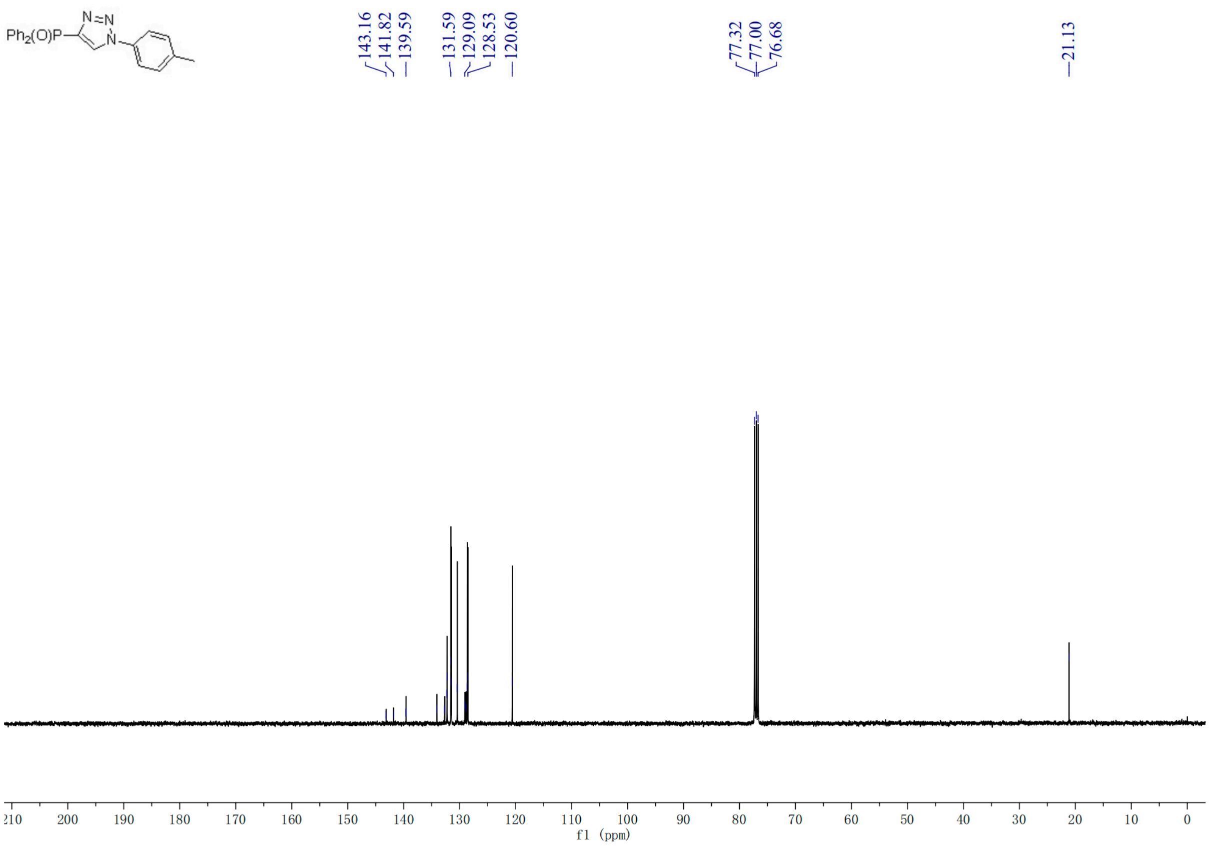

^{31}P NMR (162 MHz, Chloroform-*d*) spectrum of **3ad**

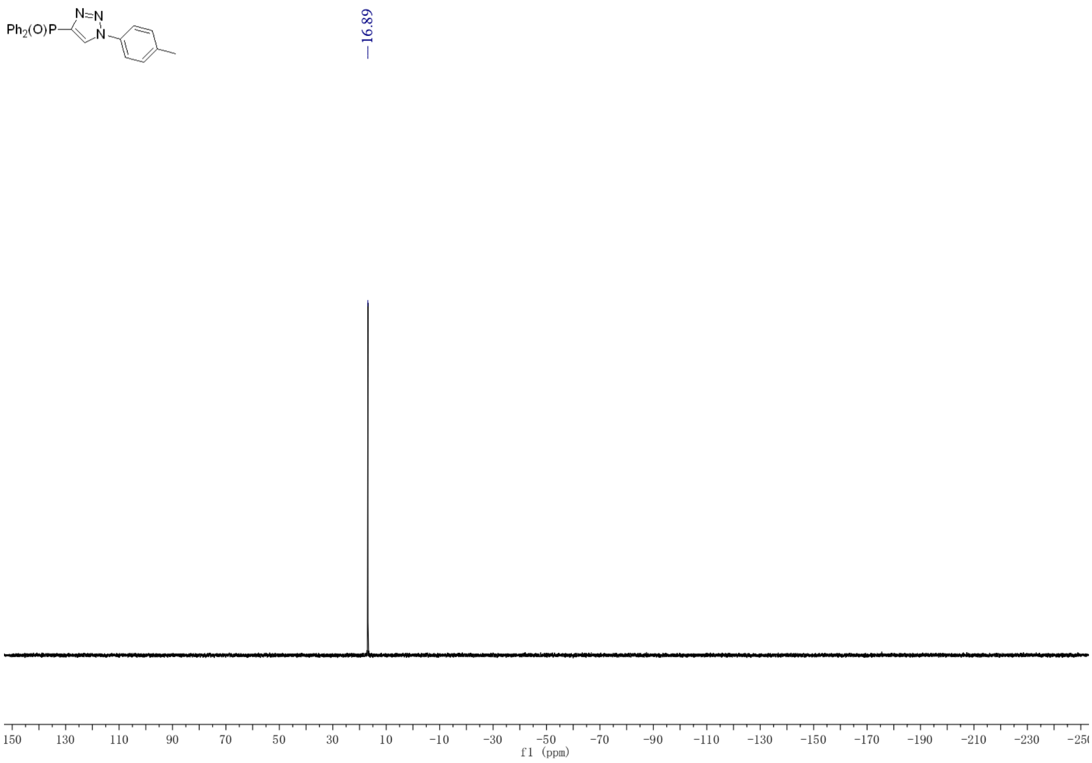

^{1}H-NMR (400 MHz, $CDCl_3$) spectrum of **5ae**

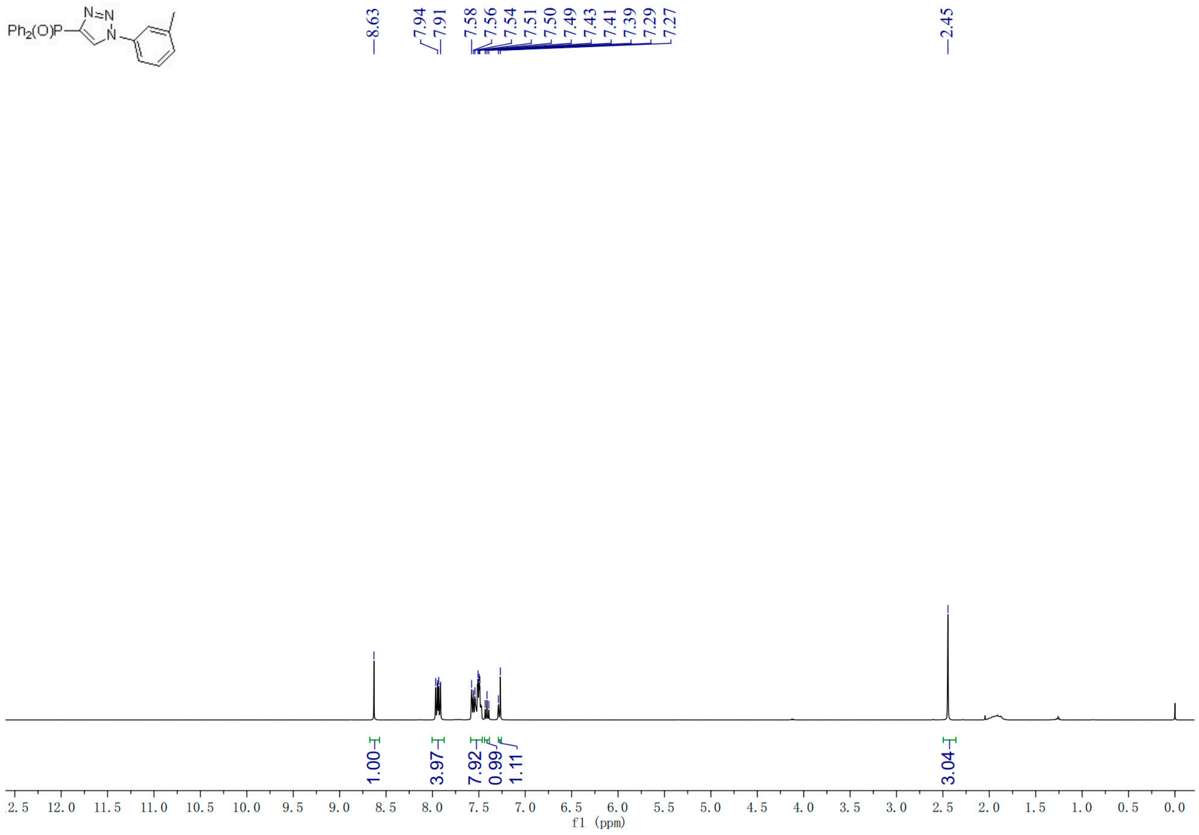

^{13}C-NMR (101 MHz, $CDCl_3$) spectrum of **5ae**

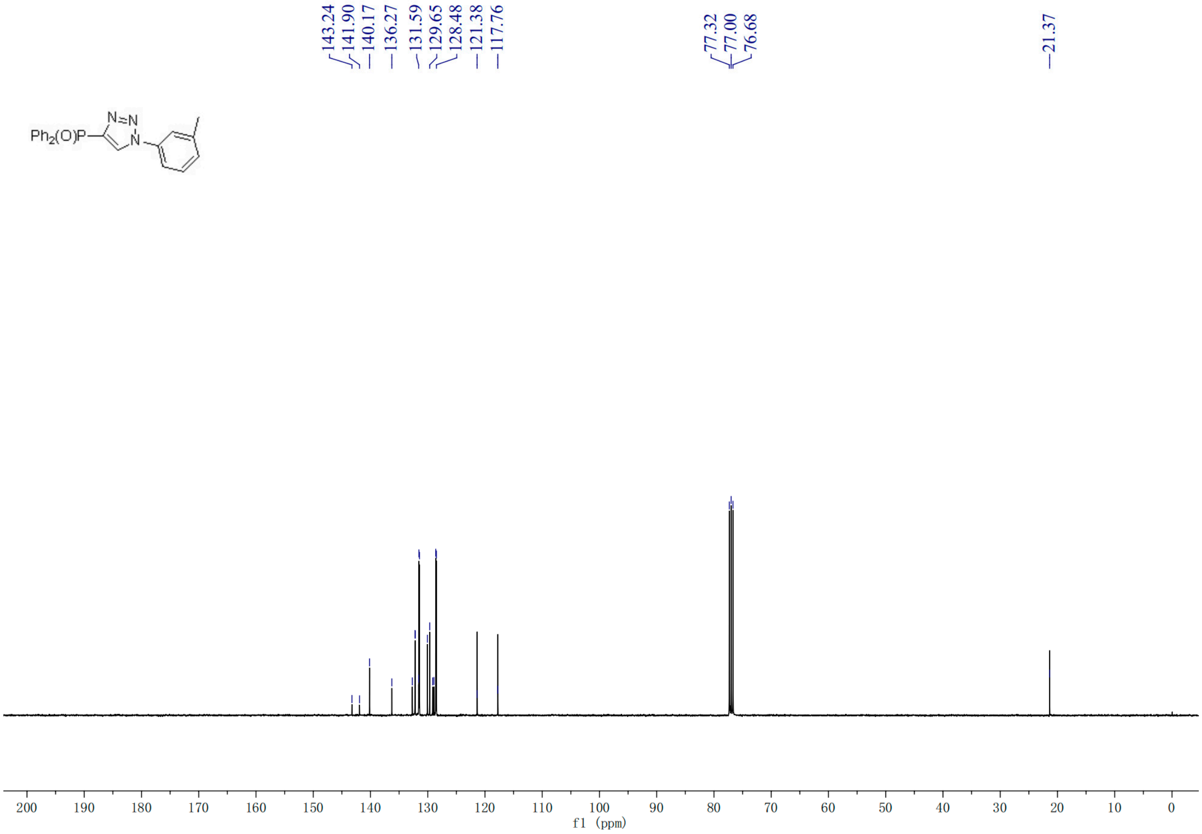

^{31}P NMR (162 MHz, Chloroform-*d*) spectrum of **5ae**

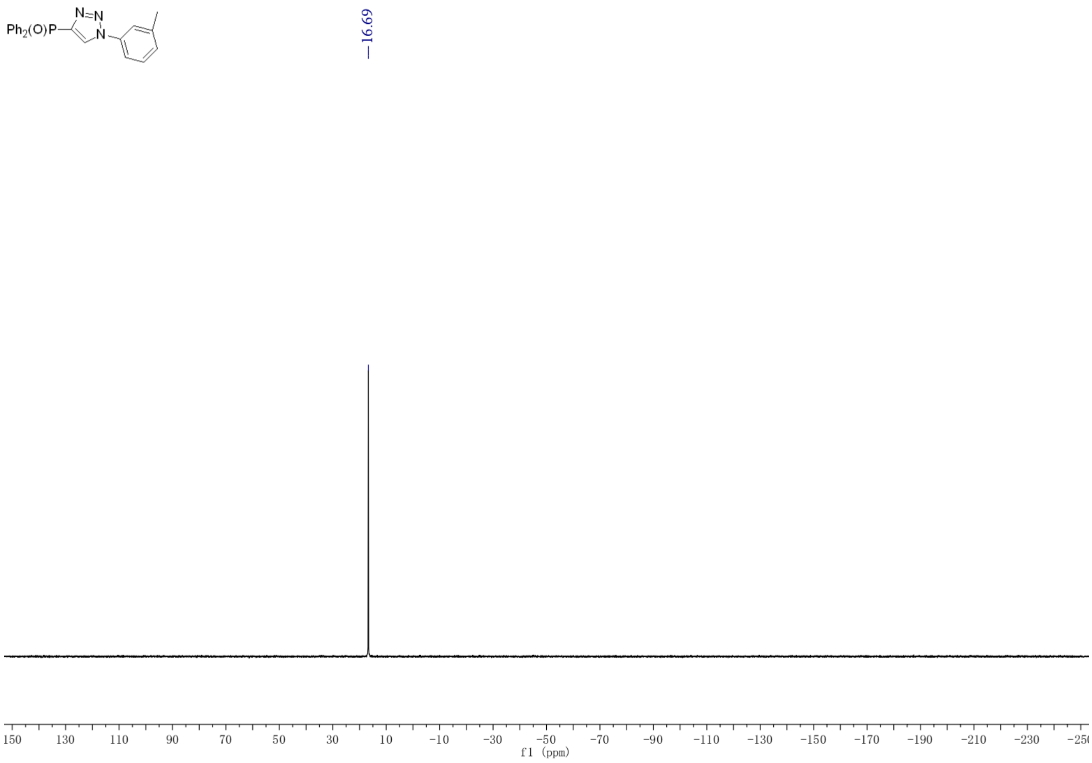

^{1}H-NMR (400 MHz, $CDCl_3$) spectrum of **3af**

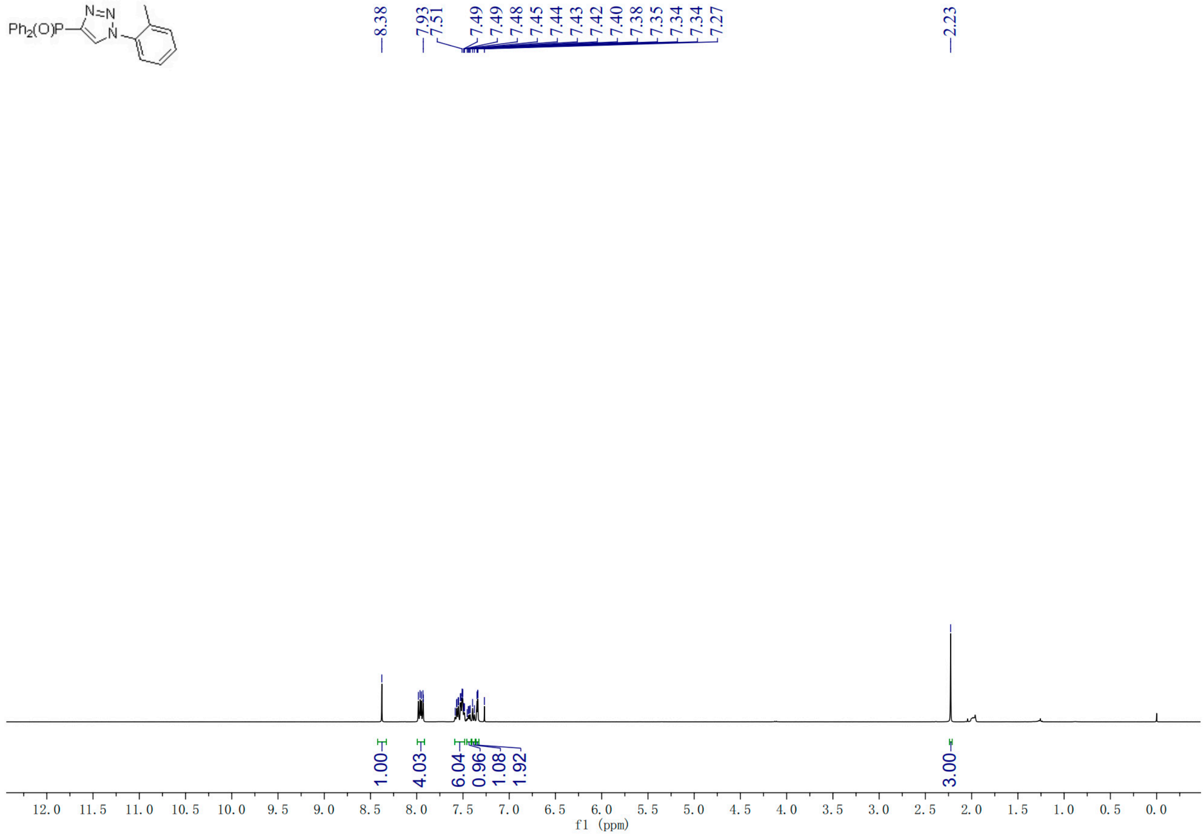

^{13}C-NMR (101 MHz, $CDCl_3$) spectrum of **3af**

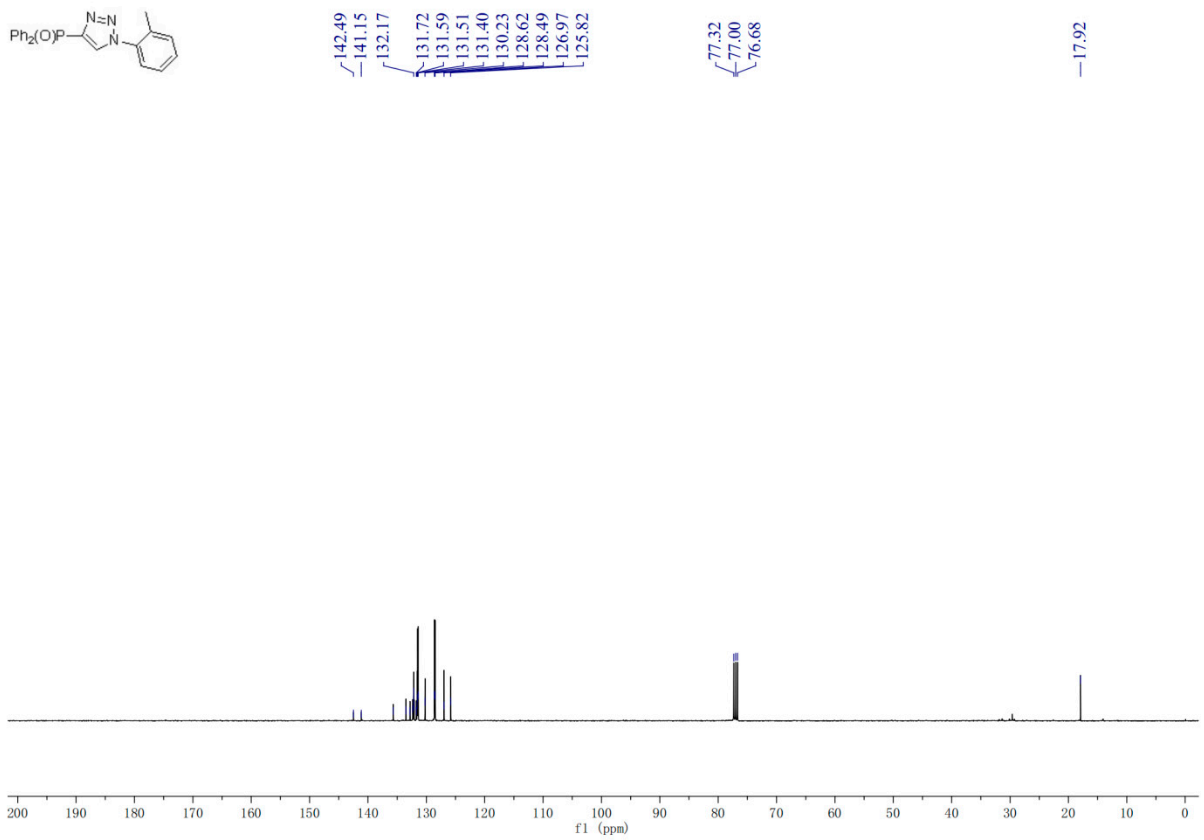

^{31}P NMR (162 MHz, Chloroform-*d*) spectrum of **3af**

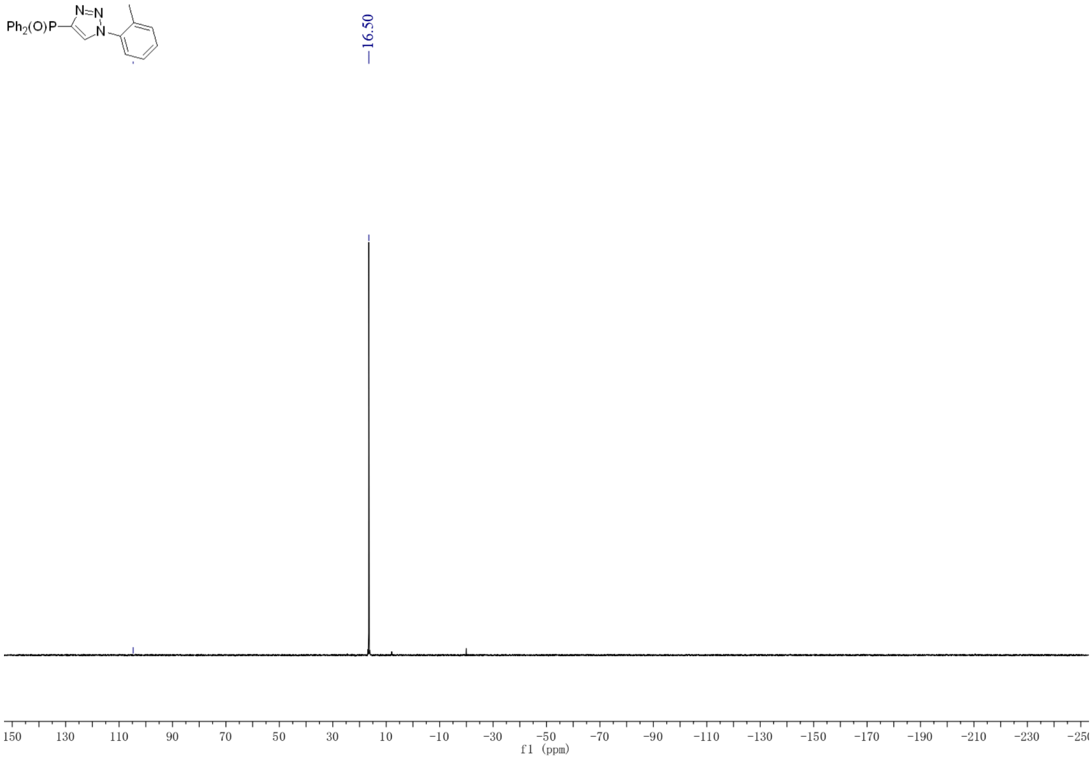

^{1}H-NMR (400 MHz, $CDCl_3$) spectrum of **3ag**

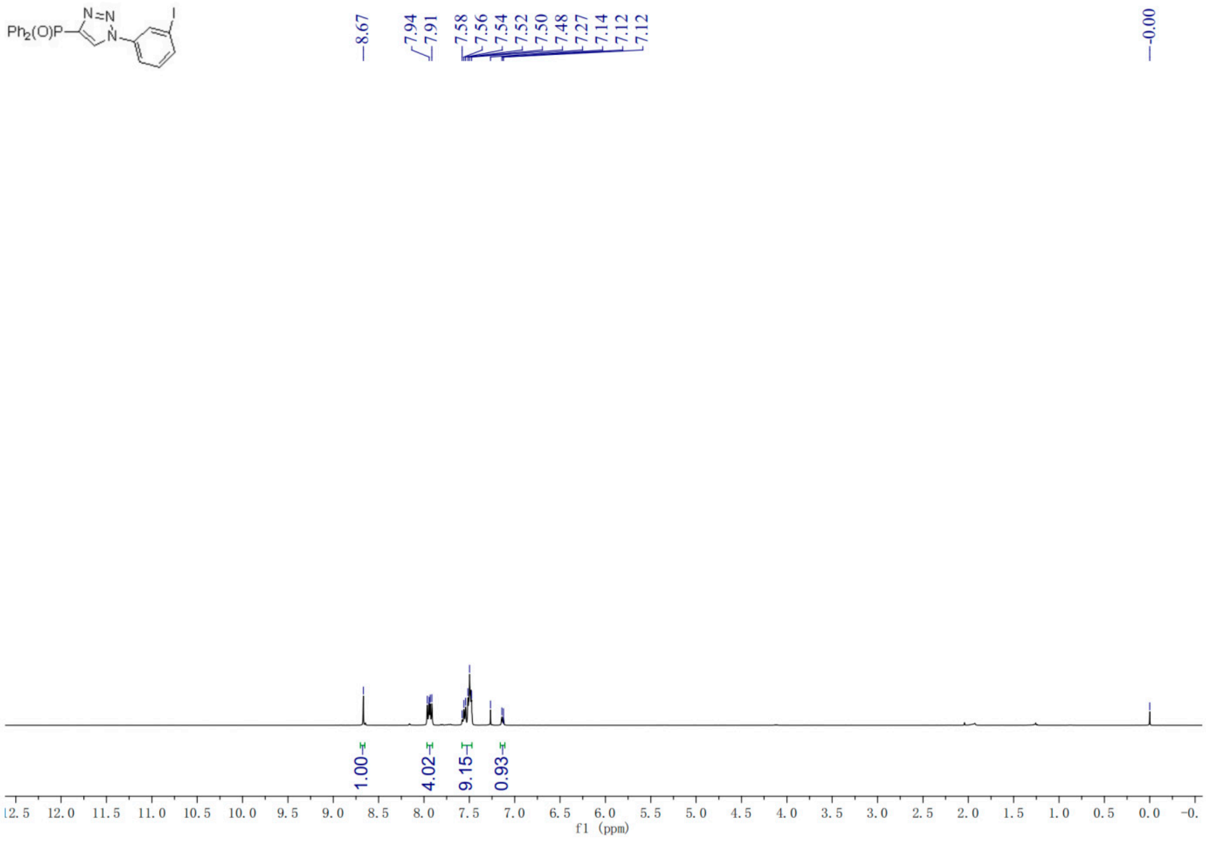

^{13}C-NMR (101 MHz, $CDCl_3$) spectrum of **3ag**

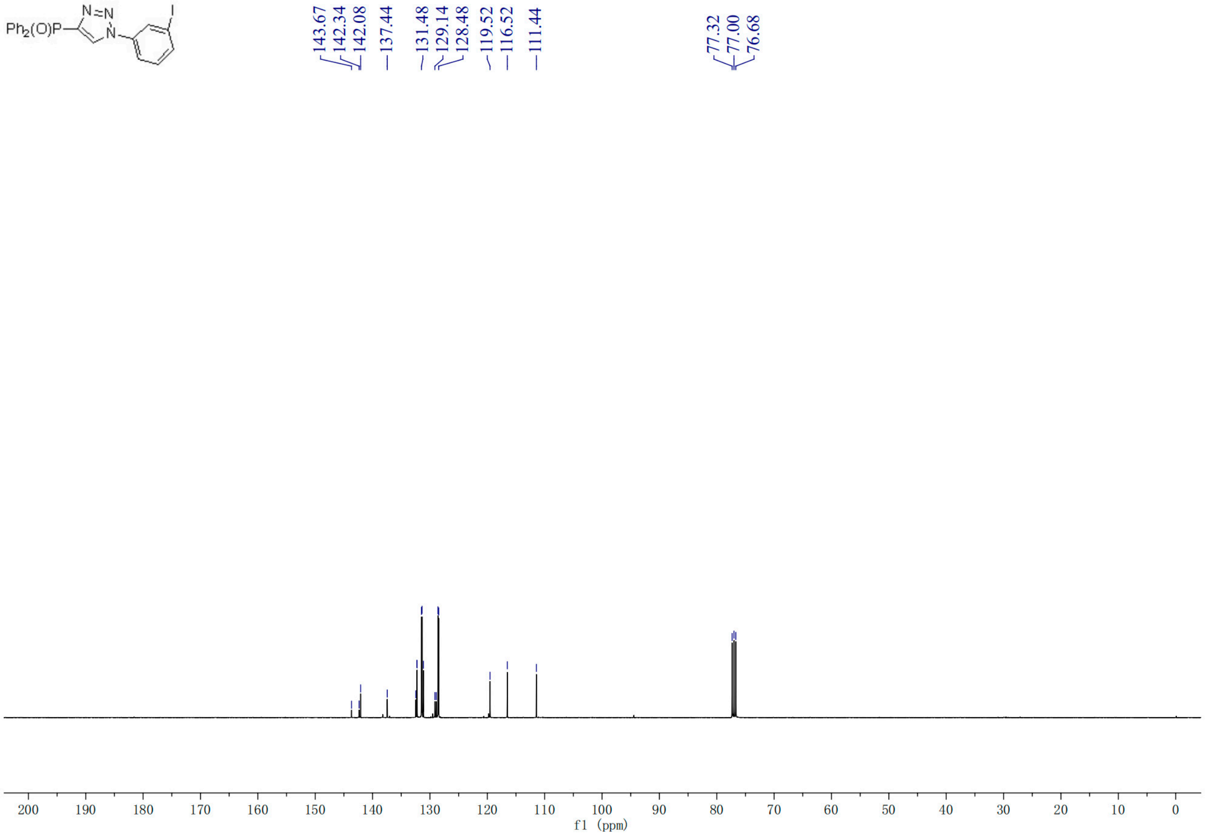

^{31}P NMR (162 MHz, Chloroform-*d*) spectrum of **3ag**

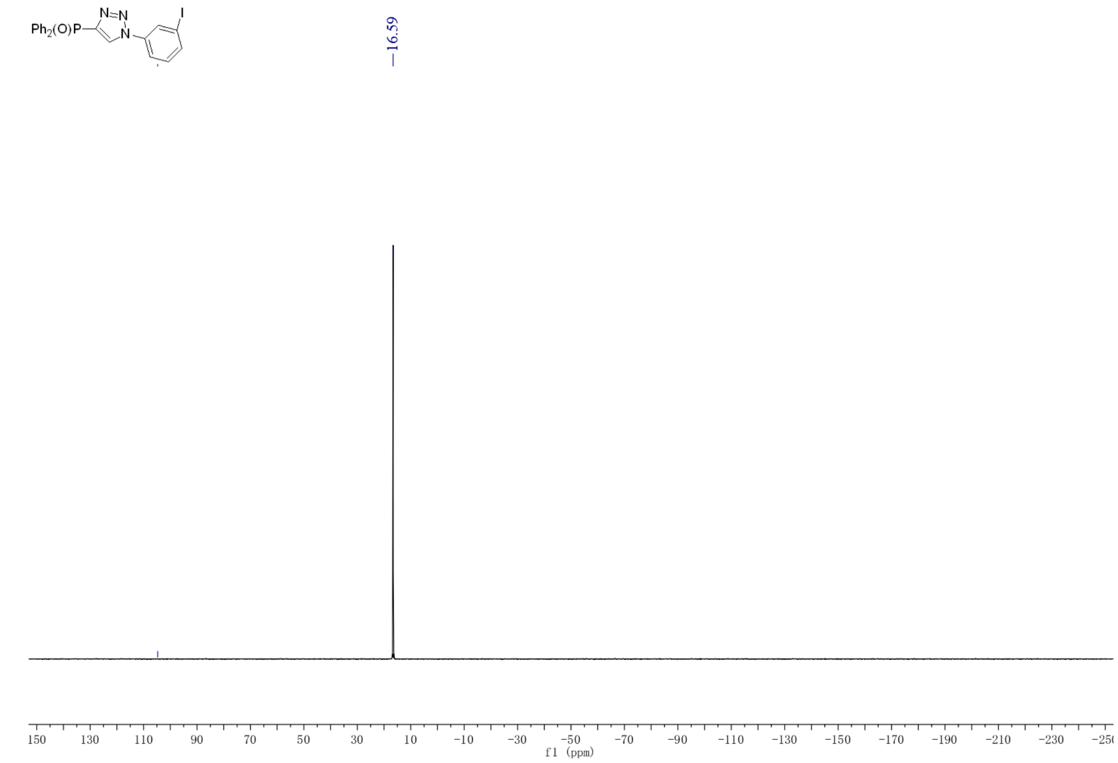

^{1}H-NMR (400 MHz, $CDCl_3$) spectrum of **3ah**

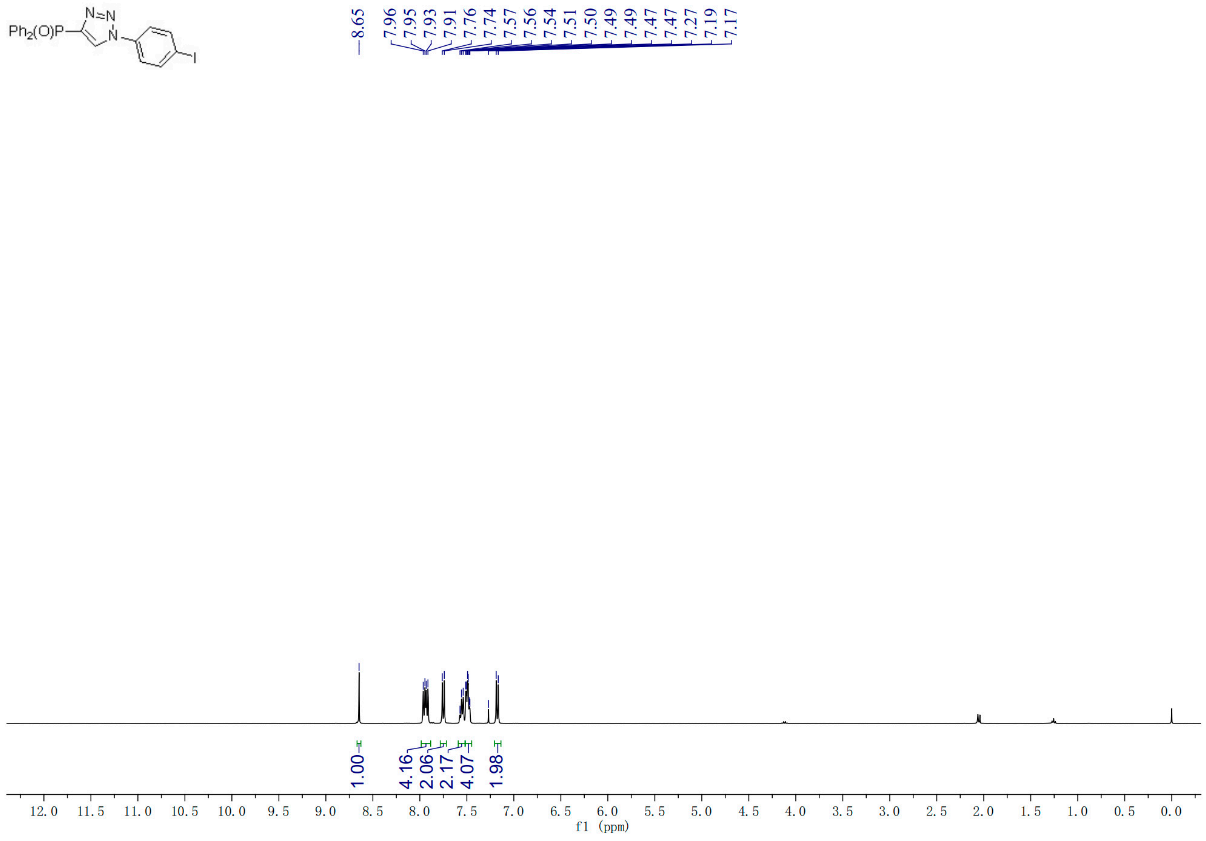

^{13}C-NMR (101 MHz, $CDCl_3$) spectrum of **3ah**

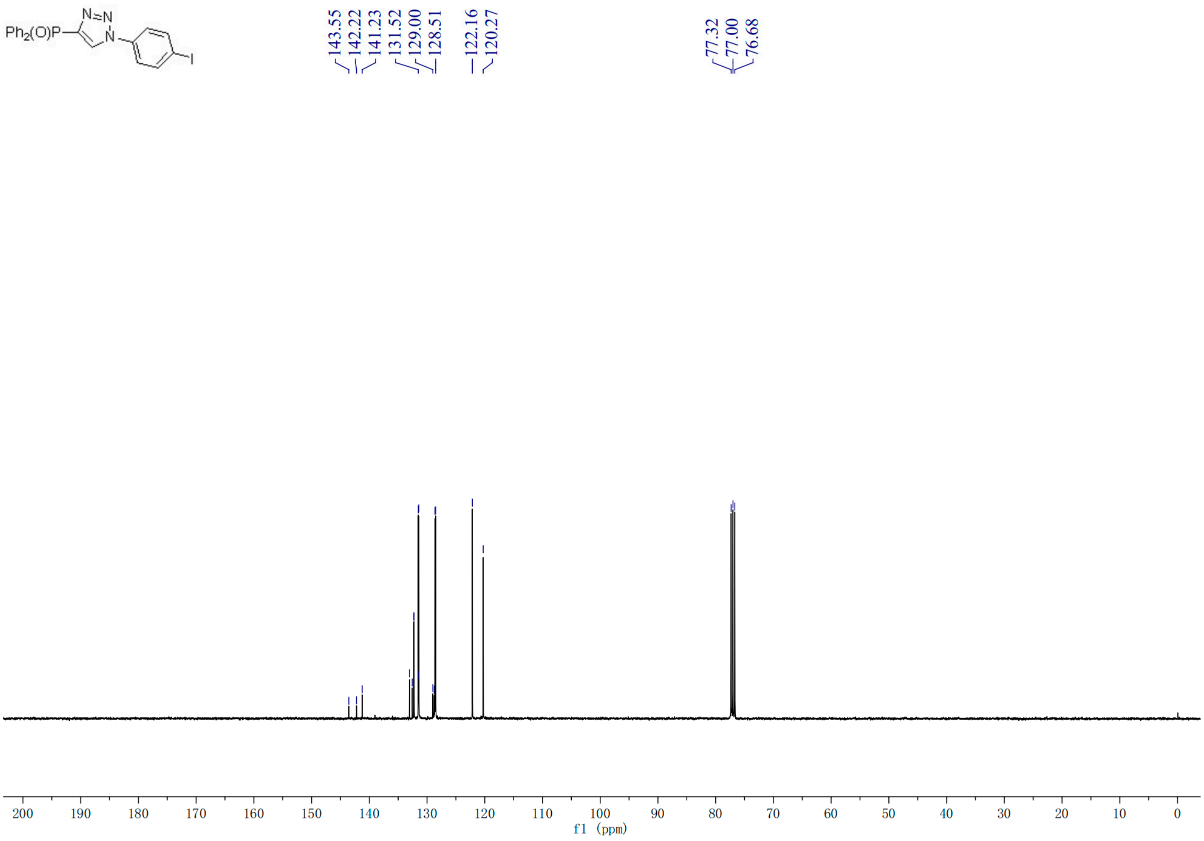

^{31}P NMR (162 MHz, Chloroform-*d*) spectrum of **3ah**

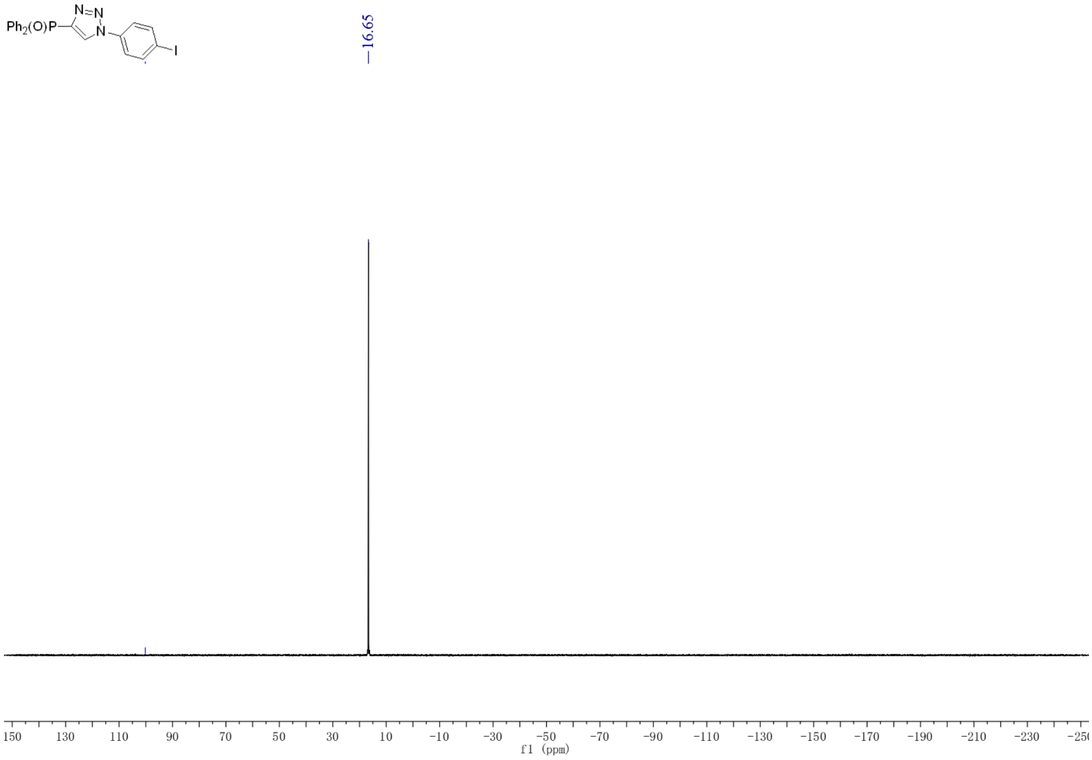

^{1}H-NMR (400 MHz, $CDCl_3$) spectrum of **4a**

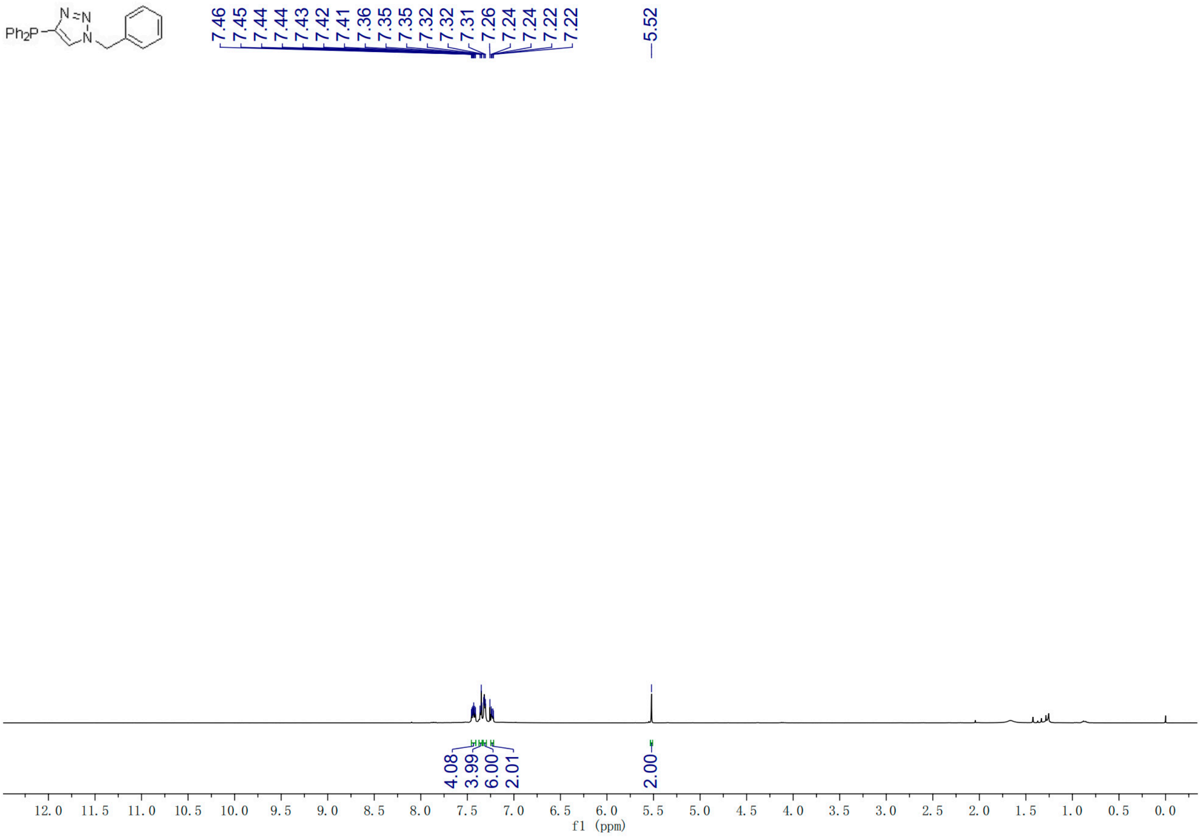

^{13}C-NMR (101 MHz, $CDCl_3$) spectrum of **4a**

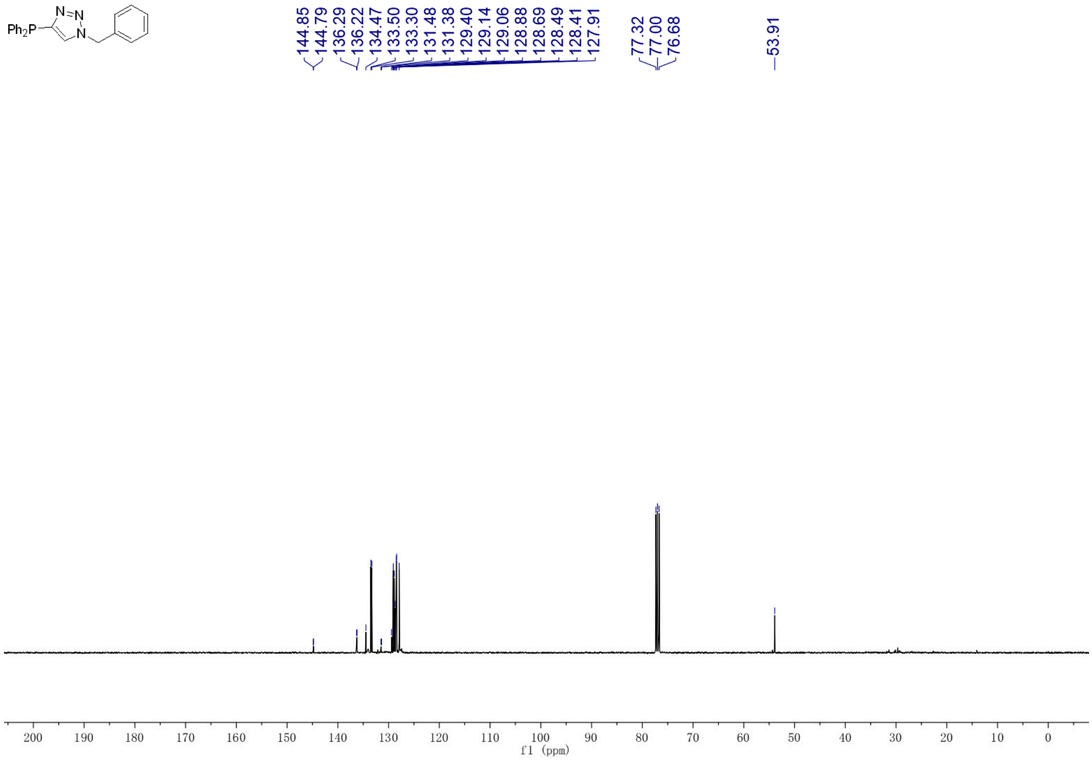

^{31}P NMR (162 MHz, Chloroform-*d*) spectrum of **4a**

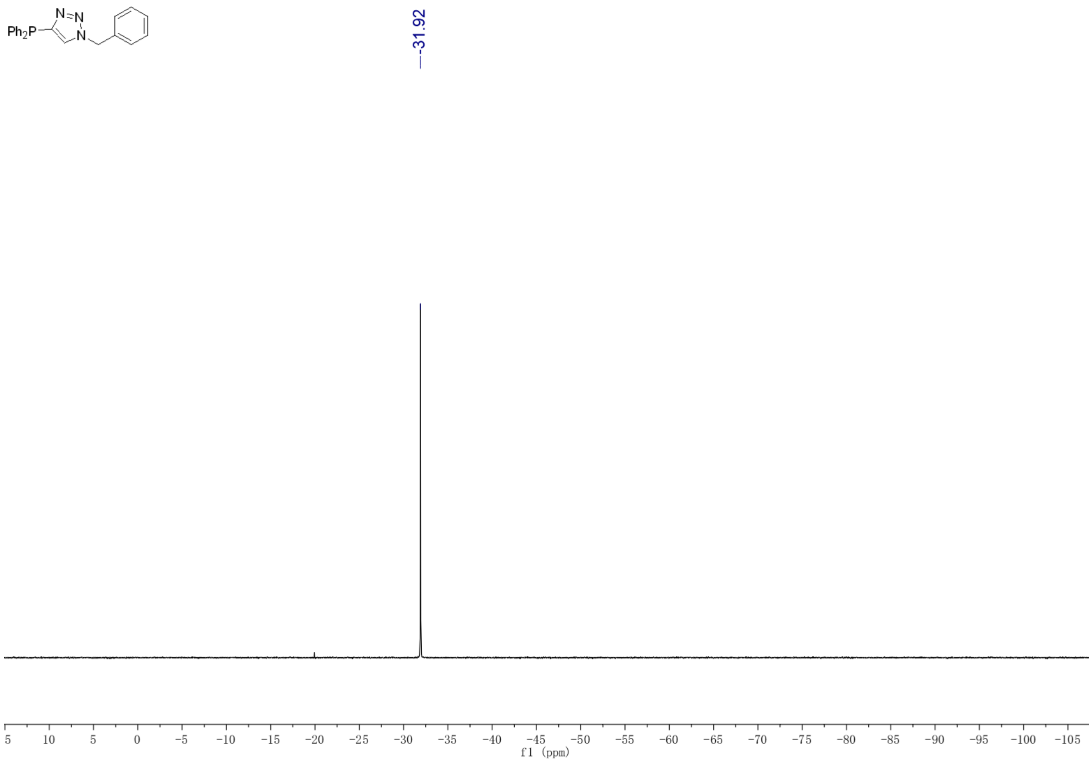

^{1}H-NMR (400 MHz, $CDCl_3$) spectrum of **4b**

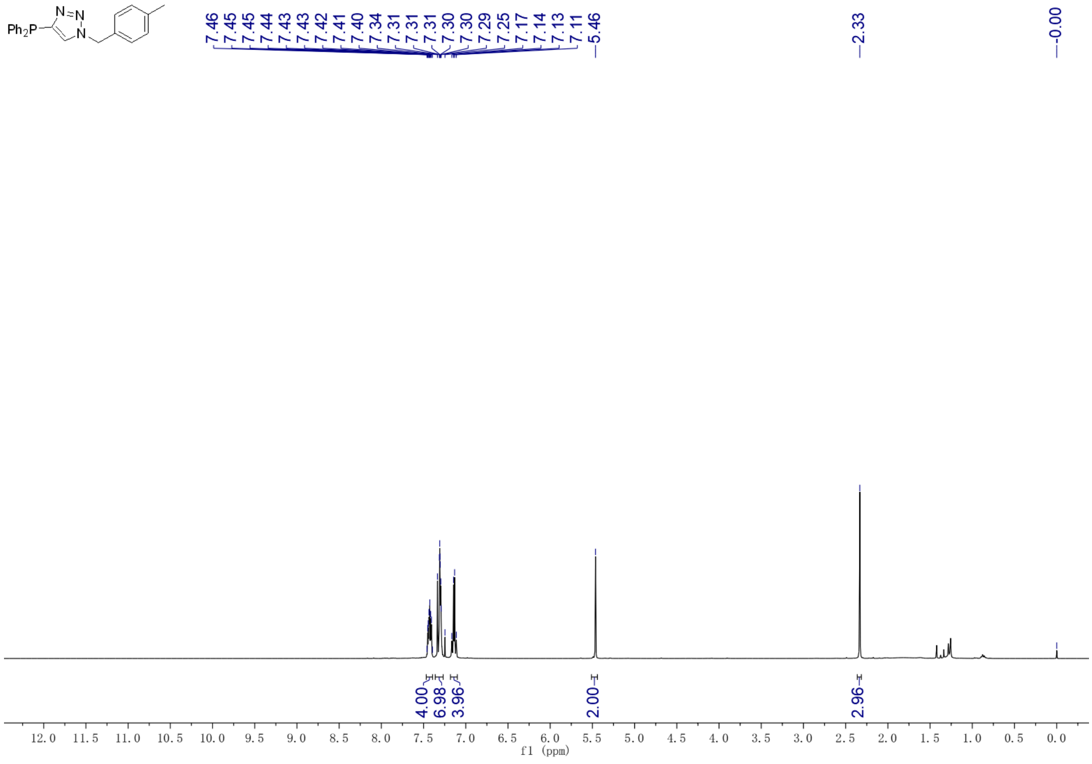

^{13}C-NMR (101 MHz, $CDCl_3$) spectrum of **4b**

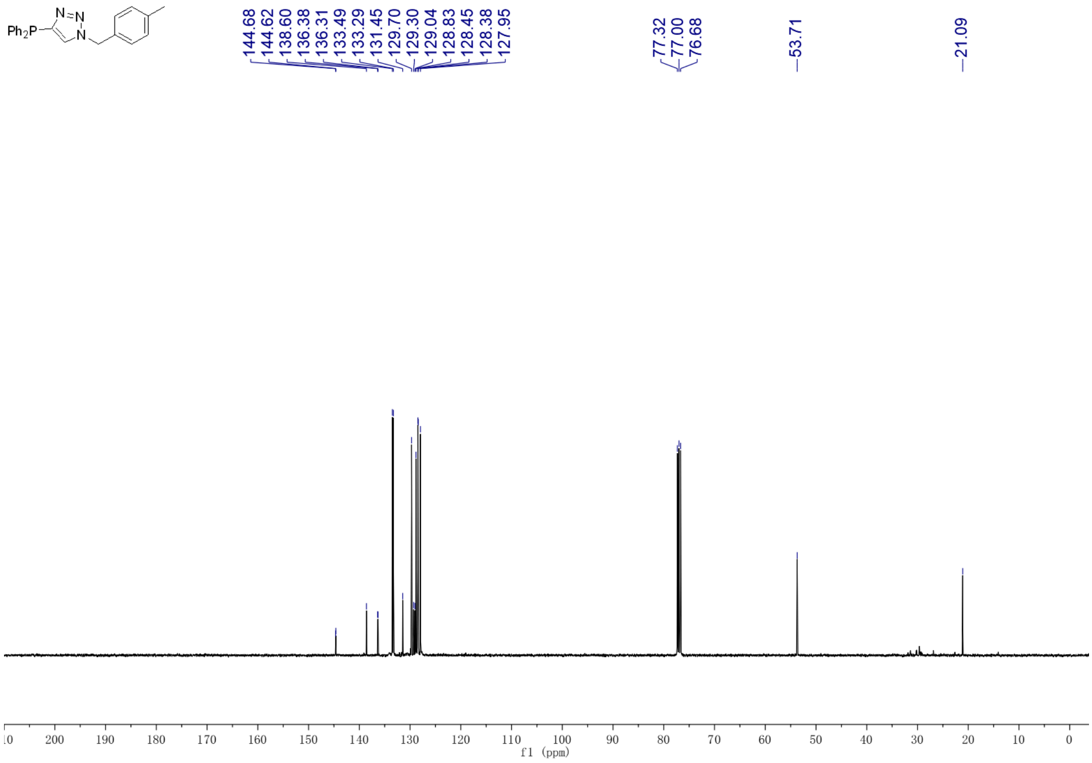

^{31}P NMR (162 MHz, Chloroform-*d*) spectrum of **4b**

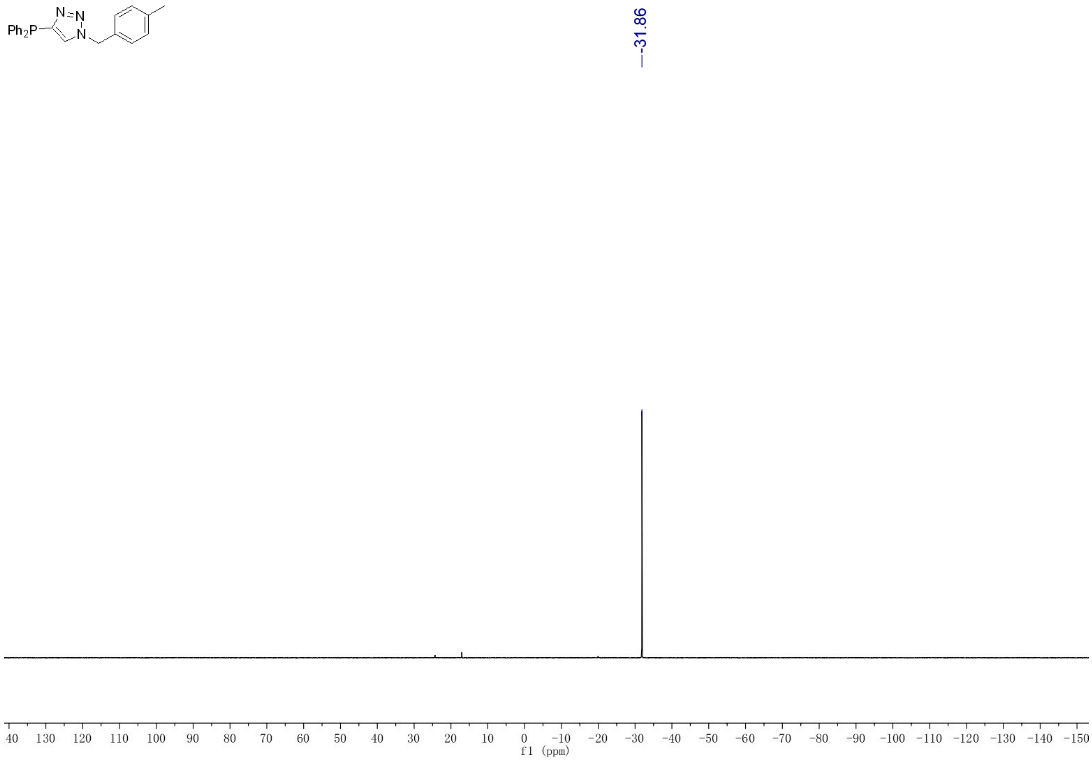

^{1}H-NMR (400 MHz, $CDCl_3$) spectrum of **4c**

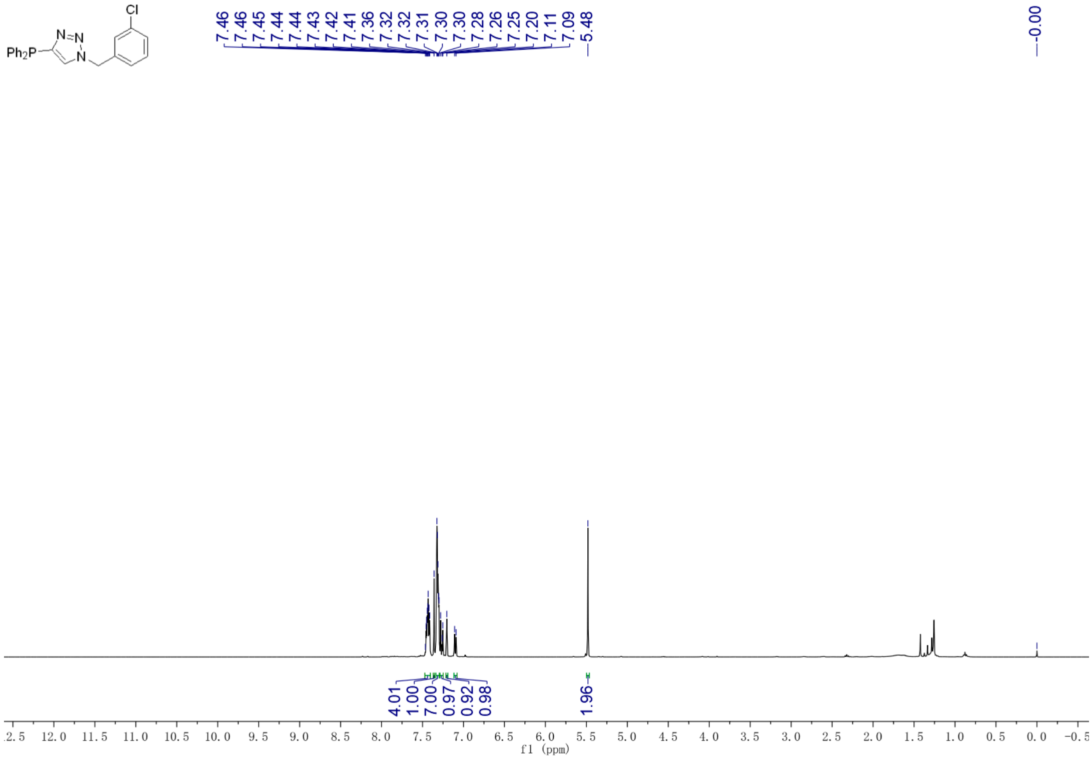

^{13}C-NMR (101 MHz, $CDCl_3$) spectrum of **4c**

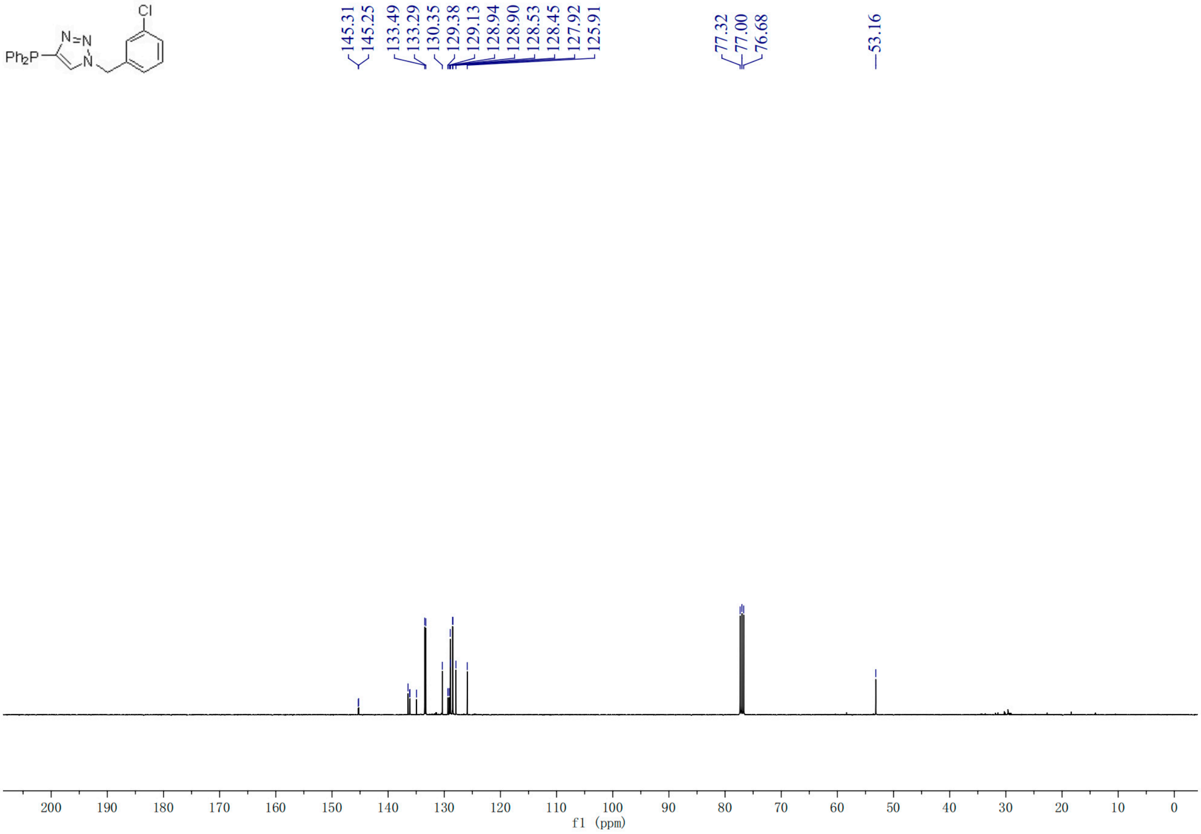

^{31}P NMR (162 MHz, Chloroform-*d*) spectrum of **4c**

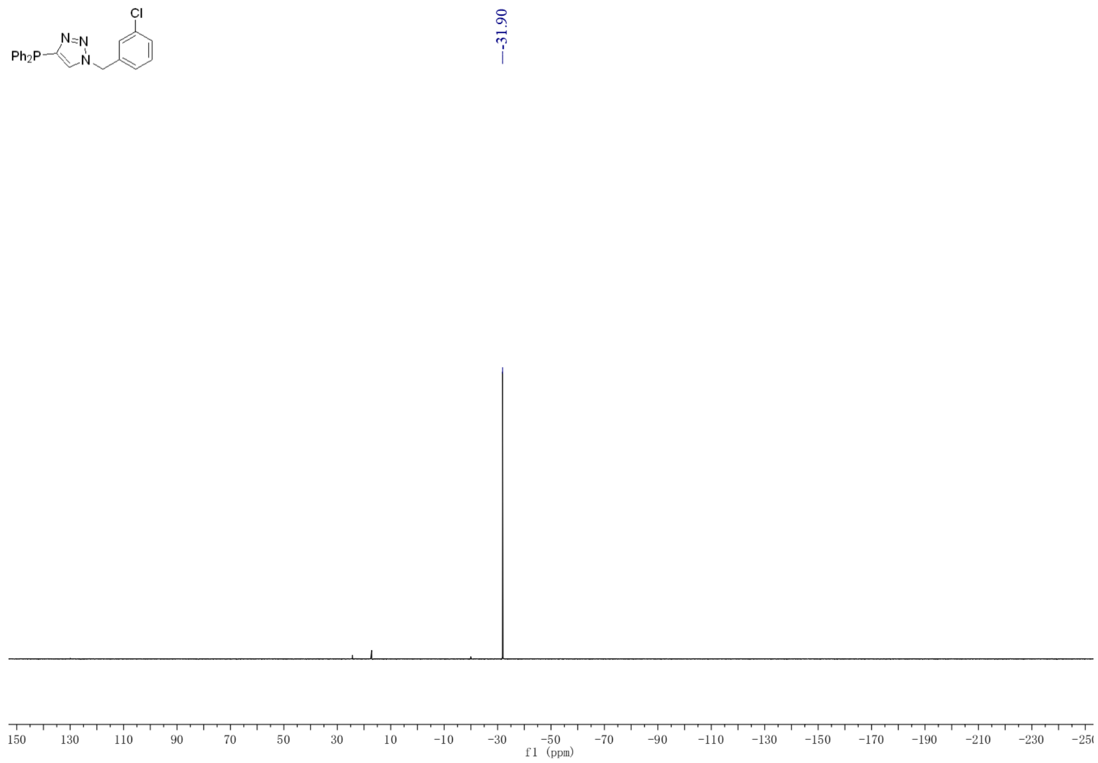

^{1}H-NMR (400 MHz, $CDCl_3$) spectrum of **4d**

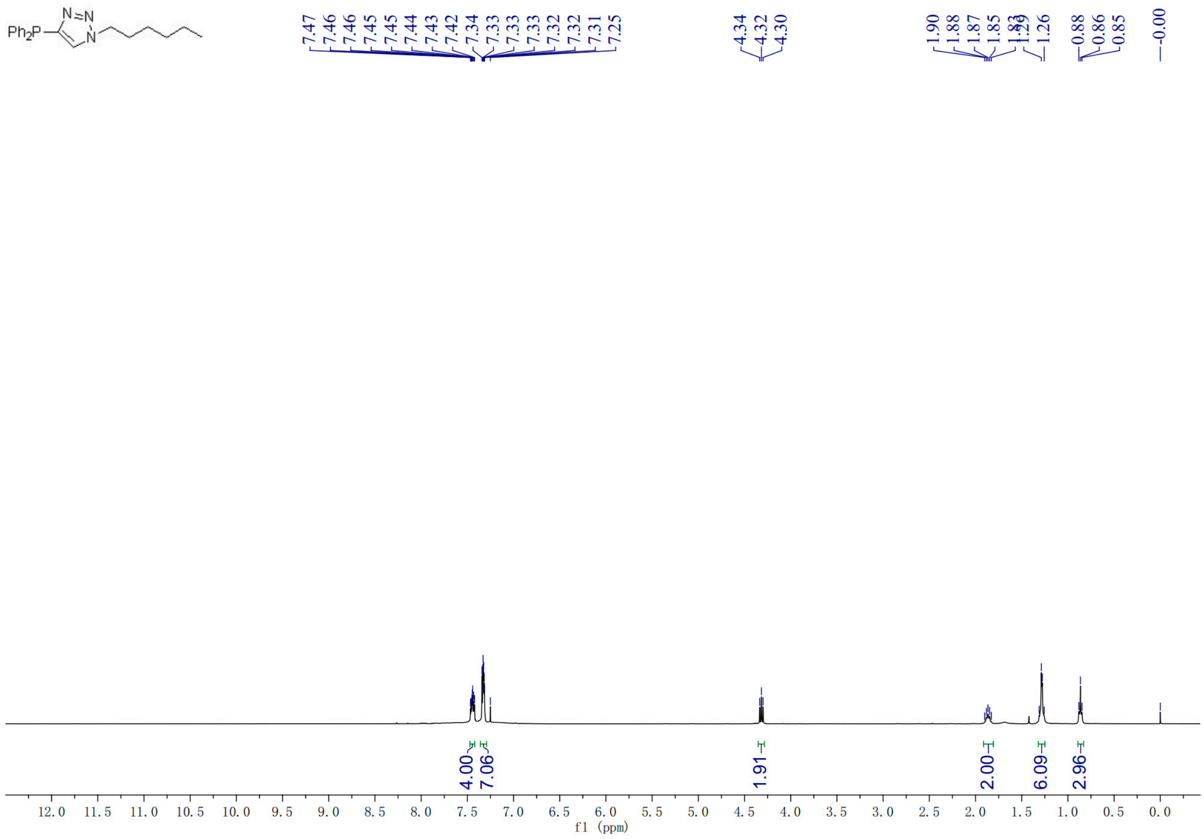

^{13}C-NMR (101 MHz, $CDCl_3$) spectrum of **4d**

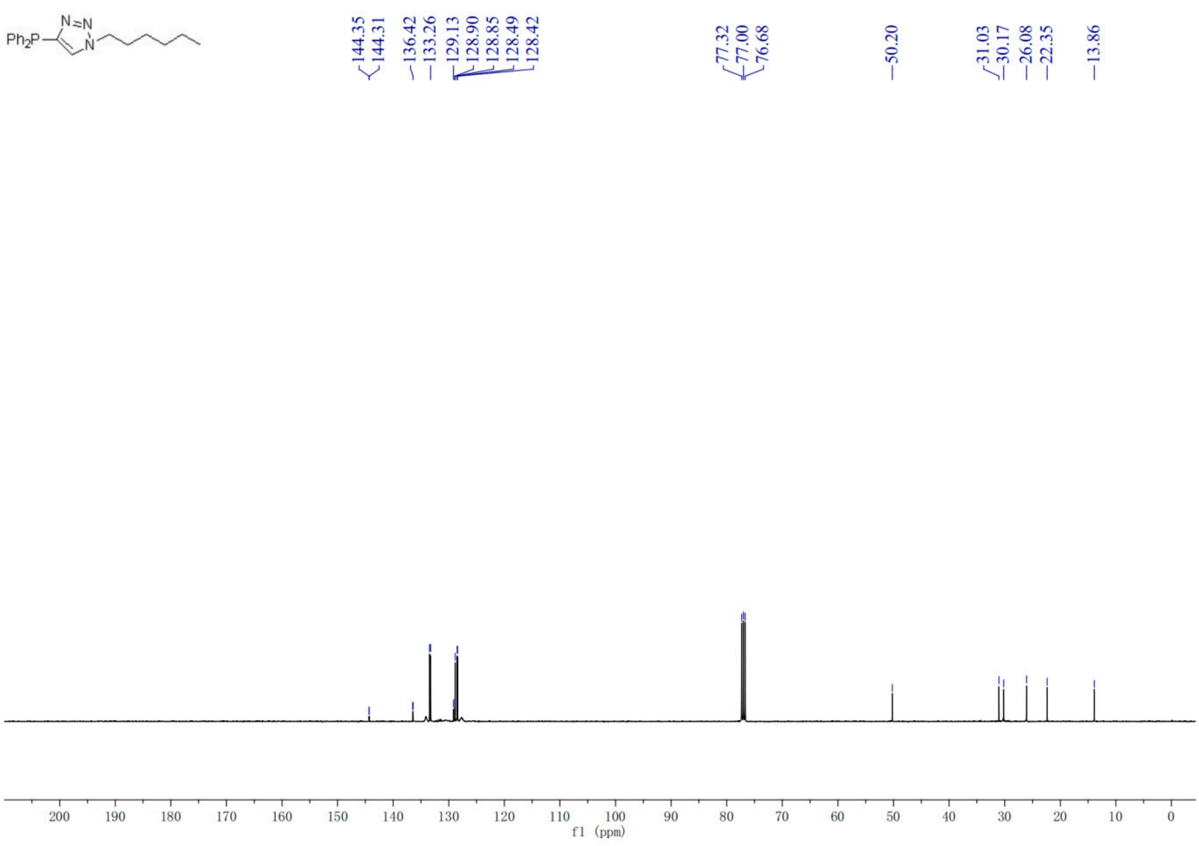

^{31}P NMR (162 MHz, Chloroform-*d*) spectrum of **4d**

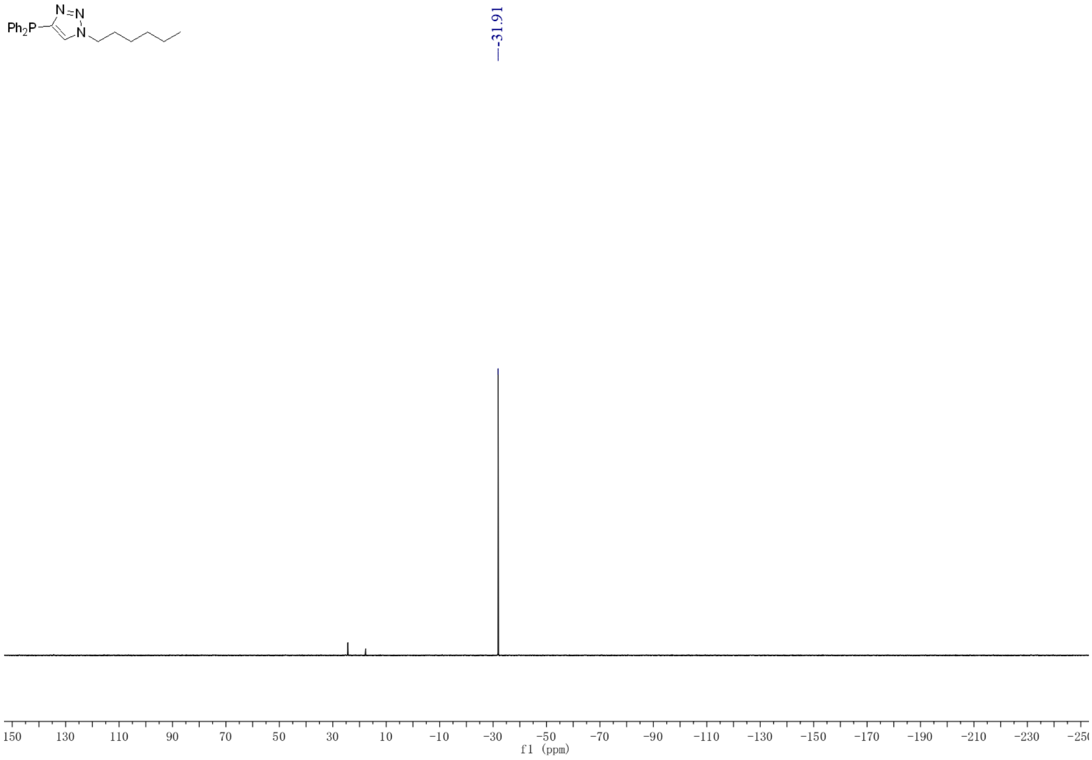

^{1}H-NMR (400 MHz, $CDCl_3$) spectrum of **4e**

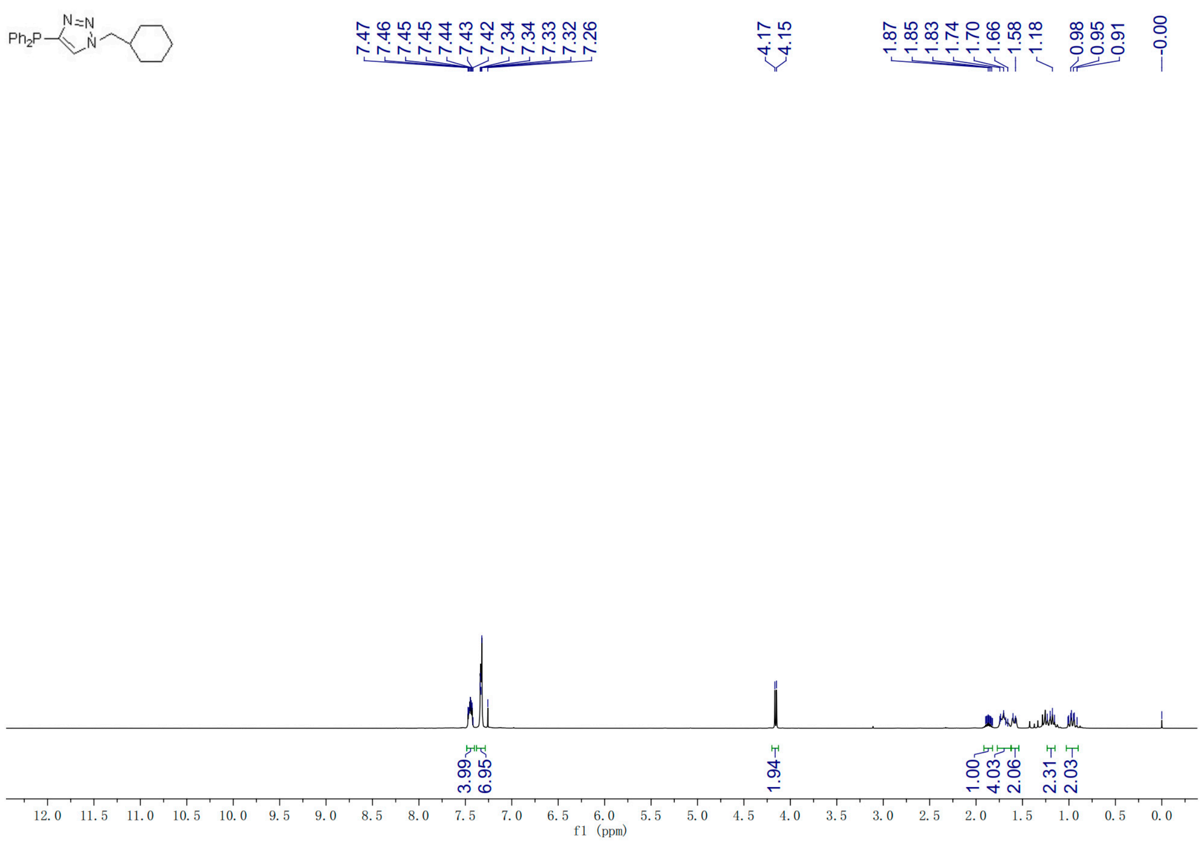

^{13}C-NMR (101 MHz, $CDCl_3$) spectrum of **4e**

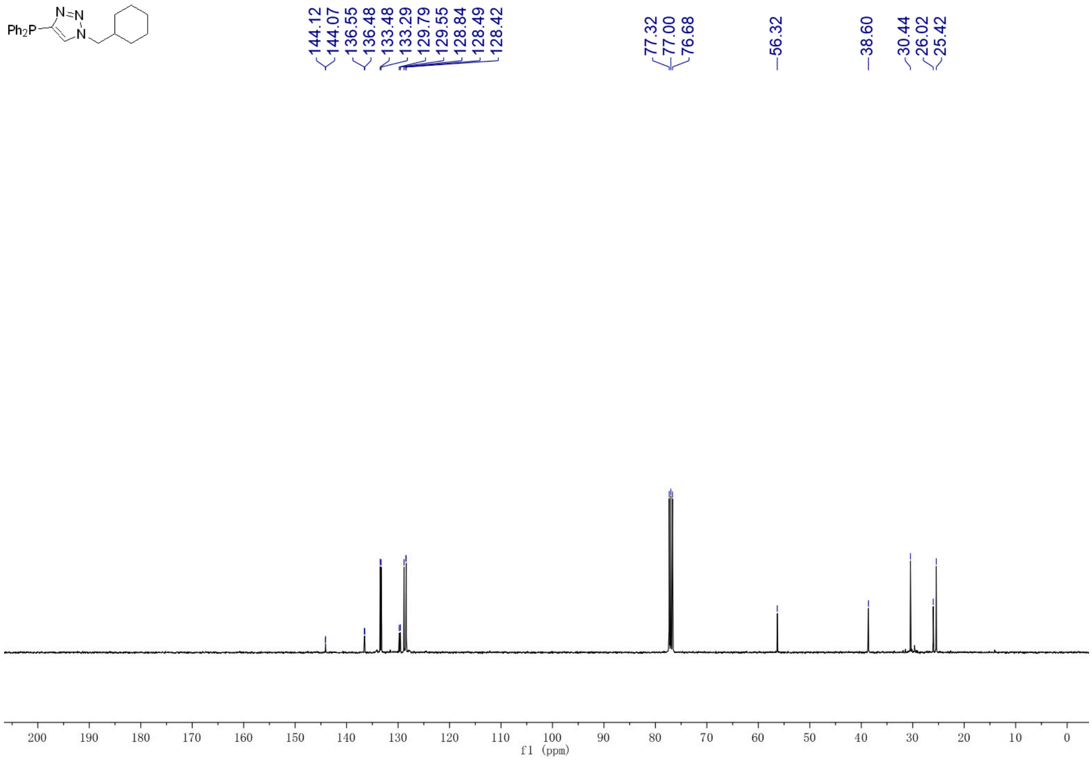

^{31}P NMR (162 MHz, Chloroform-*d*) spectrum of **4e**

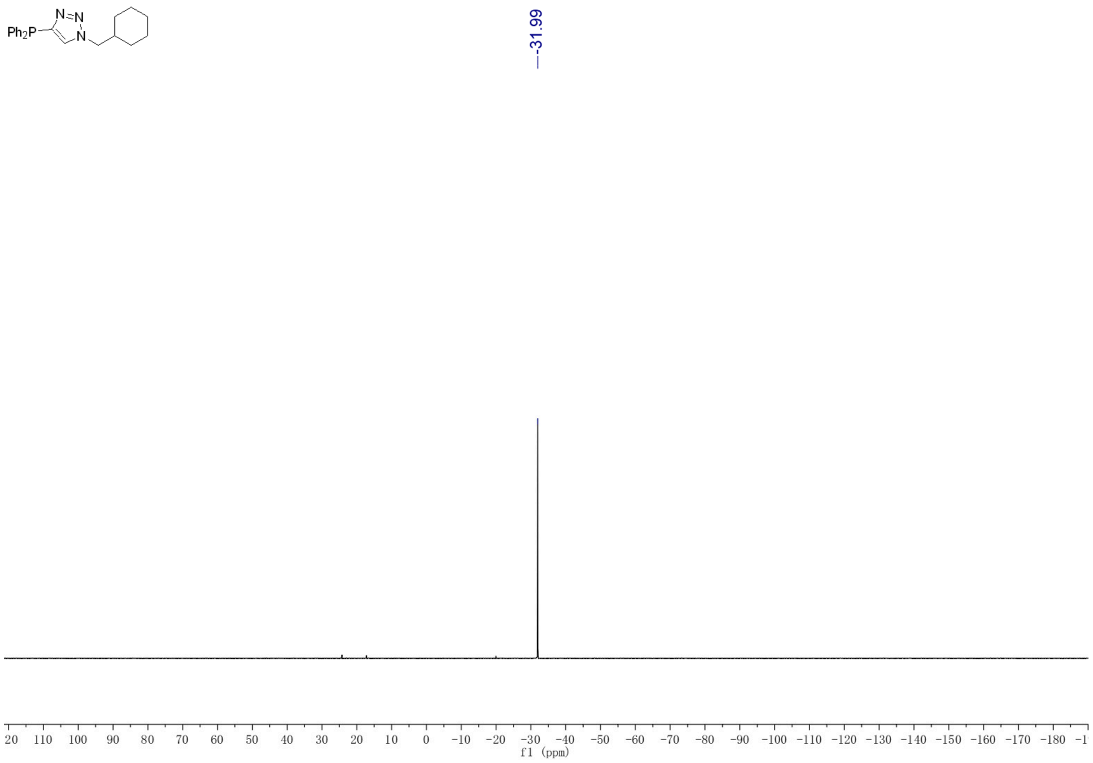

^{1}H-NMR (400 MHz, $CDCl_3$) spectrum of **5a**

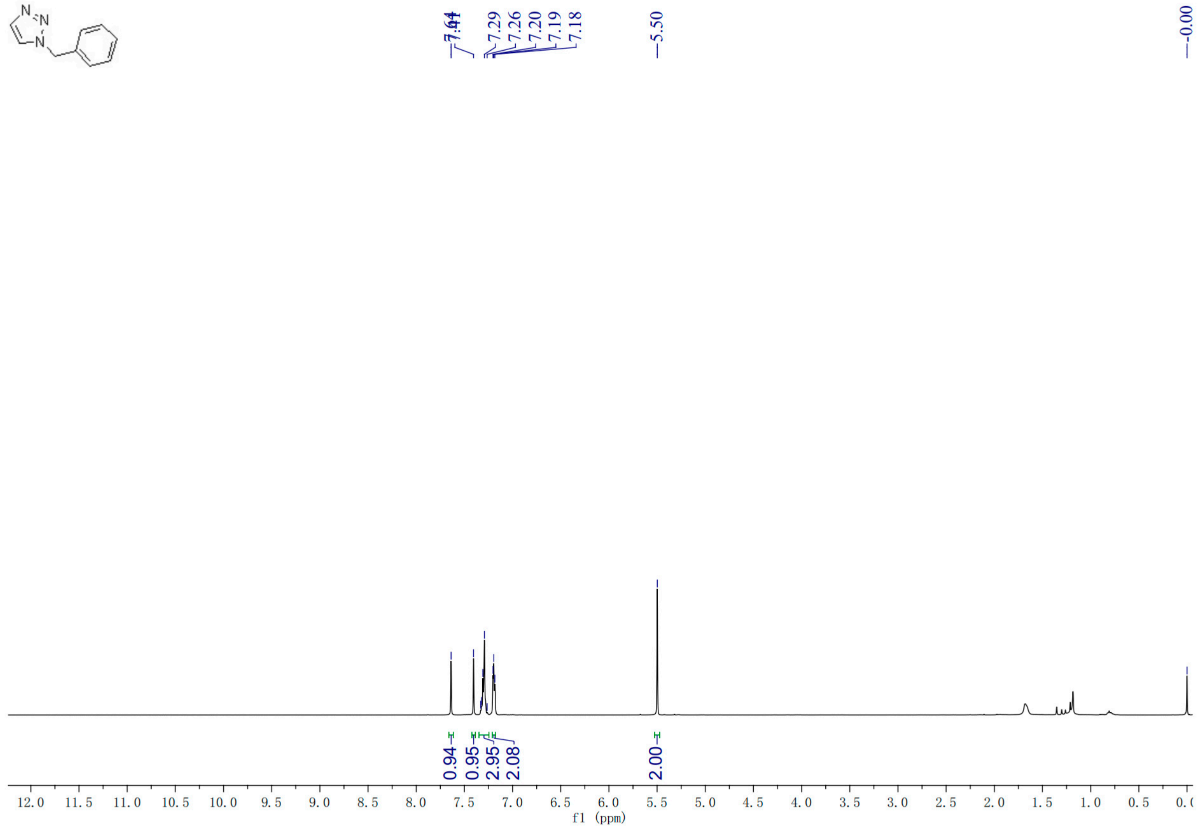

^{13}C-NMR (101 MHz, $CDCl_3$) spectrum of **5a**

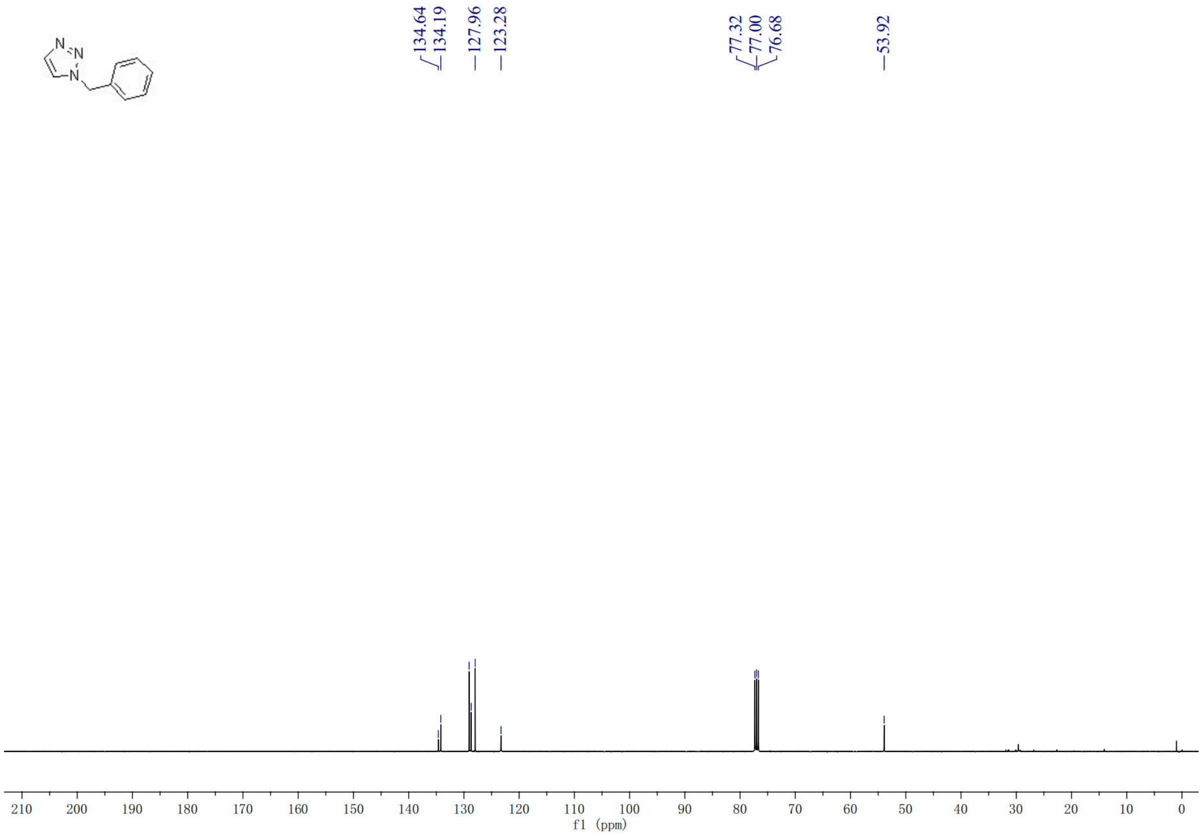

^{1}H-NMR (400 MHz, $CDCl_3$) spectrum of **5b**

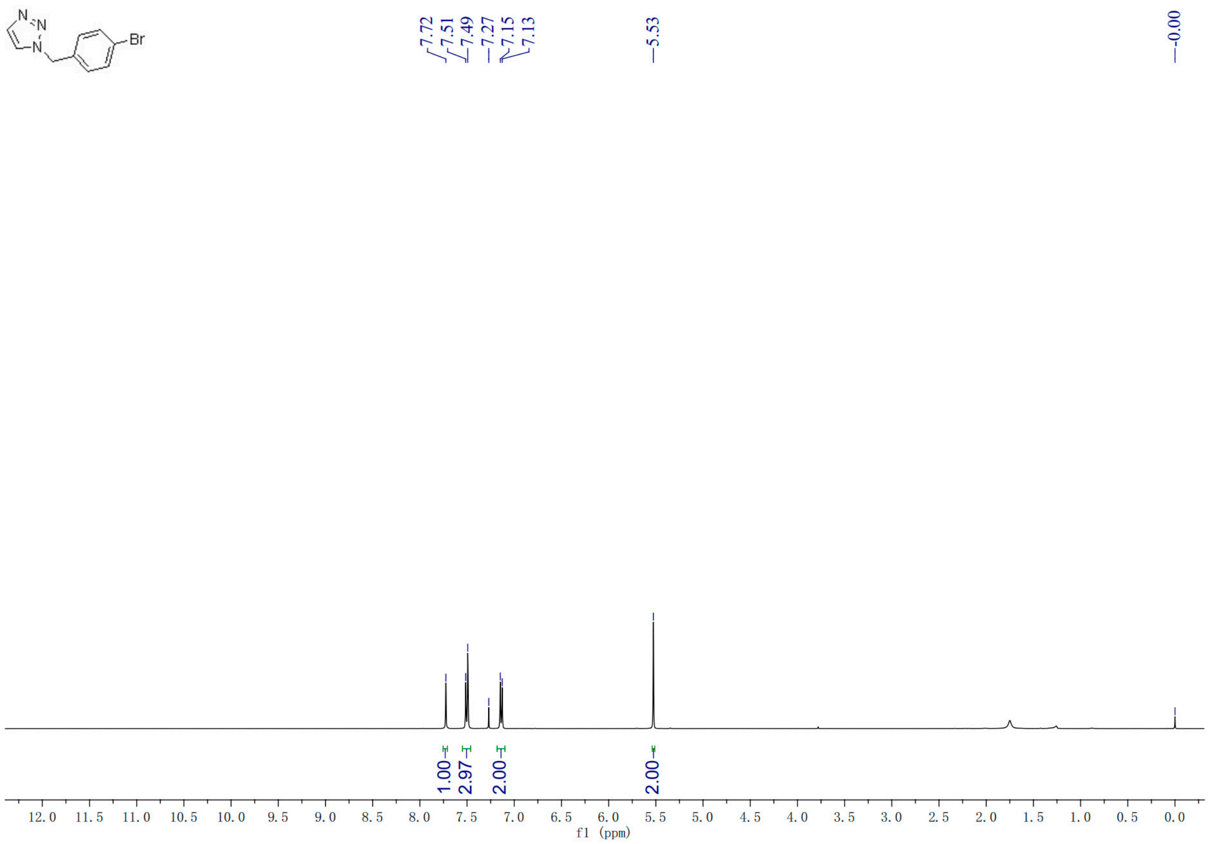

^{13}C-NMR (101 MHz, $CDCl_3$) spectrum of **5b**

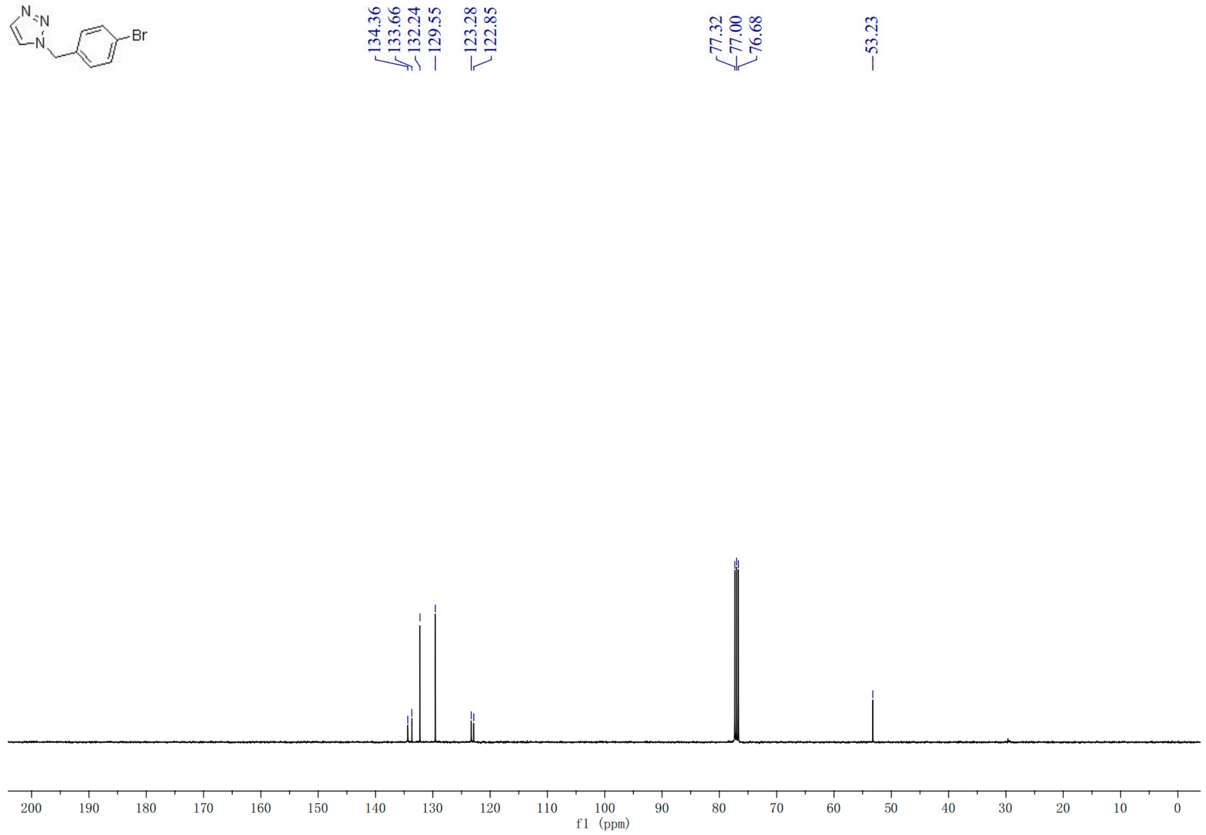

^{1}H-NMR (400 MHz, $CDCl_3$) spectrum of **5c**

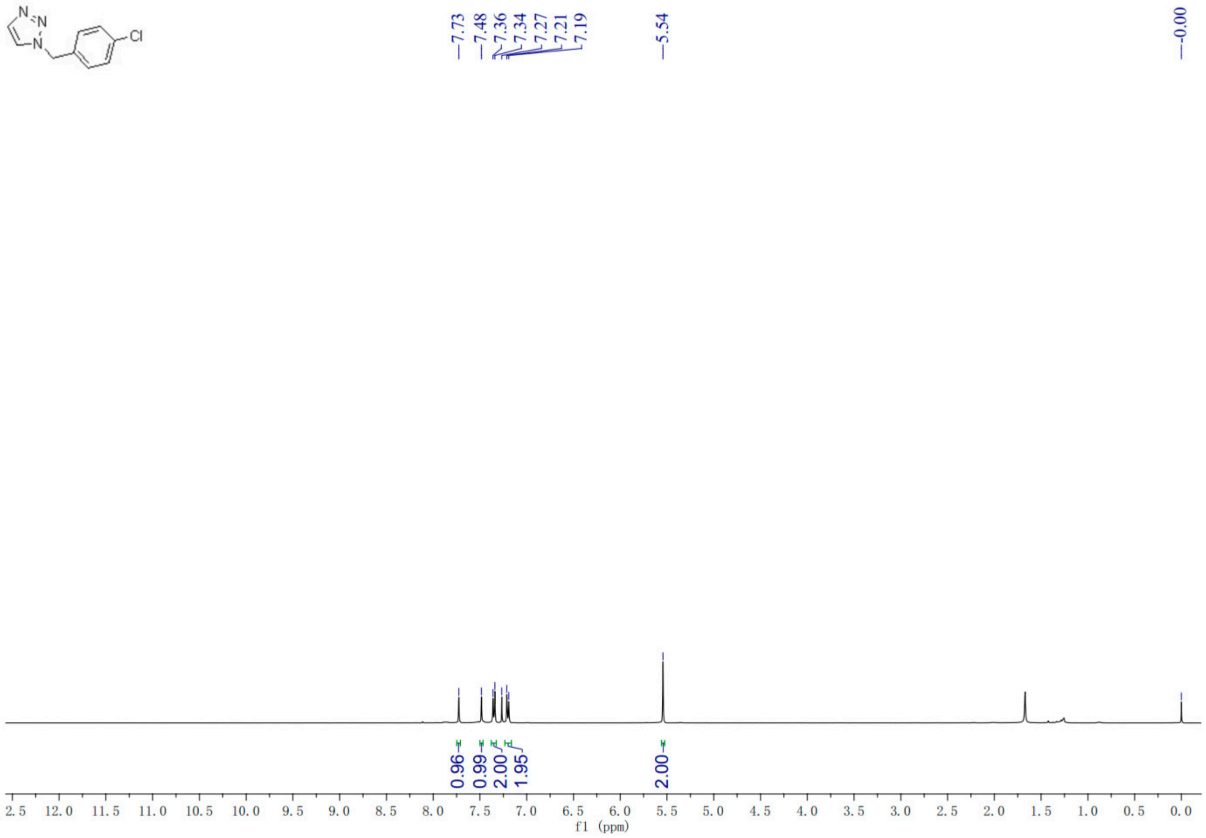

^{13}C-NMR (101 MHz, $CDCl_3$) spectrum of **5c**

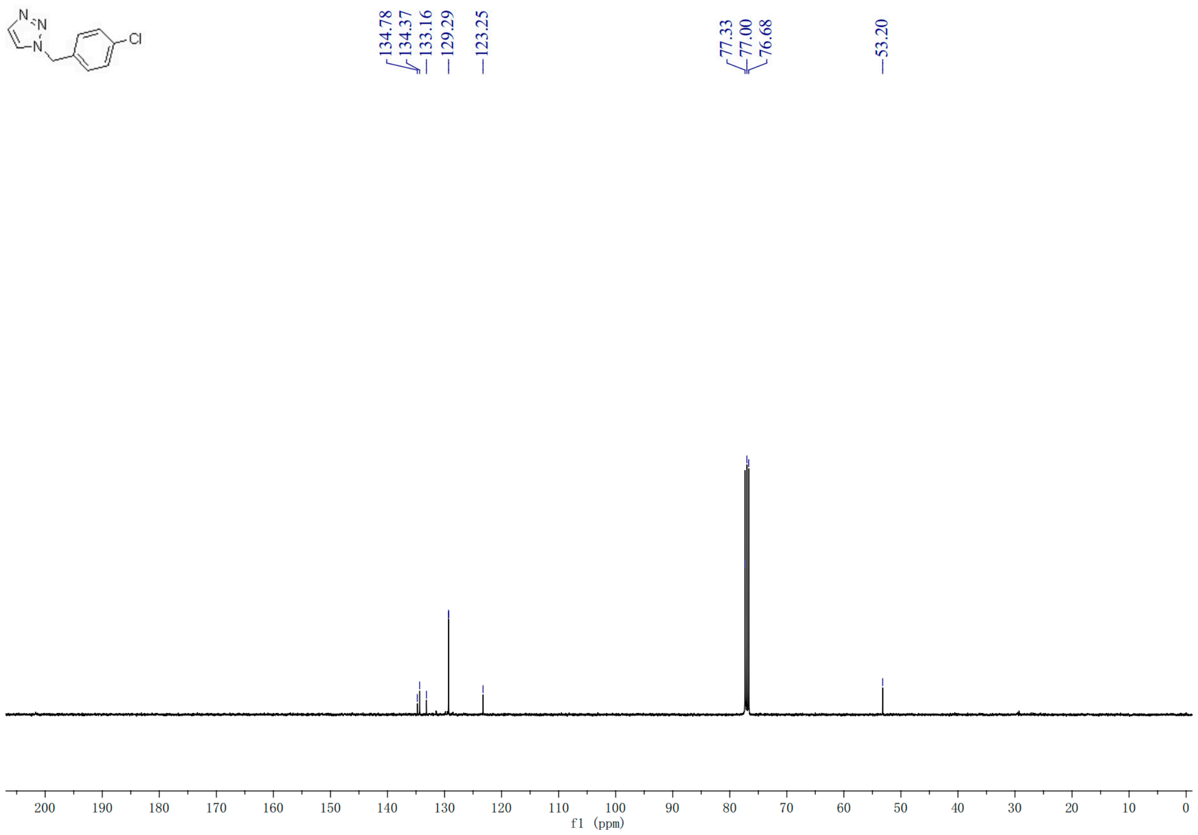
N
N
N
Cl
134.78
134.37
133.16
129.29
123.25
77.33
77.00
76.68
53.20
200 190 180 170 160 150 140 130 120 110 100 90 80 70 60 50 40 30 20 10 0
f1 (ppm)
